# An Iterative Approach Guides Discovery of the FabI Inhibitor Fabimycin, a Late-Stage Antibiotic Candidate with *In Vivo* Efficacy against Drug-Resistant Gram-Negative Infections

**DOI:** 10.1021/acscentsci.2c00598

**Published:** 2022-08-10

**Authors:** Erica N. Parker, Brett N. Cain, Behnoush Hajian, Rebecca J. Ulrich, Emily J. Geddes, Sulyman Barkho, Hyang Yeon Lee, John D. Williams, Malik Raynor, Diana Caridha, Angela Zaino, Mrinal Shekhar, Kristen A. Muñoz, Kara M. Rzasa, Emily R. Temple, Diana Hunt, Xiannu Jin, Chau Vuong, Kristina Pannone, Aya M. Kelly, Michael P. Mulligan, Katie K. Lee, Gee W. Lau, Deborah T. Hung, Paul J. Hergenrother

**Affiliations:** † Department of Chemistry and Carl R. Woese Institute for Genomic Biology, University of Illinois at Urbana−Champaign, Urbana, Illinois 61801, United States; ‡ Broad Institute of MIT and Harvard, Cambridge, Massachusetts 02142, United States; § Walter Reed Army Institute of Research, Silver Spring, Maryland 20910 United States; ∥ Department of Pathobiology, College of Veterinary Medicine, University of Illinois at Urbana−Champaign, Urbana, Illinois 61801, United States; ⊥ Department of Molecular Biology and Center for Computational and Integrative Biology, Massachusetts General Hospital, Boston, Massachusetts 02115, United States

## Abstract

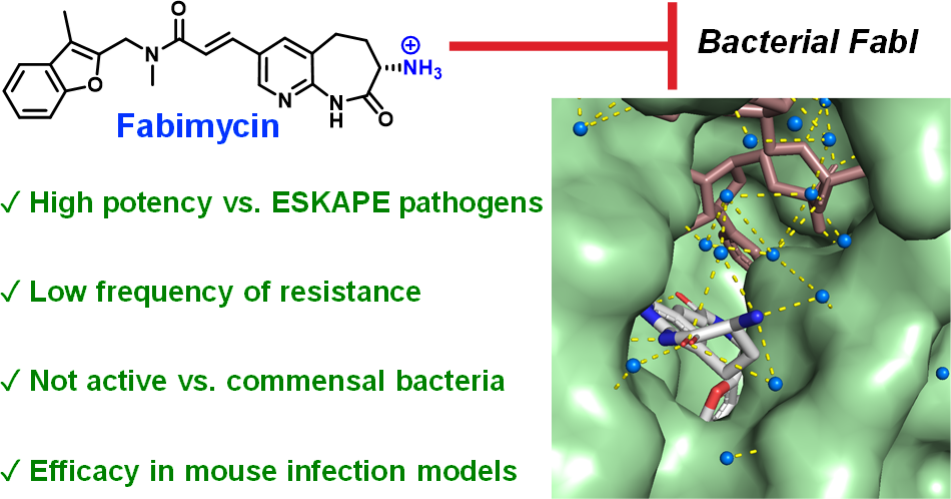

Genomic studies and experiments with permeability-deficient strains have revealed a variety of biological targets that can be engaged to kill Gram-negative bacteria. However, the formidable outer membrane and promiscuous efflux pumps of these pathogens prevent many candidate antibiotics from reaching these targets. One such promising target is the enzyme FabI, which catalyzes the rate-determining step in bacterial fatty acid biosynthesis. Notably, FabI inhibitors have advanced to clinical trials for *Staphylococcus aureus* infections but not for infections caused by Gram-negative bacteria. Here, we synthesize a suite of FabI inhibitors whose structures fit permeation rules for Gram-negative bacteria and leverage activity against a challenging panel of Gram-negative clinical isolates as a filter for advancement. The compound to emerge, called fabimycin, has impressive activity against >200 clinical isolates of *Escherichia coli*, *Klebsiella pneumoniae*, and *Acinetobacter baumannii*, and does not kill commensal bacteria. X-ray structures of fabimycin in complex with FabI provide molecular insights into the inhibition. Fabimycin demonstrates activity in multiple mouse models of infection caused by Gram-negative bacteria, including a challenging urinary tract infection model. Fabimycin has translational promise, and its discovery provides additional evidence that antibiotics can be systematically modified to accumulate in Gram-negative bacteria and kill these problematic pathogens.

## Abstract 2

Fabimycin is a bacterial FabI inhibitor that possesses potent antibiotic activity against multiple ESKAPE pathogens and efficacy in several mouse infection models, while largely sparing human commensal bacteria.

## Introduction

Novel antibiotic classes for infections caused by Gram-positive pathogens have been a success story over the last 20 years, with drugs in the oxazolidinone, mutilin, and lipopeptide classes all having notable clinical or veterinary impact.^1−3^ Further, there are additional antibiotics moving through clinical trials for Gram-positive infections, including new compound classes and antibiotics that engage unexploited biological targets.^4^ In contrast, there has not been a novel class of antibiotics FDA approved for treatment of Gram-negative pathogens contained within the group of high-priority antibiotic-resistant, nosocomoial pathogens (ESKAPE pathogens^5,6^) in over 50 years; this situation has led to increased mortality, with these Gram-negative bacteria representing four of the top six pathogens causing antibiotic-associated deaths, and some studies showing that 75% of deaths from drug-resistant pathogens are now caused by Gram-negative bacteria.^7,8^ This discovery void is largely due to the low likelihood that a given compound will accumulate inside Gram-negative bacteria, as their dense lipopolysaccharide outer membrane and promiscuous efflux pumps work in concert to prevent candidate antibiotics from reaching their target. Recent studies reveal that compounds capable of accumulating inside Gram-negative bacteria often possess certain physicochemical properties,^9−11^ explaining why high-throughput screens of millions of compounds have failed to identify Gram-negative active antibiotics.^12,13^

**Figure 1:**
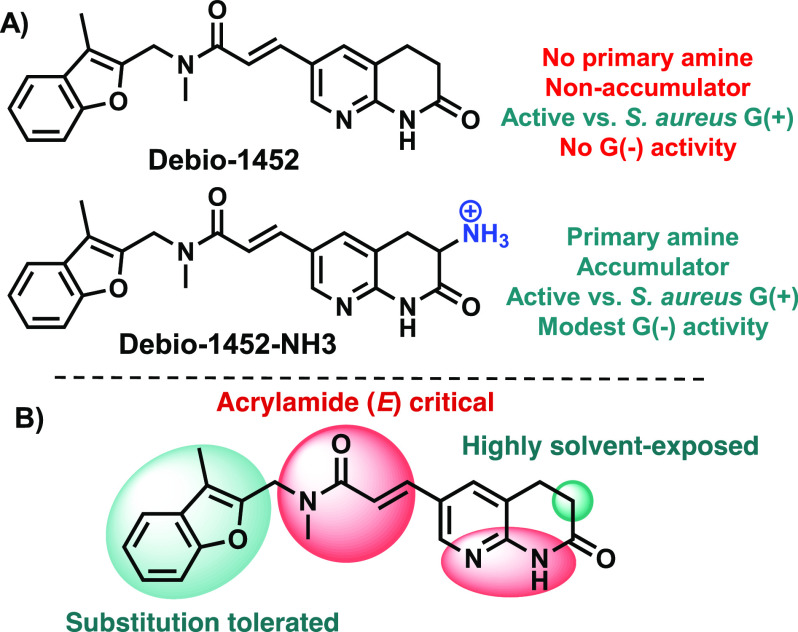
FabI inhibitors. (A) Debio-1452 is highly potent against *S. aureus*; Debio-1452-NH3 retains this potency and gains modest activity against many Gram-negative pathogens. (B) The structure–activity relationship (SAR) of Debio-1452 showing regions amenable to substitution (highlighted in green) and those critical for antibacterial activity (highlighted in red).

Encouragingly, the same biological processes that are exploited through antibiotic intervention against Gram-positive bacteria can typically be leveraged to kill Gram-negative bacteria; inhibitors of protein translation, DNA replication, and cell wall biosynthesis have broad-spectrum activity (Gram-positive and Gram-negative) provided they can enter the cell and reach their target. However, many other promising biological targets have not yet been leveraged to kill Gram-negative organisms, as the antibiotics that engage these targets do not accumulate in Gram-negative bacteria. One such outstanding target is the enoyl-acyl carrier protein reductase enzyme FabI, which catalyzes the rate-determining step in bacterial fatty acid biosynthesis.^14^ A lead compound^15,16^ identified from a biochemical high-throughput screen for FabI inhibition was optimized into Debio-1452 (Figure 1A), the phosphonoxy methyl prodrug version of which (called afabicin) is in Phase 2 clinical trials for infections caused by *Staphylococcus aureus*.^17^ While FabI is a promising exploitable target for problematic Gram-negative ESKAPE pathogens, including *Escherichia coli*, *Klebsiella pneumoniae*, and *Acinetobacter baumannii*, Debio-1452 does not accumulate inside these cells and is consequently inactive against these bacteria.^18^

We recently applied an emerging understanding of the correlation between the physicochemical traits of small molecules and their accumulation in Gram-negative bacteria to design a version of Debio-1452 that is effective against these pathogens.^19^ This compound, Debio-1452-NH3 (Figure 1A), has antibacterial activity against Gram-negative clinical isolates and efficacy in mouse infection models, and as such is the first member of this class to have notable Gram-negative antibacterial activity.^19^ In efforts to develop a more promising clinical candidate for Gram-negative infections, we now report the use of iterative compound synthesis, clinical isolate testing, and X-ray crystallography to identify fabimycin, a FabI inhibitor with enhanced antibacterial potency, improved *in vivo* tolerability, and high specificity for pathogenic versus commensal bacteria. Fabimycin shows efficacy in multiple mouse infection models, including a challenging mouse model of urinary tract infection (UTI), acute pneumonia models, and several neutropenic soft tissue models of infection with Gram-negative bacteria.

## Results

### Identification of Fabimycin and Its Activity against Drug-Resistant Gram-Negative Pathogens

Aided by recently described guidelines for design of Gram-negative penetrant compounds (the eNTRy rules^9,11,20^), Debio-1452-NH3 was discovered through a focused synthetic campaign and subsequent evaluation of just a handful of compounds.^19^ However, to advance as a lead candidate, its therapeutic window would need to be widened, as efficacy of Debio-1452-NH3 in murine infection models with Gram-negative pathogens was observed at the maximal tolerated dose (MTD).^19^ An objective was set to identify next-generation FabI inhibitors that exhibit greater potency against Gram-negative clinical isolates and better *in vivo* tolerability, expecting that such compounds could then be efficacious even in very challenging models and those of high translational relevance, such as a UTI model.

Our critical path for advancement included the synthesis of compounds and subsequent evaluation of antibacterial activity against Gram-negative clinical isolates to prioritize leads, followed by evaluation of toxicity, pharmacokinetics, and ultimately efficacy in mouse infection models. Compound design was guided by the co-crystal structure of Debio-1452 with FabI (from *S. aureus*)^21^ and the established structure–activity relationship (SAR) for this compound class, both of which point to the immutability of the *N*-methyl acrylamide (*E* configuration) and the H-bond donor/acceptor pair on the naphthyridinone ring system that interacts with key amino acid residues within the FabI active site (Figure 1B).^22−24^ In contrast, the position adjacent to the carbonyl of the naphthyridinone ring was judged to be highly solvent-exposed; additionally, other ring systems were considered as replacements for the benzofuran (Figure 1B). This analysis was instrumental for informing the proper placement of a positively charged amine to facilitate accumulation in Gram-negative bacteria but not disrupt target engagement. Given the X-ray and SAR data, and lessons learned from previous identification of Debio-1452-NH3, priority compounds were envisioned with a variety of amines and ring systems proximal to the carbonyl of the lactam.

**Figure 2:**
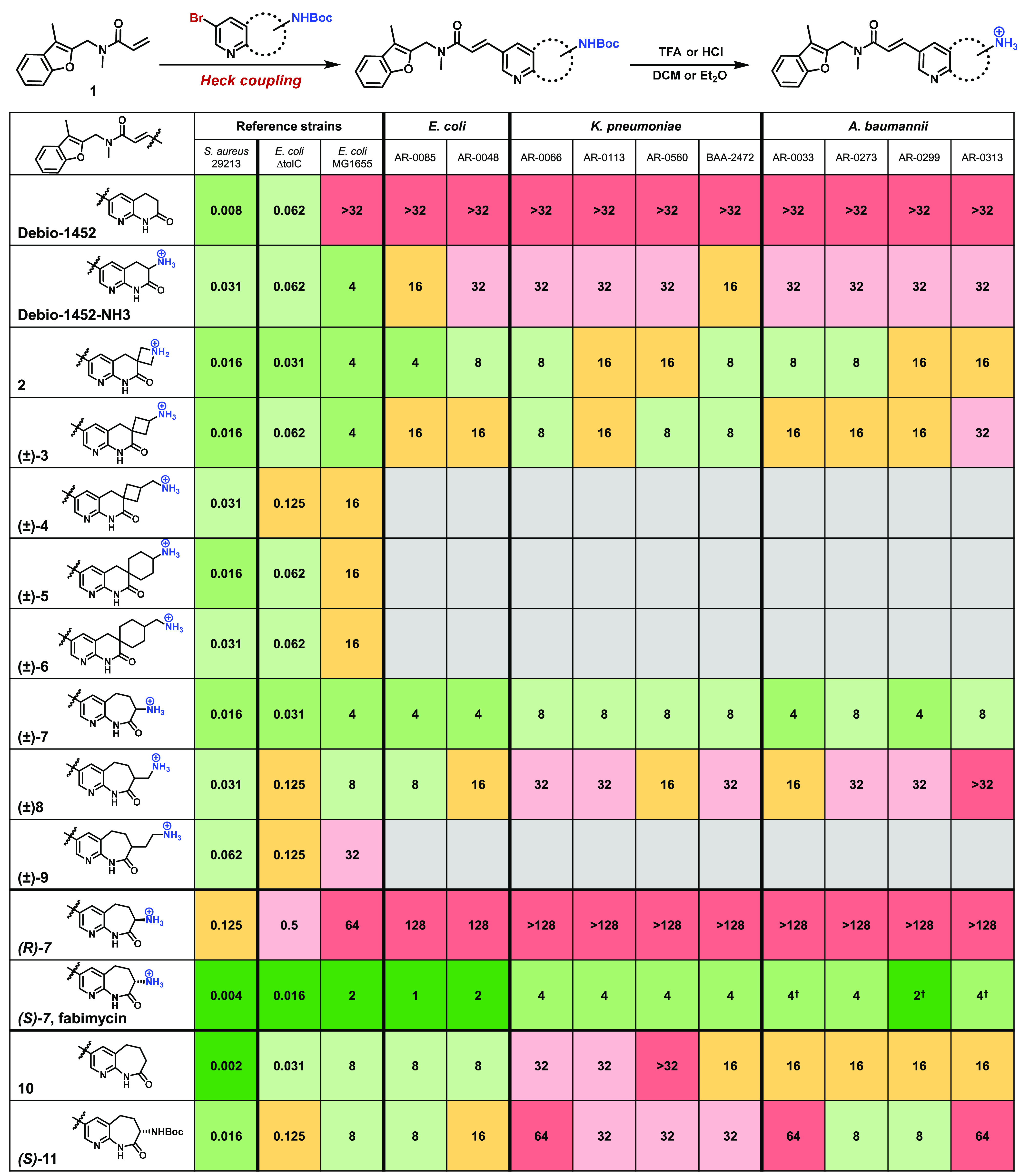
Debio-1452 analogue synthesis and antibacterial activity. The general synthetic route utilized to synthesize amine-containing compounds, and their antimicrobial activities against Gram-positive and Gram-negative bacteria. (†) indicates dose-independent trailing growth observed; see Supporting Information, Extended Data Figure S1. MIC values were determined using the microdilution broth method, as outlined by CLSI. All experiments were performed in biological triplicate. *E. coli* Δ*tolC* = JW5503.

A modular and convergent synthesis was designed leveraging a Heck coupling of acrylamide **1** with bromo functionalized acylaminopyridine rings which upon deprotection provided the final compounds (Figure 2). Brominated coupling partners were designed to give final compounds that maintained the necessary N/NH arrangement for engagement with FabI and contained the basic amine required for Gram-negative accumulation. To quickly interrogate the solvent-exposed region of the scaffold adjacent to the lactam carbonyl, ring size and shape were altered. Specifically, a series comprising four-membered ring spirocycles (**2**–**4**), six-membered ring spirocycles (**5**, **6**), and azepanones (**7**–**9**) was constructed.

The compounds synthesized, shown in Figure 2, were evaluated against Gram-positive and Gram-negative reference strains (*S. aureus* ATCC 29213 and *E. coli* MG1655, respectively), as well as an efflux-deficient *E. coli* strain (Δ*tolC*, JW5503). While compounds (*±*)-**4**, (*±*)-**5**, (*±*)-**6**, and (*±*)-**9** all had reduced antibacterial activity against *E. coli* MG1655 relative to Debio-1452-NH3, compounds **2**, (*±*)-**3**, (*±*)-**7**, and (*±*)-**8** maintained good activity (Figure 2) and were thus chosen for further evaluation. A panel of 10 clinical isolates was selected where Debio-1452-NH3 had previously demonstrated only minimal potency (MIC values of 16 or 32 μg/mL), including two *E. coli*, four *K. pneumoniae*, and four *A. baumannii* strains.^19^ As expected, Debio-1452 has no activity against this Gram-negative “challenge panel”, and Debio-1452-NH3 has only minimal activity (Figure 2). Assessment of **2**, (*±*)-**3**, (*±*)-**7**, and (*±*)-**8** revealed more potent activity for all four compounds relative to Debio-1452-NH3, with the ε-caprolactam (*±*)-**7** emerging as a promising candidate. This compound possessed superior activity against the clinical isolate challenge panel with all strains inhibited at ≤8 μg/mL. Two derivatives were constructed where the amine-decorated ε-caprolactam was coupled to alternative ring systems, substituting out the benzofuran, but neither of these compounds provided an improvement in activity (Extended Data, Figure S2).

The two enantiomers of (*±*)-**7** were separated by chiral preparatory HPLC, and biological assessment revealed the (−) enantiomer to possess significantly greater antibacterial activity than the (+) enantiomer. X-ray crystallography studies, described later herein, were used to determine that the highly active (−) enantiomer possesses the *S* stereochemical configuration. As shown in Figure 2, **(*****S*****)-7**, coined fabimycin,^25–265^ has outstanding activity against *S. aureus* and *E. coli* Δ*tolC*, an MIC value of 2 μg/mL against *E. coli* MG1655, and very good activity against the clinical isolate challenge panel, whereas **(*****R*****)-7** is significantly less active.

To probe the influence of the amine on antibacterial activity and compound accumulation, two additional derivatives were synthesized: compound **10**, with the expanded ring system but lacking the amine, and compound (***S***)-**11**, a carbamylated version of fabimycin. Of note, **10** and (***S*****)-11** both lack a primary amine. While both compounds display modest inhibition of purified bacterial FabI (Extended Data, Figure S3) and excellent antimicrobial activity against reference strains, they have diminished activity against Gram-negative clinical isolates (Figure 2). Furthermore, when assessed in a whole-cell accumulation assay^27^ in *E. coli*, **10** displayed reduced whole-cell accumulation consistent with its reduction in antibacterial activity (Extended Data, Figure S4). In contrast, other amine-containing compounds in Figure 2 show significant whole-cell accumulation in *E. coli* (Extended Data, Figure S4) (the limited aqueous solubility of (***S***)-**11** prevented it from being assessed in this assay).

An interesting aspect of FabI as an antibacterial target is the possibility for a relatively narrow spectrum of activity when compared to that observed with most antibiotics (e.g., inhibitors of protein synthesis, DNA replication, and/or cell wall biosynthesis). For example, it has been noted that ESKAPE pathogen *P. aeruginosa* possesses the FabV isoform, meaning inhibition of FabI will not be lethal in this pathogen.^28^ Certain Gram-positive pathogens (*Streptococcus sp.* and *Enterococcus sp.*) and commensal bacteria are also not reliant on FabI and thus are expected to be insensitive to FabI inhibition, suggesting that FabI inhibitors could be microbiome-sparing.^29−31^ To explore this possibility, fabimycin was assessed against a panel of pathogens possessing alternate enoyl-acyl carrier protein reductases as well as anaerobic human commensal bacterial species.^32−34^ In all cases, low/no antibacterial activity was observed against the strains assessed (*n* = 41), demonstrating the specificity of this FabI inhibitor and its potential for minimal perturbation of the microbiome (Table 1).

**Table 1:**
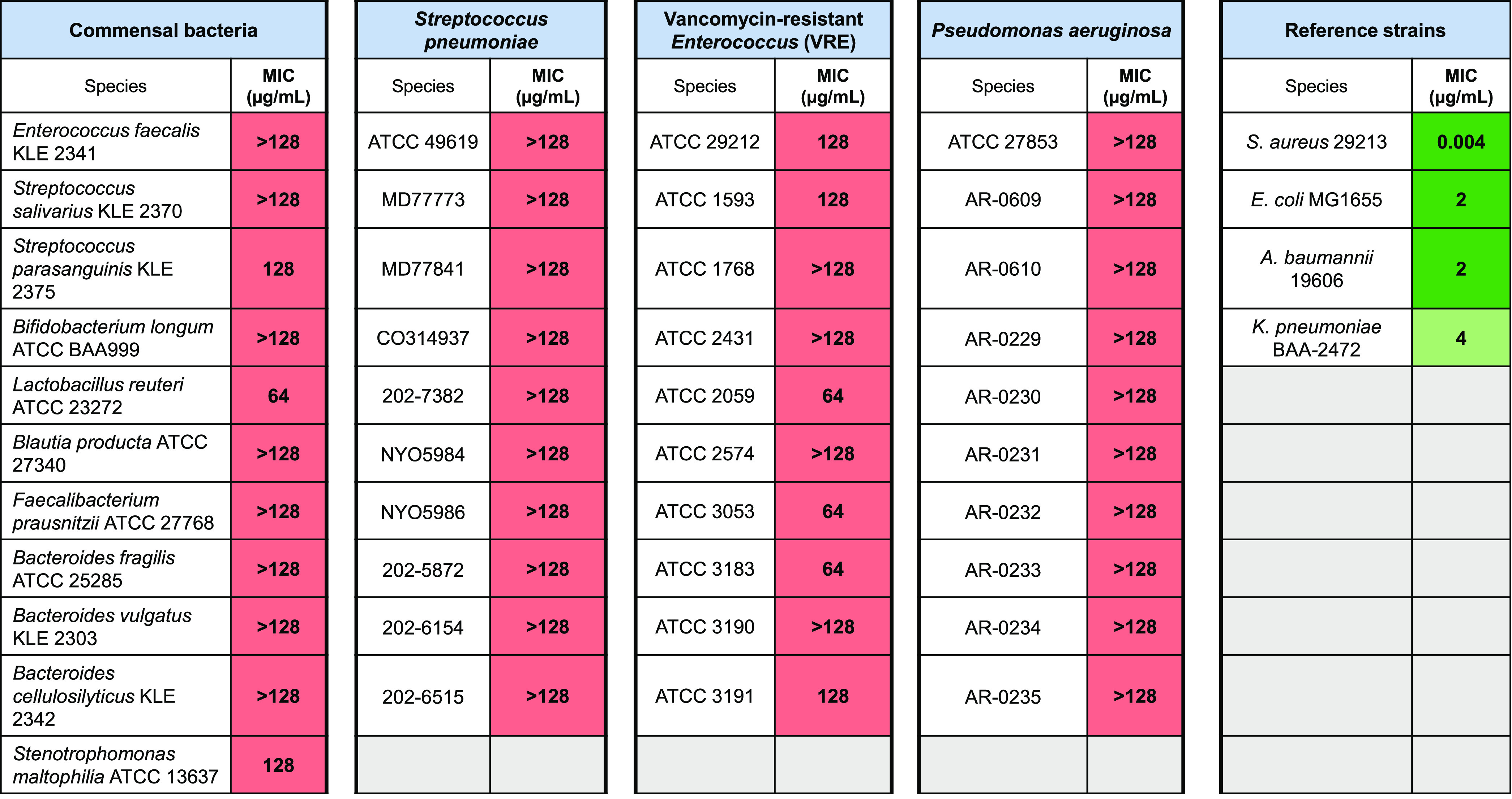
Spectrum of Fabimycin Activity^a^

a MIC experiments with commensal bacteria and pathogenic bacteria possessing alternate enoyl-acyl carrier protein reductases^28,30,35^ indicate these strains are not susceptible to fabimycin.

**Figure 3:**
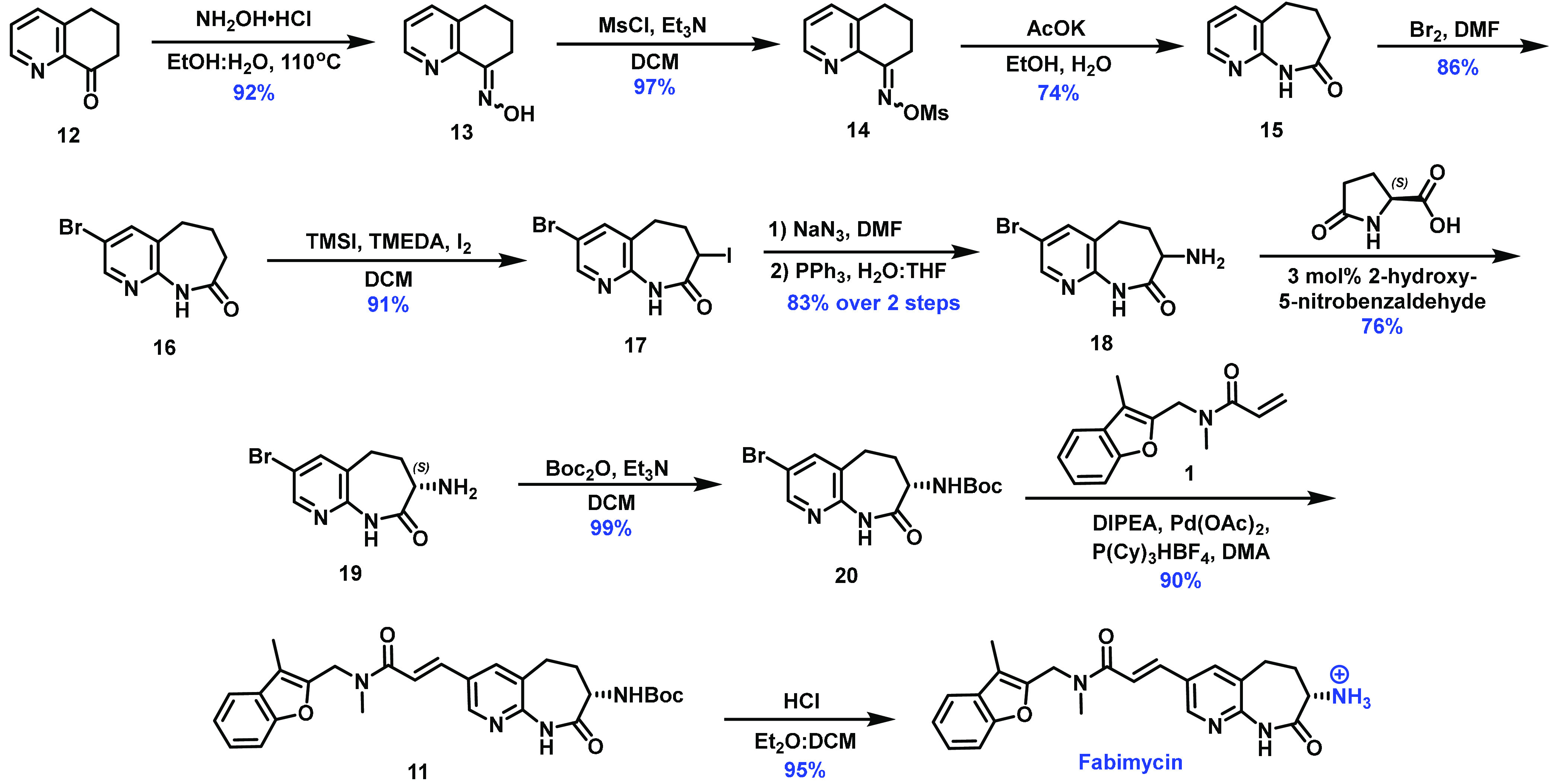
Optimized synthesis of fabimycin. The synthetic route used to access gram-scale quantities of fabimycin, utilizing dynamic kinetic resolution (DKR) to install the critical stereogenic center.

Given its promising antibacterial activity, fabimycin was advanced through a battery of mechanistic and translational experiments. While separation of (*±*)-**7** on a preparative chiral column was suitable to obtain the quantities of fabimycin required for studies such as MIC assays, it was necessary to optimize the synthetic route to enable scale-up and obtain quantities of the fabimycin required for detailed *in vivo* tolerability, pharmacokinetic analysis, and efficacy experiments. To this end, the synthetic route shown in Figure 3 was developed and employed to generate gram-scale quantities of enantiopure fabimycin.

The route proceeds through condensation of quinolone **12** with hydroxylamine, followed by activation of the resulting oxime (**13**) via treatment with mesyl chloride to generate compound **14** in good yield. After forming azepanone **15** through a Beckmann rearrangement, molecular bromine was used to produce aryl bromide **16**. Alpha-iodination of **16** provided dihalogenated **17** in good yield, and compound **17** was subjected to azidation and subsequent Staudinger reduction to afford amine **18**. This amine was the key intermediate for enantioenrichment via a dynamic kinetic resolution (DKR), proceeding through epimerization of the amine via imine formation with 2-hydroxy-5-nitrobenzaldehyde followed by selective crystallization with l-pyroglutamic acid.^36^ Optimization of this critical step involved significant screening (Extended Data, Figure S5). After enantioenrichment (up to 99.5% *ee*), amine **19** was protected to afford compound **20** (*ee* maintained) and used in a Heck coupling with acrylic amide **1** to produce **11**, which, when subjected to acidic conditions, liberated fabimycin.

**Figure 4:**
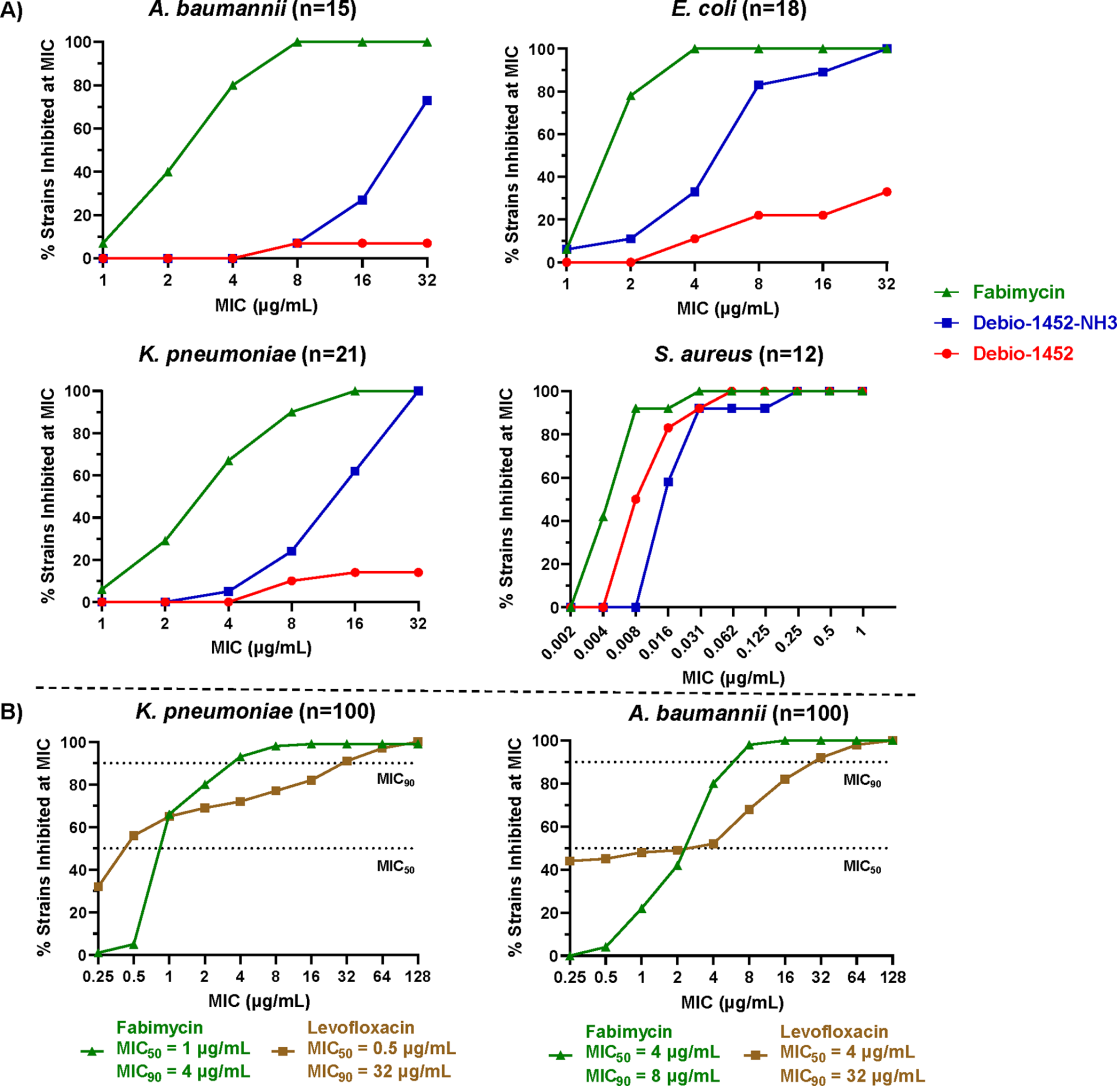
Antimicrobial activity of fabimycin against clinical isolates. (A) The susceptibility of clinical isolates (Gram-negative species and *S. aureus*) to fabimycin, Debio-1452-NH3, and Debio-1452. MICs performed in biological triplicate. (B) Further exploration of the breadth of fabimycin’s antibacterial activity against diverse clinical isolate panels of *K. pneumoniae* and *A. baumannii*, as compared to levofloxacin. MICs performed in biological duplicate.

Fabimycin was assessed for its antibacterial activity against a panel of multidrug-resistant *A. baumannii*, *E. coli*, and *K. pneumoniae* clinical isolates (54 strains in total). As shown in Figure 4A, fabimycin is markedly more potent than Debio-1452 and Debio-1452-NH3 against all the Gram-negative clinical isolates. All three compounds also maintain high potency versus panels of *S. aureus* clinical isolates (Figure 4A and Extended Data, Figure S6).

As the greatest improvement in activity of fabimycin (relative to Debio-1452-NH3) was against *K. pneumoniae* and *A. baumannii* clinical isolates, fabimycin was further assessed against more diverse and expansive clinical isolate panels of these two pathogens to determine the MIC_50_ and MIC_90_ values. Excitingly, when assessed against a panel of 100 *K. pneumoniae* clinical isolates, fabimycin inhibited 90% of the strains at 4 μg/mL, relative to 32 μg/mL for levofloxacin (Figure 4B). Also encouraging was the narrow MIC range for fabimycin, suggesting that intrinsic resistance to this compound is not prevalent in existing bacterial populations. The analogous data in 100 *A. baumannii* clinical isolates, a panel specifically curated to represent the genomic diversity of the species^37^ including 27 multidrug-resistant strains, 35 extensively drug-resistant strains, and 1 pan-resistant strain (fabimycin MIC = 1 μg/mL), are also promising, with fabimycin exhibiting a MIC_90_ value of 8 μg/mL (relative to 32 μg/mL for levofloxacin) and a narrow distribution of MIC values (Figure 4B).

### Mode of Action

**Figure 5:**
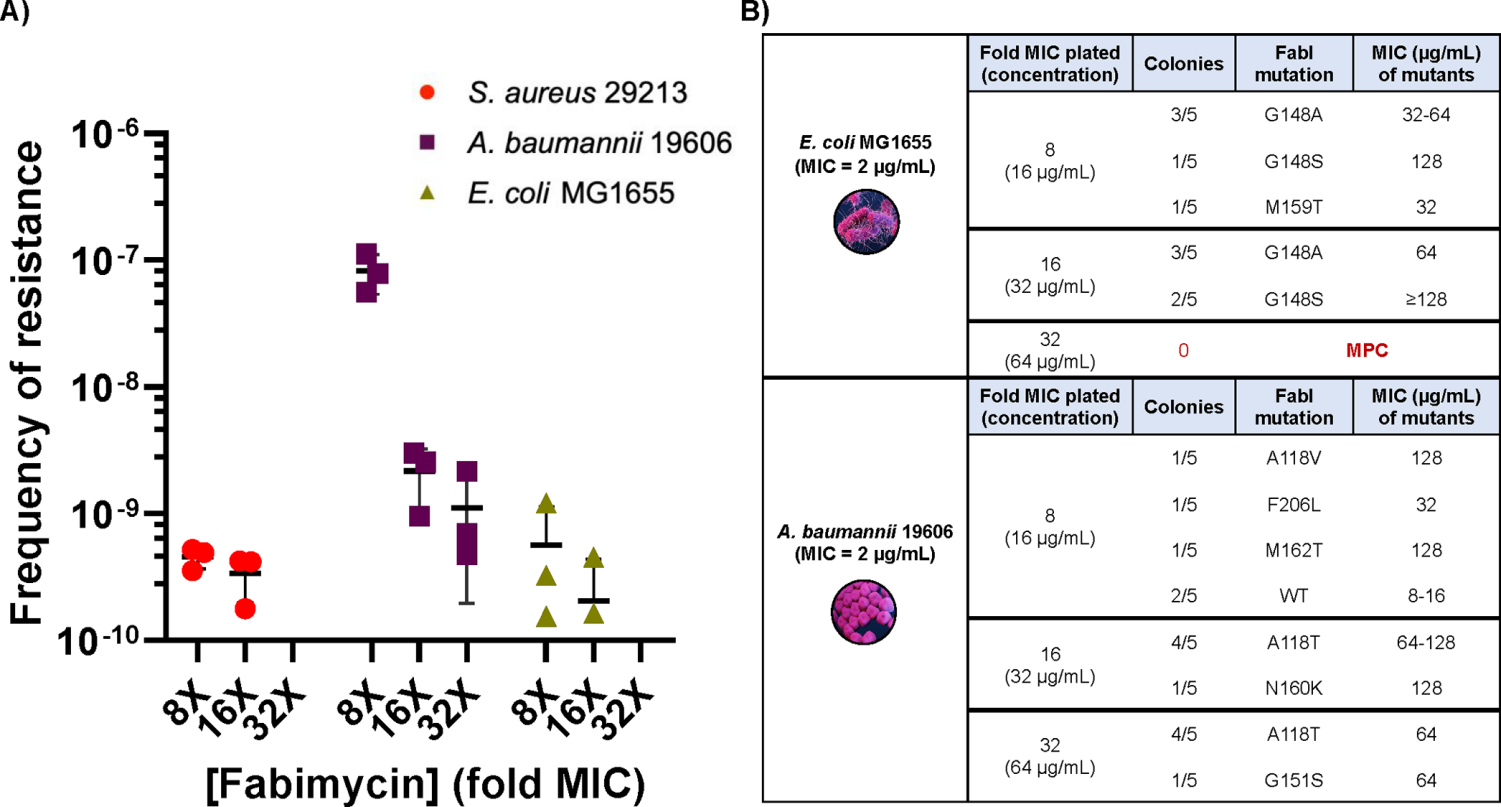
Fabimycin mode of action studies. (A) Spontaneous resistance frequencies of *S. aureus*, *E. coli*, and *A. baumannii* versus fabimycin. Data represent three replicates for each pathogen with error bars representing the SEM. (B) Point mutations in FabI observed in fabimycin-resistant colonies, and the corresponding MIC values of fabimycin versus the mutants. All MICs were performed in biological triplicate.

Spontaneous resistant mutants to fabimycin were generated in *E. coli* MG1655, *A. baumannii* 19606, and *S. aureus* ATCC 29213 at 8×, 16×, and 32× the respective MICs with low frequencies of resistance observed at 8×–16× the MIC for all pathogens (Figure 5A). Importantly, sequencing of the *fabI* gene in resistant colonies revealed mutations encoding single amino acid changes within the active site of FabI (Figure 5B). While several FabI mutations were observed in fabimycin-resistant *A. baumannii*, the MIC of the bacteria harboring mutant FabI was often near fabimycin concentrations attained *in vivo* (as shown later). In *E. coli*, the most frequently observed FabI mutation to arise in fabimycin-resistant colonies was at G148 (Figure 5B); *E. coli* with mutations at this position in FabI have been previously shown to have attenuated fitness.^19^ Encouragingly, the mutant prevention concentration (MPC, the concentration where no resistant mutants are observed) of fabimycin versus *E. coli* was found to be 64 μg/mL and, impressively, in *S. aureus*, the MPC was 0.125 μg/mL. A serial passaging experiment of *E. coli* MG1655 in the presence of a subinhibitory concentration of fabimycin over the course of 21 days led to an 8-fold increase in the MIC for fabimycin, relative to a 128-fold increase in the MIC of ciprofloxacin in the same experiment (Extended Data, Figure 7A). To investigate the rate of killing, a time–kill growth curve was generated that revealed fabimycin slowly kills *E. coli* over the course of 8 h (Extended Data, Figure 7B); however, fabimycin was not profoundly bactericidal in this experiment, mimicking its progenitor’s (Debio-1452) behavior in *S. aureus*.^21^

### Crystallography, Molecular Dynamics, and Biophysics

An interesting feature of fabimycin is its considerably enhanced antibacterial activity relative to its enantiomer **(*****R*****)-7**. Assessment in *E. coli* reveals that each enantiomer accumulates intracellularly to a similar extent (Extended Data, Figure S4). In contrast, evaluation of the two enantiomers in the FabI activity assay shows a significant differential between the two enantiomers (Extended Data, Figure S8), with fabimycin being at least five-fold more potent against *A. baumannii* and *E. coli* FabI. Of note, in this enzyme assay IC_50_ values cannot be accurately determined below ∼10 nM, so the value for fabimycin likely under-represents its biochemical potency. Taken together, the data suggest that the diminished antibacterial activity of **(*****R*****)-7** relative to fabimycin is due to reduced target engagement and not differential intracellular accumulation.

**Figure 6:**
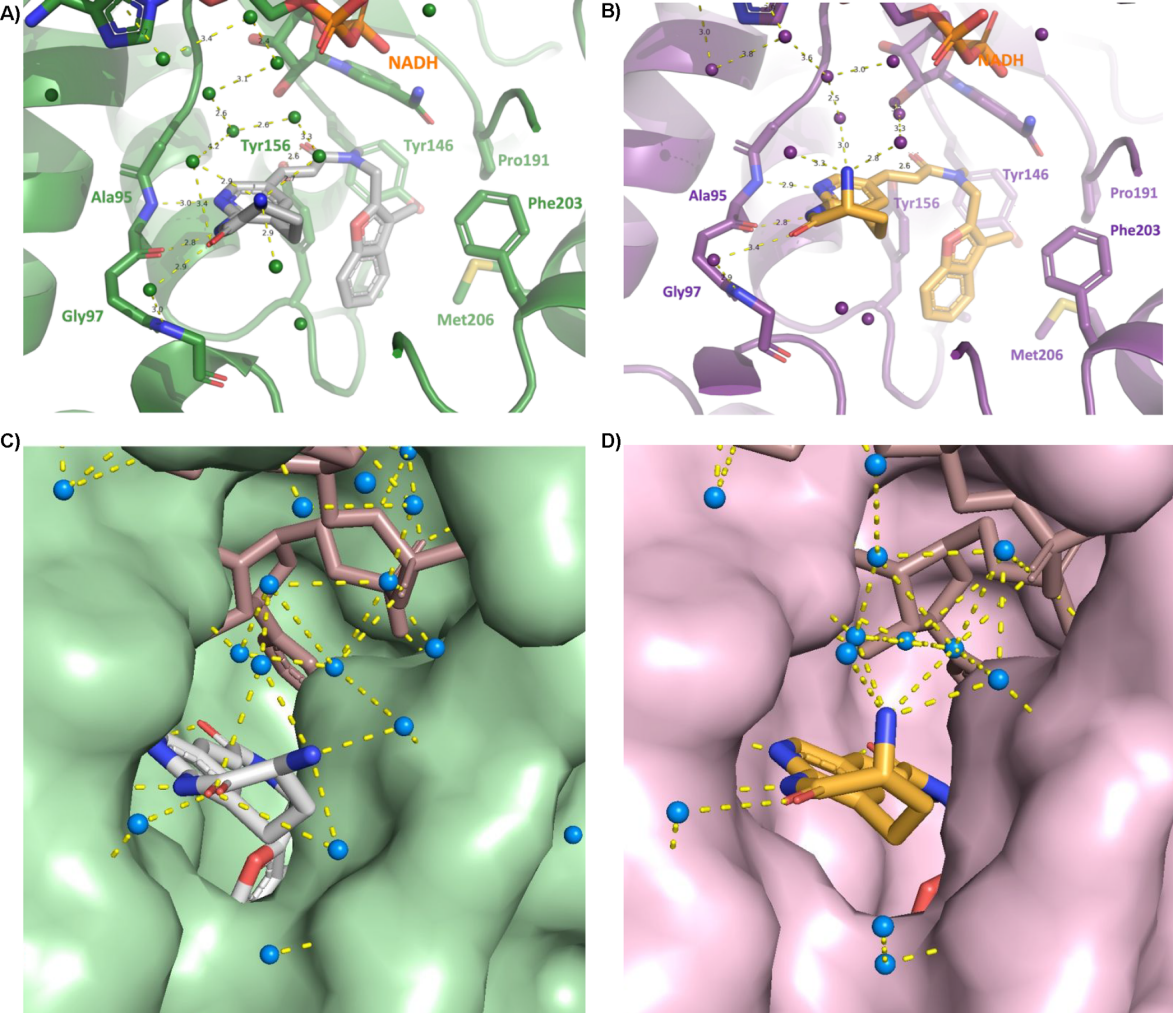
Co-crystal structures of fabimycin and its enantiomer with FabI. (A) Co-crystal structure of fabimycin with *E. coli* FabI with NADH cofactor (PDB 7UMW). (B) Co-crystal structure of **(*****R*****)-7** in *E. coli* FabI with NADH cofactor (7UM8). (C) Water network surrounding fabimycin in the *E. coli* FabI active site. (D) Water network surrounding **(*****R*****)-7** in the *E. coli* FabI active site.

To further investigate the molecular basis for the observed differential activity between enantiomers, X-ray crystal structures of fabimycin and its enantiomer **(*****R*****)*****-*****7** bound to both *E. coli* and *A. baumannii* FabI with NADH cofactor were solved with resolutions ranging from 1.5 to 2.7 Å. The general binding mode of the compounds is similar to what has been previously reported for this class of compounds and Debio-1452 in particular.^38^ In complex with *E. coli* FabI, the pyridoazepanone ring forms two hydrogen bonds with the backbone carbonyl and amide nitrogen of conserved residue Ala95 (Figure 6A,B). A water-mediated interaction is also observed between the carbonyl of the lactam and the backbone amide of Gly97. Additionally, a hydrogen bond is formed between the acrylamide linker carbonyl and the conserved residue Tyr156. The benzopyran ring is nestled in a hydrophobic pocket formed by conserved, hydrophobic residues Tyr146, Pro191, Ile153, Met206, and Phe203.

Similar to *E. coli*, the hydrogen-bonding network of the pyridoazepanone ring, interactions of the acrylamide carbonyl, and the position of the flanking benzofuran ring are maintained with the analogous amino acid residues in *A. baumannii* FabI (Extended Data, Figure S9A,B). While no significant changes of the interacting residues can be seen between fabimycin and **(*****R*****)-7** in *A. baumannii* FabI, the enhanced resolution of the *E. coli* crystal structures captures a more nuanced and detailed binding mode. In the *E. coli* structure, a large water network is observed between the ligand, NADH cofactor, and FabI enzyme. While this network exists in the binding of both enantiomers, it is larger and more tightly structured in the fabimycin co-crystal (see Figure 6C,D).

**Figure 7:**
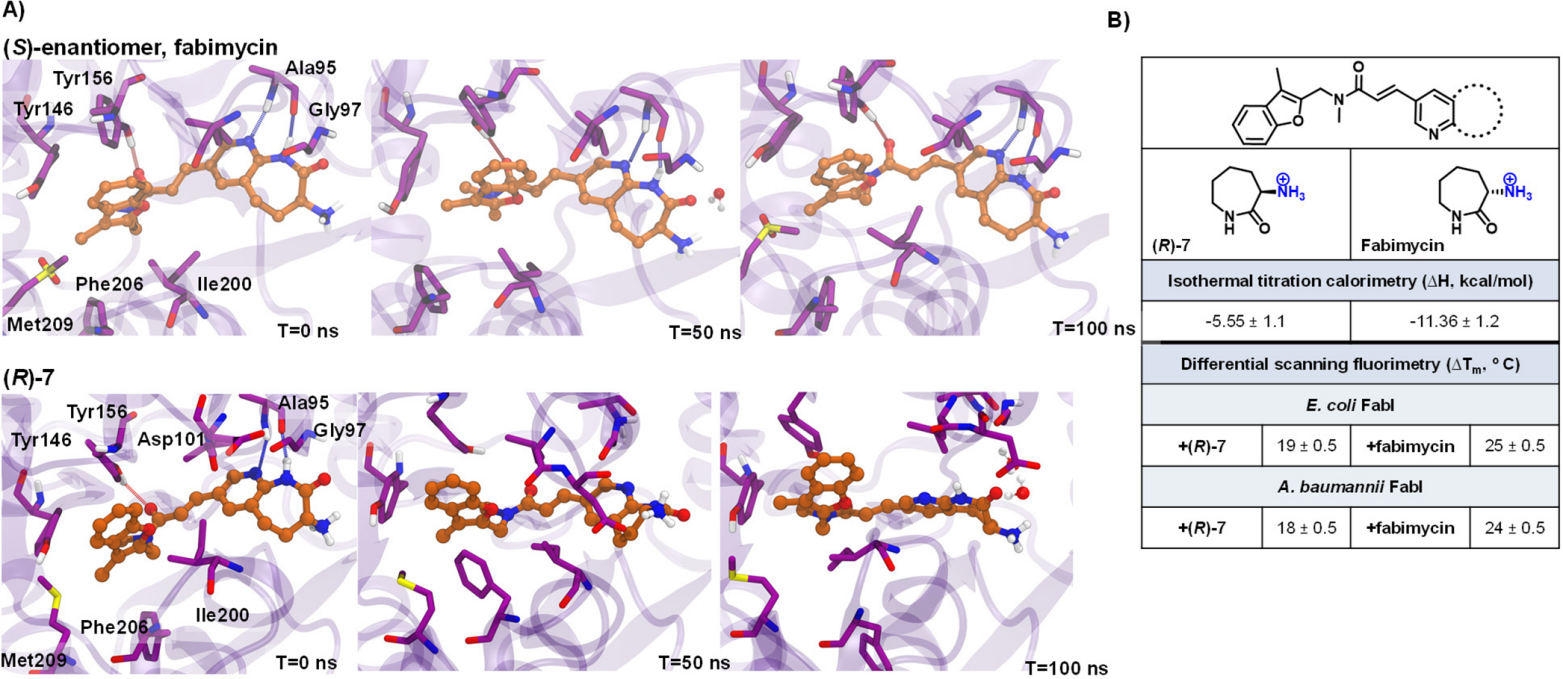
Computational and biophysical evaluation of fabimycin and its enantiomer. (A) Molecular dynamic simulations of fabimycin and its enantiomer using the co-crystal structures in *E. coli* FabI, demonstrating the enhanced flexibility (decreased stability) of **(*****R*****)-7**. (B) The determined enthalpy changes upon binding to FabI as assessed by isothermal titration calorimetry (ITC), as well as observed stabilization in a differential scanning fluorimetry assay of FabI (from *E. coli* and *A. baumannii*) upon compound binding relative to the holoenzyme. *T*_m_ values are means of technical triplicates with error shown as the standard deviation.

Intrigued by the relative similarity of the crystal structures, experiments were conducted to evaluate the stability and dynamics of the inhibitor-enzyme complex as well as the strain energy of each enantiomer. The established *E. coli* co-crystal structure was utilized for computational studies which determined that the less active **(*****R*****)**-enantiomer is more strained relative to fabimycin (∼8.30 kcal/mol higher) in the active site. This is reflected in molecular dynamic simulations that reveal the **(*****R*****)**-enantiomer to be much more flexible in the binding pocket of FabI leading to overall less-productive hydrogen-bond interactions with critical surrounding residues such as Ala95 and Tyr156 (Figure 7A). Isothermal titration calorimetry (ITC) was used to confirm the nanomolar potency of each compound and showed a doubling in enthalpy for fabimycin relative to the less active enantiomer (Figure 7B). Finally, differential scanning fluorimetry (DSF) experiments show that fabimycin enhances the stability of the enzyme–inhibitor complex significantly more than the less active enantiomer in both *E. coli* and *A. baumannii* versions of FabI (Figure 7B).

### *In Vivo* Experiments

Given the low resistance frequency and promising data with >200 Gram-negative clinical isolates, experiments were conducted to probe the suitability of fabimycin for *in vivo* infection models. As a prelude to these studies, fabimycin was evaluated against three human cell lines (HFF-1, A549, HepG2) and for its ability to lyse human red blood cells. These studies revealed fabimycin to be less cytotoxic relative to Debio-1452-NH3 and nonhemolytic even at high concentrations (200 μM, Extended Data, Table S1). Fabimycin also has lower activity against the hERG ion channel relative to both Debio-1452 and Debio-1452-NH3, and lower plasma protein binding relative to Debio-1452 (Extended Data, Table S1). Formulation and MTD studies were performed, and in all cases fabimycin was found to be better tolerated in mice with an MTD of >200 mg/kg (IP injection) relative to 50 mg/kg for Debio-1452-NH3 (Extended Data, Table S2).

**Figure 8:**
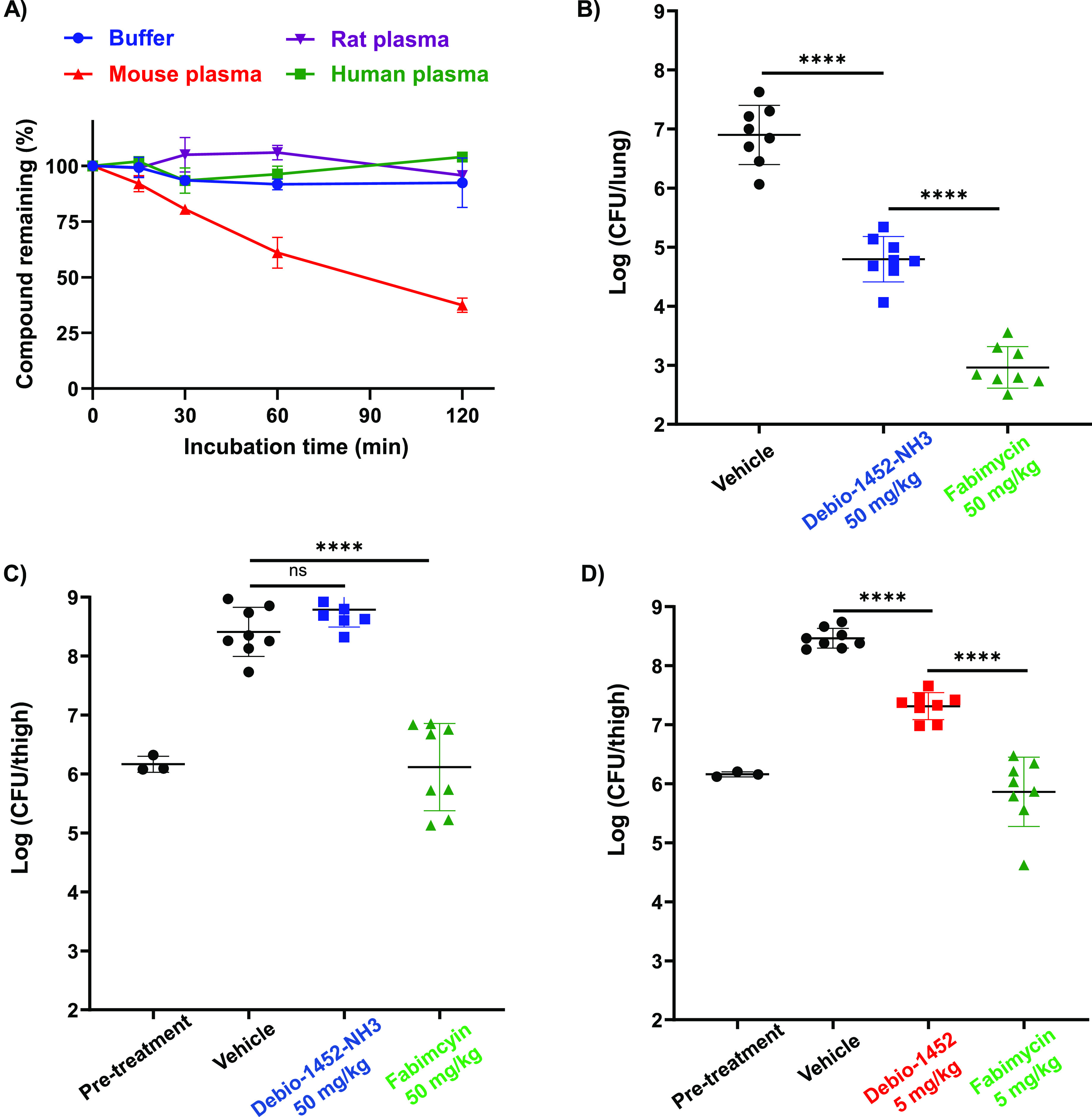
Plasma stability and *in vivo* efficacy of fabimycin. (A) Assessment of fabimycin stability in plasma. Data shown as the mean and standard deviation from two experiments. (B) Acute pneumonia infections initiated in CD-1 mice with *A. baumannii* AR-0299 (1.6 × 10^8^ CFUs per mouse intranasally). Mice were treated with vehicle (8 mice) or FabI inhibitor (8 mice per group) 4, 23, and 41 h postinfection (50 mg/kg intramuscular) and the bacterial burden evaluated at 48h postinfection. (C) Neutropenic mouse thigh infection initiated in CD-1 mice with *A. baumannii* AR-0299 (1.22 × 10^6^ CFUs per mouse intramuscular in thigh) were treated with vehicle (8 mice) or FabI inhibitor (8 mice per group) 2, 6, and 11 h postinfection (50 mg/kg intramuscular), and the bacterial burden was evaluated 26h postinfection. (D) Neutropenic mouse thigh infection initiated in CD-1 mice with *S. aureus* USA300 LAC (2.3 × 10^6^ CFU per mouse intramuscular in thigh) were treated with vehicle (eight mice) or FabI inhibitor (eight mice per group) 2 and 7 h postinfection (5 mg/kg retro-orbital IV), and the bacterial burden was evaluated 24 h postinfection. Debio-1452-tosylate used. FabI inhibitors formulated with 20% SBE-β-CD in H_2_O. In B, C, and D statistical significance was determined by one-way ANOVA with Tukey’s multiple comparisons. NS, not significant. ****P < 0.0001. Error bars represent standard deviation.

An interesting aspect of this compound class is the metabolic instability in mice leading to suboptimal pharmacokinetics;^24^ indeed, assessment of fabimycin in mouse, rat, and human plasma showed considerable instability in mouse plasma contrasted with excellent stability in rat and human plasma (Figure 8A). While this data suggests the possibility that antibacterial activity could improve as fabimycin moves toward humans, it does complicate the evaluation of this compound class in murine infection models. Thus, as a prelude to efficacy experiments in mouse infection models, a pharmacokinetic study was conducted with fabimycin using neutropenic female BALB/c mice infected with drug-resistant *A. baumannii* (fabimycin MIC = 2 μg/mL) where single doses of the compound (20, 50, 75, 100 mg/kg) were administered intravenously to the infected mice and blood taken over the course of 8 h. Encouragingly, when dosed at 75 mg/kg, fabimycin concentrations stayed above the MIC for the infectious strain for over 6 h in the thigh tissue with the *C*_max_ nearing the MPC for wild-type *E. coli* when dosed at 100 mg/kg (Extended Data, Figure S10).

With formulation, MTD, and pharmacokinetic data in hand, the efficacy of fabimycin was evaluated in murine infection models. To start, a comparative assessment was made of fabimycin and Debio-1452-NH3 in two murine infection models using a dosing regimen (50 mg/kg, intramuscular) near the MTD for Debio-1452-NH3 but well below the MTD for fabimycin. Using an extensively drug-resistant *A. baumannii* clinical isolate, fabimycin outperformed Debio-1452-NH3 in both lung and neutropenic thigh infection models and achieved a >3-fold decrease in log(CFU/lung) and >2-fold decrease log(CFU/thigh) relative to the vehicle (Figure 8B,C). In a *S. aureus* (MRSA clinical isolate) neutropenic thigh infection model fabimycin showed significantly greater reduction of bacterial burden relative to Debio-1452 when both were administered at the low dose of 5 mg/kg (Figure 8D). As the goal of these initial models was simply to assess efficacy compared to progenitor compounds, fabimycin dosing was not maximized or optimized. To address this, fabimycin was evaluated in an experiment where neutropenic, *A. baumannii-*infected mice were treated four times a day (via IV injection) with doses spanning 1.25–75 mg/kg. In this experiment, significant dose-dependent responses were observed beginning at 30 mg/kg (Extended Data, Figure S11A). When using the most efficacious dose (75 mg/kg, four times a day) in a murine neutropenic thigh infection experiment with an extensively resistant NDM-1 containing strain of *A. baumannii*, fabimycin was able to reduce bacterial burden by nearly 2 log(CFU/thigh) (Extended Data, Figure S11B).

**Figure 9:**
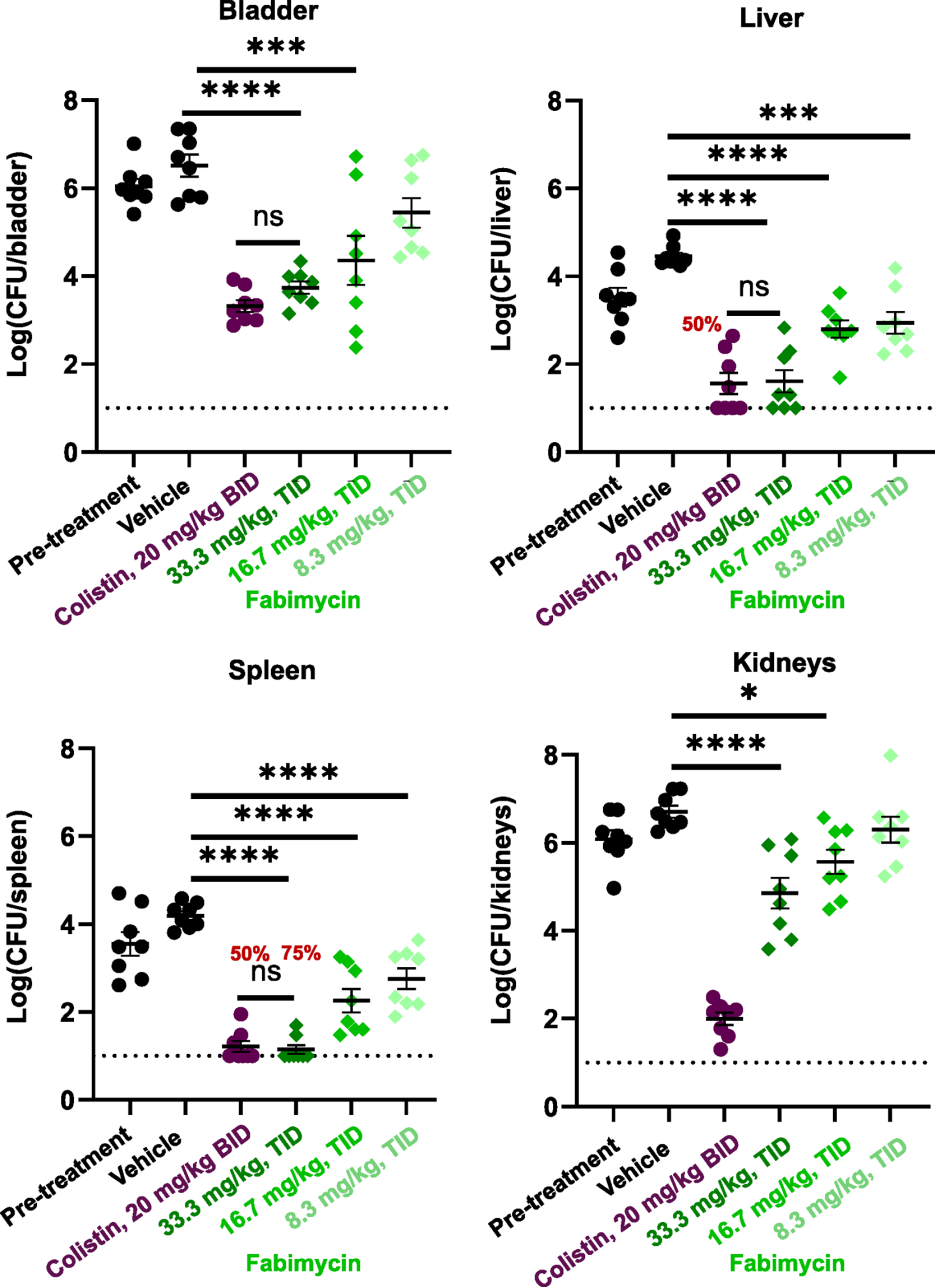
*In vivo* efficacy of fabimycin in a murine UTI model. After inducing diuresis, infection initiated in C3H/HeJ mice (8 per arm, 1.38 * 10^9^ CFU/mouse transurethral) with *E. coli* AR-0055 and treated with fabimycin (IV) at varying concentrations three times daily with bacterial enumeration at 168h postinfection. Fabimycin formulated in 17% Cremophor EL, 3% SBE-β-CD in H_2_O which was the formulation used in the vehicle arm (administered intravenously on the same schedule as fabimycin). Colistin was formulated in H_2_O with 0.9% NaCl and administered subcutaneously. Percentage in red indicates the percentage of animals with bacterial counts below the limit of detection (LOD, indicated by the dotted horizontal line). In A—D statistical significance was determined by one-way ANOVA with Tukey’s multiple comparisons. NS, not significant. *P = 0.0243, ***P < 0.001, ****P < 0.0001. Data represented as the mean with s.e.m.

With an effective dose established, we aimed to evaluate fabimycin in a murine model of an infection with high translational value. Urinary tract infections (UTIs) represent one of the biggest risks for healthy individuals in terms of exposure to antibiotic-resistant bacteria with many individuals contracting one in their lifetime (roughly 1 in 2 women and 1 in 10 men);^39^ UTIs caused by Gram-negative pathogens, particularly those that are drug-resistant, are becoming more frequent and remain a major clinical challenge.^39−41^ As *E. coli* is the causative agent in the vast majority of UTIs,^42^ fabimycin was evaluated in a murine UTI model with a challenging, extensively drug-resistant strain of carbapenem-resistant *E. coli* (fabimycin MIC = 2 μg/mL). When intravenously dosed at 33.3 mg/kg, three times a day, fabimycin was able to achieve 3.0, 2.8, 2.9, and 1.9 log_10_ reductions in bacterial load relative to the vehicle in the spleen, bladder, liver, and kidney tissues, respectively (Figure 9). Dose fractionation studies in infected mice also revealed other dosing regiments to be effective in reducing the bacterial load to a significant extent in mouse tissue (Figure 9 and Extended Data, Figure S12).

## Discussion

There is a rich history in antibacterial drug discovery of identifying and advancing chemical matter arising from whole-cell phenotypic screens. In contrast, while biochemical screens have uncovered a wealth of scaffolds with promising activity, there has typically been less interest in such compounds due to the documented inability to imbue them with whole-cell antibacterial activity.^12,13^ In this context, Debio-1452 is an interesting case study as it was developed from a progenitor compound that had only biochemical FabI inhibitory activity—it was first converted into a *S. aureus-*specific drug (Debio-1452), then to Debio-1452-NH3, and now into fabimycin, which possesses promising activity against important Gram-negative pathogens.

Relative to Debio-1452-NH3, fabimycin has significantly improved activity against *A. baumannii*, even surpassing the potency increase observed versus *E. coli*, consistent with the notion that certain FabI inhibitors can be tuned for specific bacterial homologues of FabI if the creation of pathogen-specific antibiotics is of interest.^14^ The generated X-ray co-crystal structures of fabimycin bound to *A. baumannii* and *E. coli* FabI reveal that key hydrogen-bonding interactions are conserved between the two forms of the enzyme, but no significant protein conformational differences in the active site. Given the limitations of the FabI enzymatic assay when evaluating compounds possessing low nanomolar potency, use of multiple biochemical techniques will be critical to the understanding of current and future FabI inhibitors. Overall, a combination of biochemical, structural, biophysical, and computational approaches was used to better understand the differences in whole-cell activity of fabimycin and **(*****R*****)-7**. Biochemical assessment of the inhibitory activities of fabimycin and **(*****R*****)-7** showed modest differences in potency against purified *E. coli* FabI (4.75-fold), whereas the differences in MIC activity between the two enantiomers in Gram-negative clinical isolates is much more significant (64–128 fold). Comparison of the co-crystal structures of fabimycin and **(*****R*****)-7** reveals a more expansive and tightly structured water network in the fabimcyin structure. This result is consistent with ITC data showing fabimycin to have a more favorable binding entropy, DSF results showing a larger change in melting temperature upon compound binding to FabI relative to the less-active **(R)-7**, and with molecular dynamic simulations showing fabimycin to be much less strained (i.e., more stable) in the *E. coli* FabI active site. Taken together, these results suggest that the differences in whole-cell activity between the two enantiomers is primarily due to a more favorable water interaction network and higher degree of protein and ligand stability for fabimycin.

Beyond Gram-negative activity, the ability of fabimycin to retain approximately equivalent activity as Debio-1452 against *S. aureus* is impressive, as Debio-1452 was optimized to treat *S. aureus* infections and has thus set a high bar for potency versus this pathogen, both *in vitro* and *in vivo*.^17,43,44^ The very low frequency of resistance (1.8 × 10^–10^ to 5.2 × 10^–10^, likely due at least in part to critical interactions with the amide backbone of FabI),^14,45^ outstanding performance in the serial passage experiment, and the extremely low mutation prevention concentration (0.125 μg/mL) suggest great promise for fabimycin against *S. aureus*, as the MPC is dwarfed by the concentration of fabimycin achieved in mouse plasma (*C*_max_ = 47 μg/mL, *t*_1/2_ = 1.4 h).

Inhibition of FabI is attractive due to its orthogonality (relative to the mammalian fatty acid biosynthetic analogue) and its essential nature, for certain bacteria, in the maintenance of cellular membranes.^46,47^ The nature of the FabI target is such that its inhibition will not be lethal to all types of bacteria, as many bacteria have redundant enzymes or can compensate by exogenous fatty acid uptake;^29^ indeed, fabimycin has virtually no whole-cell activity versus a sampling of such species. While this includes some pathogenic bacteria, such as *P. aeruginosa*, it also includes many commensal bacteria, suggesting that a suitable FabI inhibitor could be less damaging to the gut microbiome than the typical broad-spectrum antibiotic.^31^ In this vein, the activity of fabimycin against UTIs is very promising, given how antibiotic treatment of UTIs is known to trigger *Clostridioides difficile* infection^48^ and the recently described “gut–bladder axis” in recurrent UTIs.^49,50^

Similar to fusidic acid and some other antibiotics,^51,52^ the advancement of this class of FabI inhibitors has been complicated by the uniquely poor stability of these compounds in mouse plasma. Given the promising activity of fabimycin in mouse infection models and encouraging data that fabimycin is dramatically more stable in rat and human plasma, it is reasonable to believe that fabimycin efficacy may improve as it is used to treat infections in higher organisms. The potency of fabimycin, combined with the very low resistance frequency and apparent lack of pre-existing resistance, bodes well for its translation.

## Supporting Information Available

Supporting figures and tables, materials and methods, experimental procedures, and characterization of chemical products including LCMS traces and NMR (^1^H, ^13^C) spectra (PDF)
