# Correction to “An Iterative Approach Guides Discovery of the FabI Inhibitor Fabimycin, a Late-Stage Antibiotic Candidate with *In Vivo* Efficacy against Drug-Resistant Gram-Negative Infections”

**DOI:** 10.1021/acscentsci.2c00969

**Published:** 2022-09-06

**Authors:** Erica N. Parker, Brett N. Cain, Behnoush Hajian, Rebecca J. Ulrich, Emily J. Geddes, Sulyman Barkho, Hyang Yeon Lee, John D. Williams, Malik Raynor, Diana Caridha, Angela Zaino, Jason M. Rohde, Mark Zak, Mrinal Shekhar, Kristen A. Muñoz, Kara M. Rzasa, Emily R. Temple, Diana Hunt, Xiannu Jin, Chau Vuong, Kristina Pannone, Aya M. Kelly, Michael P. Mulligan, Katie K. Lee, Gee W. Lau, Deborah T. Hung, Paul J. Hergenrother

**Affiliations:** † Department of Chemistry and Carl R. Woese Institute for Genomic Biology, University of Illinois at Urbana−Champaign, Urbana, Illinois 61801, United States; ‡ Broad Institute of MIT and Harvard, Cambridge, Massachusetts 02142, United States; § Walter Reed Army Institute of Research, Silver Spring, Maryland 20910, United States; ∥ Department of Pathobiology, College of Veterinary Medicine, University of Illinois at Urbana−Champaign, Urbana, Illinois 61801, United States; ⊥ Department of Molecular Biology and Center for Computational and Integrative Biology, Massachusetts General Hospital, Boston, Massachusetts 02115, United States

Two authors from Walter Reed Army Institute of Research were inadvertently left off the author list for this paper. The corrected author list and affiliations are as presented in this Correction.

