# Supplementary material for: An Iterative Approach Guides Discovery of the FabI Inhibitor Fabimycin, a Late-Stage Antibiotic Candidate with In Vivo Efficacy against Drug-Resistant Gram-Negative Infections
Source: ACS Cent Sci. 2022 Aug 10;8(8):1145–58. doi: 10.1021/acscentsci.2c00598 (PMC9413440; doi:10.1021/acscentsci.2c00598)
Supplement: Supplementary file 1 — oc2c00598_si_001.pdf [file oc2c00598_si_001.pdf]

An iterative approach guides discovery of the FabI inhibitor fabimycin, a late-stage antibiotic candidate with *in vivo* efficacy against drug-resistant gram-negative infections

Erica N. Parker,[1#] Brett N. Cain,[1#] Behnoush Hajian,[2] Rebecca J. Ulrich,[1] Emily J. Geddes,[1] Sulyman Barkho,[2] Hyang Yeon Lee,[1] John D. Williams,[3] Malik Raynor,[3] Diana Caridha,[3] Angela Zaino,[2] Mrinal Shekhar,[2] Kristen A. Muñoz,[1] Kara M. Rzasa,[2] Emily R. Temple,[2] Diana Hunt,[2,5] Xiannu Jin,[3] Chau Vuong,[3] Kristina Pannone,[3] Aya M. Kelly,[1] Michael P. Mulligan,[1] Katie K. Lee,[2] Gee W. Lau,[4] Deborah T. Hung,[2,5] Paul J. Hergenrother*[1]

[1]Department of Chemistry and Carl R. Woese Institute for Genomic Biology, University of Illinois at Urbana-Champaign, Urbana, IL 61801, University of Illinois at Urbana-Champaign, Urbana, IL 61801, USA

[2]Broad Institute of MIT and Harvard, Cambridge, MA, 02142, USA

[3]Walter Reed Army Institute of Research, Silver Spring, MD, 20910, USA

[4]Department of Pathobiology, College of Veterinary Medicine, University of Illinois at Urbana-Champaign, Urbana, IL, 61801, USA

[5]Department of Molecular Biology and Center for Computational and Integrative Biology, Massachusetts General Hospital, Boston, MA, 02115, USA

[#]These authors contributed equally

*Correspondence: hergenro@illinois.edu (P.J.H.)

TABLE OF CONTENTS

I. Extended Data Figures and Tables ……………………………………4
Figure S1……………………………………4
Figure S2……………………………………5
Figure S3……………………………………6
Figure S4……………………………………7
Figure S5……………………………………8
Figure S6……………………………………9
Figure S7……………………………………10
Figure S8……………………………………11
Figure S9……………………………………12
Figure S10……………………………………13
Figure S11……………………………………14
Figure S12……………………………………15
Table S1……………………………………16
Table S2……………………………………17
Table S3……………………………………18

II. Materials and Methods for Biological Experiments……………………………………19

III. Synthetic Schemes……………………………………26
Scheme S1. Synthesis of **fabimycin**……………………………………26
Scheme S2. Synthesis of compound **2**……………………………………26
Scheme S3. Synthesis of compound **3**……………………………………26
Scheme S4. Synthesis of compound **4**……………………………………27
Scheme S5. Synthesis of compound **5**……………………………………27
Scheme S6. Synthesis of compound **6**……………………………………27
Scheme S7. Synthesis of compound **±7**……………………………………28
Scheme S8. Synthesis of compound **±8**……………………………………28
Scheme S9. Synthesis of compound **±9**……………………………………28
Scheme S10. Synthesis of compound **10**……………………………………29
Scheme S11. Synthesis of compounds **21** and **22**……………………………………29

IV. Synthetic Procedures……………………………………30
General Synthetic Remarks……………………………………30
General Synthetic Procedures……………………………………30
General procedure 1……………………………………30
General procedure 2……………………………………31

Synthetic procedures……………………………………32
Synthetic procedure 1……………………………………32
Synthetic procedure 2……………………………………32
Synthetic procedure 3……………………………………32

Synthetic procedure 4……..33
Synthetic procedure 5……..33
Synthetic procedure 6……..33
Synthetic procedure 7……..34
Synthetic procedure 8……..34
Synthetic procedure 9……..34
Synthetic procedure 10……35
Synthetic procedure 11……35
Synthetic procedure 12……35
Synthetic procedure 13……36
Synthetic procedure 14……36
Synthetic procedure 15……37
Synthetic procedure 16……37
Synthetic procedure 17……37
Synthetic procedure 18……38
Synthetic procedure 19……38
Synthetic procedure 20……38
Synthetic procedure 21……39
Synthetic procedure 22……39
Synthetic procedure 23……39
Synthetic procedure 24……40
Synthetic procedure 25……40
Synthetic procedure 26……40
Synthetic procedure 27……41
Synthetic procedure 28……41
Synthetic procedure 29……41
Synthetic procedure 30……41
Synthetic procedure 31……42
Synthetic procedure 32……42
Synthetic procedure 33……42
Synthetic procedure 34……43
Synthetic procedure 35……43
Synthetic procedure 36……43
Synthetic procedure 37……44
Synthetic procedure 38……44

V. Tabulated Characterization Data……..45

VI. ^{1}H, ^{13}C NMR Spectra……62
General Remarks……62

VII. References……119

I. Extended Data Figures and Tables

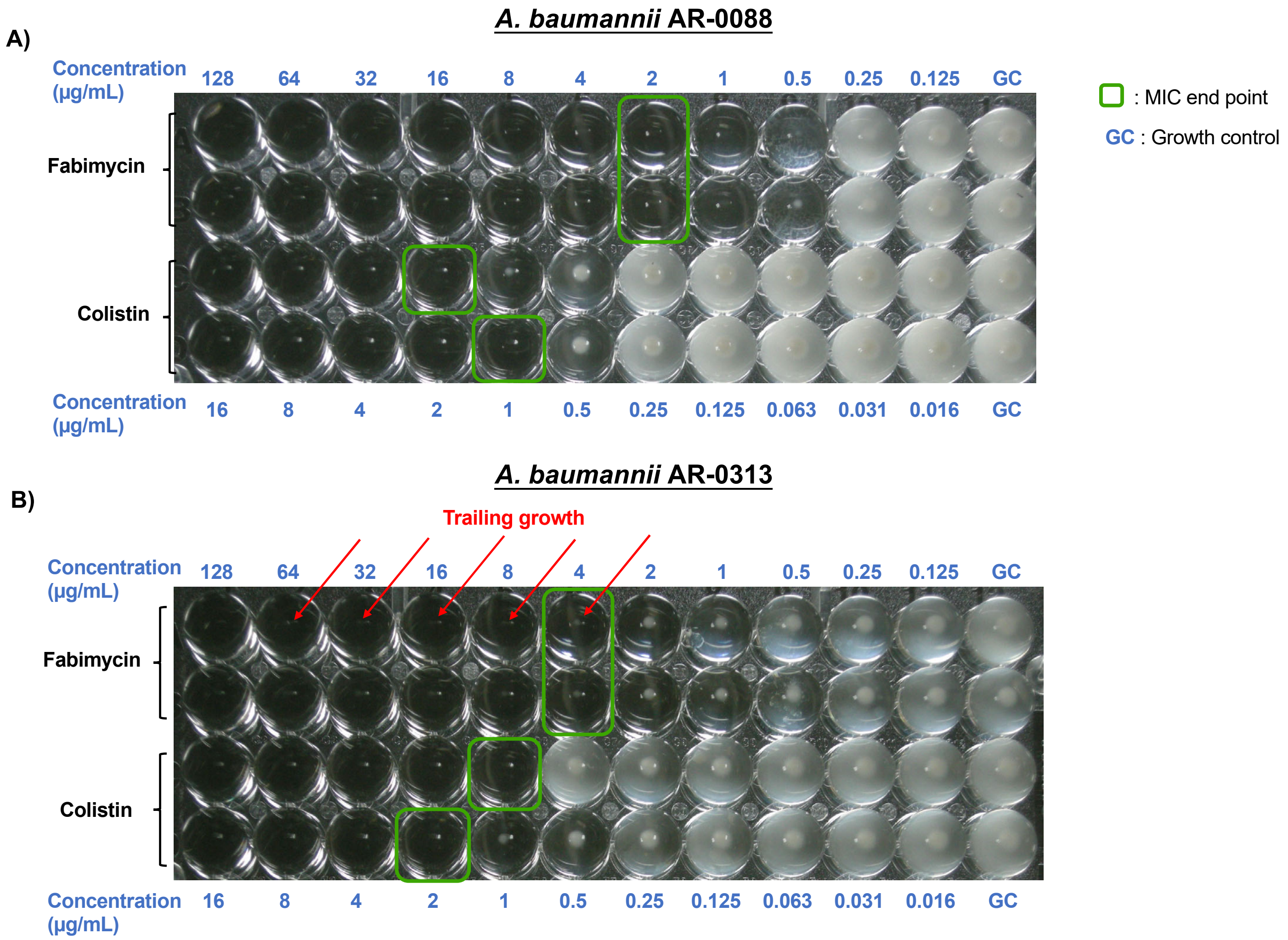

Extended Data Figure S1. Behavior of *A. baumannii* versus fabimycin in MIC experiments. A) Visual results of a typical MIC experiment of fabimycin versus *A. baumannii* (strain AR-0088) conducted in duplicate. **B)** An example of dose-independent trailing growth observed with *A. baumannii* (strain AR-0313) when tested in duplicate. MICs were called at the major reduction in bacterial growth in such cases, as shown. Similar behavior of *A. baumannii* has been observed in broth microdilution assays previously.[1, 2] Although this has not been explored thoroughly in relation to fabimycin, it could be the result of heteroresistance occurring in the tested *A. baumannii* populations.

	Reference strains		
	S. aureus 29213	*E. coli* ΔtolC (JW5503)	*E. coli* MG1655
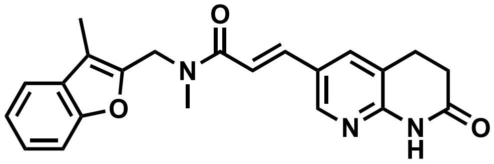 **Debio-1452**	**0.008**	**0.062**	**>32**
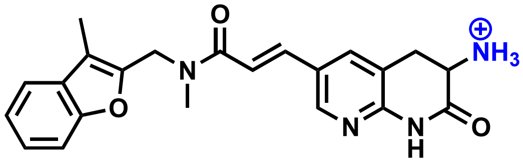 **Debio-1452-NH3**	**0.031**	**0.062**	**4**
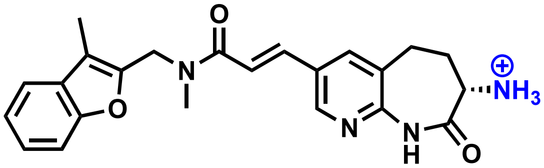 ***(S)-7*, fabimycin**	**0.004**	**0.016**	**2**
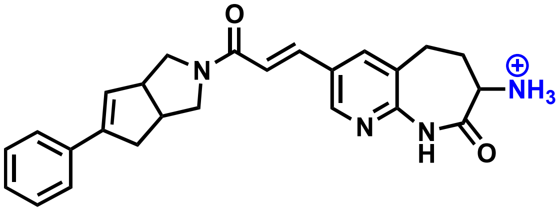 **21**	**0.062**	**0.062**	**16**
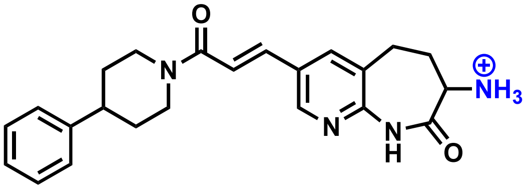 **22**	**4**	**>32**	**>32**

Extended Data Figure S2. Antibacterial effects of substituting the benzofuran on the fabimycin scaffold. MIC values (in µg/mL) were determined using the micro-dilution broth method, as outlined by the CLSI guidelines. All experiments were performed in biological triplicate. Compounds **21** and **22** were tested as stereoisomeric mixtures.

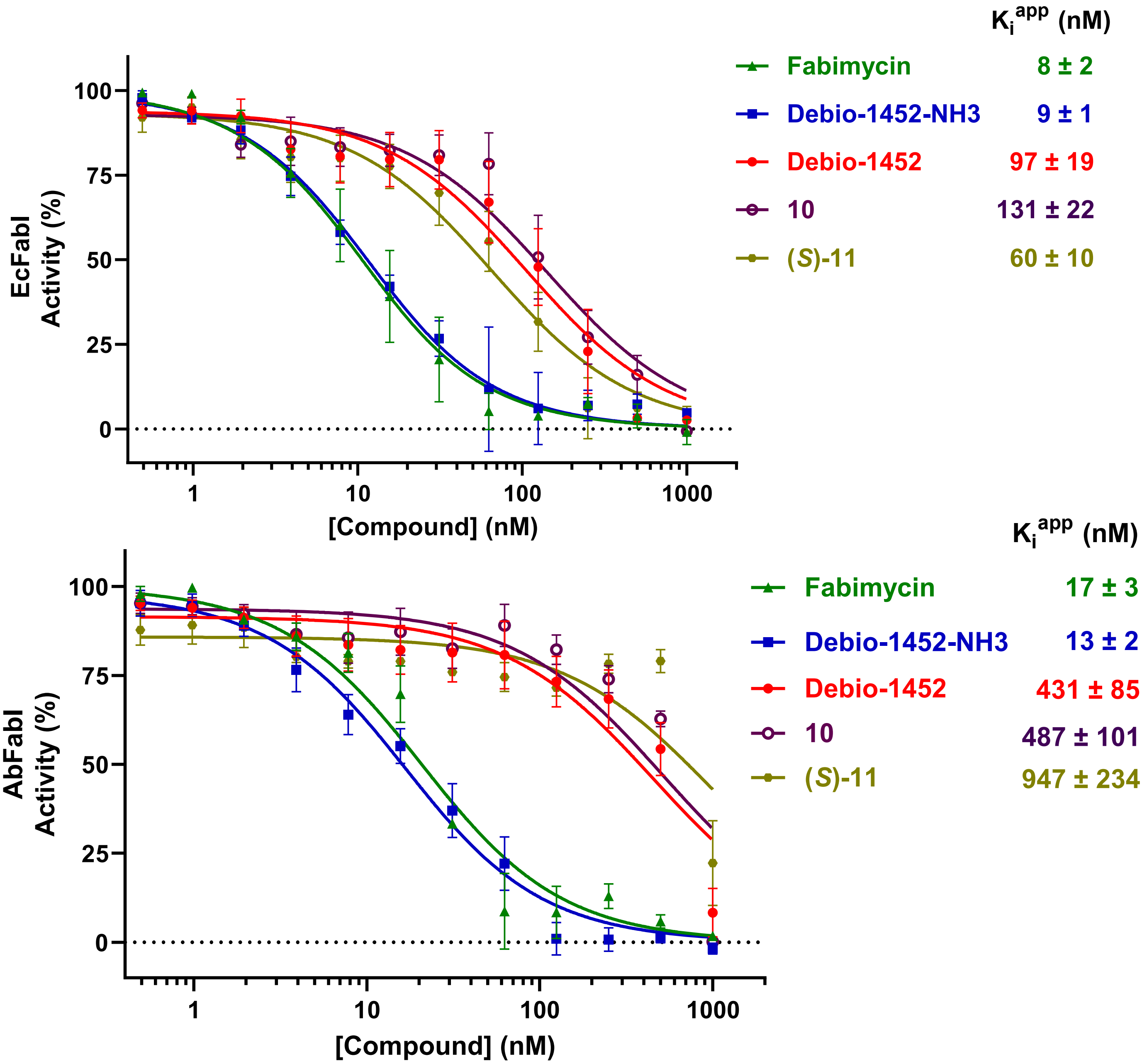

Extended Data Figure S3. *In vitro* FabI activity assay. Biochemical inhibition of FabI from *E. coli* (top) and *A. baumannii* (bottom). Assay protocol optimized from Parker and co-workers,[3] see supporting information for details. The apparent inhibition constant Ki^{app} was calculated by fitting the data to Morrison's Quadratic model. Inhibition data represents the average of technical triplicates. Error bars represent s.e.m.

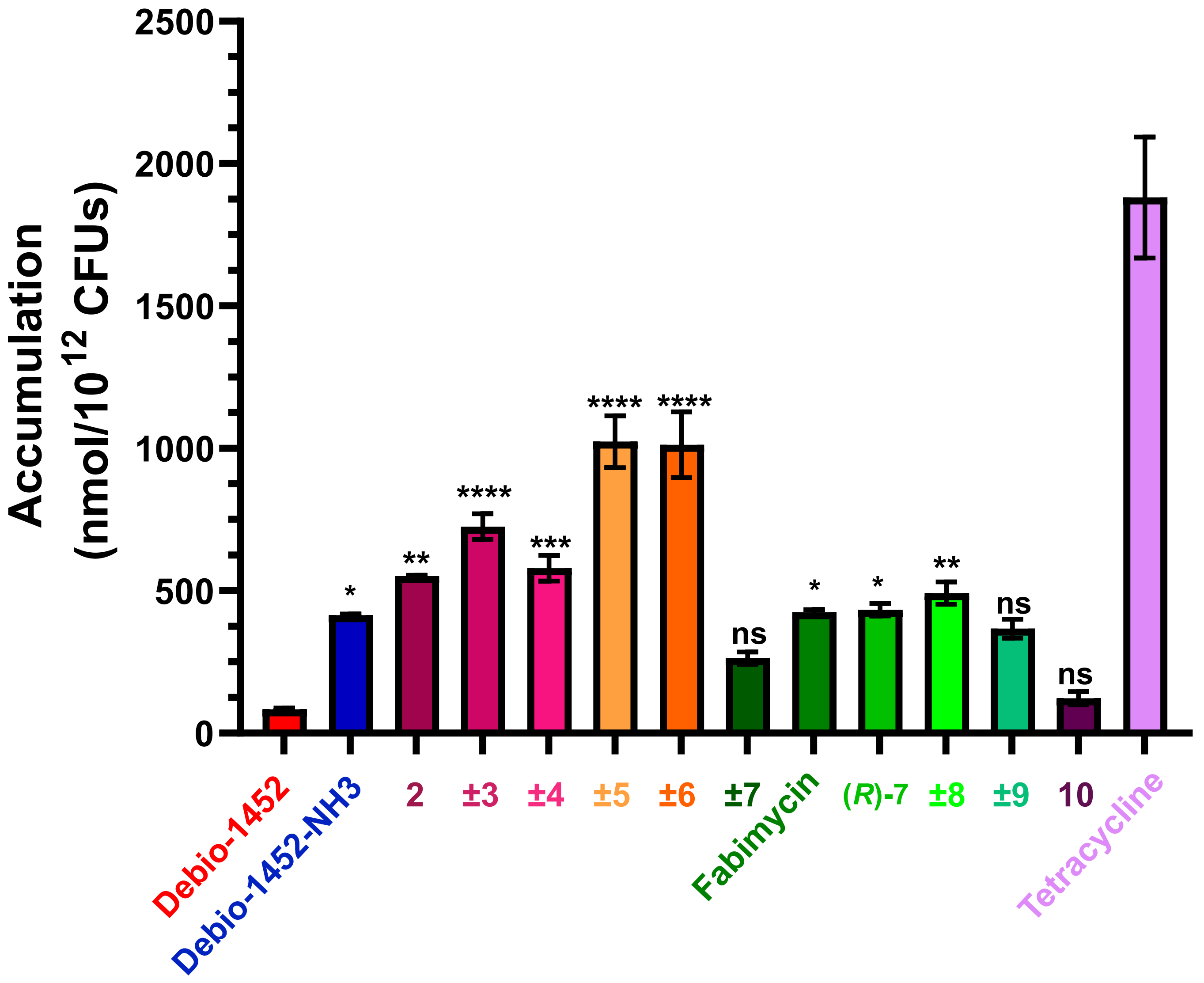

Extended Data Figure S4. Intracellular accumulation of Debio-1452 and related analogues. Evaluation of the ability of compounds to accumulate in *E. coli* MG1655 at 10 minutes, assay conducted as per Geddes and co-workers.[4] Tetracycline was used as a positive control. Measurements compared using ordinary one-way ANOVA with Šídák's multiple comparisons. Statistical significance relative to Debio-1452 is indicated by asterisks (ns, not significant when $P > 0.13$, *$P \leq 0.035$, **$P \leq 0.0063$, ***$P = 0.0007$, ****$P < 0.0001$. Data shown as the mean of three experiments with error bars representing standard error of the mean (s.e.m).

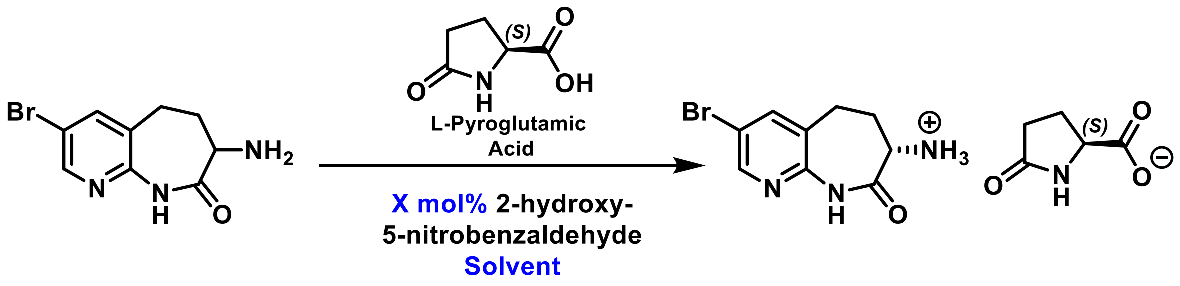

Reaction condition screening					
Solvent	mol% aldehyde	Rxn conc.	Temp	Time	% (*S*)-isomer
EtOH	3	0.1 M	65 °C	3d	**80.0**
MeCN	3	0.1 M	50 °C	2d	**88.4**
THF	3	0.1 M	50 °C	2d	**91.5**
MeOH	3	0.1 M	rt	2d	**70.9**
THF	1	0.04 M	50 °C	2d	**92.6**
THF	6	0.04 M	50 °C	2d	**84.0**
THF	1	0.01 M	50 °C	7d	**89.3**
**MeCN**	1	**0.04 M**	**50 °C**	**2d**	**94.3**
MeCN	6	0.04 M	50 °C	2d	**87.7**

Unless otherwise stated, the percentage of the major isomer was determined by ^{1}H NMR (cb500, D1=30s) with S-Binol as a CSA.

Recrystallization screening				
Recrystallization Conditions (all heated to 50 °C)	Filtration temp	Initial % (*S*)-isomer	Final % (*S*)-isomer	% Yield
Initial filtrate	50 °C	ND	98.3	79
Initial filtrate	RT	ND	97.6	91
Initial filtrate	15 °C	ND	96.3	90
MeCN	RT	98.3	98.5	ND
Acetone	50 °C	98.3	98.8	ND
**MeCN:H_2O (90:10)**	**50 °C**	**98.3**	**99.7**	**66**
Acetone:H_2O (90:10)	RT	97.3	98.8	78
MeCN:H_2O (95:5)	RT	97.3	98.5	92
MeCN:H_2O (95:5) [large scale]	RT	97.9	99.3	96

Extended Data Figure S5. Dynamic kinetic resolution optimization in fabimycin synthesis. The percentage of the major isomer was determined by ^{1}H NMR (500 MHz NMR, D1=30s) with (*S*)-Binol to show chemical shift anisotropy.

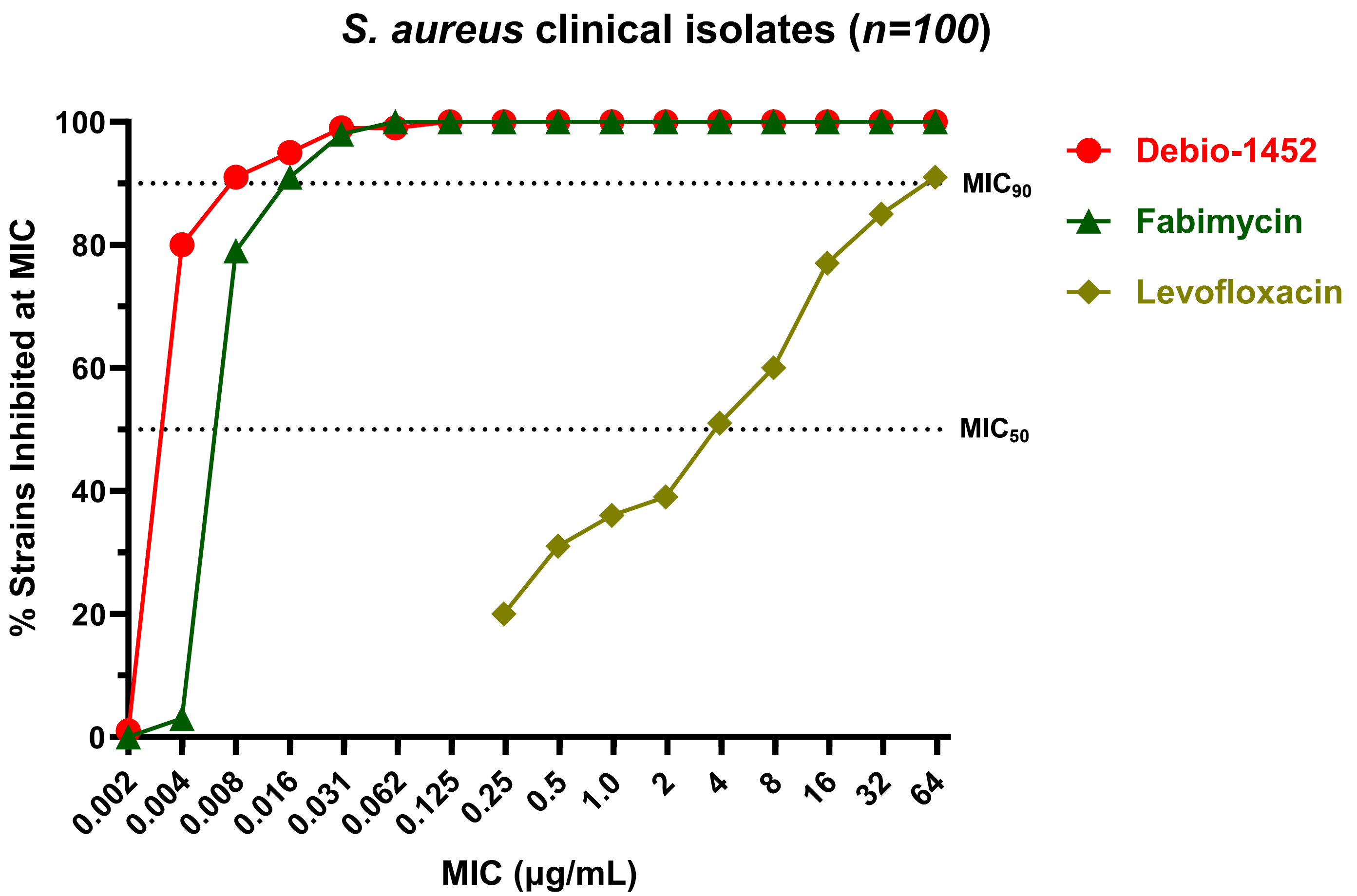

Extended Data Figure S6. Fabimycin activity versus expanded panel of *S. aureus* clinical isolates. Fabimycin's bioactivity relative to Debio-1452 and levofloxacin. MICs conducted in duplicate.

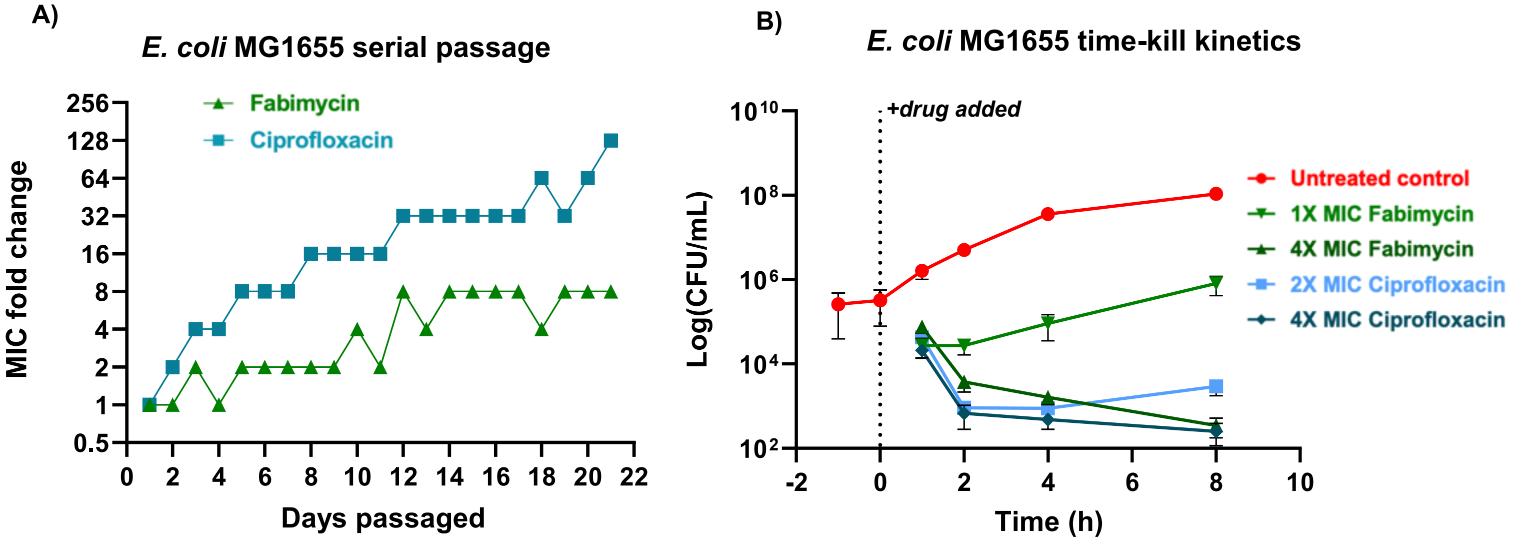

Extended Data Figure S7. A) Result of MIC fold changes due to resistance upon continuous incubation at sub-inhibitory concentrations of fabimycin or ciprofloxacin. **B)** The effect of various concentrations of fabimycin and ciprofloxacin on *E. coli* MG1655 growth.

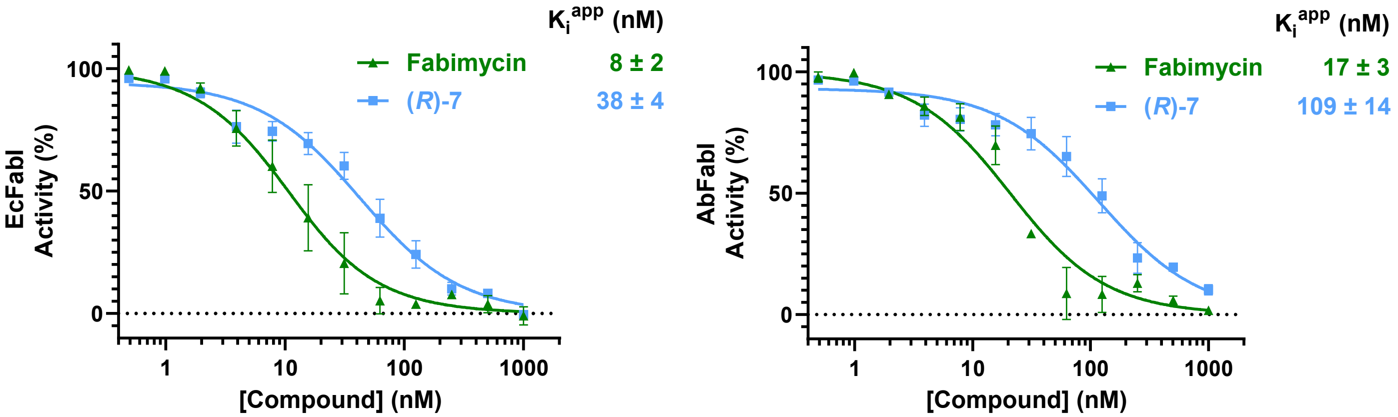

Extended Data Figure S8. Behavior of fabimycin and its enantiomer. Inhibition of purified FabI from *E. coli* and *A. baumannii* by fabimycin and **(*R*)-7**; data for fabimycin is the same as shown in **Extended Data Figure 3.** Inhibition data represents the average of technical triplicates. Error bars represent s.e.m.

A)

B)

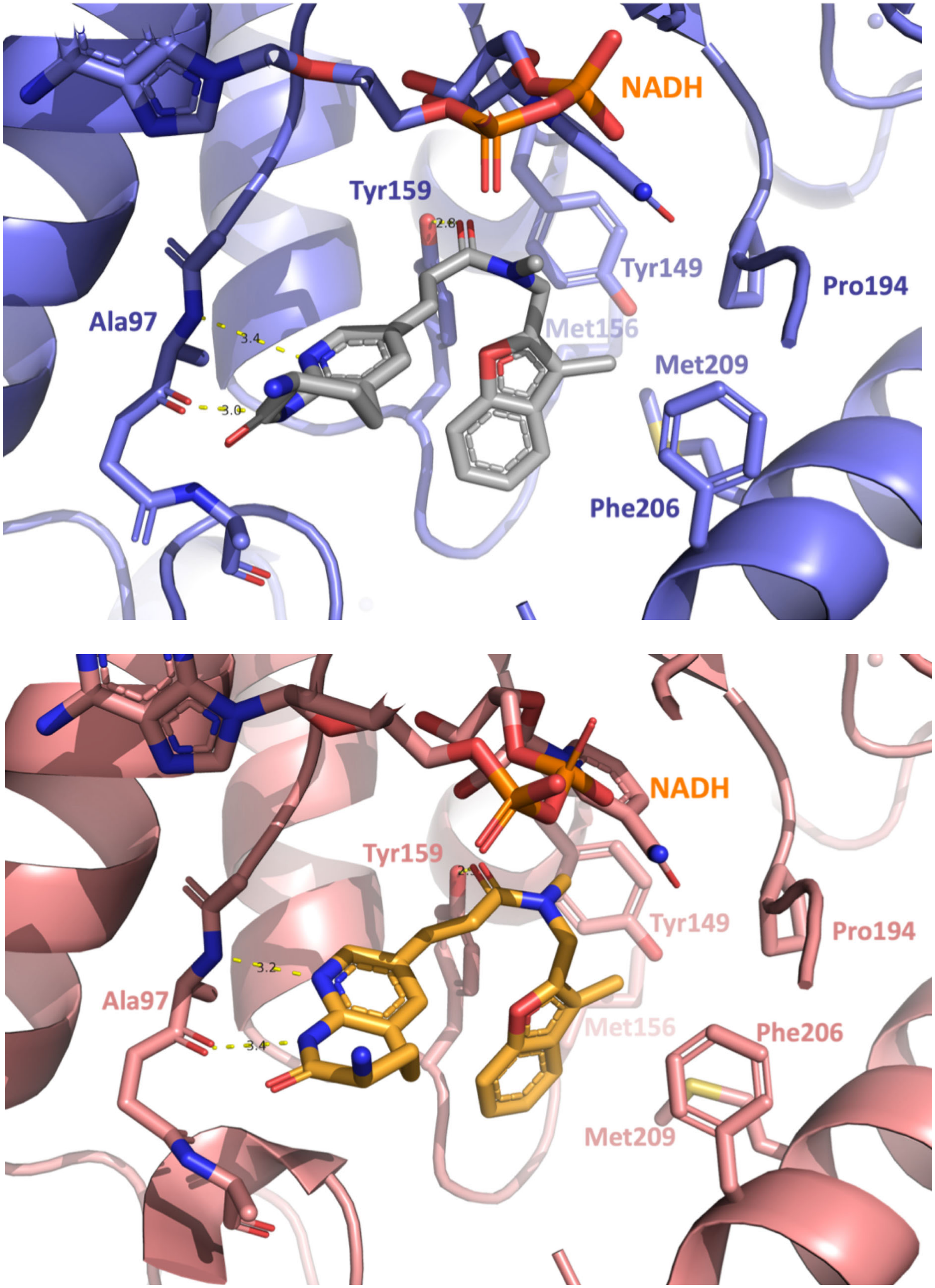

Extended Data Figure S9. Co-crystal structures of fabimycin and its enantiomer in *A. baumannii* FabI. A) Co-crystal structure of fabimycin in *A. baumannii* FabI with NADH cofactor (PDB 7UMY). **B)** Co-crystal structure of **(*R*)-7** in *A. baumannii* FabI with NADH cofactor (PDB 7UMX).

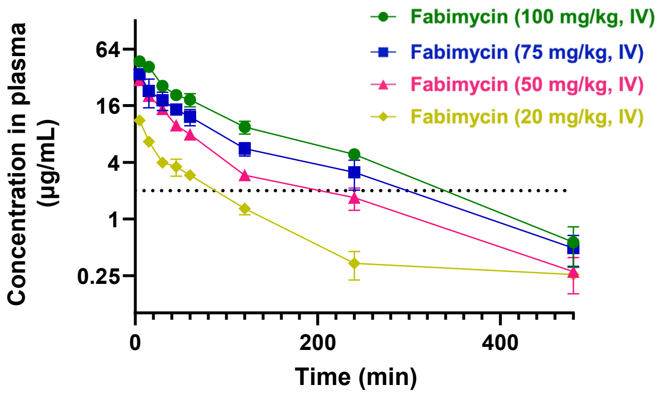

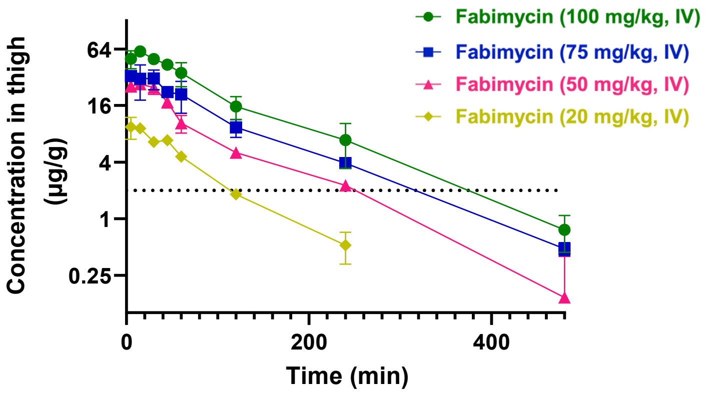

PLASMA		
	Fabimycin (100 mg/kg, IV)	Fabimycin (75 mg/kg, IV)
AUC_{last} (h*µg/mL)	69.8	45.4
$t_{1/2}$ (h)	1.4	1.4
CL (mL/min/kg)	23.5	26.9
C_{max} (µg/mL)	47.3	34.6
TimeHigh (h)	6.67	5.70

THIGH		
	Fabimycin (100 mg/kg, IV)	Fabimycin (75 mg/kg, IV)
AUC_{last} (h*µg/mL)	112	65.2
$t_{1/2}$ (h)	1.24	1.39
CL (mL/min/kg)	14.7	18.9
C_{max} (µg/g)	60.2	33.0
TimeHigh (h)	7.19	6.22

Extended Data Figure S10. Pharmacokinetic analysis of fabimycin. Results of a single-dose of fabimycin in neutropenic, *A. baumannii*-infected mice (24 mice per arm, 3 per time point). The dotted line represents fabimycin's MIC against the infective strain (2 µg/mL). Fabimycin formulated in 17% Cremophor EL, 3% SBE-β-CD in H_2O. Error bars represent the standard deviation.

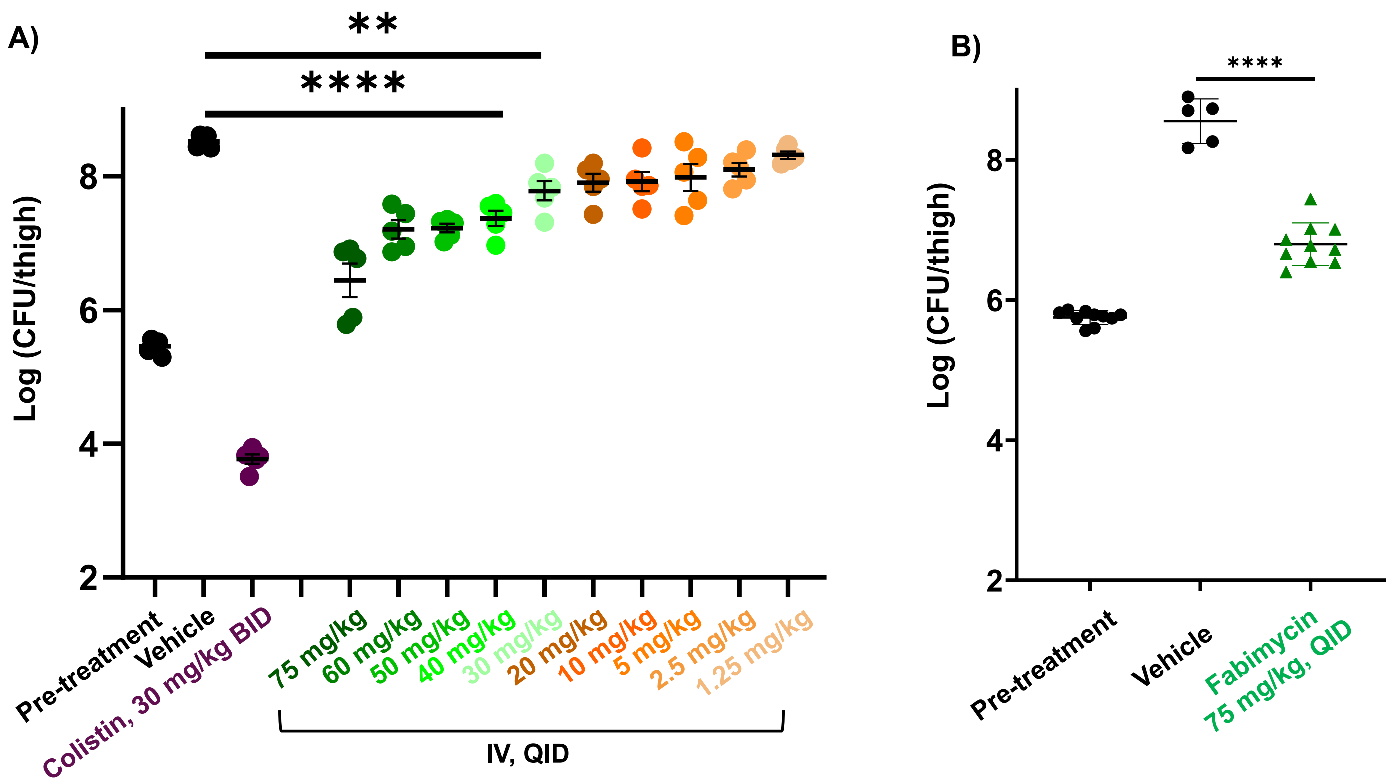

Extended Data Figure S11. Dose ranging and efficacy in *A. baumannii*-infected neutropenic mice. A) Dose ranging of fabimycin (intravenously, four-times-a-day) in neutropenic female BALB/c mice (5 per arm) infected with *A. baumannii* AR-0088 ($8.10*10^4$ CFU per mouse intramuscular, left thigh) with bacterial burden assessed at 26h post-infection. **B)** Neutropenic mouse thigh infection model initiated in female BALB/c mice with *A. baumannii* AR-0088 ($1.36*10^5$ CFU per mouse intramuscular, left thigh) were treated with vehicle (5 mice) or fabimycin (10 mice, 75 mg/kg intravenously, four times a day) and bacterial burden evaluated at 26h post-infection**.** Fabimycin formulated in 17% Cremophor EL, 3% SBE-β-CD in H_2O. In **A** and **B** statistical significance was determined by one-way ANOVA with Tukey's multiple comparisons. **P = 0.008, ****P<0.0001. Data shown as the mean with standard error.

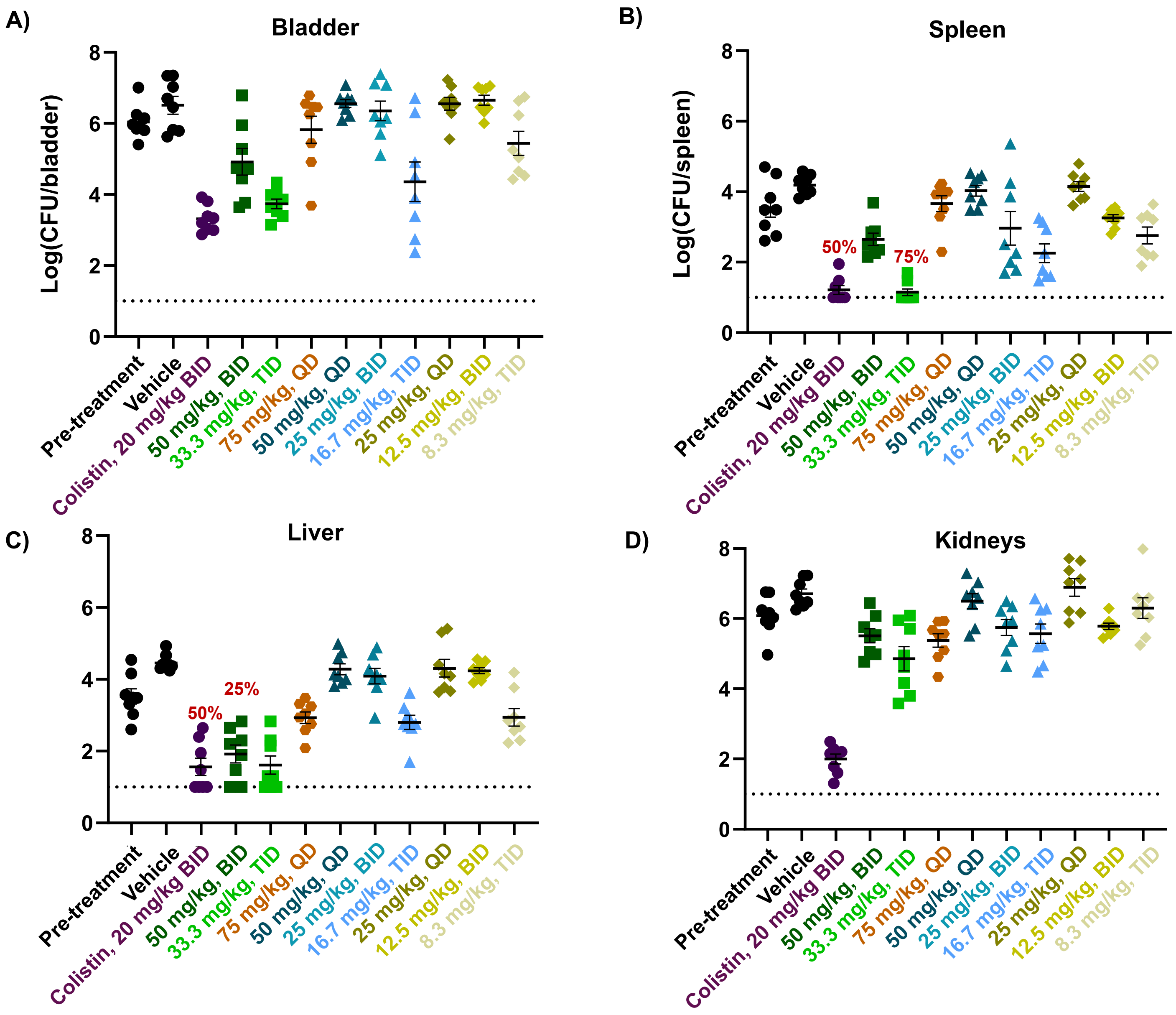

Extended Data Figure S12. Impact of various dosing regimens with fabimycin at 168h post-UTI infection with *E. coli* AR-0055. Fabimycin formulated with 17% Cremophor EL, 3% SBE-β-CD in H_2O and administered intravenously (IV) (8 mice per arm). The dotted line represents the limit of detection for this experiment and the percentage in red shown indicates the percentage of mice with bacterial counts below this threshold. Error bars represent the s.e.m.

Extended Data Table S1. *In vitro* cytotoxicity and ADME assays. Evaluation of Debio-1452 and its amine-containing counterparts against mammalian cells in culture, activity versus the hERG protein, behavior in mammalian plasma, and human red blood cells. The IC_{50} data was collected after incubation with experimental compounds for 72 h with cell viability was determined using the Alamar blue method; percentage death determined by normalizing to DMSO- and raptinal-treated cells. For mammalian cell studies n = ≥3 for fabimcyin and n = ≥2 for Debio-1452 and Debio-1452-NH3. For RBC hemolysis assay cells were treated for 2 hours at 37 ºC, positive control (Triton-X in RBC buffer) and negative control (DMSO) were used. Data is represented as mean ± s.e.m.; n = 3 independent experiments.

		Debio-1452	Debio-1452-NH3	Fabimycin
**Mammalian cells**				
H. sapiens HFF-1	IC_{50} (μM)	165 ± 42	63 ± 33	112 ± 11
	Inhibition at 30 μM (%)	18 ± 6	11 ± 2	9.5 ± 3.4
H. sapiens A549	IC_{50} (μM)	166 ± 12	68 ± 4	114 ± 3
	Inhibition at 30 μM (%)	13 ± 6	29 ± 4	-1.2 ± 3.5
H. sapiens HepG2	IC_{50} (μM)	>250	51 ± 1	75 ± 8
	Inhibition at 30 μM (%)	14 ± 1	17 ± 2	13 ± 3
**Additional ADME studies**				
hERG IC_{50} (μM)		5.5	5.7	21.8
Plasma protein binding (%)	Mouse	99.4 ± 0.1	94.5 ± 0.5	96.4 ± 0.4
	Human	98.1 ± 0.2	89.3 ± 1.3	94.0 ± 0.7
**RBC hemolysis**				
**Fabimycin concentration**	Hemolysis (%)			
50 μM	1.8 ± 0.44			
100 μM	1.9 ± 0.28			
200 μM	2.4 ± 0.40			

Extended Data Table S2. Summary of maximal tolerated dosing (MTD). Determined MTD values of amine-containing FabI inhibitors in various formulations in mice. Compounds formulated with 20% SBE-β-CD in H_2O were administered intraperitoneally (IP). Fabimycin formulated with 17% Cremophor EL, 3% SBE-β-CD in H_2O was administered intravenously (IV).

***In-vivo* (C57BL/6 mice)**	**Debio-1452-NH3**	**Fabimycin**	
Formulation	20% sulfobutyl ether(7) β-cyclodextrin (SBE-β-CD) in H_2O	20% sulfobutyl ether(7) β-cyclodextrin (SBE-β-CD) in H_2O	17% Cremophor EL, 3% SBE-β-CD in H_2O
Single-dose MTD	50 mg/kg	>200 mg/kg	100 mg/kg
Multi-day QD dosing, MTD	50 mg/kg, 5d	>100 mg/kg, 5d	75 mg/kg, 3d
One-day TID dosing MTD	ND	>200 mg/kg	75 mg/kg

Extended Data Table S3. Crystallographic statistics of inhibitor co-crystals in gram-negative FabI.

	ABFABI-S	ABFABI-R	ECFABI-S	ECFABI-R
Resolution range	28.89 - 2.74 (2.838 - 2.74)	44.95 - 2.393 (2.478 - 2.393)	58.06 - 1.54 (1.595 - 1.54)	47.2 - 1.7 (1.761 - 1.7)
Space group	C 1 2 1	C 1 2 1	P 61 2 2	P 61 2 2
Unit cell	256.215 79.137 89.425 90 110.297 90	255.12 79.25 89.9 90 110.296 90	79.5401 79.5401 323.68 90 90 120	79.63 79.63 323.68 90 90 120
Total reflections	86415 (7705)	126417 (12793)	166817 (13113)	136179 (13263)
Unique reflections	43821 (4057)	65755 (6497)	85300 (6889)	68090 (6632)
Multiplicity	2.0 (1.9)	1.9 (2.0)	2.0 (1.9)	2.0 (2.0)
Completeness (%)	98.50 (90.77)	98.68 (98.56)	93.92 (77.26)	99.98 (99.98)
Mean I/sigma(I)	8.40 (1.06)	11.39 (3.06)	16.06 (5.31)	19.55 (3.14)
R-merge	0.04869 (0.4639)	0.03444 (0.1868)	0.02527 (0.1216)	0.0183 (0.1933)
R-meas	0.06886 (0.6561)	0.0487 (0.2642)	0.03574 (0.172)	0.02588 (0.2734)
R-pim	0.04869 (0.4639)	0.03444 (0.1868)	0.02527 (0.1216)	0.0183 (0.1933)
CC1/2	0.999 (0.624)	0.999 (0.839)	0.999 (0.913)	1 (0.896)
CC*	1 (0.876)	1 (0.955)	1 (0.977)	1 (0.972)
Reflections used in refinement	43733 (4003)	65755 (6497)	85294 (6887)	68085 (6631)
Reflections used for R-free	1990 (185)	2004 (199)	1993 (160)	1984 (193)
R-work	0.2557 (0.3262)	0.2077 (0.2960)	0.2136 (0.2785)	0.1652 (0.2669)
R-free	0.2961 (0.3387)	0.2508 (0.3410)	0.2447 (0.2912)	0.1896 (0.2995)
CC(work)	0.959 (0.435)	0.848 (0.399)	0.954 (0.752)	0.973 (0.896)
CC(free)	0.816 (0.407)	0.837 (0.251)	0.933 (0.638)	0.961 (0.919)
Number of non-hydrogen atoms	11921	12224	4344	4491
macromolecules	11455	11611	3843	3825
ligands	588	588	148	148
solvent	22	169	353	518
Protein residues	1551	1558	519	517
RMS(bonds)	0.007	0.003	0.009	0.018
RMS(angles)	1.33	0.62	1.13	1.53
Ramachandran favored (%)	94.65	96.37	97.28	97.47
Ramachandran allowed (%)	4.5	3.56	2.72	2.34
Ramachandran outliers (%)	0.85	0.06	0	0.19
Rotamer outliers (%)	3	1.72	0.26	0
Clashscore	39.68	28.61	3.57	2.94
Average B-factor	94.48	45.31	21.23	27.92
macromolecules	94.28	45.23	20.9	26.9
ligands	101.27	48.98	14.62	22.92
solvent	61.83	41.44	27.58	36.94
Number of TLS groups	1	1	1	1

II. Materials and Methods for Biological Experiments

Bacterial strains

S. aureus ATCC 29213 and *E. coli* MG1655 were obtained from the American Type Culture Collection (ATCC). *E. coli* BW25113 and *E. coli* JW5503 were obtained from the Keio Collection. AR-bank strains were obtained from the Centers for Disease Control and Prevention and FDA Antibiotic Resistance Isolate Bank.

Antimicrobial susceptibility tests

Susceptibility testing was performed in biological triplicate, unless noted otherwise, using the micro-dilution broth method as outlined by the Clinical and Laboratory Standards Institute. Bacteria were cultured with cation-adjusted Mueller Hinton broth (Sigma Aldrich, catalogue number: 90922).

Accumulation assay

The accumulation assay[4] was performed in triplicate with each batch containing tetracycline as a positive control. *E. coli* MG1655 was used in these experiments. For each replicate, 2.5 mL of an overnight culture of *E. coli* was diluted into 250 mL of fresh Luria Bertani (LB) broth (Lennox) and grown at 37 ºC with shaking to an optical density (OD_{600}) of 0.55. The bacteria were pelleted at 3220 r.c.f. for 10 min at 4 ºC and the supernatant was discarded. The pellets were resuspended in 40 mL of phosphate buffered saline (PBS) and pelleted as before, and the supernatant discarded. The pellets were resuspended in 8.8 mL of fresh PBS and aliquoted into Eppendorf tubes (875 µL each). The number of colony-forming units (CFUs) was determined by a calibration curve. The samples were equilibrated at 37 ºC with shaking for 10 min. These time points were short enough to minimize metabolic and growth changes (no changes in OD_{600} or CFUs observed). After incubation, 800 µL of the cultures were carefully layered on 700 µL of silicone oil (9:1 AR20/Sigma High Temperature, cooled to -78 ºC). Bacteria were pelleted through the oil by centrifuging at 13000 r.c.f. for 2 min at room temperature (with the supernatant remaining above the oil); the supernatant and oil were then removed by pipetting. To lyse the samples, each pellet was dissolved in 200 µL of water, and then they were subjected to three freeze-thaw cycles of three minutes in liquid nitrogen followed by three minutes in a water bath at 65 ºC. The lysates were pelleted at 13000 r.c.f. for 2 min at room temperature and the supernatant was collected (180 µL). The debris was re-suspended in 100 µL of methanol and pelleted as before. The supernatants were removed and combined with the previous supernatants collected. Finally, remaining debris was removed by centrifuging at 20000 r.c.f. for 10 min at room temperature. Supernatants were analyzed by LC-MS/MS.

Samples were analyzed with the 550 QTRAP LC-MS/MS system (AB Sciex) with a 1200 series HPLC system (Agilent Technologies) including a degasser, an autosampler, and a binary pump. The liquid chromatography separation was performed on an Agilent SB-Aq column (4.6 x 50 mm, 5 µM) (Agilent Technologies) with mobile phase A (0.1% formic acid in water) and mobile phase B (0.1% formic acid in acetonitrile). The flow rate was 0.3 mL/min. The linear gradient was as follows: 0-3 min, 100% mobile phase A; 10-15 min 2% mobile phase A; 15.5-21 min, 100% mobile phase A. The autosampler was set at 5 ºC. The injection volume was 15 µL. Mass spectra were acquired with both positive electrospray ionization at the ion spray voltage of 5500 V and negative electrospray ionization at the ion spray voltage of -4,500 V. The source temperature was 450 ºC. The curtain gas, ion source gas 1, and ion source gas 2 were 33, 50, and 65 psi, respectively. Multiple reaction monitoring was used for quantification. Debio-1452: *m/z* 376.1 → 244.1; Debio-1452-NH3: *m/z* 391.2 → 171.0. Fabimycin: *m/z* 405.3 → 145.3. Power analysis was not used to determine the number of replicates. Error bars represent the standard error of the mean of three biological replicates. All compounds evaluated in biological assays were ≥95% pure.

Time-kill kinetics

An overnight culture of *E. coli* MG1655 was diluted in 5 mL of MH broth to ~5.0 * 10^5 CFU/mL and incubated at 37 ºC while shaking for 1 hour. The bacteria were then challenged with various concentrations of drug MIC and observed over the course of 8 hours. At each time point 100 µL of culture was collected and serially diluted in sterile PBS before plating on drug-free agar plates. Colonies were counted after incubation at 37 ºC overnight and CFU/mL calculated.

Serial passaging

Serial passaging procedure was done as reported in literature.[5] Briefly, *E. coli* MG1655 cells were grown in 0.6 mL of MH media containing fabimycin and ciprofloxacin (control) at different concentrations. Cells were added to the respective compounds at 0.25X, 0.5X, 1X, 2X, and 4X to start. At 22-24 hours the cultures were checked for growth. Cultures which displayed robust growth (often two times below the MIC) were diluted 1:100 into fresh media containing compound at varying concentrations based off of the MIC. This serial passaging was repeated for 21 days. Any cultures that grew at higher than the tested MIC levels were streaked on drug-free agar plates and MIC determined by broth microdilution.

Cell culture

HFF-1 cells (male, newborn) were obtained from the ATCC. HFF-1 cells were grown in Dulbecco's Modified Eagle's minimum essential medium with 15% fetal bovine serum (Gemini Benchmark; catalogue number: 100-106), 100 µg/mL penicillin and 100 µg/mL streptomycin. A549 cells (male, adult) were obtained from the ATCC. A549 cells were grown in Roswell Park Memorial Institute (RPMI) media with 100 µg/mL penicillin and 10% fetal bovine serum (FBS). HepG2 cells were authenticated by the University of Arizona Genetics Core using an autosomal STR profile. HepG2 cells were grown in Dulbecco's Modified Eagle's minimum essential medium with 10% fetal bovine serum. All cells were cultured at 37 ºC under a 5% CO_2 environment. Cell lines were authenticated by a commercial vendor and inspected visually in house. Cell lines were not tested for *Mycoplasma* contamination. The media was prepared by the University of Illinois School of Chemical Sciences Cell Media Facility.

Cell viability

H. sapiens HFF-1, A549, and HepG2 cells were seeded (3,000-6,000 cells/well) in a 96-well plate (Greiner Bio-One; catalogue number: 655180) and allowed to attach overnight. For half-maximum inhibitory concentration (IC_{50}) determination, compounds **Debio-1452**, **Debio-1452-NH3**, and **fabimycin** were dissolved in dimethyl sulfoxide (1% DMSO final; 100 µL/well)).[6] Raptinal (100 µM) was used as a dead control.[7] On each plate, at least three technical replicates per compound were performed. After 72 h post-treatment, cell viability was assessed using the Alamar Blue method.[8] Stock Alamar Blue solution (10 µL 440 µM resazurin (Sigma-Aldrich; catalogue number: R7017) in sterile 1X PBS) was added to each well, and the plate was incubated for 3-4 h. Conversion of Alamar Blue was measured with a plate reader (SpectraMax M3; Molecular Devices) by fluorescence (excitation wavelength: 555 nm; emission wavelength: 585 nm; cutoff 570 nm; autogain). Percentage death was determined by normalizing to DMSO-treated cell and Raptinal-treated cells. For IC_{50} determination, the data were plotted as compound concentration versus the percentage of dead cells and fitted to a logistic-dose-response curve using OriginPro 2015 (OriginLab). Unless noted otherwise, the data were generated in triplicate and IC_{50} values were reported as the average of three separate experiments along with s.e.m. values.

Transformation, expression, and purification of AbFabI and EcFabI

Recombinant plasmids harboring genes encoding AbFabI and EcFabI in pET21GG2-His(6) vector were constructed by GenScript, separately. BL21(DE3) competent E. coli cells (NewEngland BioLabs) were transformed with the recombinant plasmids separately using the manufacturer protocol. Transformed cells were grown in LB medium

supplemented with 100 µg/mL ampicillin at 37°C until OD600 reached 0.6-0.7. The cells were induced with 1mM IPTG and incubated for 20 hours at 18°C and spun down at 8000 rpm for 15 minutes. Cell pellet was resuspended in the lysis buffer (50 mM Tris-HCl pH 8.0, 500 mM NaCl, 20 mM imidazole, 10% glycerol, 0.5 mM TCEP, protease inhibitor and Lysonase) and passed through microfluidizer followed by centrifugation and filtration to remove cell debris. The clarified lysate was supplemented with 20 mM imidazole and passed over a HisTrap column (Cytiva) pre-equilibrated with the wash buffer (50 mM Tris-HCl pH 8.0, 500 mM NaCl, 20 mM imidazole, 10% glycerol, 0.5 mM TCEP). The column was washed with the wash buffer and eluted over a 20-500 mM imidazole gradient. The appropriate fractions were pooled, and the histidine tag was removed by TEV cleavage. The protein mixture was passed over a HisTrap column to separate the cleaved protein from the uncleaved ones. The cleaved protein was pooled and passed over an S75 size exclusion column (GE LifeSciences) pre-equilibrated with 50 mM Tris-HCl pH 8.0, 500 mM NaCl, 0.5 mM TCEP and 10% glycerol. Fractions containing pure protein were pooled, concentrated at 10 mg/mL and stored at -80°C.

Co-crystallization of AbFabI and EcFabI with NADH and inhibitors

All the crystallization trials were performed by hanging drop vapor diffusion method. Pure protein was exchanged into the crystallization buffer containing 50 mM Tris-HCl pH 8.0, 300 mM NaCl, and 0.5 mM TCEP. Protein concentration was adjusted to 10 mg/mL and incubated with 1mM NADH and 2mM inhibitor overnight at 4°C. Drops were setup by mixing the protein mixture with crystallization solution at 2 µL : 2 µL and 2 µL : 3 µL drop ratios and let to equilibrate against 500 µL of well solution at 20°C. AbFabI crystals were grown within 1-2 weeks from solution comprising 100 mM HEPES pH 6.5-7.5, 20% PEG 4K and 15%-20% 2-propanolol. EcFabI crystals were grown within 1-2 weeks from solution comprising 100 mM Tris pH 7.5-8.5 and 0.5-1.5 M sodium citrate tribasic.

Crystallography data collection and structure determination

X-ray data were collected at Brookhaven National Lab (NSLSII AMX). Data were indexed and scaled using iMosflm. Structure was solved by molecular replacement using Phaser and previously reported structures (AbFabI PDB ID 6AHE and EcFabI PDB ID 4JQC). The programs Coot and Phenix were used for structure refinement. Data collection and refinement statistics are reported in **Extended Data Table S3**.

***In vitro* FabI enzymatic inhibition assay**

Recombinant *E. coli* and *A. baummani* FabI enzymes were used in a continuous NADH absorbance assay (20 µL in 384w format). Compounds and the Crotonyl-CoA substrate (Sigma–Aldrich 28007) were dispensed into a plate (GeinerBio-One 781101). The reaction was initiated by the addition of a reaction master mix solution containing enzyme and NADH (Sigma–Aldrich N8129) suspended in reaction buffer (50 mM HEPES pH 7.4, 150 mM NaCl, 1 mM EDTA, 0.01 mg/mL BSA, 0.01% Tween). Final concentrations were 5 nM enzyme, 150 µM Crotonyl-CoA, 380 µM NADH, 0.05% DMSO in 1x reaction buffer. After shaking for 5 seconds the reaction was monitored at NADH absorbance (340nm, 25°C) using a Molecular Devices SpectraMax M5 microplate reader. The linear portion of the progress curve was fit using plate-reader software. Percentage activity was calculated relative to DMSO only and no-substrate controls in each plate and the data were fit to Morrison's quadratic using Graphpad Prism 9.3 to obtain apparent inhibition constant (Ki app) values.

Differential scanning fluorimetry (DSF)

Recombinant enzymes (15 µM) were incubated with NADH and compounds (500 mM and 500 µM, respectively) in assay buffer (50 mM HEPES pH 7.4, 150 mM NaCl, 1 mM EDTA, 5% DMSO). SYPRO™ Orange (ThermoFisher S6650) was added to each reaction for a final concentration of 2x. The dye signal was monitored in a Roche Lightcycler 480 Real-Time PCR System as a function of a temperature ramp (25-95°C; 0.1°/s) and the Tm was calculated using the instrument's software.

Selection of resistant mutants

Resistant mutants were generated using the large inoculum method. Briefly, *S. aureus* 29213, *E. coli* MG1655, and *A. baumannii* 19606 (~1 x 10^{10} CFUs) was plated on 100-mm plates (*E. coli* and *A. baumannii*) or 150-mm plates (*S. aureus*) of MH agar containing either 128, 64, or 32 µg/mL fabimycin (for gram-negatives) or 0.125, 0.062, and 0.031 µg/mL fabimycin (for *S. aureus*). Plates were incubated at 37 ºC for 48 h and counted for the presence of colony forming units. Glycerol stocks of selected colonies were made, and resistant colonies confirmed by growing on agar plates containing the same concentration of fabimycin that they were generated at.

Sequencing of bacterial *fabI*

The FabI gene was amplified by PCR. Fabimycin-resistant colonies were picked and diluted in 100 µL sterile, nuclease-free H_2O. PCR reactions were then set up by combining 14 µL H_2O, 1 µL 20 µM primer mix, 10 µL template (bacteria solution in H_2O), and 25 µL MiFi Mix (Bioline). The reaction was performed on a C1000 Thermal Cycler (Bio-Rad) under the following conditions for 35 cycles: initial denature: 95 ºC for 3 min; denature 95 ºC for 15 s; anneal: 57 ºC for 15 s; extend 72 ºC for 30 s; final extend: 72 ºC for 3 min. A 10 µL portion of PCR reaction mixture was analyzed by agarose gel electrophoresis to confirm the single 1.4-kilobase pair product. PCR products were purified using a GeneJET PCR Purification Kit (Thermo Fischer Scientific). PCR amplicons were submitted to the Core DNA Sequencing Facility at the University of Illinois at Urbana-Champaign for Sanger sequencing with overlapping internal primers. All primers were obtained from Integrated DNA Technologies.

Primers for sequencing of *fabI* gene in select pathogens:

Organism		Sequence	Reference
E. coli MG1655	Forward	5'-GGG-GCC-AGC-GTT-TCT-TTT-TC-3'	Parker, 2020[3]
	Reverse	5'-AAA-CAT-GGA-GAC-GGT-GCT-GG	
A. baumannii 19606	Forward	5'-GTG-AGA-TCG-GCA-TGA-CAC-AA-3'	Lin, 2017[9]
	Reverse	5'-CTG-AAG-TCC-GCT-ACC-GTT-AT-3'	

Compound stability in mammalian plasma

Candidate drugs were incubated with plasma and parent drug levels were monitored at various time points (generally t=0, 10, 20, 30, and 60 minutes) to determine the drug's half-life in the presence of the chosen system. Each drug is tested in duplicate. Compounds were diluted from 10 mM DMSO stocks to 1 µM in a 100 mM pH 7.4 phosphate buffer and incubated at 37 °C with 0.5 mg/mL of human, rat, or mouse plasma (BioreclamationIVT, Baltimore, MD). Time points were collected at 0 min (after addition of drug), 15 min, 30 min, 60 min, and 120 min following quenching with an equal volume of acetonitrile. The supernatant was collected after centrifugation, and the sample analyzed by LC-MS where the percentage of compound remaining is plotted vs time to determine the drug's half-life.

Compound plasma protein binding

Candidate drugs were incubated with plasma and parent drug levels are monitored after 4 hours to determine the drug's free concentration in the presence of the plasma proteins. The standard assay involves equilibrium dialysis, in which compound is allowed to equilibrate between a protein-containing compartment from a protein-free compartment. Each

drug is tested in duplicate. Compounds were diluted from 10mM DMSO stocks to 5 μM in mouse or human plasma (BioreclamationIVT, Baltimore, MD). The dialysis plate (Rapid Equilibrium Dialysis (RED) device; Thermo Scientific, Waltham, MA) was prepared by adding pH 7.4 phosphate buffer to one chamber of the RED device and dosing solution to the other chamber. The plate was sealed with an adhesive tape and incubated at 37 °C while shaking for 4 hours. Equal volumes of post-dialysis samples were removed from both the plasma and the buffer chambers into separate microcentrifuge tubes, and equal volumes (100μL) of fresh phosphate buffer and plasma were added to the tubes, respectively. Samples were then treated with a quenching solution (precipitation buffer (cold 90:20 acetonitrile:water with 0.1% formic acid) containing an internal standard). Sample mixtures were then centrifuged and the supernatant removed for LC-MS analysis. Percentage binding is determined using the following equation: Binding % = (Concentration of test compound in plasma at equilibrium (Cpe) – Concentration of test compound in buffer at equilibrium (Cb)) / Cpe x 100.

Human red blood cell hemolysis

Whole human blood in citrate phosphate dextrose was obtained from BioIVT and stored at 4 ºC and used before expiration date. 100 μL of whole blood was combined with 500 μL saline (0.9% NaCl) and centrifuged for 5 min at 300xg. The supernatant was carefully removed from the erythrocyte pellet and the liquid was discarded. The pellet was further washed 3X in 500 μL saline. The erythrocyte pellet was resuspended in 800 μL of Red Blood Cell Buffer (10 mM Na_2HPO_4, 150 mM NaCl, 1 mM $MgCl_2$, pH 7.4). To a 0.5 mL Eppendorf tube or a PCR plate was added 1.0 μL of 30X compound in DMSO and 19 μL RBC buffer. For negative controls, 1.0 μL DMSO was combined with 19 μL RBC buffer. For positive controls 1.0 μL 30% Triton X-100 was combined with 19 μL RBC buffer. Tubes were briefly centrifuged. Next, 10 μL of washed erythrocyte suspension was added to each tube and sealed. After incubation at 37 ºC for 2 hours, the samples were centrifuged for 5 min at 300xg and 20 μL of the supernatant was carefully removed and transferred to the wells of a clear flat-bottomed 384-well plate. Absorbance was measured at 540 nm.

Mouse maximum tolerated dose (MTD) of fabimycin

The protocol to assess fabimycin's murine MTD when formulated in 20% sulfobutyl ether(7) β-cyclodextrin (SBE-β-CD) in H_2O was approved by the Institutional Animal Care and Use Committee (IACUC) at the University of Illinois at Urbana-Champaign (protocol number: 19181). In these studies, 10- to 12-week-old female C57BL/6 mice purchased from Charles River were used. Mice were randomly chosen and divided into subsequent groups. No additional randomization was used to allocate the experimental groups; blinding was not performed for subsequent quantitation. The MTD of a single dosage of a given compound was determined before evaluating the MTD of multiple dosages. Compounds were administered by intraperitoneal (IP) injection, and mice were monitored for signs of toxicity for 2 weeks (single dose). The single-dose MTDs were determined first. For multiple doses, the compound was given by daily IP injection for five consecutive days and mice were monitored for signs of toxicity for 1 month. The MTD was the highest dosage with acceptable toxicity (<20% weight loss). MTD values shown for Debio-1452-NH3 are as previously reported.[3] MTD values for fabimycin when formulated in 17% Cremophor EL/3% SBE-β-CD were drawn from infection models conducted with neutropenic *A. baumannii*-infected mice (see below).

Pharmacokinetic assessment

The protocol to assess fabimycin's pharmacokinetic properties in *A. baumanii*-infected mice (strain: AR-0088) was approved by the IACUC at Pharmacology Discovery Services, Taiwan, Ltd. (protocol mumber: PK001-09212018). In this study, neutropenic female BALB/c mice 7-8 weeks in age from BioLASCO Taiwan (AAALAC-certified Charles River Licensee) weighing 18 ± 2 grams were used.[10] All animals were specific pathogen free. Mice were rendered neutropenic via cyclophosphamide (CP) administration by two intraperitoneal (IP) injections following a standard method for mouse thigh infection models.[11] The first CP dose, 150 mg/kg, was administered at four days before infection (Day –4); and the second, 100 mg/kg, at 24 h before infection (Day –1), prior to infection on Day 0.

To inoculate the mice, *A. baumannii* cultures were first generated by seeding brain heart infusion (BHI, 20 mL) with an aliquot (0.2 mL) of a single-used glycerol stock of *A. baumannii* AR-0088 stored at -80 ºC and incubating at 35-37 ºC with shaking (250 rpm) for 6 hours. An aliquot of this culture (1.0 mL) was used to seed fresh BHI media (99 mL) and this shaken (250 rpm) at 35-37 ºC for 16 hours. Bacterial cells were harvested by centrifuging 20 mL of this outgrowth culture (3,500 x g) for 15 minutes and re-suspending in fresh cold phosphate buffered saline (PBS, 10 mL). The optical density at 620 nm was measured and used to guide dilutions. PBS suspensions were stored on ice for no more than 1 hour prior to animal inoculation. The bacterial count in the challenge organism suspension was enumerated by dilution plating on to nutrient gar plates followed by 20-24 hour incubation. The target inoculum is $1.0 * 10^5$ CFU/mouse for the strain used here.

Fabimycin was IV administered once followed by terminal blood sampling at 8 time points with cardiac puncture (5, 15, 30, 45, 60, 120, 240, and 480 minutes). Terminal blood collection from cardiac puncture was conducted under CO_2 euthanasia. Blood, 0.3-0.4 mL, was drawn into tubes coated with K_2EDTA, mixed gently and kept on ice and centrifuged at 12,000 × g for 5 minutes at 4°C within 1 hour of collection. The plasma was harvested and stored at -80 °C until further processing. Thigh tissues were aseptically harvested from the left thigh of the infected animals, weighed, and homogenized in sterile PBS (3 mL, pH 7.4) with a Polytron homogenizer. The thigh homogenate was collected and stored at -80 °C until further processing.

For LC-MS/MS sample processing a 20 µL aliquot of each plasma and thigh sample was transferred to 96 well plate. A 300 µL aliquot of 0.01 ng/µL internal standard (IS), oxybutynin, in MeOH/FA=95/5 was added to each tube except for the double blank and carryover controls. A 300 µL aliquot of MeOH/FA=95/5, without oxybutynin, was added to the double blank and carryover assay wells. The equilibrated mixture was vortexed for 1 min and then centrifuged at 4,000 rpm for 5 min at 20 ˚C to precipitate protein. After centrifugation, 50 µL of the supernatant was transferred to a new well of the 96 well plate and 500 µL H_2O/FA=100/0.2 added for LC-MS/MS analysis. Plasma and thigh samples from treatment groups and the calibration curve samples were processed on the same test occasion using the same procedure. The calibration curve samples were generated by spiking aliquots of drug-free plasma or thigh homogenates with the test article dilution. The quality control (QC) samples, QH (high), QM (medium) and QL (low) were each assayed in duplicate. The PK parameters for the IV doses were obtained from the non-compartmental analysis (NCA) of the plasma and thigh homogenate data using WinNonlin (Phoenix® Build 8.3.1.5014).

LC-MS/MS runs were conducted with a SCIEX Triple Quad™ 5500+ mass spectrometer. Method development was conducted to determine appropriate conditions for LC-MS/MS analysis of fabimycin. A titration of a stock solution was performed to correlate peak areas and the corresponding concentration.

Acute pneumonia bacterial burden model

The protocol was approved by the IACUC at the University of Illinois at Urbana-Champaign (protocol number 17271). Seven- to eight-week-old CD-1 mice were purchased from Charles River Laboratories and acclimatized for 4-7 days. All animals were housed in a pathogen-free environment and received sterile food and water. Mice were randomly chosen and divided into subsequent groups. No additional randomization was used to allocate the experimental groups; blinding was not performed for subsequent quantitation. For the preparation of each inoculum, overnight cultures of clinical isolates were diluted into LB broth and grown to log-phase growth at 37 ºC before establishing infection. For this model, infection with *A. baumannii* (AR-0299) was established by intranasal inoculation with $1.6 * 10^8$ CFU/mouse. Mice were

treated at 4, 23, and 41 hours post-infection with FabI inhibitors (intramuscular, 50 mg/kg). Compounds were formulated in 20% sulfobutyl ether(7) β-cyclodextrin from solid immediately before treatment.

Neutropenic thigh infection model

The protocol was approved by the IACUC at the University of Illinois at Urbana-Champaign (protocol number 17271). Acclimated CD-1 mice (see above) were rendered neutropenic prior to the experiment via cyclophosphamide treatment. For the gram-negative infection model *A. baumannii* (AR-0299) infection was established intramuscular at $1.22 * 10^6$ CFU/mouse. Mice were treated with FabI inhibitors at 2, 6, and 11 hours post-infection with bacterial burden being assessed at 26 hours post-infection. For the gram-positive infection model *S. aureus* USA300LAC was established intramuscular injection at $2.3 * 10^6$ CFU/mouse. Mice were treated with FabI inhibitors at 2 and 7 hours post-infection with burden being assessed at 24 hours post-infection.

Dose-ranging and efficacy of fabimycin

The protocol to assess fabimycin's efficacy in *A. baumannii*-infected mice (strain: AR-0088) was approved by the IACUC at Pharmacology Discovery Services, Taiwan, Ltd. (protocol number: IM003-01282019). In this study, neutropenic female BALB/c mice 7-8 weeks in age from BioLASCO Taiwan (AAALAC-certified Charles River Licensee) weighing 18 ± 2 grams were used. The mice were rendered neutropenic and inoculated in the same method as used for the pharmacokinetic study. Fabimycin was formulated in 17% Cremophor EL/3% SBE-β-CD and IV administered. Colistin was formulated in 0.9% NaCl and subcutaneously (SC) administered. Animals were observed at 5 or 30 minutes after dosing with respect to IV and SC route to detect acute toxicity, which was recorded and reported, if observed.

Animals were checked at 12 hours after infection for humane endpoints. Animals were humanely sacrificed if found in a moribund state. Euthanasia was performed following the Pharmacology Discovery Services IACUC approved SOP "Euthanasia Working Instruction" (document QWCN38), which follows the 2020 AVMA Guidelines on Euthanasia. Animals were euthanized using compressed CO_2 gas in a CO_2 gas chamber. Tissues were not recovered if animals were euthanized prior to the scheduled sacrifice time point. To determine efficacy, animals were sacrificed with CO_2 asphyxiation at the scheduled time points for tissue harvest, at 2 or 26 hours after infection. The thigh tissue was aseptically harvested from each of the sacrificed animals, weighed, and homogenized in 3 mL sterile PBS (pH 7.4) with a Polytron homogenizer. Bacterial burden in the tissue homogenates was determined by performing 10-fold serial dilutions and plating 0.1 mL of each to nutrient agar plates. Colonies were counted after 18 - 24 h incubation. The colony-forming unit value per tissue (CFU/thigh) was calculated.

Urinary tract infection model

The protocol to assess fabimycin's efficacy in a urinary tract infection was approved by the IACUC (protocol number: IM011-12282020). In this study specific pathogen-free C3H/HeJ ($Tlr4^{Lps\text{-}d}$) mice that were 7-8 weeks were used and provided with 5% glucose for 6 days prior to infection and for the duration of the experiment.[12, 13] On day 0, mice were infected with *E. coli* AR-0055 ($1.38 * 10^9$ CFU/mouse, transurethral) while under pentobarbital anesthesia (50 mg/kg, IP). Treatment was initiated 96 hours post-infection with test articles. Fabimycin was formulated in 17% Cremophor EL/3% SBE-β-CD and administered intravenously while the control, colistin, was formulated in 0.9% NaCl and administered subcutaneously. Doses were administered once, twice, or three times daily for 3 consecutive days with animal sacrifice and burden enumeration at 168 hours post-infection. Tissues were collected and pathogen burden evaluated by dilution plating technique on nutrient agar plates.

III. Synthetic schemes

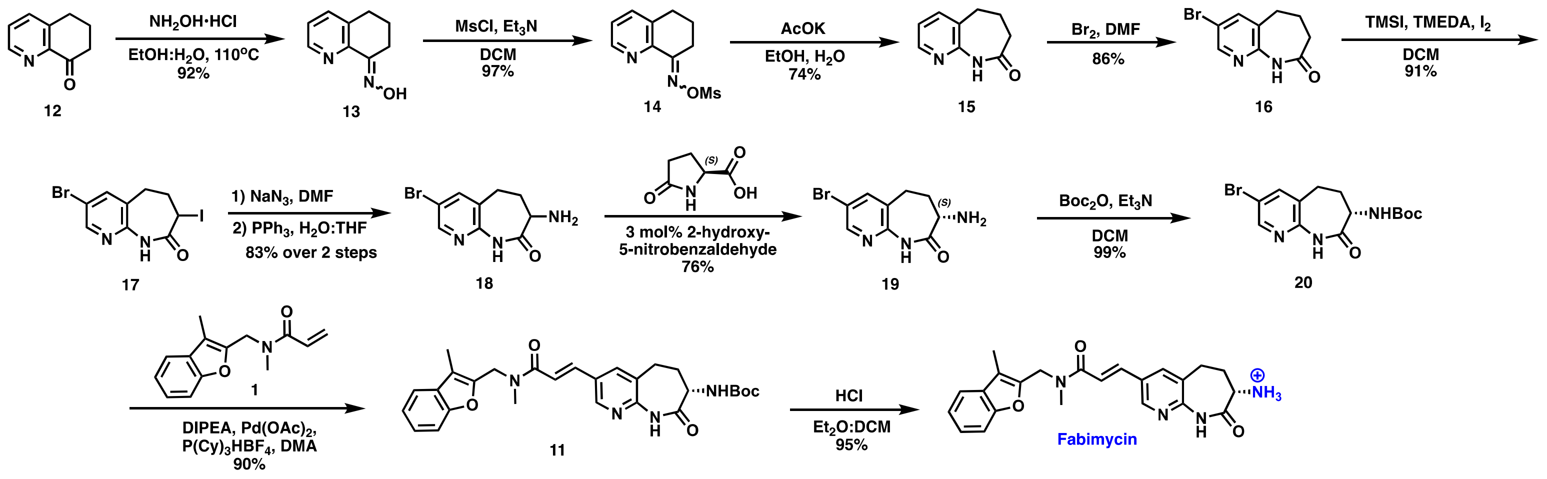

Scheme S1. Synthesis of **fabimycin**.

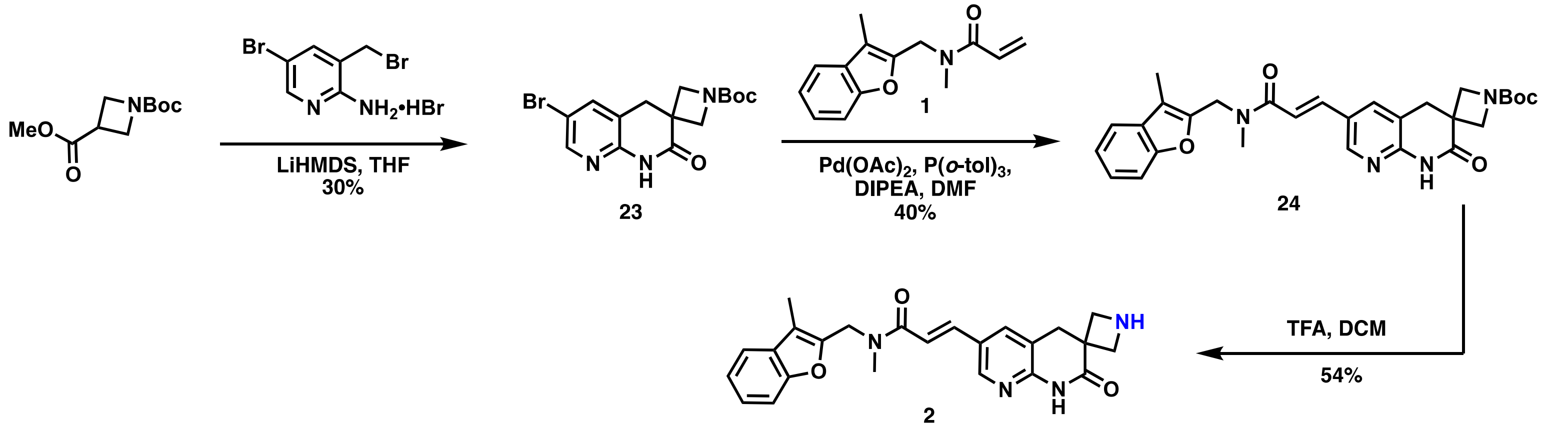

Scheme S2. Synthesis of compound **2**.

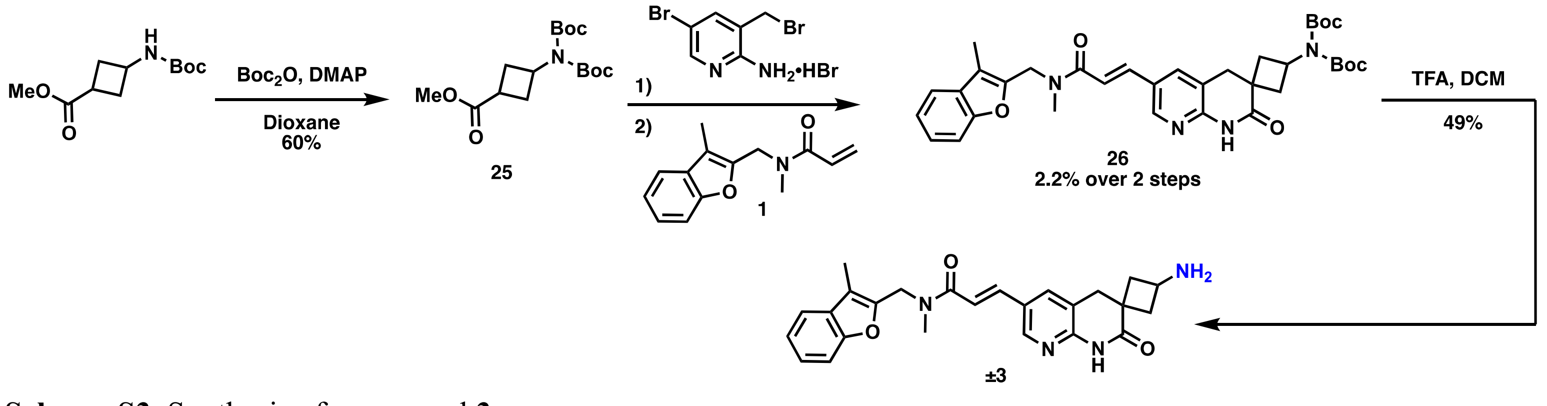

Scheme S3. Synthesis of compound **3**.

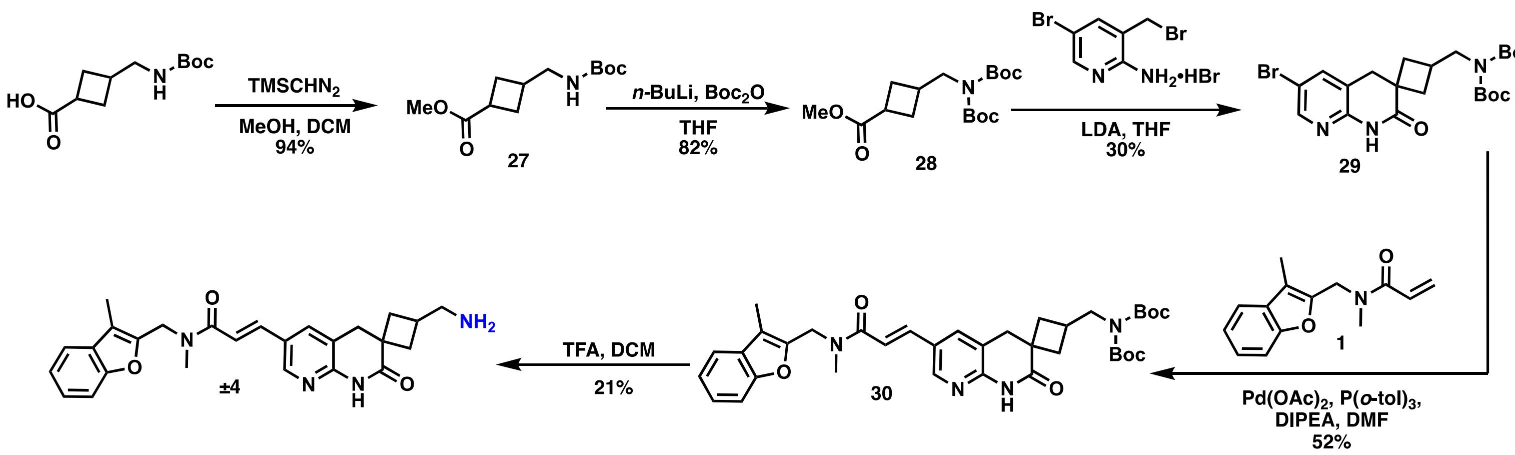

Scheme S4. Synthesis of compound **4**.

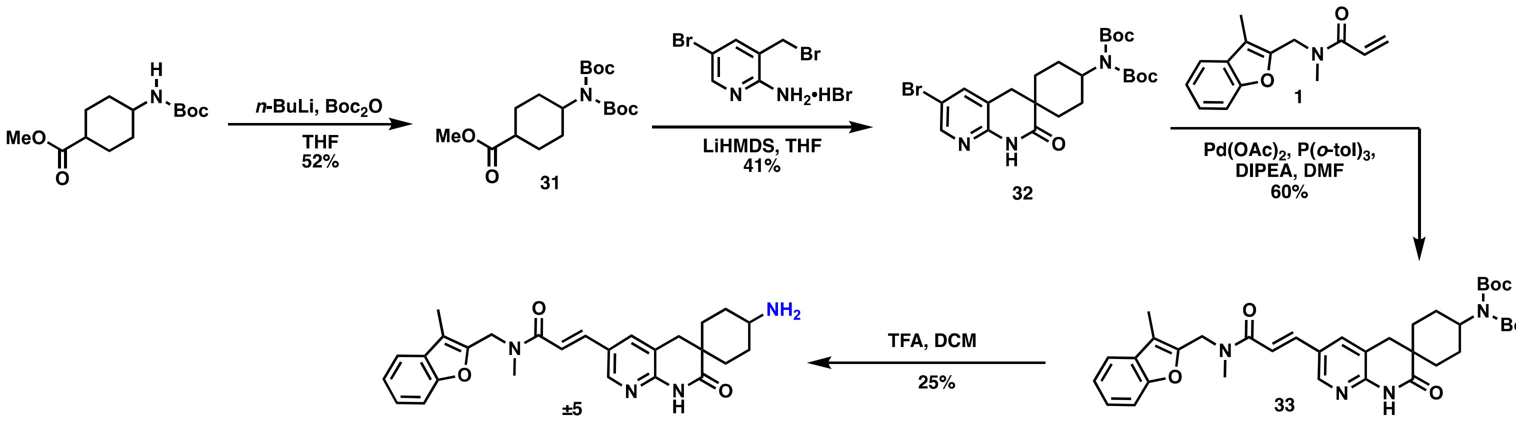

Scheme S5. Synthesis of compound **5**.

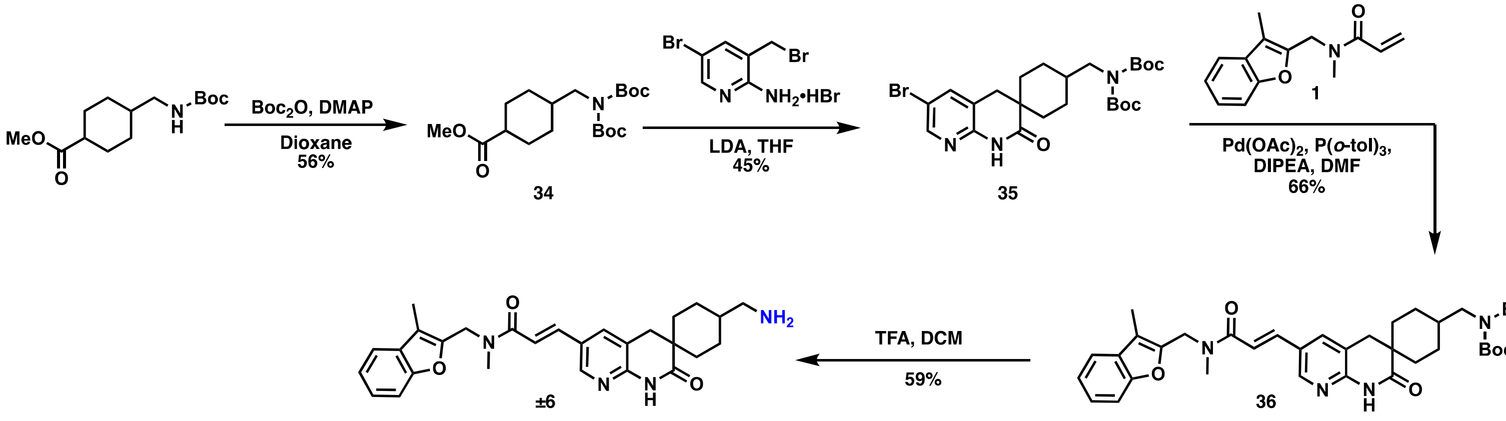

Scheme S6. Synthesis of compound **6**.

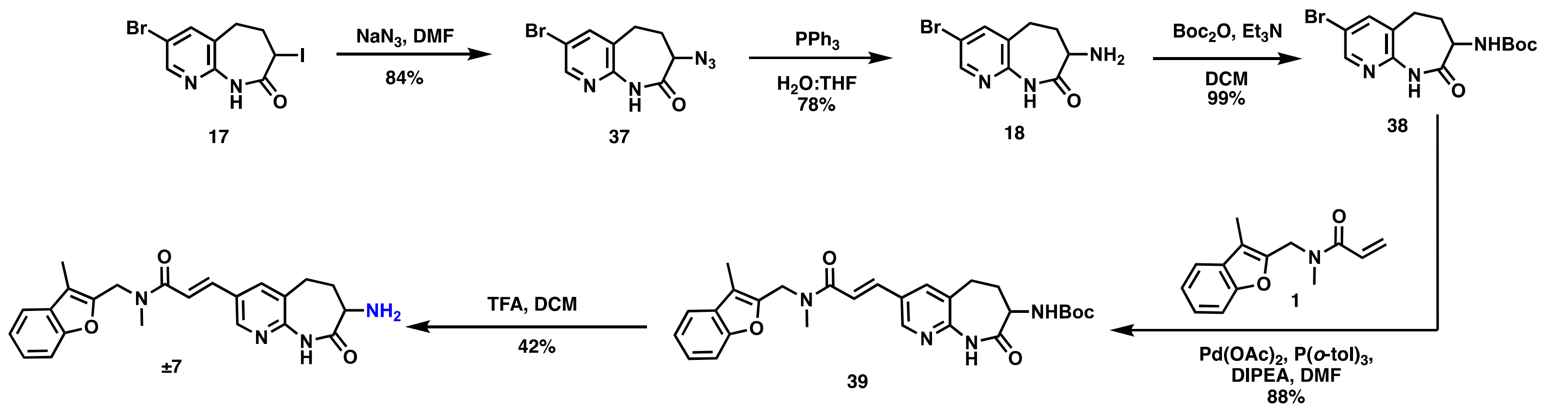

Scheme S7. Synthesis of compound ±**7**.

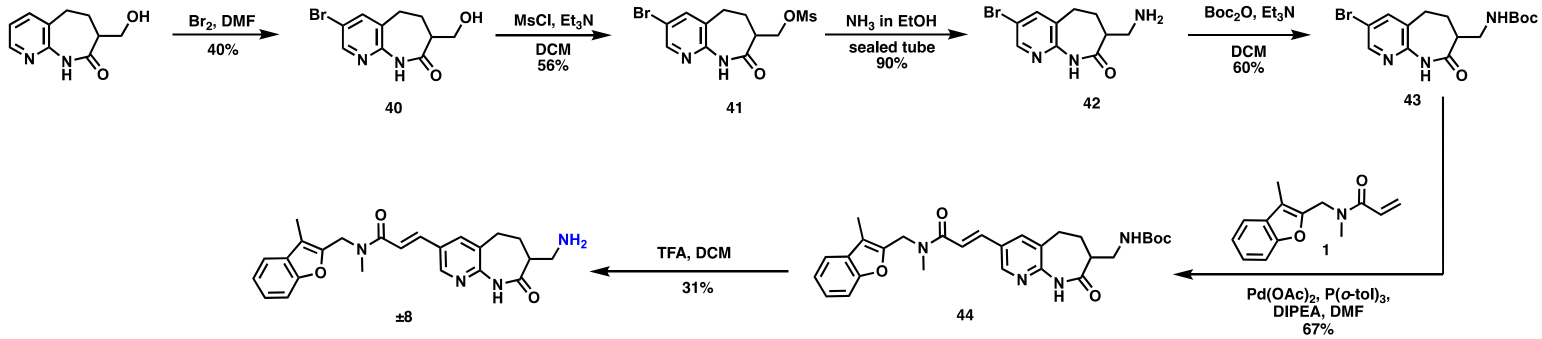

Scheme S8. Synthesis of compound ±**8**.

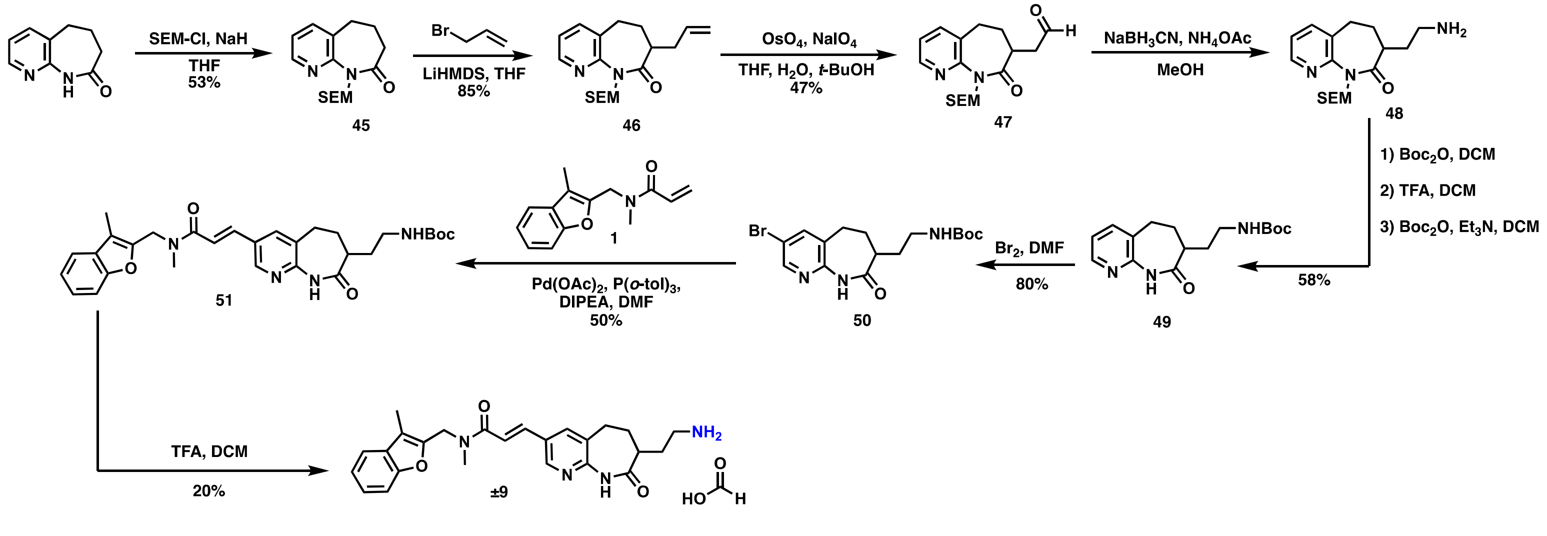

Scheme S9. Synthesis of compound ±**9**.

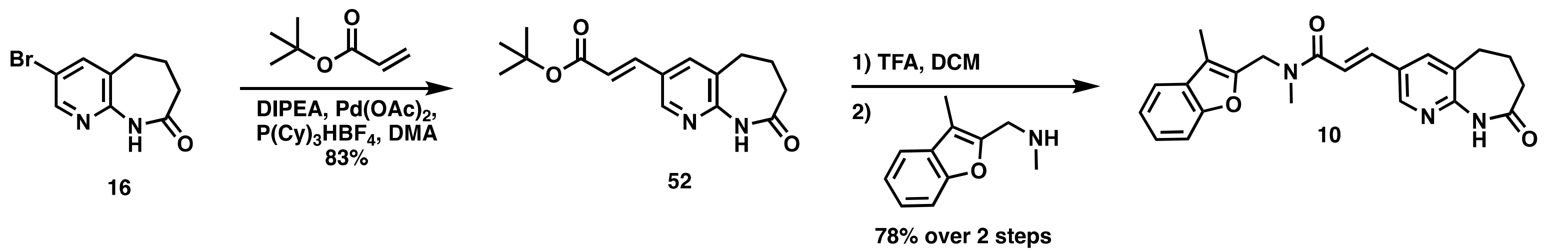

Scheme S10. Synthesis of compound **10**.

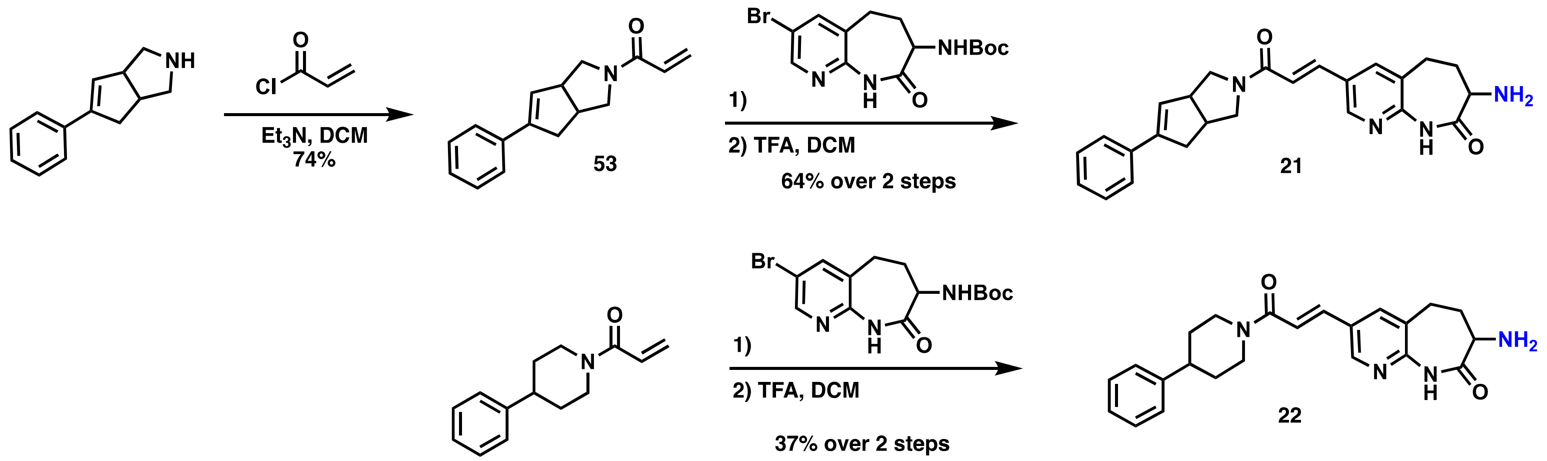

Scheme S11. Synthesis of compounds **21** and **22**.

IV. Synthetic Procedures

General Synthetic Remarks

All reactions were performed under inert atmosphere using nitrogen gas unless otherwise specified. Chemical reagents were purchased from commercial sources and used without further purification. Anhydrous solvents were either purchased from commercial suppliers or obtained after being passed through columns packed with activated alumina under nitrogen positive pressure using a PureSolv MD-5 (Inert, previously Innovative Technology inc.) solvent purification system. Flash chromatography was performed using silica gel (230-400 mesh).

^{1}H, ^{13}C, and 2D NMR experiments were recorded on a Bruker 600 MHz NMR system equipped with a broad-band Prodigy CryoProbe (cooled with liquid nitrogen) and/or Varian Unity Inova 600 MHz NMR system equipped with an autoX broadband probe and/or a Bruker Avance III HD 500 MHz NMR system equipped with a CryoProbe (cooled with liquid helium) and/or NMR instruments from WuXi AppTec. Spectra were obtained in the following solvents (reference peaks also included in ^{1}H and ^{13}C NMRs: deuterated chloroform-*d* (^{1}H NMR 7.26 ppm; ^{13}C NMR 77.16 ppm), DMSO-d_6 (^{1}H NMR 2.50 ppm; ^{13}C NMR 39.52 ppm).All of the chemical shifts are expressed in ppm (δ), coupling constants (*J*, Hz) and peak patterns as broad (br), singlet (s), doublet (d), triplet (t), quartet (q) and multiplet (m). High resolution mass spectra (HRMS) were obtained in the School of Chemical Sciences Mass Spectrometry Laboratory on a Waters Q-TOF Ultima quadrupole time of flight spectrometer using electrospray ionization (ESI). Purity of the final compounds were purified to ≥95% as assessed by an Agilent Technologies 1290 Infinity II LC/MS equipped with a Phenomenex Kinetex column (2.1 mm ID x 50 mm, 1.7 µM particle size, 100 Å pore size).

Compounds **2-9**, **21-51**, and **53-55** were synthesized and characterized by WuXi AppTec.

General Synthetic Procedures:

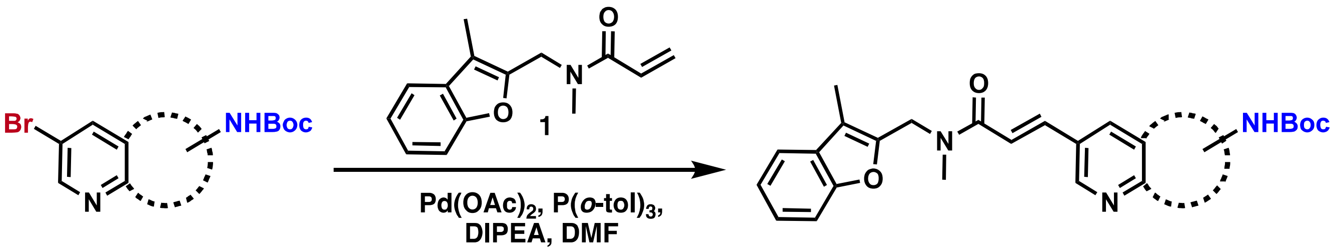

General Procedure I. A flask containing a solution of the bromo-functionalized starting material (**23, 29, 32, 35, 38, 43, 50, 53**; 1.0 equiv.), acrylamide **1** (1.1-1.4 equiv.), DIPEA (2.0 equiv.), Pd(OAc)$_2$ (0.1-0.5 equiv.), and tri(*o*-tolyl)phosphine (0.2-0.3 equiv.) in DMF (0.08M-0.12M) was stirred and heated at 105 °C for 12-16 hours under a nitrogen atmosphere. Upon completion, the reaction was diluted with H_2O and extracted with EtOAc before drying over Na_2SO_4, filtering, and concentrating. The crude material was purified via flash column chromatography (petroleum ether:EtOAc eluent systems) to give desired compounds in 11-88% yield.

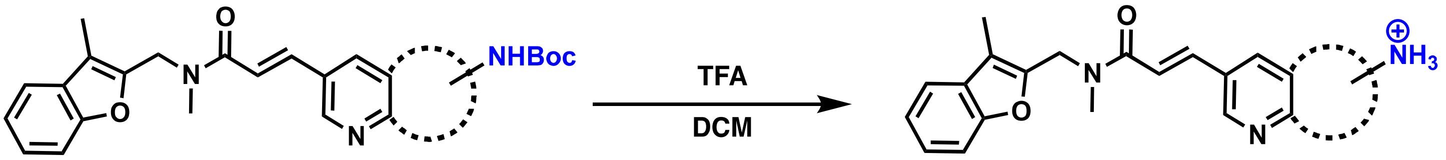

General Procedure II. To a flask containing the Boc-protected starting material (**24, 26, 30, 33, 36, 39, 44, 51**) dissolved in DCM (0.008-0.06M) was added TFA (0.5-5 mL) at 0 ºC. The reaction was stirred at cooler temperatures (0-15 ºC) for 30 min — 2 hours until reaction completion. Organics were evaporated, the residue diluted in organic solvent (DCM, MeOH, or MeCN) and basified to pH 7-8 and concentrated to afford a crude residue. This material was purified via reverse phase Prep-HPLC to give final compounds in 20-59% yield.

Synthetic Procedures

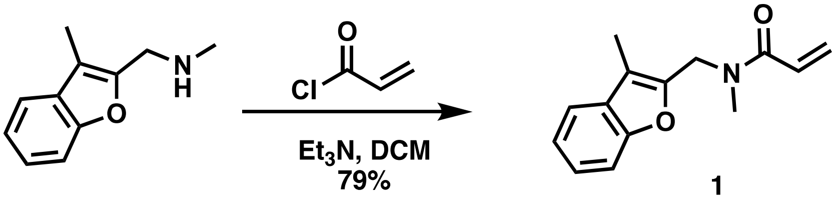

1

Synthetic Procedure 1. *N,N*-diisopropylethylamine (15.4 mmol, 1.5 equiv.) was added dropwise to a solution of *N*-methyl-1-(3-methylbenzofuran-2-yl)methanamine (10.3 mmol, 1.0 equiv.) in DCM (75 mL) at room temperature. After 10 min, acryloyl chloride (20.6 mmol, 2.0 equiv.) was added dropwise and the reaction mixture was stirred overnight. The solvent was removed under reduced pressure and purification by flash column chromatography (1:99 to 3:97, MeOH:DCM) yielded compound **1** in 79% yield (1.861 g) as a colorless oil.

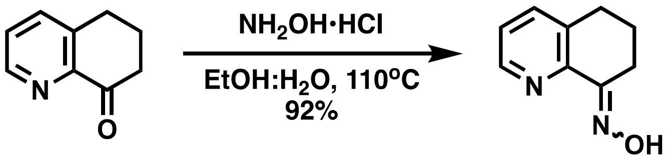

12 13

Synthetic Procedure 2. Hydroxylamine hydrochloride (204 mmol, 2.0 equiv.) and sodium acetate (202 mmol, 1.98 equiv.) was added to a solution of **12** (102 mmol, 1.0 equiv.) in 2:1 EtOH:H_2O (775 mL) and the reaction mixture was heated to 95 °C for 4 hours. The reaction mixture was then concentrated under reduced pressure. The solids were suspended in water and stirred vigorously for 30 min at room temperature before the mixture was filtered and the solid rinsed 3X with 20 mL of water to afford compound **13** in 92% yield (15.14 g).

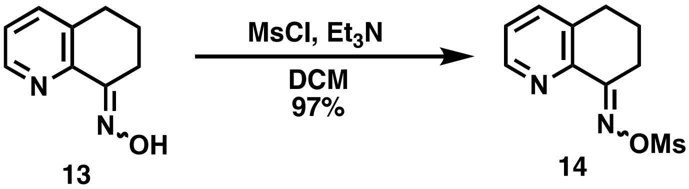

13 14

Synthetic Procedure 3. Methanesulfonyl chloride (186 mmol, 2.0 equiv.) was added dropwise to a solution of **13** (93 mmol, 1.0 equiv.) and triethylamine (465 mmol, 5.0 equiv.) in DCM (560 mL) at 0 °C. The reaction mixture was allowed to return to room temperature and stirred for 1 hour. Then, the reaction mixture was washed with water (3X, 600mL, NaCl added during wash) followed by brine (200 mL). The combined organic extracts were dried over sodium sulfate and concentrated under reduced pressure. Trituration with 1:2 DCM:pentane yielded compound **14** in 97% yield (20.74 g).

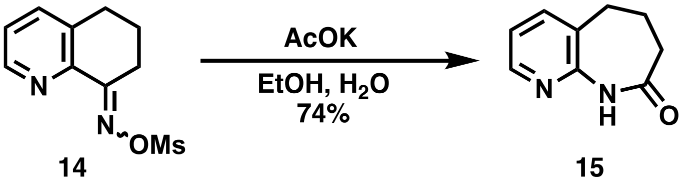

14 15

Synthetic Procedure 4. Potassium acetate (735.6 mmol, 12.26 equiv.) was added to a solution of **14** (60 mmol, 1.0 equiv.) in EtOH:H_2O (1:2, 560 mL total) and the reaction mixture was heated to 110 ºC for 12 hours. Upon completion, the reaction was continually saturated with NaCl during each extraction with EtOAc (5X, 500mL, 20 g NaCl added during first extraction and in smaller increments thereafter). The combined organic extracts were dried over sodium sulfate and concentrated under reduced pressure. Trituration with 1:3 DCM:pentane (72 mL) yielded 6.19 g of **15**. The filtrate from trituration was concentrated under reduced pressure and purification by flash purification column chromatography (10:90 to 60:40, EtOAc:DCM with 1% MeOH) to afford an additional 1.04 g of **15** for a total yield of 74% (7.23 g).

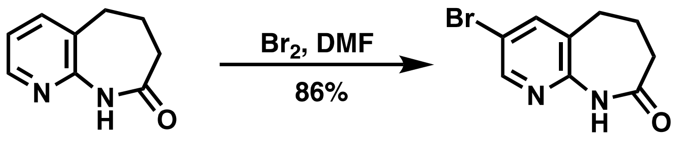

15 **16**

Synthetic Procedure 5. To a solution of **15** (44 mmol, 1.0 equiv.) in DMF (132 mL, 0.33M) was added Br_2 (88 mmol, 2.0 equiv.) at 0 ºC. After addition, the mixture was returned to room temperature and stirred for 2.5 hours. Upon reaction completion, the mixture was quenched with 10% sodium bisulfite (aqueous, 55 mL) followed by careful neutralization with saturated aqueous sodium bicarbonate (150 mL). The solution was cooled to 0 ºC and filtered. The solid was washed with chilled water (4X, 30-40 mL) and dried to afford 8.01 g of compound **16** as a white solid. The filtrate was extracted with EtOAc (4X, 300 mL) and the combined organic extracts were washed with saturated aqueous sodium bicarbonate and brine before being dried over sodium sulfate and concentrated under reduced pressure. Trituration with 1:2 EtOAc:pentane (30 mL) afforded an additional 1.13 g of compound **16** with a total yield of 86% (9.14 g).

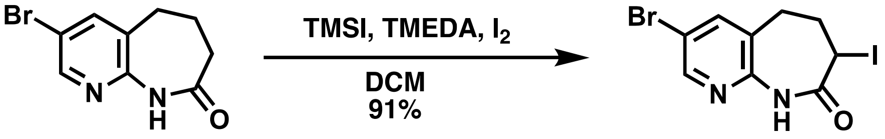

16 **17**

Synthetic Procedure 6. To a solution of **16** (16 mmol, 1.0 equiv.) in dichloromethane (32 mL, 0.5M) was added freshly distilled tetramethylethylenediamine (48 mmol, 3.0 equiv.) and trimethylsilyl iodide (48.5 mmol, 3.03 equiv.) at 0 °C. The reaction mixture was stirred for 40 min followed by the addition of I_2 (24 mmol, 1.5 equiv.) was added. After 1 hour, an additional portion of I_2 was added (16 mmol, 1.0 equiv.) and stirred for an additional 1 hour. Upon reaction completion the mixture was quenched with 10% sodium bisulfite (aqueous) and diluted with water until the total volume of the mixture was 400 mL. The product was extracted with dichloromethane (600 mL, then 300 mL). The combined extracts were dried over Na_2SO_4, filtered, and concentrated under reduced pressure. Purification by flash purification column chromatography (1:99 to 15:85, EtOAc:DCM) afforded compound **17** in 91% yield (5.30 g) as a white solid.

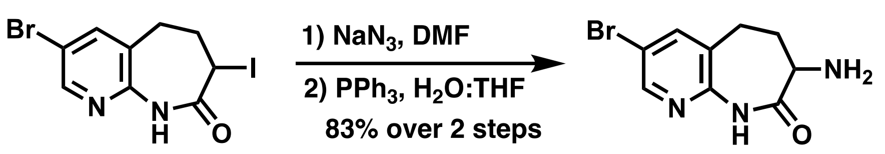

17 **18**

Synthetic Procedure 7. To a solution of **17** (14.23 mmol, 1.0 equiv.) in DMF (100 mL, 0.14M) was added sodium azide (42.7 mmol, 3.0 equiv.). The resulting mixture was stirred at room temperature overnight. Upon reaction completion, the mixture was diluted with water (1000 mL) and extracted with EtOAc (4 X 300 mL). The combined organic layers were dried over Na_2SO_4, filtered, and concentrated under reduced pressure. The crude product was dissolved in THF:H_2O (1:1, 280 mL) and triphenylphosphine added (28.56 mmol, 2.0 equiv.) The resulting mixture was heated to 50 °C overnight. After concentrating to remove THF, the mixture was diluted with water (600 mL) and MeOH (100 mL). The aqueous phase was washed with EtOAc:Et_2O (1:3, 400 mL) to remove triphenylphosphine and triphenylphoshine oxide. The aqueous phase was further washed with ether (100 mL; water added as necessary to avoid emulsion). The combined organic layers were carefully extracted with MeOH:H_2O (1:6, 4X, 150 mL). The aqueous phase was concentrated to remove MeOH. Potassium carbonate (50 g) was added and the aqueous phase was extracted several times with 500 mL portions of $CHCl_3$. The combined organic layers were dried over Na_2SO_4, filtered, and concentrated under reduced pressure. Trituration with 1:1 EtOAc:pentane (50 mL) afforded compound **18** in 83% yield over two steps (3.02 g) as a white solid.

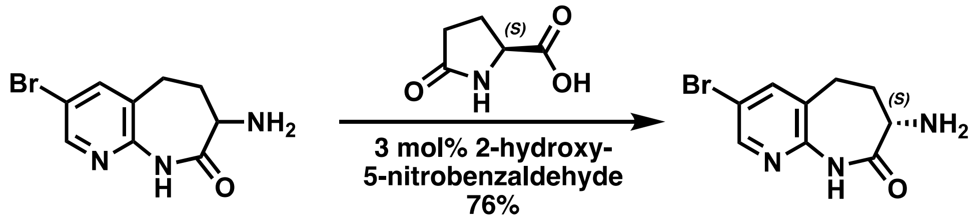

18 19

Synthetic Procedure 8. A solution of 2-hydroxy-5-nitrobenzadehyde (0.04 mmol, 0.01 equiv.) in MeCN (1 mL, 6.68 mg/mL) was made before adding a solution of **18** (4 mmol, 1.0 equiv.) and L-pyroglutamic acid (1 eq, 4 mmol, 1.0 equiv; Sigma-Aldrich, product number: 83160) in MeCN (99 mL). The reaction was stirred vigorously at 50 ºC for 2 days. The reaction mixture was then cooled to 15-25ºC and filtered. The solid was rinsed with chilled MeCN (3X, 20 mL) and dried to yield a crude off-white solid. This was triturated by suspending in 95:5 MeCN:H_2O (61 mL) and stirring for 2 hours at 50 ºC. The reaction was cooled to room temperature, filtered, and dried. This trituration was repeated to yield a white solid. This was suspended in dilute ammonium hydroxide (1:24 NH_4OH:H_2O, 100 mL) and stirred for 15 minutes before extracting the free-base product with DCM (3X, 50 mL), drying over Na_2SO_4, filtering, and concentrating in vacuo to afford compound **19** in 76% yield (777 mg, 98.6% *ee*) as a white solid.

Note: To monitor reaction progress 0.75 mL-1.0 mL of the reaction mixture was aliquoted into a small vial followed by the addition of 1:24 NH_4OH:H_2O (1 mL) and stirred for 5 min. The product was extracted with DCM (2X, 1mL), the combined organic extracts were dried over Na_2SO_4, filtered, and concentrated. Enantiopurity of the free base was determined by ^1H NMR ($CDCl_3$, 500 MHz) with the addition of 2-3 mg of (S)-(−)-1,1′-Bi(2-naphthol) as a chiral solvating agent.

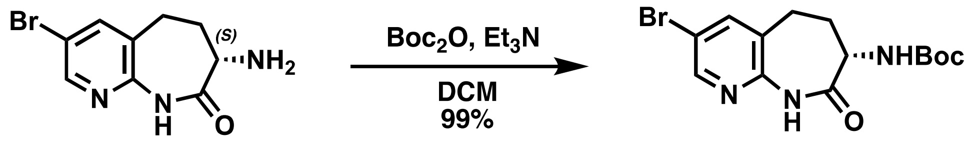

19 20

Synthetic Procedure 9. Triethylamine (4.5 mmol, 1.5 equiv.) was added dropwise to a solution of **19** (3 mmol, 1.0 equiv.) in DCM (75 mL) at 0 ºC followed by the dropwise addition of di-*tert*-butyl dicarbonate (3.3 mmol, 1.1 equiv.).

The reaction mixture was allowed to warm to room temperature and stirred overnight. The reaction was quenched with saturated aqueous sodium bicarbonate and extracted with DCM. The combined organic extracts were washed with brine, dried over sodium sulfate, and concentrated under reduced pressure. Purification by flash purification column chromatography (5:95 to 60:40, EtOAc:DCM) yielded compound **20** in 99% yield (1.06 g) as a white solid.

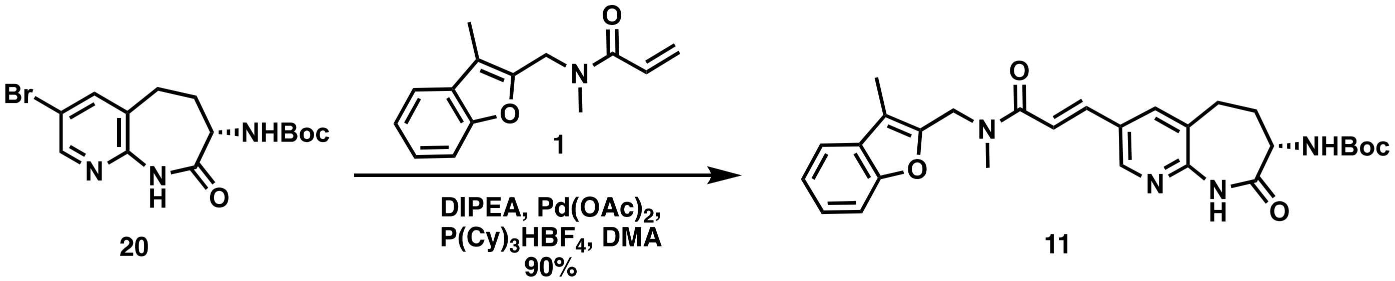

Synthetic Procedure 10. Anhydrous dimethylacetamide (32 mL, sparged with N_2 before use) was added to a flask containing **1** (4.425 mmol, 1.5 equiv.), **20** (2.95 mmol, 1.0 equiv.), palladium(II) acetate (0.148 mmol, 0.05 equiv.), and tricyclohexylphosphine tetrafluoroborate (0.295 mmol, 0.1 equiv.) followed by the addition of DIPEA (8.85 mmol, 3.0 equiv.; distilled and sparged with N_2 before using). The reaction mixture was placed in a pre-heated oil bath and stirred at 98-102 ºC for 24 hours. Upon completion, the reaction mixture was diluted with EtOAc and filtered through a pad of celite where the filtrate was washed with saturated aqueous sodium bicarbonate. The combined organic extracts were dried over sodium sulfate and concentrated under reduced pressure. A slurry was made with the crude residue, a small amount of activated charcoal, and silica before concentrating and performing flash column chromatography on the charged silica (dry-loading) using an eluent system of 5:95 to 80:20 (EtOAc:DCM with 1% MeOH) to afford compound **11** in 90% yield (1.34 g) as an off-white solid.

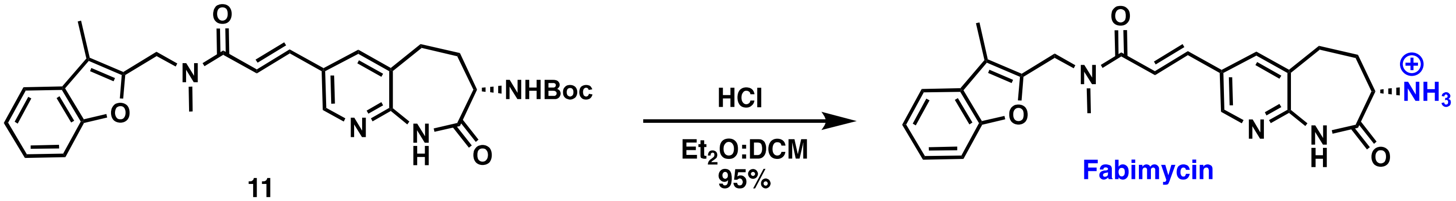

Synthetic Procedure 11. A suspension of **11** (2.4 mmol, 1.0 equiv.) in 1:1 DCM:HCl, 1M in ether (30 mL) was stirred at room temperature for 3 hours. Upon completion, the reaction mixture was concentrated from $CHCl_3$ several times followed by trituration with MeOH:$CHCl_3$:ether (1:1:18, 40 mL). The resulting gel was rinsed with ether (3X, 10-20 mL) with thorough mixing, and dried to afford **fabimycin** in 95% yield (1.01 g) as an off-white solid.

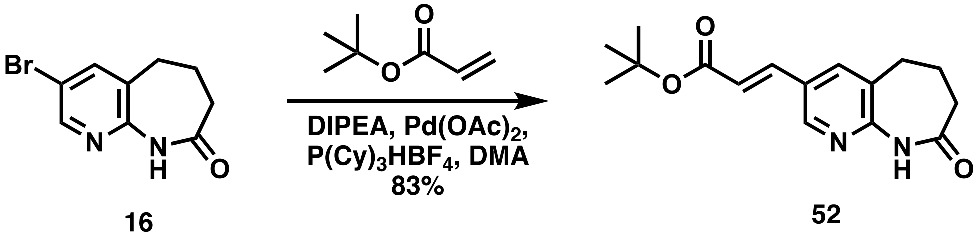

Synthetic Procedure 12. Anhydrous dimethylacetamide (32 mL, sparged with N_2 before using) was added to a flask containing **16** (2.5 mmol, 1.0 equiv.), palladium(II) acetate (0.125 mmol, 0.05 equiv.), and tricyclohexylphosphine tetrafluoroborate (0.25 mmol, 0.1 equiv.) followed by the addition of *tert*-butyl acrylate (3.75 mmol, 1.5 equiv.; sparged with N_2 before using), DIPEA (3.75 mmol, 1.5 equiv; distilled and sparged with N_2 before using). The reaction mixture

was heated to 130 ºC under microwave irradiation for 1 hour. Upon reaction completion, the reaction was diluted with EtOAc and filtered through a pad of celite. The filtrate was washed with saturated sodium bicarbonate. The combined organic extracts were dried over sodium sulfate and concentrated under reduced pressure. Purification by flash purification column chromatography (10:90 to 40:60, EtOAc:DCM) followed by trituration with ether/pentane yielded compound **52** in 83% yield (596 mg) as a white solid.

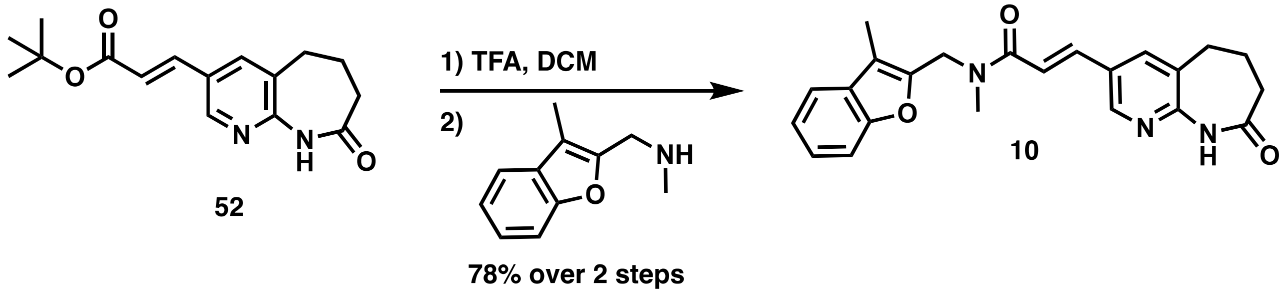

Synthetic Procedure 13. Compound **52** (2 mmol, 1.0 equiv.) was dissolved trifluoroacetic acid:DCM(1:1, 16 mL) and stirred at room temperature for 2 hours. The reaction mixture was then concentrated several times from DCM before suspending the crude material in 4 M HCl in dioxane (8 mL), stirring for 30 min, filtering, and rinsing with ether to afford the crude HCl salt intermediate as a white solid and used without further purification.

For step 2, a portion of this intermediate (0.5 mmol, 1.0 equiv.) was dissolved in dimethylacetamide (2 mL) followed by the addition of *N*-methyl-1-(3-methylbenzofuran-2-yl)methanamine (0.5 mmol, 1.0 equiv.), 1-hydroxy-7-azabenzotriazole (0.55 mmol, 1.1 equiv.), DIPEA (1.1 mmol, 2.2 equiv.), and 1-ethyl-3-(3-dimethylaminopropyl)carbodiimide (0.5 mmol, 1.1 equiv.). This was heated to 60 ºC for 12 hours. Upon reaction completion, the mixture was diluted with saturated sodium bicarbonate and extracted with chloroform. The combined organic extracts were dried over sodium sulfate and concentrated under reduced pressure. Purification by flash purification column chromatography (1:99 to 8:92, MeOH:$CHCl_3$) followed by trituration with 2:1 ether/pentane to afford compound **10** in 78% overall yield (163 mg) as an off-white solid.

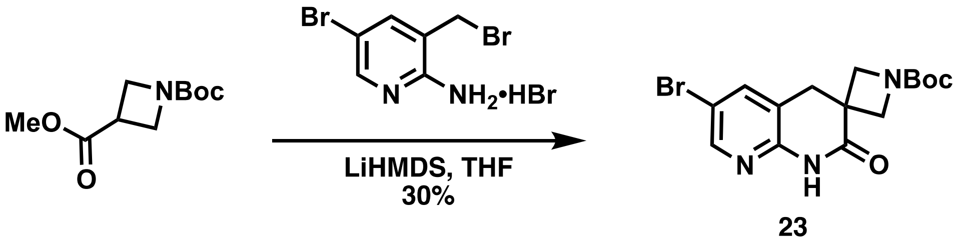

Synthetic Procedure 14. To a solution of 1-*tert*-butyl 3-methyl azetidine-1,3-dicarboxylate (1.00 g, 4.6 mmol, 2.0 equiv.) in THF (100 mL, 0.05M) was added LiHMDS (9.3 mmol, 4 equiv.) at -78 °C. The reaction was stirred at -78 to -10 °C for 1 hour. Then, 5-bromo-3-(bromomethyl)pyridin-2-amine (2.3 mmol, 1.0 equiv., HBr salt) was added and the mixture stirred at -78 °C for 1 hour. The reaction mixture was then warmed to 25 °C for 12 hours. Upon reaction completion, the mixture was poured into saturated NH_4Cl solution (100 mL) at 0 °C. This mixture was stirred at 0 °C for 1 hour before being extracted with EtOAc (3 x 30 mL). The combined organic layers were washed with brine (50 mL), dried over Na_2SO_4, filtered, and concentrated in vacuo to give provide a crude residue. This was purified by column chromatography (100% EtOAc) to afford compound **23** as a yellow solid in 30% yield (460 mg).

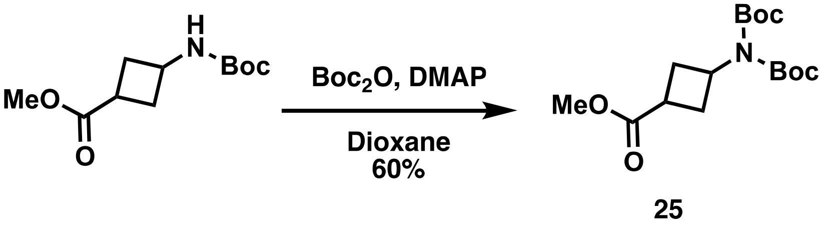

Synthetic procedure 15. To a solution of methyl 3-(tert-butoxycarbonylamino)cyclobutanecarboxylate (1.58 g, 6.89 mmol, 1.0 equiv.) and Boc_2O (4.51 g, 20.6 mmol, 3.0 equiv.) in dioxane (80 mL, 0.09M) was added DMAP (420 mg, 3.45 mmol, 0.5 equiv.). The reaction mixture was stirred at 120 °C for 20 hours under nitrogen atmosphere. Then, additional Boc_2O (3.01 g, 13.7 mmol, 2.0 equiv.) was added. The reaction mixture was stirred at 120 °C for 4 hours under nitrogen atmosphere. Upon reaction completion, the mixture was concentrated in vacuo to provide a crude residue. This was purified by column chromatography (petroleum ether/EtOAc = 50:1) to afford the compound **25** in 60% yield (1.38 g) as a yellow oil.

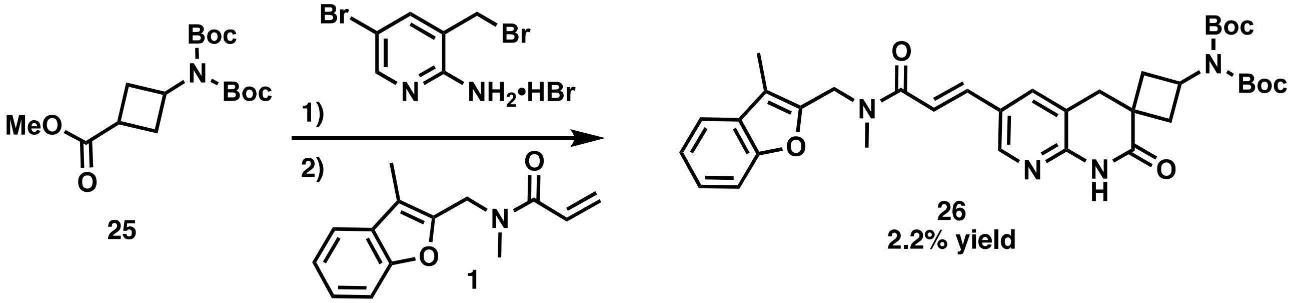

Synthetic procedure 16. To a flask containing LiHMDS (10 mmol, 5 equiv.) in THF (30 mL) was added a solution of **25** (1.33 g, 4.04 mmol, 2 equiv.) in THF (30 mL) at -70 °C. The reaction mixture was stirred at -70 °C for 1 hour under nitrogen atmosphere. Then, 5-bromo-3-(bromomethyl)pyridin-2-amine (700 mg, 2.02 mmol, 1. 0 equiv.) was added. This was warmed to 20 °C and stirred for 16 hours under nitrogen atmosphere. Upon reaction completion, the mixture was diluted with saturated NH_4Cl solution (20 mL) and EtOAc (20 mL). The aqueous phase was extracted with EtOAc (3 X 30 mL) before the combined organic layers were concentrated in vacuo to provide a crude residue. This was purified by column chromatography (EtOAc:MeOH = 10:1) and used in the next step without further purification. The Heck coupling to afford compound **26** was performed according to **general procedure 1** to afford compound **26** in 2.2% yield overall.

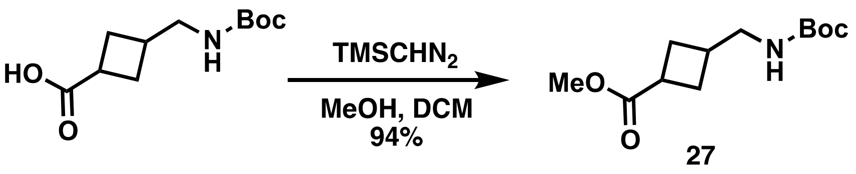

Synthetic procedure 17. To a solution of 3-[(tert-butoxycarbonylamino)methyl]cyclobutanecarboxylic acid (2.60 g, 11.3 mmol, 1.0 equiv.) in equal parts DCM and MeOH (60 mL total, 0.19M) was added $TMSCHN_2$ (34 mmol, 3 equiv.) at 0 °C. The reaction mixture was stirred at 15 °C for 3 hrs. Upon completion, the reaction was concentrated in vacuo. The crude residue was purified by column chromatography (petroleum ether:EtOAc = 50:1) to afford compound **27** in 94% yield (2.60 g) as colorless oil.

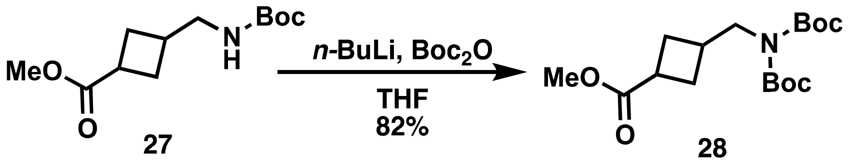

27 28

Synthetic procedure 18. To a solution of **27** (2.60 g, 10.7 mmol, 1.0 equiv.) in THF (5 mL, 2.1M) was added *n*-BuLi (12.75 mmol, 1.2 equiv.) at -78 °C. The reaction mixture was stirred for 30 min before adding a solution of Boc_2O (3.5 g, 16 mmol, 1.5 equiv.) in THF (2 mL) dropwise. The reaction was warmed to 10 °C and stirred for 12 hrs. Upon completion, the reaction was quenched with saturated NH_4Cl solution and extracted with EtOAc (3 x 30 mL). The combined organic layers were washed with brine, dried over Na_2SO_4, filtered, and concentrated in vacuo to provide a crude residue. This residue was purified by column chromatography (petroleum ether:EtOAc = 20:1) to afford compound **28** in 82% yield (3.00 g) as colorless oil.

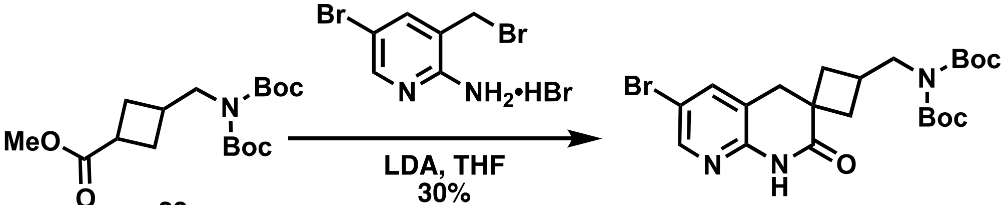

28 29

Synthetic procedure 19. To a solution of LDA (8.64 mmol, 4 equiv.) in THF (100 mL) was added a solution of **28** (1.49 g, 4.32 mmol) in THF (15 mL) at -78 °C. The reaction mixture was stirred at -78 °C for 1 hour. Then, 5-bromo-3-(bromomethyl)pyridin-2-amine (750 mg, 2.16 mmol, 1.0 equiv.) was added in one-portion. The reaction mixture was then warmed to 10 °C and stirred for 12 hours. Upon completion the reaction was diluted with saturated NH_4Cl solution (70 mL) and EtOAc (20 mL). The aqueous phase was extracted with EtOAc (80 mL) before combining organic layers and drying over Na_2SO_4, filtering, and concentrating in vacuo to provide a crude residue. This was purified by column chromatography (petroleum ether:EtOAc = 2:1) to afford compound **29** in 30% yield (430 mg) as yellow oil.

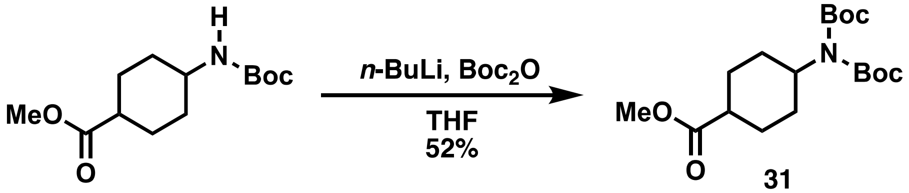

31

Synthetic procedure 20. To a solution of methyl 4-(tert-butoxycarbonylamino)cyclohexanecarboxylate (1.80 g, 7.00 mmol, 1.0 equiv.) in THF (50 mL, 0.14M) was added *n*-BuLi (9.1 mmol, 1.3 equiv.) at -78 °C. This was stirred at -78°C for 30 minutes before adding Boc_2O (1.83 g, 8.39 mmol, 1.2 equiv.). The reaction mixture was warmed to 20 °C and stirred for 12 hrs. Upon completion, the reaction was quenched with water (100 mL) and extracted with EtOAc (3 x 50 mL). The combined organic layers were washed with brine, dried over Na_2SO_4, filtered, and concentrated in vacuo. The crude residue was purified by column chromatography (petroleum ether: EtOAc = 20:1) to afford compound **31** in 52% yield (1.30 g) as a colorless oil.

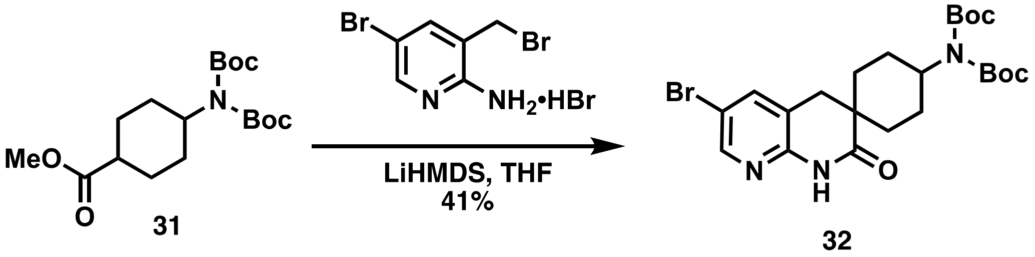

Synthetic procedure 21. A solution of **31** (1.55 g, 4.32 mmol, 2.0 equiv.) in THF (5 mL) was added to LiHMDS (7.14 mL, 7.14 mmol, 3.3 equiv.) at -78 °C and the reaction was allowed to stir at this temperature for 1 hr. Then, 5-bromo-3-(bromomethyl)pyridin-2-amine (750 mg, 2.16 mmol, 1.0 equiv.) was added. The reaction mixture was stirred at 10 °C for 12 hrs. Upon completion, the reaction was quenched with water (10 mL), extracted with EtOAc (3 x 50 mL). The combined organic layers were washed with brine, dried over Na_2SO_4, filtered, and concentrated in vacuo. The crude residue was purified by reverse phase flash chromatography (0.5% NH_3•H_2O) to afford compound **32** in 41% yield (450 mg) as a white solid.

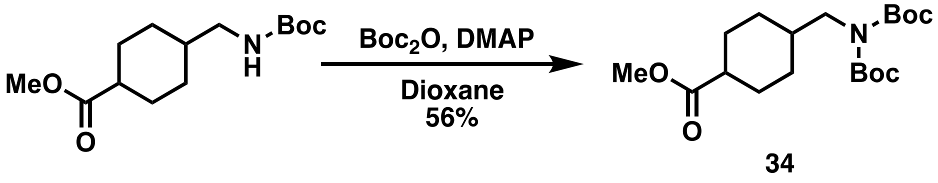

Synthetic procedure 22. A solution of methyl 4-[(tert-butoxycarbonylamino)methyl]cyclohexanecarboxylate (4.70 g, 17.3 mmol, 1.0 equiv.) and DMAP (423 mg, 3.46 mmol, 0.2 equiv.) in dioxane (100 mL, 0.17M) was made before adding Boc_2O (11.3 g, 51.9 mmol, 3 equiv.). This was stirred at 102 °C for 12 hours. Additional Boc_2O (11.3 g, 51.9 mmol, 3 equiv.) and DMAP (423 mg, 3.46 mmol, 0.2 equiv.) was added and the reaction stirred at 102 °C for another 12 hours. Upon completion, the reaction was concentrated in vacuo to provide a crude residue which was purified by column chromatography (petroleum ether:EtOAc = 30:1) to afford compound **34** in 56% yield (3.66 g) as a colorless oil.

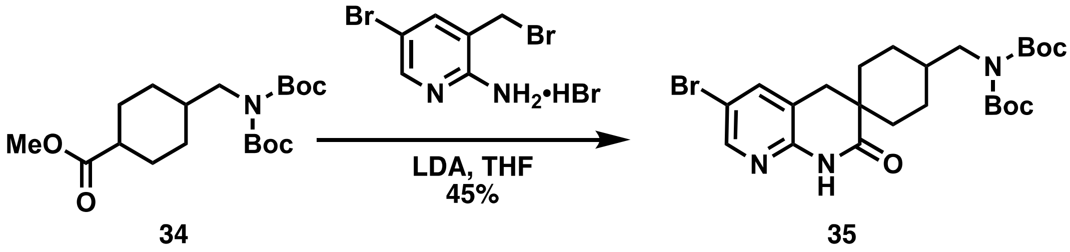

Synthetic procedure 23. To a solution of LDA (3.46 mmol, 4 equiv.) in THF (20 mL) was added a solution of **34** (642 mg, 1.73 mmol, 2 equiv.) in THF (10 mL) at -78 °C and stirred for 1 hour. Then, 5-bromo-3-(bromomethyl)pyridin-2-amine (300 mg, 864 μmol, 1.0 equiv.) was added in one portion. The reaction mixture was stirred for 30 min, then warmed to 15 °C and stirred for 12 hours. Upon completion, saturated NH_4Cl solution (30 mL) and EtOAc (20 mL) was added. The aqueous phase was extracted with EtOAc (30 mL). The combined organic layers were dried over Na_2SO_4, filtered, and concentrated in vacuo to provide a crude residue. This was purified by column chromatography (petroleum ether:EtOAc = 2:1) to afford compound **35** in 45% yield (208 mg) as yellowish solid.

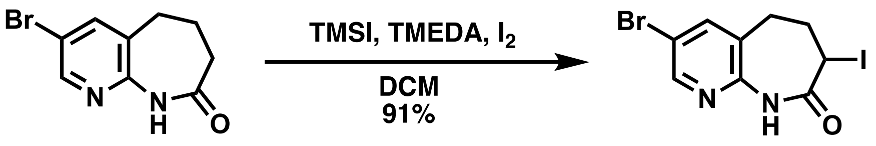

17

Synthetic procedure 24. A solution of 3-bromo-5,6,7,9-tetrahydropyrido[2,3-b]azepin-8-one (16 mmol, 1.0 equiv.) in DCM (32 mL) was made before adding freshly distilled TMEDA (148 mmol, 3.0 equiv.) and TMSI (48.5 mmol, 3.03 equiv.) at 0 °C. After stirring for 40 min, I_2 (24 mmol, 1.5 equiv..) was added. The resulting mixture was stirred at 0 °C for 1 hour before an additional portion of I_2 was added (16 mmol, 1.0 equiv.) and again stirred for 1 hour. Upon completion, the reaction was quenched with 10% sodium bisulfite (aq.) and diluted with water until the total volume of the mixture was 400 mL. This was extracted with DCM (600 mL, then 300 mL). The combined extracts were dried over sodium sulfate, filtered, and concentrated under reducted pressure. This residue was purified by column chromatography (1:99 to 15:85, EtOAc:DCM) to afford compound **17** in 91% yield (5.30 g) as a white solid.

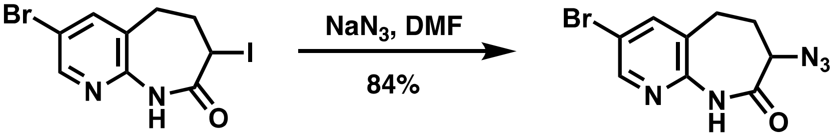

17 37

Synthetic procedure 25. In route to synthesize compound ±**7** (**Scheme S7**): To a solution of **17** (1.00 g, 2.72 mmol, 1.0 equiv.) in DMF (20 mL, 0.14M) was added NaN_3 (531 mg, 8.17 mmol, 3 equiv.). The resulting mixture was stirred at 25 °C for 12 hours. Upon completion, the reaction mixture was diluted with H_2O (100 mL) and extracted with EtOAc (2 X 200 mL). The combined organic layers were dried over Na_2SO_4, filtered, and concentrated in vacuo to afford compound **37** in 84% yield (650 mg) as a yellow solid.

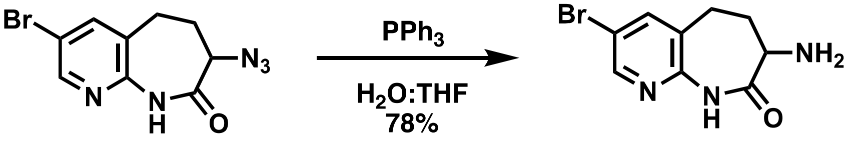

37 18

Synthetic procedure 26. In route to synthesize compound ±**7** (**Scheme S7**): A solution of **37** (0.65 g, 2.30 mmol, 1.0 equiv.) in equal parts THF and H_2O (40 mL total, 0.06M) was made before addition of PPh_3 (1.21 g, 4.61 mmol, 2.0 equiv.). The resulting mixture was stirred at 50 °C for 12 hours. Upon completion, the reaction mixture was concentrated in vacuo to remove the organic solvent. The aqueous phase was extracted with EtOAc (3 X 30 mL). The combined organic layers were then dried over Na_2SO_4, filtered, and concentrated in vacuo to produce a crude brown solid. This was triturated with (petroleum ether:EtOAc=1:2, 30 mL) to afford compound **18** in 78% yield (700 mg) as a deep pink solid.

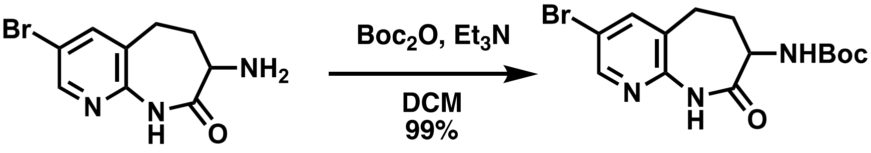

18 38

Synthetic procedure 27. To a solution of **18** (700 mg, 1.80 mmol, 1.0 equiv.) in DCM (100 mL, 0.018M) was added Et_3N (2.71 mmol, 1.5 equiv.) and Boc_2O (433 mg (455 μL), 1.98 mmol, 1.1 equiv.). The resulting mixture was stirred at 25 °C for 3 hours. Upon completion, the reaction was quenched with H_2O (100 mL) and extracted with DCM (100 mL). The combined organic layers were dried over Na_2SO_4, filtered, and concentrated in vacuo to give a brown solid. This was purified by column chromatography (EtOAc:MeOH = 10:1) to afford compound **38** in 99% yield (0.85 g) as a red solid.

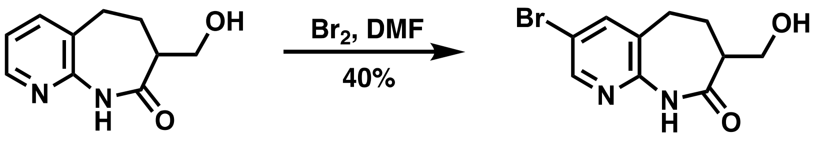

40

Synthetic procedure 28. To a solution of 7-(hydroxymethyl)-5,6,7,9-tetrahydropyrido[2,3-b]azepin-8-one (1 g, 5.20 mmol, 1.0 equiv.) in DMF (15 mL, 0.35M) was added Br_2 (997 mg, 6.24 mmol, 1.2 equiv.) drop-wise at 0 °C. The resulting mixture was stirred at 25 °C for 2 hours. Upon completion, the reaction was poured into H_2O (100 mL) and extracted with EtOAc (2 X 120 mL). The combined organic layers were dried over Na_2SO_4, filtered, and concentrated in vacuo to give crude brown oil. This was purified by column chromatography (EtOAc) to afford compound **40** in 40% yield (570 mg) as a yellow solid.

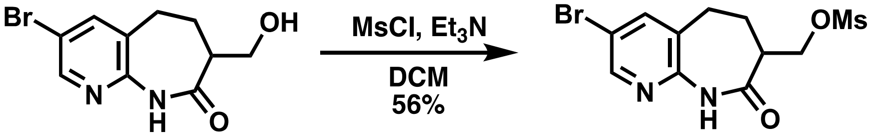

40 41

Synthetic procedure 29. To a solution of **40** (560 mg, 2.07 mmol, 1.0 equiv.) in DCM (15 mL, 0.14M) was added mesyl chloride (0.845 g, 7.38 mmol, 3.5 equiv.) and Et_3N (836 mg, 8.26 mmol, 4 equiv.) at 0 °C. The resulting mixture was stirred at 15 °C for 16 hours. Upon completion, the reaction was quenched with H_2O (10 mL). The aqueous phase was extracted with DCM (10 mL). The combined organic layers were dried over Na_2SO_4, filtered, and concentrated in vacuo to give a crude brown residue. The residue was triturated with petroleum ether:EtOAc=3:1 (4 mL total) to afford compound **41** in 56% yield (410 mg) as a yellow solid.

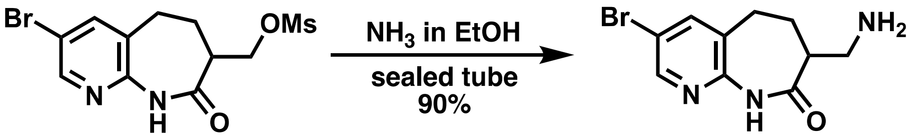

41 42

Synthetic procedure 30. To a solution of **41** (160 mg, 458 umol, 1.0 equiv.) in EtOH (10 mL, 0.05M) was added NH_3/EtOH (20 mL). The resulting mixture was stirred at 80 °C for 9 hours in a sealed tube. Upon completion, the mixture was concentrated in vacuo to afford compound **42** in 90% yield (150 mg) as a brown oil.

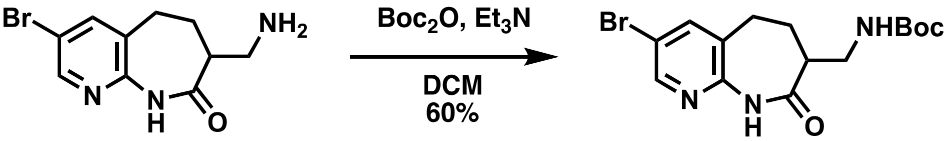

42 **43**

Synthetic procedure 31. To a solution of **42** (150 mg, 333 µmol, 1.0 equiv.) in DCM (40 mL, 0.008M) was added Boc_2O (727 mg, 3.33 mmol, 10 equiv.) and Et_3N (937 µL, 6.74 mmol, 20 equiv.). The resulting mixture was stirred at 15 °C for 2 hours. Upon completion, the reaction was concentrated in vacuo to give a crude brown residue. This was purified by column chromatography (petroleum ether:EtOAc = 1:1) to afford compound **43** in 60% yield (120 mg) as a yellow solid.

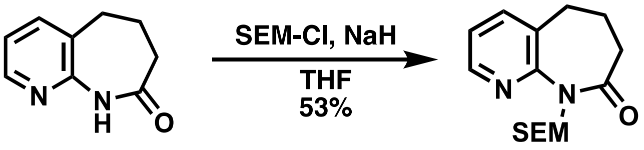

45

Synthetic procedure 32. To a mixture of NaH (2.96 g, 73.9 mmol, 1.5 equiv.) in DMF (150 mL) was added a solution of 5,6,7,9-tetrahydropyrido[2,3-b]azepin-8-one (8.00 g, 49.3 mmol, 1.0 equiv.) in DMF (50 mL) at 0 °C. The reaction was stirred at 15 °C for 1 hour. Then, 2-(trimethylsilyl)ethoxymethyl chloride (12.3 g, 73.9 mmol, 1.5 equiv.) was added slowly at 0 °C. The reaction mixture was then stirred at 15 °C for 16 hours. Upon completion, saturated NH_4Cl solution (300 mL) was added to the reaction and the mixture was extracted with EtOAc (3 X 400 mL). The combined organic layers were dried over Na_2SO_4, filtered, and concentrated in vacuo to provide a crude brown residue. This was purified by column chromatography (petroleum ether:EtOAc = 2:1) to afford compound **45** in 53% yield (7.70 g) as a yellow oil.

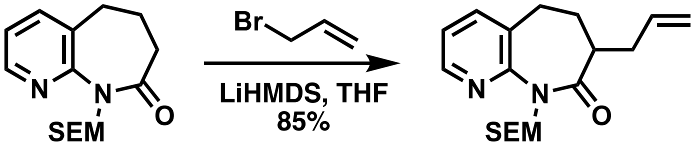

45 **46**

Synthetic procedure 33. To a solution of **45** (4.20 g, 14.3 mmol, 1.0 equiv.) in THF (500 mL, 0.03M) was added LiHMDS (43.0 mmol, 3.0 equiv.) at -70 °C. This was stirred at 15 °C for 1 hour before adding 3-bromoprop-1-ene (6.95 g, 57.4 mmol, 4.0 equiv.) at -70 °C. The reaction mixture was warmed up to 15 °C and stirred for 2 hours. Upon completion, saturated NH_4Cl aqueous solution (300 mL) was added. The mixture was extracted with EtOAc (2 X 300 mL) and the combined organic layers were dried over Na_2SO_4, filtered, and concentrated in vacuo to provide a crude yellow residue. This was purified by column chromatography (petroleum ether:EtOAc = 10:1) to afford compound **46** in 85% yield (4.10 g) as a yellow oil.

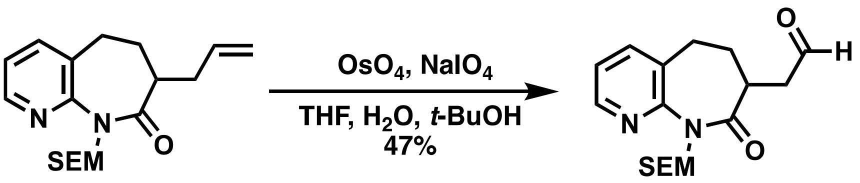

46 **47**

Synthetic procedure 34. To a solution of **46** (2.30 g, 6.92 mmol, 1.0 equiv.) in equal parts THF and H_2O (60 mL total, 0.115M) was added a solution of OsO_4 (71.7 µL, 1.38 mmol, 0.2 equiv.) in *t*-BuOH (2 mL). Then, sodium periodate (2.30 g, 10.7 mmol, 1.55 equiv.) was added. The reaction was stirred at 15 °C for 30 minutes. Upon completion, the reaction was quenched with H_2O (50 mL) and extracted with EtOAc (3 X 50 mL). The combined organic layers were dried over Na_2SO_4, filtered, and concentrated in vacuo to provide a crude black residue. This was purified by column chromatography (petroleum ether:EtOAc = 3:1) to afford compound **47** in 47% yield (1.10 g) as deep green oil.

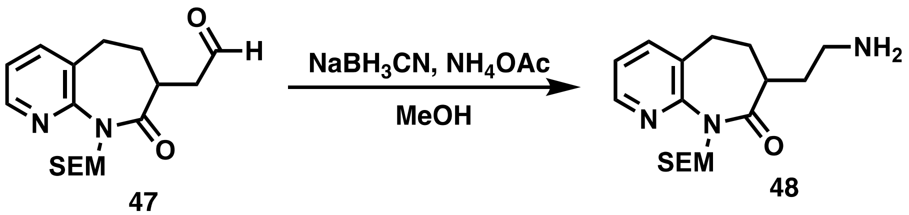

47 **48**

Synthetic procedure 35. To a solution of **47** (800 mg, 2.39 mmol, 1.0 equiv.) in MeOH (160 mL, 0.015M) was added NH_4OAc (92.1 g, 1.20 mol, 0.5 equiv.). The reaction mixture was stirred at 15 °C for 30 minutes. Then sodium cyanoborohydride (450 mg, 7.18 mmol, 3 equiv.) was added. The reaction mixture was stirred at 15 °C for 30 minutes. Upon completion, the reaction was diluted with DCM and concentrated in vacuo to provide a crude brown residue. The residue was diluted with saturated Na_2CO_3 (200 mL) and extracted with EtOAc (2 X 250 mL). The combined organic layers were dried over Na_2SO_4, filtered, and concentrated in vacuo to afford compound **48** (1.10 g, 72% purity) as deep gray oil.

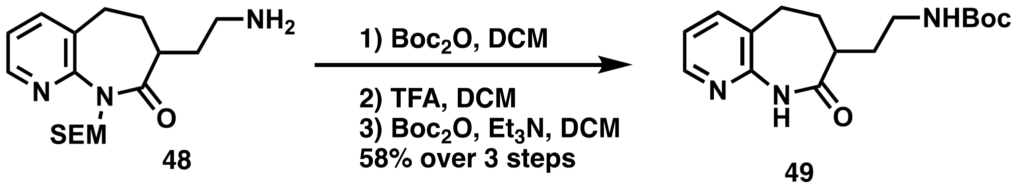

48 **49**

Synthetic procedure 36. To a solution of **48** (1.00 g, 2.98 mmol 1.0 equiv.) in DCM (100 mL, 0.03M) was added Boc_2O (3.25 g, 14.9 mmol, 5 equiv.). The reaction was stirred at 15 °C for 2 hours before another batch of Boc_2O (3.25 g, 14.9 mmol, 5 equiv.) was added and stirred at 15 °C for another 12 hours. Upon completion, the reaction mixture was concentrated in vacuo to provide a crude deep purple residue. This was purified by column chromatography (petroleum ether:EtOAc = 1:1) to afford the Boc-protected primary amine intermediate as colorless oil. This was dissolved in DCM (10 mL) and TFA added (8 mL, 108 mmol, 36 equiv.) before stirring at 25 ºC for 2 hours. The mixture was concentrated in vacuo to provide a brown residue which was dissolved in MeOH before adding K_2CO_3 and stirring at 15 ºC for 10 min. After filtering and concentrating the filtrate in vacuo, a yellow oil (400 mg) was produced. This intermediate was dissolved in DCM (20 mL) before adding Et_3N (1.02 mL, 7.31 mmol) and Boc_2O (956 mg, 4.38 mmol). The reaction was stirred at 15 ºC for 2 hours. Upon completion, the reaction was concentrated in vacuo to produce a crude yellow residue. This was purified by column chromatography (petroleum ether:EtOAc = 1:2) to afford compound **49** in 58% yield (330 mg) as on off-white solid.

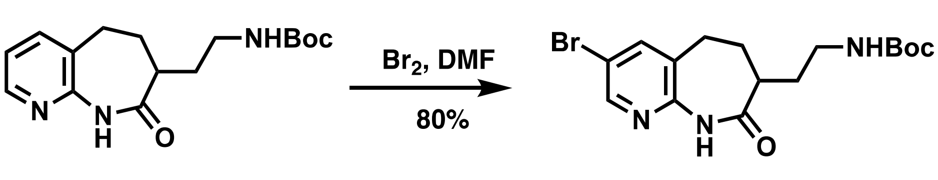

49 **50**

Synthetic procedure 37. To a solution of mixture of **49** (300 mg, 0.982 mmol, 1.0 equiv.) in DMF (20 mL, 0.05M) was added Br_2 (313 mg, 1.96 mmol, 2 equiv.). The reaction was stirred at 15 °C for 2 hours. Upon completion, the reaction mixture was diluted with EtOAc (250 mL) and washed with a saturated solution of brine (3 X 100 mL). The organic layer was dried over Na_2SO_4, filtered, and concentrated in vacuo to provide a crude yellow residue which was purified by column chromatography (petroleum ether:EtOAc = 2:1) to afford compound **50** in 80% yield (335 mg) as a yellow solid.

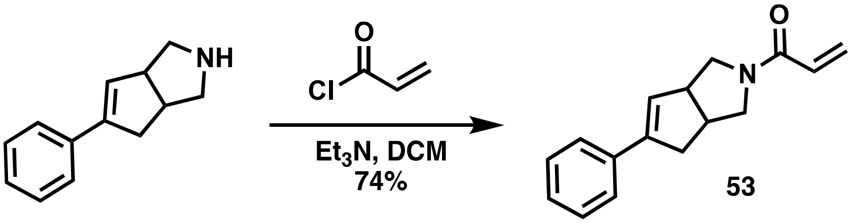

Synthetic procedure 38. To a solution of 5-phenyl-1,2,3,3a,6,6a-hexahydrocyclopenta[*c*]pyrrole (500 mg, 2.7 mmol, 1.0 equiv.) and triethylamine (546 mg, 5.4 mmol, 2.0 equiv.) in DCM (10 mL) was added prop-2-enoyl chloride (293 mg, 3.24 mmol, 1.2 equiv.) at 0 °C. The reaction mixture was stirred at 25 °C for 2 hours. Upon completion, the reaction mixture was washed with brine (5 mL). The organic layer was dried over Na_2SO_4 and concentrated in vacuo. The residue was purified by column chromatography (petroleum ether:EtOAc = 20:1) to afford compound **53** in 74% yield (480 mg) as a white solid.

V. Tabulated Characterization Data

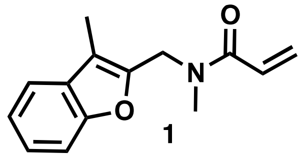

1

Compound **1**, *N*-methyl-*N*-((3- methylbenzofuran-2-yl)methyl)acrylamide, was synthesized according to **synthetic procedure 1**. The reaction was performed on 15.4 mmol scale, providing the desired compound in 79% yield (1.86 g).

Note: Tabulated NMR data for acrylamide derivatives consist of two rotamers that exist at room temperature and is reflected in the reported integral values when applicable and results in the doubling of signals for most ^{13}C nuclei.

^{1}H NMR (500 MHz, Chloroform-*d*): δ 7.51 – 7.45 (m, 1H), 7.43 – 7.36 (m, 1H), 7.32 – 7.18 (m, 2H), 6.85 (dd, *J* = 16.8, 10.6 Hz, 0.35H), 6.59 (dd, *J* = 16.7, 10.4 Hz, 0.65H), 6.42 – 6.33 (m, 1H), 5.80 – 5.67 (m, 1H), 4.77 (s, 1.3H), 4.62 (s, 0.7H), 3.13 (s, 1.95H), 3.02 (s, 1.05H), 2.29 (s, 1.95H), 2.25 (s, 1.05H).

^{13}C NMR (126 MHz, Chloroform-*d*): δ 167.07, 166.29, 154.26, 154.23, 148.93, 147.52, 129.84, 129.48, 128.48, 128.22, 128.04, 127.54, 124.75, 124.30, 122.59, 122.37, 119.49, 113.74, 113.36, 111.20, 111.07, 45.21, 42.26, 35.36, 33.64, 7.95.

Experimental information for the above compound has been previously reported.[3, 14]

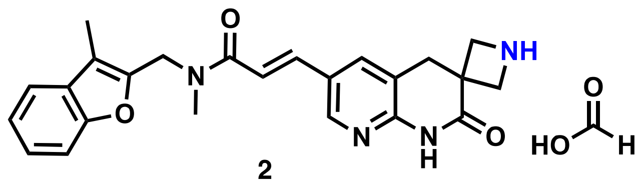

2

Compound **2**, ((*E*)-N-methyl-N-[(3-methylbenzofuran-2-yl)methyl]-3-(7-oxospiro[5,8-dihydro-1,8- naphthyridine-6,3'-azetidine]-3-yl)prop-2-enamid)), was synthesized according to **general procedure 2**. The reaction was performed on a 0.14 mmole scale providing the desired compound in 54% yield (36 mg).

Note: Tabulated NMR data for acrylamide derivatives consist of two rotamers that exist at room temperature and is reflected in the reported integral values when applicable and results in the doubling of signals for most ^{13}C nuclei.

^{1}H NMR (400 MHz, DMSO-*d*6): δ 10.87 (s, 1H), 8.41 - 8.31 (m, 2H, includes formate), 8.15 (s, 1H), 7.57 - 7.22 (m, 6H), 5.00 - 4.79 (m, 2H), 3.98 (d, J = 8.0 Hz, 2H), 3.42 - 3.31 (m, 2H), 3.30 (s, 2H), 3.19 - 2.93 (m, 3H), 2.26 (s, 3H).

^{13}C NMR (101 MHz, DMSO-*d*6): δ 171.13, 165.70, 165.34, 165.28, 153.52, 153.49, 151.57, 149.28, 148.82, 147.64, 147.55, 138.42, 138.01, 134.92, 129.35, 129.26, 126.06, 125.99, 124.51, 124.30, 122.52, 122.43, 119.62, 119.50, 118.00, 117.85, 117.06, 112.98, 112.83, 110.88, 110.83, 51.06, 44.09, 41.68, 40.77, 34.87, 33.44, 33.21, 7.44, 7.41.

HRMS (ESI) *m/z* calc for $C_{24}H_{25}N_4O_3$ $[M+H]^+$: 417.1927, found: 417.1921.

LCMS (220 nm): *m/z* for $C_{24}H_{25}N_4O_3$ $[M+H]^+$: 417.2, 98.3% pure.

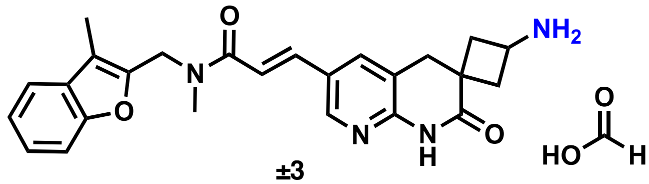

±3

Compound **±3,** ((*E*)-3-(3'-amino-7-oxo-spiro[5,8-dihydro-1,8-naphthyridine-6,1'-cyclobutane]-3-yl)- N-methyl-N-[(3-methylbenzofuran-2-yl)methyl]prop-2-enamide, was synthesized according to **general procedure 2**. The reaction was performed on a 0.08 mmole scale providing the desired compound in 49% yield (18.6 mg).

Note: Tabulated NMR data for acrylamide derivatives consist of two rotamers that exist at room temperature and is reflected in the reported integral values when applicable and results in the doubling of signals for most ^{13}C nuclei.

^{1}H NMR (400 MHz, DMSO-d_6/D_2O): δ 8.40 (s, 1H, formate), 8.39 - 8.36 (br s, 1H), 8.10 (d, J = 9.2 Hz, 1H), 7.60 - 7.23 (m, 6H), 4.97 - 4.76 (m, 2H), 3.74 - 3.70 (m, 1H), 3.21 - 3.10 (m, 3H), 3.09 - 2.90 (m, 2H), 2.51 - 2.50 (m, 1H), 2.38 - 2.34 (m, 1H), 2.25 (s, 3H), 2.24 - 2.02 (m, 1H), 2.01 - 1.99 (m, 1H).

HRMS (ESI): *m/z* calc for $C_{25}H_{27}N_4O_3$ $[M+H]^+$: 431.2083, found: 431.2075.

LCMS (254 nm): *m/z* for $C_{25}H_{27}N_4O_3$ $[M+H]^+$: 431.2, ≥99% pure.

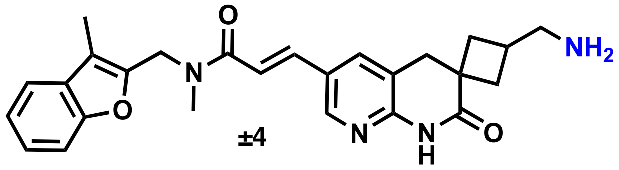

Compound ±**4**, (*E*)-3-[3'-(aminomethyl)-7-oxo-spiro[5,8-dihydro-1,8-naphthyridine-6,1'-cyclobutane] -3-yl]-N-methyl-N-[(3-methylbenzofuran-2-yl)methyl]prop-2-enamide, was synthesized according to **general procedure 2.** The reaction was performed on 157 µmol scale providing the desired compound 21% yield (17 mg).

Note: Tabulated NMR data for acrylamide derivatives consist of two rotamers that exist at room temperature and is reflected in the reported integral values when applicable and results in the doubling of signals for most ^{13}C nuclei.

^{1}H NMR (400 MHz, DMSO-d_6/D_2O): δ 8.41 (s, 1H, formate), 8.40 - 8.38 (m, 1H), 8.10 - 8.03 (m, 1H), 7.60 - 7.22 (m, 6H), 4.97 - 4.76 (m, 2H), 3.16 - 3.07 (m, 3H), 3.00 - 2.92 (m, 2H), 2.83 - 2.80 (m, 2H), 2.56 - 2.50 (m, 1H), 2.33 - 2.26 (m, 1H), 2.24 (s, 3H), 2.13 - 1.71 (m, 3H).

HRMS (ESI): *m/z* calc for $C_{26}H_{29}N_4O_3$ $[M+H]^+$: 445.2240, found: 445.2243.

LCMS (254 nm): *m/z* for $C_{26}H_{29}N_4O_3$ $[M+H]^+$: 445.3, ≥99% pure.

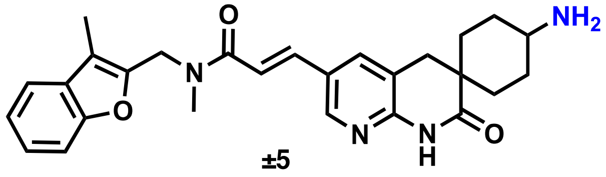

Compound ±**5**, (*E*)-3-(4'-amino-7-oxo-spiro[5,8-dihydro-1,8-naphthyridine-6,1'-cyclohexane]-3-yl)-N- methyl-N-[(3-methylbenzofuran-2-yl)methyl]prop-2-enamide, was synthesized according to **general procedure 2**. The reaction was performed on a 379 µmole scale providing the desired compound in 25% yield (43.0 mg).

Note: Tabulated NMR data for acrylamide derivatives consist of two rotamers that exist at room temperature and is reflected in the reported integral values when applicable and results in the doubling of signals for most ^{13}C nuclei. Exchangeable protons not observed.

^{1}H NMR (400 MHz, DMSO-d_6, 80 ºC): δ 8.34 (d, J = 2.0 Hz, 1H), 7.97 (s, 1H), 7.59 - 7.42 (m, 3H), 7.32 - 7.15 (m, 3H), 4.85 (br s, 2H), 3.22 (s, 3H), 2.79 (s, 2H), 2.76 - 2.68 (m, 1H), 2.27 (s, 3H), 1.95 - 1.83 (m, 2H), 1.68 - 1.58 (m, 2H), 1.54 - 1.39 (m, 2H), 1.26 - 1.11 (m, 2H).

HRMS (ESI): *m/z* calc for $C_{27}H_{31}N_4O_3$ $[M+H]^+$: 459.2396, found: 459.2394.

LCMS (254 nm): *m/z* for $C_{27}H_{31}N_4O_3$ $[M+H]^+$: 459.2, ≥99% pure.

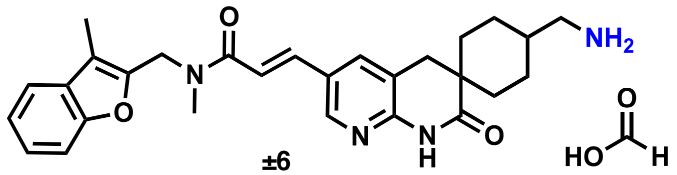
±6

Compound **±6**, (*E*)-3-[4'-(aminomethyl)-7-oxo-spiro[5,8-dihydro-1,8-naphthyridine-6,1'-cyclohexane] -3-yl]-N-methyl-N-[(3-methylbenzofuran-2-yl)methyl]prop-2-enamide, was synthesized according to **general procedure 2.** The reaction was performed on a 208 µmole scale to provide the desired compound in 59% yield (64.0 mg).

Note: Tabulated NMR data for acrylamide derivatives consist of two rotamers that exist at room temperature and is reflected in the reported integral values when applicable and results in the doubling of signals for most ^{13}C nuclei.

^{1}H NMR (400 MHz, DMSO-*d*$_6$/D$_2$O): δ 8.41 (s, 1H, formate), 8.38 - 8.33 (m, 1H), 8.01 (s, 1 H), 7.61 - 7.20 (m, 6 H), 4.97 - 4.76 (m, 2H), 3.17 - 2.91 (m, 3H), 2.78 (s, 2H), 2.68 - 2.62 (m, 2H), 2.24 (s, 3H), 1.80 - 1.76 (m, 2H), 1.63 - 1.56 (m, 3H), 1.43 - 1.37 (m, 2H), 1.18 - 1.13 (m, 2H).

^{13}C NMR (101 MHz, DMSO-*d*$_6$): δ 175.17, 165.84, 165.75, 165.39, 153.52, 153.48, 151.73, 149.31, 148.82, 147.38, 147.25, 138.70, 138.29, 134.24, 129.36, 129.29, 125.62, 125.54, 124.54, 124.33, 122.55, 122.46, 119.65, 119.53, 118.11, 117.49, 117.31, 113.01, 112.85, 110.91, 110.85, 44.08, 43.98, 41.68, 37.73, 35.21, 34.86, 33.43, 31.66, 25.59, 7.48, 7.42.

HRMS (ESI): *m/z* calc for $C_{28}H_{33}N_4O_3$ [M+H]$^+$: 473.2553, found: 473.2548.

LCMS (254 nm): *m/z* for $C_{28}H_{33}N_4O_3$ [M+H]$^+$: 473.3, 98.3% pure.

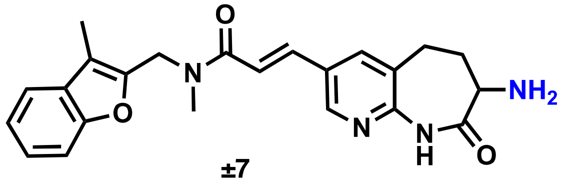
±7

Compound ±**7**, (*E*)-3- (7-amino-8-oxo-6,7,8,9-tetrahydro-5H-pyrido[2,3-b]azepin-3-yl) -N-methyl-N-((3-methylbenzofuran-2-yl)methyl)acrylamide, was synthesized according to **general procedure 2**. The reaction was performed on a 396 µmole scale to provide the desired product in 42% yield (68.7 mg).

Note: Tabulated NMR data for acrylamide derivatives consist of two rotamers that exist at room temperature and is reflected in the reported integral values when applicable and results in the doubling of signals for most ^{13}C nuclei.

^{1}H NMR (400 MHz, DMSO-*d*$_6$): δ 10.30 (br s, 1H), 8.50 (d, *J* = 10.4 Hz, 1H), 8.20 (d, *J* = 6.0 Hz, 1H), 7.60 - 7.50 (m, 2H), 7.50 - 7.20 (m, 4H), 5.10 - 4.80 (m, 2H), 3.20 - 2.90 (m, 4H), 2.70 - 2.69 (m, 1H), 2.30- 2.20 (m, 4H), 1.9 - 1.70 (m, 2H).

^{13}C NMR (101 MHz, DMSO): δ 176.28, 165.61, 165.23, 153.50, 153.46, 152.76, 149.23, 148.74, 147.22, 147.12, 138.23, 137.82, 136.03, 129.34, 129.25, 128.27, 127.40, 127.31, 124.50, 124.30, 122.50, 122.42, 119.61, 119.49, 118.93, 118.76, 113.00, 112.85, 110.87, 110.82, 51.89, 44.08, 41.68, 38.09, 34.86, 33.38, 27.95, 27.89, 7.44, 7.37.

HRMS (ESI): *m/z* calc for $C_{23}H_{25}N_4O_3$ [M+H]$^+$: 405.1927, found: 405.1923.

LCMS (254 nm): *m/z* for $C_{23}H_{25}N_4O_3$ [M+H]$^+$: 405.1, ≥99% pure.

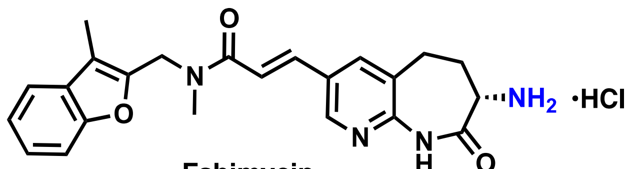

Fabimycin

Compound **(*S*)-7**, **fabimycin**, (*S*,*E*)-3-(7-amino-8-oxo-6,7,8,9-tetrahydro-5*H*-pyrido[2,3-*b*]azepin-3-yl)-N-methyl-N-((3-methylbenzofuran-2-yl)methyl)acrylamide hydrochloride, was synthesized according to **synthetic procedure 11**. The reaction was performed on a 2.4 mmole scale to provide the desired product in 95% yield (1.01 g).

^{1}H NMR (600 MHz, DMSO-*d6*, 25 ºC): δ 10.92 (s, 1H), 8.67 – 8.58 (m, 1H), 8.32 (s, 3H), 8.27 – 8.21 (m, 1H), 7.64 – 7.21 (m, 6H), 5.06 – 4.75 (m, 2H), 3.92 – 3.83 (m, 1H), 3.24 – 2.89 (m, 3H), 2.85 – 2.70 (m, 2H), 2.60 – 2.52 (m, 1H), 2.27 (d, *J* = 4.2 Hz, 3H), 2.24 – 2.15 (m, 1H).

^{1}H NMR (600 MHz, DMSO-*d6*, 120 ºC): δ 10.75 – 10.01 (m, 1H), 8.56 (d, *J* = 2.2 Hz, 1H), 8.36 (s, 3H), 8.12 – 8.06 (m, 1H), 7.58 – 7.50 (m, 2H), 7.45 (d, *J* = 8.1 Hz, 1H), 7.36 – 7.26 (m, 2H), 7.24 (t, *J* = 1.1 Hz, 1H), 4.86 (s, 2H), 3.82 (dd, *J* = 11.5, 7.7 Hz, 1H), 3.12 (s, 3H), 2.91 – 2.79 (m, 2H), 2.66 (tt, *J* = 12.2, 7.7 Hz, 1H), 2.30 – 2.24 (m, 4H).

^{13}C NMR (151 MHz, DMSO-*d6*, 120 ºC): δ 168.24, 165.04, 153.13, 150.54, 148.54, 146.43, 136.56, 136.33, 128.94, 127.91, 126.55, 123.61, 121.75, 119.82, 118.73, 112.15, 110.07, 50.03, 42.65, 33.81, 31.68, 26.36, 6.55.

HRMS (ESI): *m/z* for $C_{23}H_{25}N_4O_3$ $[M+H]^+$: 405.1927, found: 405.1918.

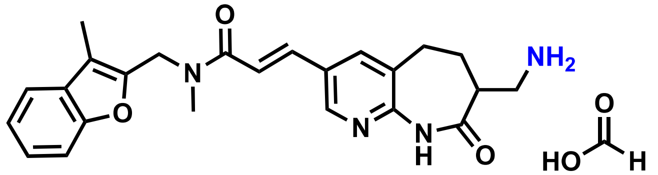

±8

Compound **±8**, (*E*)-3-(7-(aminomethyl)-8-oxo-6,7,8,9-tetrahydro-5H-pyrido [2,3-b] azepin-3-yl)-N-methyl -N-((3-methylbenzofuran-2-yl)methyl)acrylamide, was synthesized according to **general procedure 2**. The reaction was performed on a 134 µmole scale to provide the desired product in 31% yield (19.7 mg).

Note: Tabulated NMR data for acrylamide derivatives consist of two rotamers that exist at room temperature and is reflected in the reported integral values when applicable and results in the doubling of signals for most ^{13}C nuclei.

^{1}H NMR (400 MHz, DMSO-*d*$_6$): δ 8.60 - 8.50 (m, 1H), 8.30 (s, 1H, formate), 8.20 (d, *J* = 5.2 Hz, 1H), 7.60 - 7.20 (m, 6H), 5.10 - 4.70 (m, 2H), 3.20 - 2.90 (m, 4H), 2.80 - 2.50 (m, 4H), 2.30 (s, 3H), 2.27 - 2.20 (m, 1H), 1.94 - 1.90 (m, 1H).

^{13}C NMR (101 MHz, DMSO-*d*$_6$): δ 173.27, 165.58, 165.20, 164.91, 153.47, 152.69, 149.23, 148.72, 147.35, 147.25, 138.12, 137.71, 136.28, 129.33, 129.26, 128.44, 127.75, 127.66, 124.52, 124.32, 122.53, 122.45, 119.63, 119.51, 119.17, 119.01, 113.04, 112.87, 110.85, 44.10, 41.69, 41.21, 34.88, 33.38, 32.49, 28.36, 7.45, 7.38.

HRMS (ESI): *m/z* calc for $C_{24}H_{27}N_4O_3$ $[M+H]^+$: 419.2083, found: 419.2083.

LCMS (ESI): *m/z* for $C_{24}H_{27}N_4O_3$ $[M+H]^+$: 419.3, ≥99% pure.

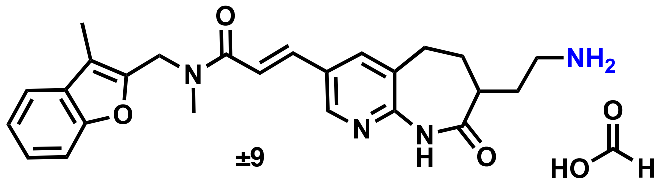

Compound **±9**, (*E*)-3-(7-(2-aminoethyl)-8-oxo-6,7,8,9-tetrahydro-5H-pyrido[2,3-b]azepin-3-yl)-N- methyl-N-((3-methylbenzofuran-2-yl)methyl)acrylamide, was synthesized according to **general procedure 2**. The reaction was performed on a 0.188 mmole scale to provide the desired product in 20% yield (16.0 mg).

Note: Tabulated NMR data for acrylamide derivatives consist of two rotamers that exist at room temperature and is reflected in the reported integral values when applicable and results in the doubling of signals for most ^{13}C nuclei.

^{1}H NMR (400 MHz, DMSO-*d*$_6$): δ 8.59 - 8.50 (m, 1H), 8.39 (br s, 1H, formate), 8.17 (d, *J* = 6.4 Hz, 1H), 7.59 - 7.21 (m, 6H), 5.02 - 4.79 (m, 2H), 3.20 - 2.92 (m, 3H), 2.76 - 2.53 (m, 4H), 2.49 - 2.45 (d, *J* = 7.2 Hz, 1H), 2.26 (s, 4H), 1.99 - 1.81 (m, 2H), 1.56 - 1.37 (m, 1H).

^{13}C NMR (101 MHz, DMSO-*d*$_6$): δ 174.25, 165.61, 165.24, 153.50, 153.46, 153.01, 152.97, 149.24, 148.73, 147.27, 147.17, 138.25, 137.86, 136.14, 129.33, 129.26, 128.47, 127.50, 127.41, 124.51, 124.32, 122.52, 122.45, 119.63, 119.51, 118.89, 118.76, 113.02, 112.85, 110.84, 44.11, 41.69, 38.43, 37.02, 35.24, 34.89, 33.38, 29.34, 28.78, 7.45, 7.38.

HRMS (ESI): *m/z* calc for $C_{25}H_{29}N_4O_3$ [M+H]$^+$: 433.2240, found: 433.2238.

LCMS (254 nm): *m/z* for $C_{25}H_{29}N_4O_3$ [M+H]$^+$: 433.2, 98.4% pure.

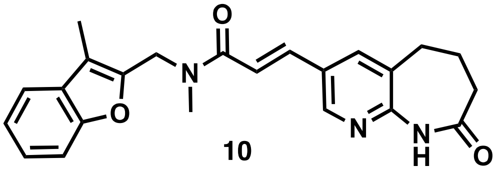

Compound **10**, (*E*)-*N*-methyl-*N*-((3-methylbenzofuran-2-yl)methyl)-3-(8-oxo-6,7,8,9-tetrahydro-5*H*-pyrid0o[2,3-b]azepin-3-yl)acrylamide, was synthesized according to **synthetic procedure 13**. The reaction was performed on a 0.55 mmole scale to produce the desired product in 78% yield (163 mg) over 2 steps.

Note: Tabulated NMR data for acrylamide derivatives consist of two rotamers that exist at room temperature and is reflected in the reported integral values when applicable and results in the doubling of signals for most ^{13}C nuclei.

^{1}H NMR (600 MHz, DMSO-*d*$_6$): δ 10.1 (s, 1H), 8.54-8.50 (m, 1H), 8.18-8.13 (m, 1H), 7.59-7.46 (m, 3.4H), 7.30-7.21 (m, 2.6H), 5.0 (s, 0.8H), 4.80 (s, 1.2H), 3.18 (s, 1.7H), 3.18 (s, 1.8H), 2.92 (s, 1.2H), 2.71 (t, *J* = 7.2 Hz, 2H), 2.28-2.22 (m, 5H), 2.17-2.11 (m, 2H).

^{13}C NMR (151 MHz, DMSO-*d*$_6$): δ 173.24, 165.62, 165.26, 153.53, 153.47, 153.43, 153.38, 149.25, 148.76, 147.23, 147.12, 138.26, 137.86, 136.53, 129.35, 129.27, 127.96, 127.34, 127.25, 124.54, 124.34, 122.54, 122.46, 119.65, 119.53, 118.84, 118.69, 113.04, 112.88, 110.92, 110.86, 44.09, 41.68, 34.88, 33.57, 33.41, 29.39, 29.32, 27.17, 7.48, 7.40.

HRMS (ESI): *m/z* calc for $C_{23}H_{24}N_3O_3$ [M+H]$^+$: 390.1818, found: 390.1799.

LCMS (254 nm): *m/z* for $C_{23}H_{24}N_3O_3$ [M+H]$^+$: 390.2, ≥99% pure.

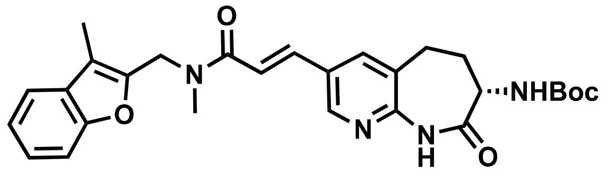

11

Compound **11**, *tert*-butyl (*S*,*E*)-(3-(3-(methyl((3-methylbenzofuran-2-yl)methyl)amino)-3-oxoprop-1-en-1-yl)-8-oxo-6,7,8,9-tetrahydro-5*H*-pyrido[2,3-*b*]azepin-7-yl)carbamate, was synthesized according to **synthetic procedure 10**. The reaction was performed on a 2.95 mmole scale to provide the desired product in 90% yield (1.34 g).

Note: Tabulated NMR data for acrylamide derivatives consist of two rotamers that exist at room temperature and is reflected in the reported integral values when applicable and results in the doubling of signals for most ^{13}C nuclei.

^{1}H NMR (600 MHz, DMSO-*d6*): δ 10.33 (d, *J* = 2.3 Hz, 1H), 8.61 – 8.53 (m, 1H), 8.21 –8.14 (m, 1H), 7.59 – 7.54 (m, 2.4H), 7.51 – 7.47 (m, 1H), 7.32 – 7.22 (m, 2.6H), 7.17 – 7.11 (m, 1H), 5.01 (s, 0.8H), 4.80 (s, 1.2H), 3.92 – 3.83 (m, 1H), 3.19 (s, 1.8H), 2.93 (s, 1.2H), 2.77 – 2.59 (m, 2H), 2.31 – 2.23 (m, 4H), 2.18 – 2.06 (m, 1H), 1.37 – 1.23 (m, 9H).

^{13}C NMR (151 MHz, DMSO-*d6*): δ 172.29, 165.60, 165.22, 155.16, 153.53, 153.48, 152.62, 152.58, 149.23, 148.74, 147.43, 147.33, 138.20, 137.79, 136.39, 136.37, 129.35, 129.27, 127.83, 127.65, 127.56, 124.54, 124.33, 122.53, 122.45, 119.65, 119.52, 119.10, 118.96, 113.05, 112.91, 110.93, 110.86, 78.10, 51.30, 44.07, 41.68, 34.86, 33.65, 33.38, 28.14, 27.81, 27.52, 27.45, 7.48, 7.41.

HRMS (ESI): *m/z* calc for $C_{28}H_{33}N_4O_5$ $[M+H]^+$: 505.2445, found: 505.2437.

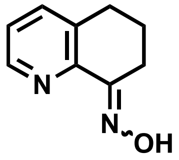

13

Compound **13**, 6,7-dihydroquinolin-8(5H)-one oxime, was synthesized according to **synthetic procedure 2.** The reaction was performed on a 102 mmole scale to provide the desired compound in 92% yield (15.14 g).

^{1}H NMR (500 MHz, $CDCl_3$): δ 8.64 (s, 1H), 8.53 (d, *J* = 4.6 Hz, 1H), 7.47 (d, *J* = 7.7 Hz, 1H), 7.18 (dd, *J* = 7.7, 4.6 Hz, 1H), 2.92 (t, *J* = 6.6 Hz, 2H), 2.81 (t, *J* = 6.1 Hz, 2H), 1.92 (p, *J* = 6.4 Hz, 2H).

^{13}C NMR (126 MHz, $CDCl_3$): δ 153.80, 148.90, 148.51, 136.89, 134.94, 123.82, 29.15, 23.98, 20.94.

HRMS (ESI): *m/z* calc for $C_9H_{11}N_2O$ $[M+H]^+$: 163.0866, found: 163.0877.

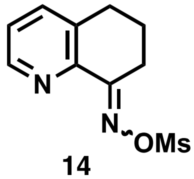

14

Compound **14**, (*E*)-6,7-dihydroquinolin-8(5H)-one O-methylsulfonyl oxime, was synthesized according to **synthetic procedure 3.** The reaction was performed on a 93 mmole scale to provide the desired compound in 97% yield (20.74 g).

^{1}H NMR (500 MHz, DMSO-*d6*): δ 8.56 (dd, *J* = 4.5, 1.4 Hz, 1H), 7.75 (dd, *J* = 8.0, 1.6 Hz, 1H), 7.47 (dd, *J* = 7.8, 4.6 Hz, 1H), 3.34 (s, 3H), 2.93 (t, *J* = 6.6 Hz, 2H), 2.84 (t, *J* = 6.0 Hz, 2H), 1.84 (p, *J* = 6.5 Hz, 2H).

^{13}C NMR (126 MHz, DMSO-*d6*): δ 162.13, 148.18, 145.90, 137.73, 137.34, 125.82, 36.60, 27.77, 25.51, 20.17.

HRMS (ESI): *m/z* calc for $C_{10}H_{13}N_2O_3S$ $[M+H]^+$: 241.0641, found: 241.0648.

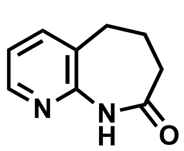

15

Compound **15**, 5,6,7,9-tetrahydro-8H-pyrido[2,3-b]azepin-8-one, was synthesized according to **synthetic procedure 4**. The reaction was performed on a 60 mmole scale to provide the desired compound in 74% yield (7.23 g).

^{1}H NMR (500 MHz, DMSO-*d6*): δ 11.36 (s, 1H), 8.42 (dd, *J* = 4.6, 1.6 Hz, 1H), 7.58 (dd, *J* = 7.7, 1.4 Hz, 1H), 7.25 (dd, *J* = 7.7, 4.6 Hz, 1H), 2.85 – 2.65 (m, 4H), 1.76 (p, *J* = 6.6 Hz, 2H).

^{13}C NMR (126 MHz, DMSO-*d6*): δ 152.39, 149.07, 147.67, 136.46, 134.62, 123.28, 28.44, 24.00, 20.60.

HRMS (ESI): *m/z* calc for $C_9H_{11}N_2O$ $[M+H]^+$: 163.0866, found: 163.0879.

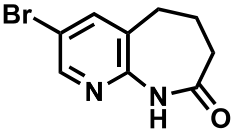

16

Compound **16**, 3-bromo-5,6,7,9-tetrahydro-8*H*-pyrido[2,3-*b*]azepin-8-one, was synthesized according to **synthetic procedure 5**. The reaction was performed on a 44 mmole scale to provide the desired compound in 86% yield (9.14 g).

^{1}H NMR (500 MHz, DMSO-*d6*): δ 10.03 (s, 1H), 8.35 (d, *J* = 2.4 Hz, 1H), 7.97 (d, *J* = 2.4 Hz, 1H), 2.69 (t, *J* = 7.3 Hz, 2H), 2.23 (t, *J* = 7.3 Hz, 2H), 2.12 (p, *J* = 7.3 Hz, 2H).

^{13}C NMR (126 MHz, DMSO-*d6*): δ 173.07, 151.77, 146.77, 140.63, 130.55, 114.59, 33.29, 28.89, 27.17.

HRMS (ESI): *m/z* calc for $C_9H_{10}BrN_2O$ $[M+H]^+$: 240.9971, found: 240.9970.

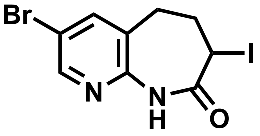

17

Compound **17**, 3-bromo-7-iodo-5,6,7,9-tetrahydro-8*H*-pyrido[2,3-*b*]azepin-8-one, was synthesized according to **synthetic procedure 6**. The reaction was performed on a 16 mmole scale to provide the desired compound in 91% yield (5.30 g).

^{1}H NMR (500 MHz, $CDCl_3$): δ 8.42-8.35 (m, 2H), 7.69 (s, 1H), 4.84 (t, *J* = 6.7 Hz, 1H), 3.06 (dt, *J* = 15.0, 7.8 Hz, 1H), 2.86 (dt, *J* = 15.0, 6.3 Hz, 1H), 2.63 (q, *J* = 6.7 Hz, 2H).

^{13}C NMR (126 MHz, $CDCl_3$): δ 169.35, 149.53, 148.04, 141.46, 127.45, 115.85, 36.50, 31.14, 23.04.

HRMS (ESI): *m/z* calc for $C_9H_9BrIN_2O$ $[M+H]^+$: 366.8937, found: 366.8940.

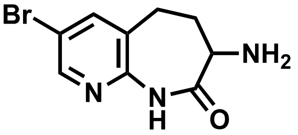

18

Compound **18**, 7-amino-3-bromo-5,6,7,9-tetrahydro-8*H*-pyrido[2,3-*b*]azepin-8-one, was synthesized according to **synthetic procedure 7**. The reaction was performed on a 14.2 mmole scale to provide the desired compound in 83% yield over two steps (3.02 g).

^{1}H NMR (500 MHz, DMSO-*d6*): δ 10.21 (br s, 1H), 8.35 (d, *J* = 2.4 Hz, 1H), 7.97 (d, *J* = 2.4 Hz, 1H), 3.16 (dd, *J* = 11.6, 7.8 Hz, 1H), 2.69 (dd, *J* = 13.9, 6.8 Hz, 1H), 2.56 (td, *J* = 13.2, 8.0 Hz, 1H), 2.31 (m, 1H), 1.79 (m, 1H), 1.69 (br s, 2H).

^{13}C NMR (126 MHz, DMSO-*d6*): δ 176.20, 151.13, 146.83, 140.20, 130.84, 114.69, 51.66, 38.09, 27.54.

HRMS (ESI): m/z calc for $C_9H_{11}BrN_3O$ $[M+H]^+$: 256.0080, found: 256.0078.

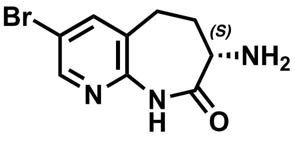

19

Compound **19**, (*S*)-7-amino-3-bromo-5,6,7,9-tetrahydro-8*H*-pyrido[2,3-*b*]azepin-8-one, was synthesized according to **synthetic procedure 8**. The reaction was performed on a 4 mmole scale to provide the desired compound in 76% yield (777 mg).

Note: NMR spectra of free base with (*S)*-Binol additive shown. Binol peaks not annotated in ^{1}H tabulation but are included in ^{13}C tabulation.

^{1}H NMR (500 MHz, $CDCl_3$): δ 8.36 (br s, 1H), 8.33 (s, 1H), 7.68 (s, 1H), 3.17 (dd, *J* = 11.4, 7.6 Hz, 1H), 2.80 (td, *J* = 13.5, 8.1 Hz, 1H), 2.63 (dd, *J* = 14.2, 7.3 Hz, 1H), 2.47 (m, 1H), 1.91 (m, 1H).

^{13}C NMR (126 MHz, $CDCl_3$): δ 176.33, 153.22, 149.85, 147.87, 140.91, 133.74, 131.27, 129.98, 129.40, 128.43, 127.41, 124.42, 123.97, 118.28, 116.15, 111.74, 52.34, 37.88, 28.43.

HRMS (ESI): *m/z* calc for $C_9H_{11}BrN_3O$ $[M+H]^+$: 256.0085, found: 256.0080.

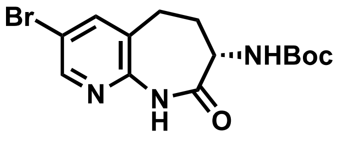

20

Compound **20**, *tert*-butyl (*S*)-(3-bromo-8-oxo-6,7,8,9-tetrahydro-5*H*-pyrido[2,3-*b*]azepin-7-yl)carbamate, was synthesized according to **synthetic procedure 9**. The reaction was performed on a 3 mmole scale to provide the desired product in 99% yield (1.06 g).

^{1}H NMR (500 MHz, $CDCl_3$): δ 8.36 (s, 1H), 8.25 (s, 1H), 7.71 (s, 1H), 5.53 (d, *J* = 7.3 Hz, 1H), 4.31-4.22 (m, 1H), 2.95-2.84 (m, 1H), 2.80-2.65 (m, 2H), 2.09-1.98 (m, 1H), 1.41 (s, 9H).

^{13}C NMR (126 MHz, $CDCl_3$): δ 172.15, 155.07, 149.31, 148.09, 141.20, 129.65, 116.69, 80.16, 51.23, 35.91, 28.43, 27.98.

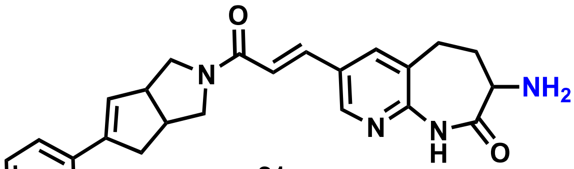

21

Compound **21**, (*E*)-7-amino-3-(3-oxo-3-(5-phenyl-3,3a,6,6a-tetrahydrocyclopenta[c]pyrrol-2(1H)-yl)prop-1- en-1-yl)-6,7-dihydro-5H-pyrido[2,3-b]azepin-8(9H)-one, was synthesized according to **general procedure 2**. The reaction was performed on a 194 µmole scale to provide the desired product in 81% yield (73.0 mg).

Note: Tabulated NMR data for acrylamide derivatives consist of two rotamers that exist at room temperature and is reflected in the reported integral values when applicable and results in the doubling of signals for most ^{13}C nuclei.

^{1}H NMR (400 MHz, DMSO-*d*$_6$/D_2O): δ 8.52 - 8.46 (m, 1H), 8.31 (s, 1H, FA), 8.12 (d, *J* = 11.6 Hz, 1H), 7.48 - 7.44 (m, 2H), 7.43 - 7.38 (m, 1H), 7.33 -7.30 (m, 2H), 7.27 - 7.20 (m, 1H), 7.07 - 7.00 (m, 1H), 6.16 (s, 1H), 4.04 - 3.91 (m, 1H), 3.81 - 3.41 (m, 5H), 3.20 - 3.10 (m, 1 H), 3.04 - 2.84 (m, 2H), 2.81 - 2.59 (m, 3H), 2.12 - 1.98 (m, 1H).

^{13}C NMR (101 MHz, DMSO-d_6): δ 172.39, 164.18, 163.19, 163.16, 151.96, 151.93, 147.42, 141.67, 141.23, 136.53, 136.41, 135.47, 135.44, 128.45, 128.34, 127.87, 127.84, 127.57, 127.54, 125.82, 125.77, 120.85, 120.78, 52.82, 52.61, 50.91, 50.69, 50.33, 50.26, 48.59, 38.44, 38.35, 35.07, 27.26.

HRMS (ESI): *m/z* calc for $C_{25}H_{27}N_4O_2$ $[M+H]^+$: 415.2134, found: 415.2122.

LCMS (254 nm): *m/z* for $C_{25}H_{27}N_4O_2$ $[M+H]^+$: 415.3, ≥99% pure.

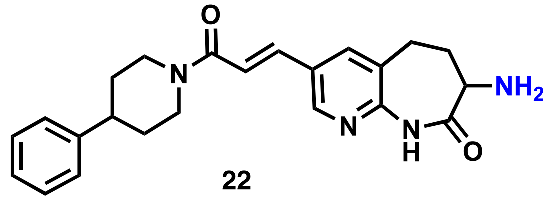

22

Compound **22**, (*E*)-7-Amino-3-(3-oxo-3-(4-phenylpiperidin-1-yl)prop-1-en-1-yl)-6,7-dihydro-5H-pyrido [2,3-b]azepin-8(9H)-one, was synthesized according to **general procedure 2**. The reaction was performed on a 305 μmole scale to provide the desired product in 51% yield (61.0 mg).

Note: Tabulated NMR data for acrylamide derivatives consist of two rotamers that exist at room temperature and is reflected in the reported integral values when applicable and results in the doubling of signals for most ^{13}C nuclei.

^{1}H NMR (400 MHz, DMSO-d_6): δ 10.22 (br s, 1H), 8.50 (d, *J* = 2.0 Hz, 1H), 8.17 (d, *J* = 2.0 Hz, 1H), 7.50 - 7.40 (m, 1H), 7.40 - 7.30 (m, 1H), 7.31 - 7.20 (m, 4H), 7.20 - 7.10 (m, 1H), 4.65 (d, *J* = 12.8 Hz, 1H), 4.45 (d, *J* = 12.8 Hz, 1H), 3.19 - 3.14 (m, 2H), 2.91 - 2.80 (m, 1H), 2.80 - 2.51 (m, 4H), 2.40 - 2.30 (m, 1H), 2.01 - 1.80 (m, 4H), 1.70 - 1.40 (m, 2H).

^{13}C NMR (101 MHz, DMSO-d_6): δ 176.25, 164.05, 152.59, 147.11, 145.60, 137.58, 135.89, 128.41, 128.27, 127.51, 126.72, 126.18, 119.08, 51.87, 45.61, 42.30, 41.91, 38.09, 33.85, 32.81, 27.88.

HRMS (ESI): *m/z* calc for $C_{23}H_{27}N_4O_2$ $[M+H]^+$: 391.2134, found: 391.2127.

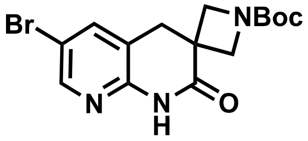

23

Compound **23,** *tert*-butyl 6-bromo-2-oxo-spiro[1,4-dihydro-1,8-naphthyridine-3,3'-azetidine] -1'-carboxylate, was synthesized according to **synthetic procedure 14**. The reaction was performed on a 4.6 mmole scale to provide the desired compound in 30% yield (460 mg).

LCMS (254 nm): *m/z* for $C_{15}H_{19}BrN_3O_3$ $[M+H]^+$: 368.0, 370.0, 86% pure.

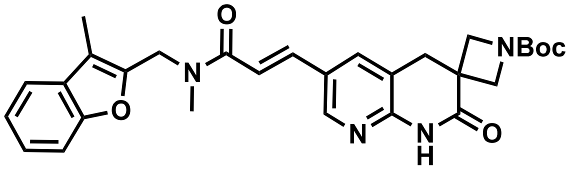

24

Compound **24,** 6-[(*E*)-3-[methyl-[(3-methylbenzofuran-2-yl)methyl]amino]-3-oxo-prop-1-enyl]-2-oxo-spiro [1,4-dihydro-1,8-naphthyridine-3,3'-azetidine]-1'-carboxylate, was synthesized according to **general procedure 1**. The reaction was performed on a 706 μmole scale to provide the desired compound in 40% yield (160 mg).

LCMS (254 nm): *m/z* for $C_{29}H_{33}N_4O_5$ $[M+H]^+$: 517.3, 92.6% pure.

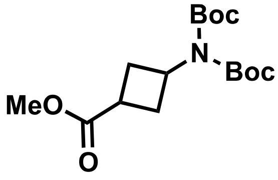

25

Compound **25,** methyl 3-[bis(*tert*-butoxycarbonyl)amino]cyclobutanecarboxylate was synthesized according to **synthetic procedure 15**. The reaction was performed on a 6.89 mmole scale to provide the desired compound in 60% yield (1.38 g).

^{1}H NMR (400 MHz, $CDCl_3$): δ 4.73 – 4.20 (m, 1H), 3.72 – 3.68 (m, 3H), 2.76 – 2.47 (m, 5H), 1.50 – 1.48 (m, 18H).

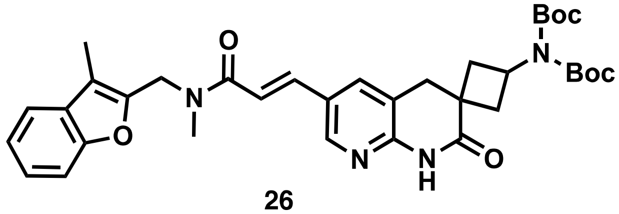

26

Compound **26,** *tert*-butyl N-tert-butoxycarbonyl-N-[6-[(*E*)-3-[methyl-[(3-methylbenzofuran-2-yl)methyl] amino]-3-oxo-prop-1-enyl]-2-oxo-spiro[1,4-dihydro-1,8-naphthyridine-3,3'-cyclobutane]-1'-yl]carbamate, was synthesized according to **synthetic procedure 16**. The reaction was performed on a 4 mmole scale to provide the desired compound in 2.2% yield over 2 steps (30 mg).

LCMS (254 nm): *m/z* for $C_{35}H_{43}N_4O_7$ $[M+H]^+$: 631.2, 72% pure.

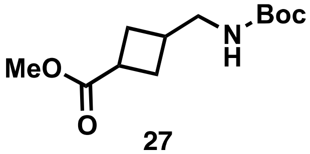

27

Compound **27,** methyl 3-(((*tert*-butoxycarbonyl)amino)methyl)cyclobutanecarboxylate, was synthesized according to **synthetic procedure 17**. The reaction was performed on a 11 mmole scale to provide the desired compound in 94% (2.60 g).

^{1}H NMR (400 MHz, $CDCl_3$): δ 4.60 - 4.51 (m, 1H), 3.70 - 3.67 (m, 3H), 3.25 - 3.00 (m, 3H), 2.50 - 2.25 (m, 3H), 2.02 - 1.95 (m, 2H), 1.45 (s, 9H).

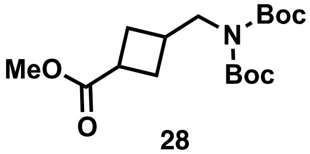

28

Compound **28**, methyl 3-[[bis(*tert*-butoxycarbonyl)amino]methyl]cyclobutanecarboxylate, was synthesized according to **synthetic procedure 18**. The reaction was performed on a 10.7 mmole scale to provide the desired compound in 82% yield (3.0 g).

^{1}H NMR (400 MHz, $CDCl_3$): δ 3.77 - 3.59 (m, 5H), 3.15 - 2.90 (m, 1H), 2.68 - 2.50 (m, 1H), 2.35 - 2.31 (m, 2H), 2.08 - 2.01 (m, 2H), 1.51 (s, 18H).

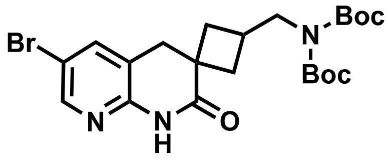

29

Compound **29**, *tert*-butyl N-[(6-bromo-2-oxo-spiro[1,4-dihydro-1,8-naphthyridine-3,3'-cyclobutane]-1'-yl) methyl]-N-*tert*-butoxycarbonyl-carbamate, was synthesized according to **synthetic procedure 19**. The reaction was performed on a 4.3 mmole scale to provide the desired compound in 30% yield (430 mg).

LCMS (254 nm) *m/z* for $C_{22}H_{31}BrN_3O_5$ $[M+H]^+$: 496.2, 498.2; 75% pure.

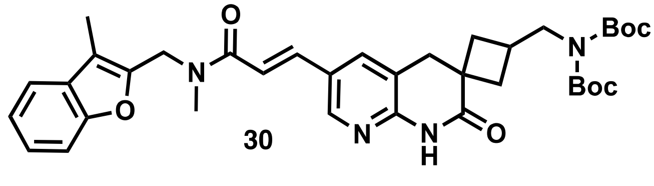

30

Compound **30**, *tert*-butyl N-tert-butoxycarbonyl-N-[[6-[(*E*)-3-[methyl-[(3-methylbenzofuran-2-yl)methyl] amino]-3-oxo-prop-1-enyl]-2-oxo-spiro[1,4-dihydro-1,8-naphthyridine-3,3'-cyclobutane]-1'-yl]methyl]carbamate, was synthesized according

to **general procedure 1**. The reaction was performed on a 302 µmole scale providing the desired compound in 52% yield (167 mg).

LCMS (254 nm) *m/z* for $C_{36}H_{45}N_4O_7$ $[M+H]^+$: 645.5, 62% pure.

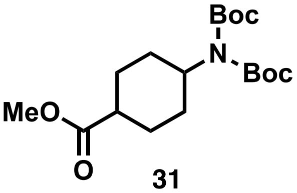

31

Compound **31,** methyl 4-[bis(*tert*-butoxycarbonyl)amino]cyclohexanecarboxylate, was synthesized according to **synthetic procedure 20**. The reaction was performed on a 7 mmole scale to provide the desired compound in 52% yield (1.30 g).

^{1}H NMR (300 MHz, $CDCl_3$): δ 4.01 - 3.90 (m, 1H), 3.70 (s, 3H), 2.65 - 2.57 (m, 1H), 2.31 - 2.22 (m, 2H), 2.05 - 1.89 (m, 2H), 1.71 - 1.62 (m, 2H), 1.56 - 1.51 (m, 2H), 1.49 (s, 18H).

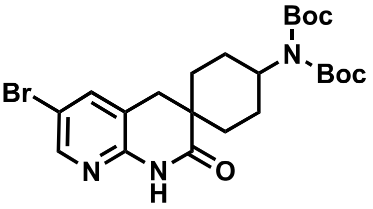

32

Compound **32**, *tert*-butyl N-(6-bromo-2-oxo-spiro[1,4-dihydro-1,8-naphthyridine-3,4'-cyclohexane]-1'-yl) -N-*tert*-butoxycarbonyl-carbamate, was synthesized according to **synthetic procedure 21**. The reaction was performed on a 4.3 mmole scale to provide the desired product in 41% yield (450 mg).

^{1}H NMR (400 MHz, $CDCl_3$): δ 8.50 (br s, 1H), 8.24 (d, J = 1.6 Hz, 1H), 7.59 (d, J = 1.6 Hz, 1H), 4.04 - 3.87 (m, 1H), 2.70 (s, 2H), 2.48 - 2.41 (m, 2H), 2.11 - 1.93 (m, 2H), 1.73 - 1.64 (m, 2H), 1.52 (s, 18H), 1.40 - 1.27 (m, 2H).

LCMS (254 nm): *m/z* for $C_{23}H_{33}BrN_3O_5$ $[M+H]^+$: 510.0, 512.0; ≥99% pure.

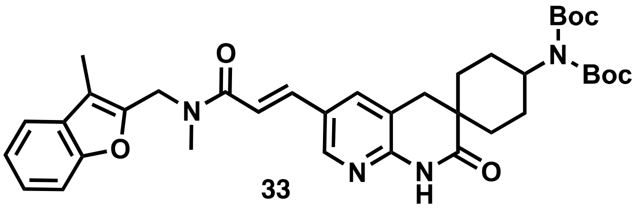

33

Compound **33**, *tert*-butyl N-tert-butoxycarbonyl-N-[6-[(*E*)-3-[methyl-[(3-methylbenzofuran-2-yl) methyl]amino]-3-oxo-prop-1-enyl]-2-oxo-spiro[1,4-dihydro-1,8-naphthyridine-3,4'-cyclohexane]-1'-yl]carbamate, was synthesized according to **general procedure 1**. The reaction was performed on a 979 µmole scale to provide the desired product in 60% yield (450 mg).

Note: Tabulated NMR data for acrylamide derivatives consist of two rotamers that exist at room temperature and is reflected in the reported integral values when applicable and results in the doubling of signals for most ^{13}C nuclei.

^{1}H NMR (400 MHz, $CDCl_3$): δ 8.30 (s, 1H), 8.18 (s, 1H), 7.75 - 7.59 (m, 2H), 7.56 - 7.46 (m, 1H), 7.45 - 7.39 (m, 1H), 7.36 - 7.27 (m, 1H), 7.26 - 7.18 (m, 1H), 7.17 - 6.86 (m, 1H), 4.97 - 4.66 (m, 2H), 4.05 - 3.90 (m, 1H), 3.34 - 3.03 (m, 3H), 2.80 - 2.65 (m, 2H), 2.52 - 2.37 (m, 2H), 2.32 (s, 3H), 2.07 - 1.99 (m, 2H), 1.73 - 1.65 (m, 2H), 1.51 (s, 18H), 1.40 - 1.29 (m, 2H).

LCMS (254 nm): *m/z* for $C_{37}H_{47}N_4O_7$ $[M+H]^+$: 659.3, 90% pure

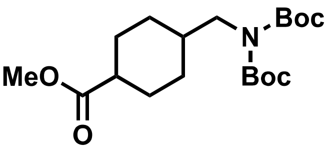

34

Compound **34**, methyl 4-(((di-*tert*-butoxycarbonyl)amino)methyl)cyclohexanecarboxylate, was synthesized according to **synthetic procedure 22**. The reaction was performed on a 17.3 mmole scale to provide the desired product in 56% yield (3.66 g).

[1]**H NMR** (400 MHz, $CDCl_3$): δ 3.66 (s, 3H), 3.43 (d, J = 7.2 Hz, 2H), 2.25 - 2.20 (m, 1H), 2.01 - 1.96(m, 2H), 1.81 - 1.74 (m, 2H), 1.65 - 1.60 (m, 1H), 1.51 - 1.40 (s, 18H), 1.39 - 1.34 (m, 2H), 1.02 - 0.97 (m, 2H).

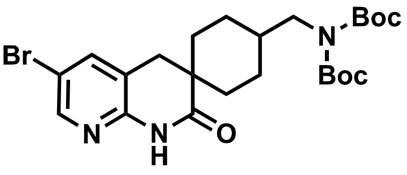

35

Compound **35**, *tert*-butyl N-[(6-bromo-2-oxo-spiro [1,4-dihydro-1,8-naphthyridine-3,4'-cyclohexane]-1'-yl) methyl]-N-*tert*-butoxycarbonyl-carbamate, was synthesized according to **synthetic procedure 23**. The reaction was performed on a 1.7 mmole scale to provide the desired product in 45% yield (208 mg).

[1]**H NMR** (400 MHz, DMSO-d_6): δ 10.51 (s, 1H), 8.20 (s, 1H), 7.81 (s, 1H), 3.40 - 3.37 (m, 2H), 2.82 (s, 2H), 1.79 - 1.74 (m, 2H), 1.61 - 1.59 (m, 2H), 1.40 (s, 18H), 1.18 - 1.16 (m, 1H), 1.16 - 1.13 (m, 4H).

LCMS (254 nm): *m/z* for $C_{24}H_{35}BrN_3O_5$ $[M+H]^+$: 524.3, 526.3, 94% pure.

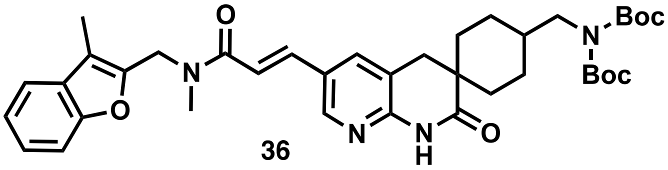

Compound **36**, *tert*-butyl N-*tert*-butoxycarbonyl-N-[[6-[(*E*)-3-[methyl-[(3-methylbenzofuran-2-yl) methyl]amino]-3-oxo-prop-1-enyl]-2-oxo-spiro[1,4-dihydro-1,8-naphthyridine-3,4'-cyclohexane]-1'-yl]methyl]carbamate, was synthesized according to **general procedure 1**. The reaction was performed on a 324 µmole scale to provide the desired product in 66% yield (145 mg).

LCMS (254 nm): *m/z* for $C_{38}H_{49}N_4O_7$ $[M+H]^+$: 673.4, 98% pure.

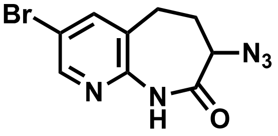

37

Compound **37**, 7-zzido-3-bromo-6,7-dihydro-5H-pyrido[2,3-b]azepin-8(9H)-one, was synthesized according to **synthetic procedure 25**. The reaction was performed on a 2.7 mmole scale to provide the desired compound in 84% yield (650 mg).

[1]**H NMR** (400 MHz, DMSO-d_6): δ 10.59 (s, 1H), 8.39 (d, *J* = 2.4 Hz, 1H), 8.01 (d, *J* = 2.4 Hz, 1H), 4.10 (dd, *J* = 11.2, 8.0 Hz, 1H), 2.80 - 2.70 (m, 1H), 2.70 - 2.60 (m, 1H), 2.50 - 2.41 (m, 1H), 2.17 - 2.09 (m, 1H).

LCMS (254 nm): *m/z* for $C_9H_9BrN_5O$ $[M+H]^+$: 282.0, 284.0; 95.8% pure.

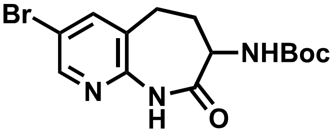

38

Compound **38**, *tert*-butyl (3-bromo-8-oxo-6,7,8,9-tetrahydro-5H-pyrido[2,3-b]azepin-7-yl)carbamate, was synthesized according to **synthetic procedure 27**. The reaction was performed on a 2.71 mmole scale to provide the desired compound in 99% yield (850 mg).

[1]**H NMR** (400 MHz, DMSO-d_6): δ 10.31 (s, 1H), 8.40 (d, *J* = 2.0 Hz, 1H), 8.00 (d, *J* = 2.0 Hz, 1H), 7.11 (d, *J* = 8.4 Hz, 1H), 3.88 - 3.82 (m, 1H), 2.75 - 2.71 (m, 1H), 2.61 - 2.50 (m, 1H), 2.31 - 2.25 (m, 1H), 2.12 - 2.09 (m, 1H), 1.31 (s, 9H).

LCMS (254 nm): *m/z* for $C_{14}H_{19}BrN_3O_3$ $[M+H]^+$: 355.9, 357.9; 87% pure.

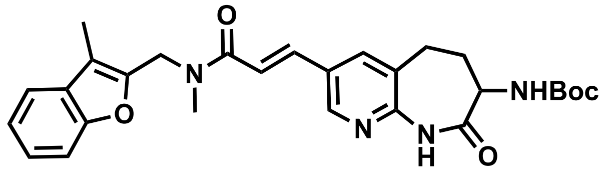

39

Compound **39**, (*E*)-*tert*-butyl (3-(3-(methyl ((3-methylbenzofuran-2-yl) methyl)amino)-3-oxoprop-1-en-1-yl) -8-oxo-6,7,8,9-tetrahydro-5H-pyrido[2,3-b]azepin-7-yl)carbamate, was synthesized according to **general procedure 1**. The reaction was performed on a 1.11 mmole scale to provide the desired compound in 88% yield (485 mg).

Note: Tabulated NMR data for acrylamide derivatives consist of two rotamers that exist at room temperature and is reflected in the reported integral values when applicable and results in the doubling of signals for most ^{13}C nuclei.

^{1}H NMR (400 MHz, $CDCl_3$): δ 8.91 (br s, 1H), 8.61 - 8.41 (m, 1H), 7.80 - 7.70 (m, 2H), 7.50 (d, *J* = 7.2 Hz, 1H), 7.40 (d, *J* = 7.6 Hz, 1H), 7.31 - 7.30 (m, 1H), 7.28 (d, *J* = 7.6 Hz, 1H), 6.90 (d, *J* = 15.6 Hz, 1H), 5.60 (d, *J* = 7.2 Hz, 1H), 4.90 - 4.70 (m, 2H), 4.31 - 4.28 (m, 1H), 3.30 - 3.10 (m, 3H), 2.90 (d, *J* = 8.4 Hz, 1H), 2.80 - 2.70 (m, 2H), 2.30 (s, 3H), 2.10 - 2.09 (m, 1H), 1.4 (s, 9H).

LCMS (254 nm): *m/z* for $C_{28}H_{33}N_4O_5$ $[M+H]^+$: 505.2, 98.3% pure.

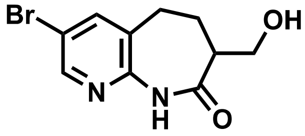

40

Compound **40**, 3-bromo-7-(hydroxymethyl)-6,7-dihydro-5H-pyrido[2,3-b]azepin-8(9H)-one, was synthesized according to **synthetic procedure 28**. The reaction was performed on a 5.20 mmole scale to provide the desired compound in 40% yield (570 mg).

^{1}H NMR (400 MHz, $CDCl_3$): δ 8.37 (d, *J* = 2.0 Hz, 1H), 8.23 (br s, 1H), 8.00 (s, 1H), 7.73 (d, *J* = 2.0 Hz, 1H), 3.80 - 3.75 (m, 2H), 2.87 - 2.77 (m, 1H), 2.75 - 2.71 (m, 1H), 2.63 - 2.58 (m, 1H), 2.33 - 2.29 (m, 1H), 2.20 - 2.17 (m, 1H).

LCMS (254 nm): *m/z* for $C_{10}H_{12}BrN_2O_2$ $[M+H]^+$: 270.9, 272.9; 92.7% pure.

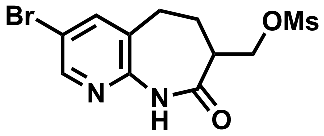

41

Compound **41,** (3-bromo-8-oxo-6,7,8,9-tetrahydro-5H-pyrido[2,3-b]azepin-7-yl)methyl methanesulfonate, was synthesized according to **synthetic procedure 29**. The reaction was performed on a 2.07 mmole scale to provide the desired product in 56% yield (410 mg).

^{1}H NMR (400 MHz, $CDCl_3$): δ 8.40 (d, *J* = 2.0 Hz, 1H), 8.16 (br s, 1H), 7.73 (d, *J* = 2.0 Hz, 1H), 4.60 (dd, *J* = 10.0, 7.2 Hz, 1H), 4.22 (dd, *J* = 10.0, 5.2 Hz, 1H), 3.06 (s, 3H), 2.89 - 2.85 (m, 2H), 2.78 - 2.72 (m, 1H), 2.43 - 2.35 (m, 1H), 2.14 - 2.06 (m, 1H).

LCMS (254 nm): *m/z* for $C_{11}H_{14}BrN_2O_4S$ $[M+H]^+$: 348.9, 350.9; ≥99% pure.

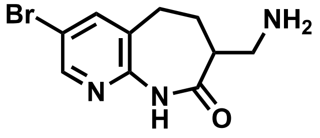

42

Compound **42**, 7-(aminomethyl)-3-bromo-6,7-dihydro-5H-pyrido[2,3-b]azepin-8(9H)-one, was synthesized according to **synthetic procedure 30**. The reaction was performed on a 458 µmole scale to provide the desired product in 90% yield (150 mg).

LCMS (254 nm): *m/z* for $C_{10}H_{13}BrN_3O$ $[M+H]^+$: 269.9, 271.9; 89.6% pure.

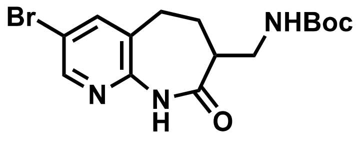

43

Compound **43**, *tert*-butyl ((3-bromo-8-oxo-6,7,8,9-tetrahydro-5H-pyrido [2,3-b]azepin-7-yl)methyl) carbamate, was synthesized according to **synthetic procedure 31**. The reaction was performed on a 333 µmole scale to provide the desired product in 60% yield (120 mg).

^{1}H NMR (400 MHz, $CDCl_3$): δ 8.34 (d, *J* = 2.0 Hz, 1H), 7.85 (br s, 1H), 7.70 (d, *J* = 2.0 Hz, 1H), 5.25 (t, *J* = 5.6 Hz, 1H), 3.31 - 3.23 (m, 2H), 2.89 - 2.72 (m, 1H), 2.72 - 2.67 (m, 1H), 2.65 - 2.55 (m, 1H), 2.30 - 2.21 (m, 1H), 2.13 - 2.00 (m, 1H), 1.41 (s, 9H).

LCMS (254 nm): *m/z* for $C_{15}H_{21}BrN_3O_3$ $[M+H]^+$: 370.0, 372.0; 92.6% pure.

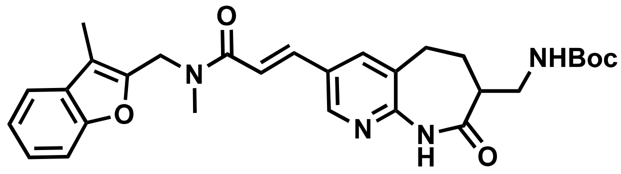

44

Compound **44**, (*E*)-*tert*-butyl ((3-(3-(methyl ((3-methylbenzofuran-2-yl) methyl) amino)-3-oxoprop-1-en-1- yl)-8-oxo-6,7,8,9-tetrahydro-5H-pyrido[2,3-b]azepin-7-yl)methyl)carbamate, was synthesized according to **general procedure 1**. The reaction was performed on a 405 µmole scale to provide the desired compound in 67% yield (142 mg).

Note: Tabulated NMR data for acrylamide derivatives consist of two rotamers that exist at room temperature and is reflected in the reported integral values when applicable and results in the doubling of signals for most ^{13}C nuclei.

^{1}H NMR (400 MHz, $CDCl_3$): δ 8.40 (s, 1H), 7.80 (s, 1H), 7.70 - 7.60 (m, 2H), 7.50 (d, *J* = 7.2 Hz, 1H), 7.40 (d, *J* = 7.6 Hz, 1H), 7.31 - 7.30 (m, 1H), 7.30 - 6.90 (m, 2H), 5.20 (br s, 1H), 4.90 - 4.71 (m, 2H), 3.40 - 3.30 (m, 1H), 3.31 - 3.30 (m, 1H), 3.30 - 3.10 (m, 3H), 2.90 - 2.89 (m, 1H), 2.80 - 2.71 (m, 1H), 2.70 - 2.50 (m, 1H), 2.31 - 2.30 (m, 3H), 2.33 - 2.30 (m, 1H), 2.10 - 2.06 (m, 1H), 1.41 - 1.4 (m, 9H).

LCMS (254 nm): *m/z* for $C_{29}H_{35}N_4O_5$ $[M+H]^+$: 519.3, ≥99% pure.

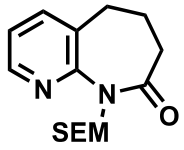

Compound **45**, 9-((2-(trimethylsilyl)ethoxy)methyl)-6,7-dihydro-5H-pyrido[2,3-b]azepin-8(9H)-one, was synthesized according to **synthetic procedure 32**. The reaction was performed on a 73.9 mmole scale to provide the desired product in 53% yield (7.70 g).

45

^{1}H NMR (400 MHz, $CDCl_3$): δ 8.40 (dd, *J* = 4.8, 1.6 Hz, 1H), 7.55 (dd, *J* = 7.2, 1.6 Hz, 1H), 7.11 (dd, *J* = 7.2, 4.8 Hz, 1H), 5.51 (s, 2H), 3.67 - 3.57 (m, 2H), 2.77 (t, *J* = 7.2 Hz, 2H), 2.44 - 2.33 (m, 2H), 2.31 - 2.15 (m, 2H), 0.96 - 0.77 (m, 2H), -0.03 - -0.15 (m, 9H).

LCMS (254 nm): *m/z* for $C_{15}H_{25}N_2O_2Si$ $[M+H]^+$: 293.2, 94.1% pure.

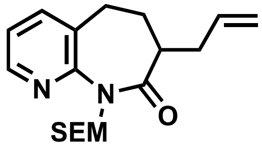

Compound **46**, 7-allyl-9-((2-(trimethylsilyl)ethoxy)methyl)-6,7-dihydro-5H-pyrido[2,3-b]azepin-8(9H)-one, was synthesized according to **synthetic procedure 33**. The reaction was performed on a 14.3 mmole scale to provide the desired product in 85% yield (4.10 g).

46

^{1}H NMR (400 MHz, $CDCl_3$): δ 8.40 (dd, *J* = 4.8, 1.6 Hz, 1H), 7.53 (dd, *J* = 7.6, 1.6 Hz, 1H), 7.11 (dd, *J* = 7.6, 4.8 Hz, 1H), 5.76 - 5.60 (m, 1H), 5.55 - 5.46 (m, 2H), 5.04 - 4.91 (m, 2H), 3.70 - 3.56 (m, 2H), 2.92 -

2.81 (m, 1H), 2.69 - 2.52 (m, 2H), 2.40-2.36 (m, 1H), 2.24 - 2.08 (m, 2H), 2.04 - 1.98 (m, 1H), 0.98 - 0.82 (m, 2H), -0.03 - -0.14 (m, 9H).

LCMS (254 nm): *m/z* for $C_{18}H_{29}N_2O_2Si$ $[M+H]^+$: 333.2, 97.4% pure.

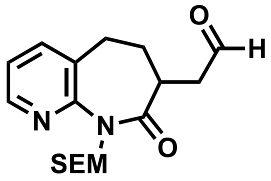

47

Compound **47**, 2-(8-oxo-9-((2-(trimethylsilyl)ethoxy)methyl)-6,7,8,9-tetrahydro-5H-pyrido[2,3-b] azepin-7-yl)acetaldehyde, was synthesized according to **synthetic procedure 34**. The reaction was performed on a 6.92 mmole scale to provide the desired compound in 47% yield (1.10 g).

^{1}H NMR (400 MHz, $CDCl_3$): δ 9.73 (s, 1H), 8.43 (dd, *J* = 4.8, 1.6 Hz, 1H), 7.55 (dd, *J* = 7.2, 1.6 Hz, 1H), 7.13 (dd, *J* = 7.2, 4.8 Hz, 1H), 5.52 (s, 2H), 3.67 - 3.51 (m, 2H), 3.10 (dd, *J* = 17.2, 8.0 Hz, 1H), 2.96 - 2.85 (m, 2H), 2.66 (dd, *J* = 13.6, 6.4 Hz, 1H), 2.50 - 2.45 (m, 1H), 2.30 - 2.18 (m, 1H), 2.11 - 2.06 (m, 1H), 0.91 - 0.85 (m, 2H), -0.04 - -0.08 (m, 9H).

LCMS (254 nm): *m/z* for $C_{17}H_{27}N_2O_3Si$ $[M+H]^+$: 335.1, ≥99% pure.

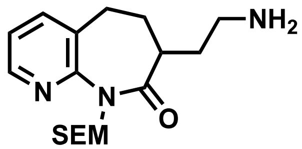

48

Compound **48**, 7-(2-aminoethyl)-9-((2-(trimethylsilyl)ethoxy)methyl)-6,7-dihydro-5H-pyrido[2,3-b] azepin-8(9H)-one, was synthesized according to **synthetic procedure 35**. The reaction was performed on a 3.29 mmole scale to provide 1.10 g of the desired product in 72% purity.

LCMS (254 nm): *m/z* for $C_{17}H_{27}N_3O_2Si$ $[M+H]^+$: 336.3, 72.6% pure.

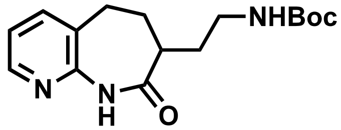

49

Compound **49**, *tert*-butyl (2-(8-oxo-6,7,8,9-tetrahydro-5H-pyrido[2,3-b]azepin-7-yl)ethyl)carbamate, was synthesized according to **synthetic procedure 36**. The reaction was performed on a 2.98 mmole scale to provide the desired compound in 58% yield.

^{1}H NMR (400 MHz, $CDCl_3$): δ 8.36 - 8.28 (m, 1H), 8.10 - 7.80 (m, 1H), 7.61 - 7.53 (m, 1H), 7.13 - 7.04 (m, 1H), 4.57 (br s, 1H), 3.52 - 3.07 (m, 2H), 2.87 - 2.76(m, 1H), 2.65 - 2.66 (m, 1H), 2.53 - 2.07 (m, 4H), 2.04 - 1.97 (m, 1H), 1.38 (s, 9H).

LCMS (254 nm): *m/z* for $C_{16}H_{24}N_3O_3$ $[M+Na]^+$: 328.3, 73.7% pure.

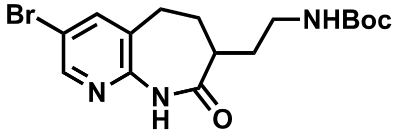

50

Compound **50**, *tert*-butyl (2-(3-bromo-8-oxo-6,7,8,9-tetrahydro-5H-pyrido [2,3-b]azepin-7-yl)ethyl) carbamate, was synthesized according to **synthetic procedure 37**. The reaction was performed on a 0.982 mmole scale to provide the desired product in 80% yield (335 mg).

^{1}H NMR (400 MHz, $CDCl_3$): δ 8.40 - 8.27 (m, 1H), 8.11 - 8.00 (m, 1H), 7.74 - 7.42 (m, 1H), 4.63 (br s, 1H), 3.17 - 3.05 (m, 1H), 2.88 - 2.79 (m, 1H), 2.74 - 2.64 (m, 1H), 2.56 - 2.22 (m, 2H), 2.15 - 1.92 (m, 2H), 1.77 - 1.68 (m, 1H), 1.65 - 1.55 (m, 1H), 1.47 - 1.31 (m, 9H).

LCMS (254 nm): *m/z* for $C_{16}H_{22}BrN_3O_3$ $[M+Na]^+$: 406.1, 408.1; 52.5% pure.

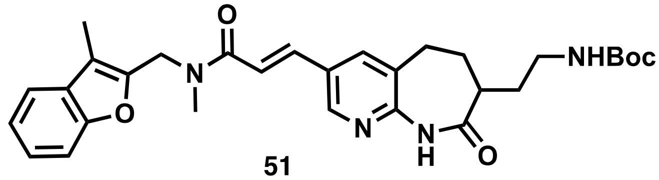

51

Compound **51**, (*E*)-*tert*-butyl (2-(3-(3-(methyl ((3-methylbenzofuran-2-yl)methyl)amino)-3-oxoprop- 1-en-1-yl)-8-oxo-6,7,8,9-tetrahydro-5H-pyrido[2,3-b]azepin-7-yl)ethyl)carbamate, was synthesized according to **general procedure 1**. The reaction was performed on a 530 µmole scale to provide the desired product in 50% yield (110 mg).

Note: Tabulated NMR data for acrylamide derivatives consist of two rotamers that exist at room temperature and is reflected in the reported integral values when applicable and results in the doubling of signals for most ^{13}C nuclei.

^{1}H NMR (400 MHz, $CDCl_3$): δ 8.49 - 8.40 (m, 1H), 7.91 - 7.65 (m, 3H), 7.52 - 7.48 (m, 1H), 7.46 - 7.40 (m, 1H), 7.32 - 7.29 (m, 1H), 7.26 - 6.88 (m, 2H), 4.88 - 4.72 (m, 2H), 3.29 - 3.06 (m, 5H), 2.93 - 2.83 (m, 1H), 2.78 - 2.68 (m, 1H), 2.54 - 2.38 (m, 2H), 2.33 (s, 3H), 2.13 - 1.97 (m, 2H), 1.42 - 1.32 (m, 9H).

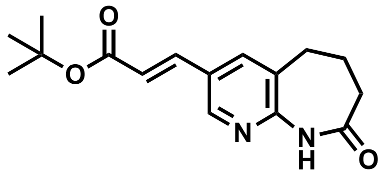

52

Compound **52**, *tert*-butyl (*E*)-3-(8-oxo-6,7,8,9-tetrahydro-5*H*-pyrido[2,3-b]azepin-3-yl)acrylate, was synthesized according to **synthetic procedure 12**. The reaction was performed on a 2.5 mmole scale to provide the desired compound in 83% yield (596 mg).

^{1}H NMR (500 MHz, $CDCl_3$): δ 8.38 (d, *J* = 2.0 Hz, 1H), 8.08 (s, 1H), 7.68 (d, *J* = 2.0 Hz, 1H), 7.53 (d, *J* = 16.0 Hz, 1H), 6.38 (d, *J* = 16.0 Hz, 1H), 2.84 (t, *J* = 7.2 Hz, 2H), 2.51 (t, *J* = 7.2 Hz, 2H), 2.26 (t, *J* = 7.2 Hz, 2H), 1.54 (s, 9H).

^{13}C NMR (126 MHz, $CDCl_3$): δ 173.87, 165.83, 152.63, 147.09, 139.09, 137.01, 127.68, 127.43, 121.55, 81.09, 34.33, 30.55, 28.32, 26.83.

HRMS (ESI): *m/z* calc for $C_{16}H_{21}N_2O_3$ $[M+H]^+$: 289.1552, found: 289.1554.

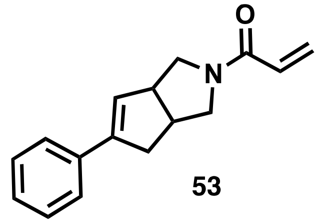

53

Compound **53**, 1-(5-phenyl-3,3a,6,6a-tetrahydrocyclopenta[c]pyrrol-2(1H)-yl)prop-2-en-1-one, was synthesized according to **synthetic procedure 38**. The reaction was performed on a 2.7 mmole scale to provide the desired compound in 74% yield (480 mg).

^{1}H NMR (400 MHz, $CDCl_3$): δ 7.46 - 7.39 (m, 2H), 7.38 - 7.30 (m, 2H), 7.29 - 7.24 (m, 1H), 6.55 - 6.28 (m, 2H), 6.03 (dd, *J* = 1.2, 19.2 Hz, 1H), 5.67 - 5.63 (m, 1H), 4.03 - 3.87 (m, 1H), 3.85 - 3.52 (m, 3H), 3.39 - 3.25 (m, 1H), 3.20 - 2.95 (m, 2H), 2.72 - 2.56 (m, 1H).

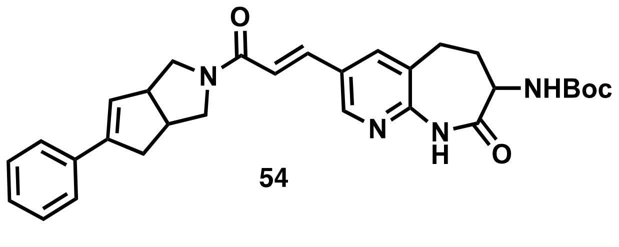

54

Compound **54**, (*E*)-*tert*-butyl (8-oxo-3-(3-oxo-3-(5-phenyl-3,3a,6,6a-tetrahydrocyclopenta[c]pyrrol-2(1H) -yl)prop-1-en-1-yl)-6,7,8,9-tetrahydro-5H-pyrido[2,3-b]azepin-7-yl)carbamate, was synthesized according to **general procedure 1**. The reaction was performed on a 280 µmole reaction to provide the desired product in 79% yield (115 mg).

^{1}H NMR (400 MHz, $CDCl_3$): δ 8.47 - 8.39 (m, 1H), 8.18 (d, *J* = 7.6 Hz, 1H), 7.74 - 7.57 (m, 2H), 7.45 (d, *J* = 7.2 Hz, 2H), 7.38 - 7.26 (m, 3H), 6.80 - 6.68 (m, 1H), 6.05 (d, *J* = 13.6 Hz, 1H), 5.59 - 5.52 (m, 1H), 4.34 - 4.23 (m, 1H), 4.09 - 3.98 (m, 1H), 3.91 - 3.87 (m, 1H), 3.86 - 3.79 (m, 1H), 3.72 - 3.58 (m, 1H), 3.45 - 3.36 (m, 1H), 3.23 - 3.02 (m, 2H), 2.86 - 2.63 (m, 4H), 2.12 - 1.99 (m, 1H), 1.42 (s, 9H).

LCMS (254 nm): *m/z* for $C_{30}H_{35}N_4O_3$ $[M+H]^+$: 515.2, ≥99% pure.

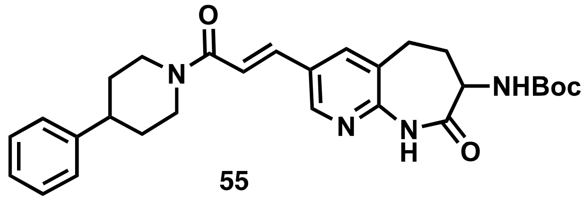

55

Compound **55**, (*E*)-*tert*-butyl (8-oxo-3-(3-oxo-3-(4-phenylpiperidin-1-yl)prop-1-en-1-yl)-6,7,8,9-tetrahydro- 5H-pyrido[2,3-b]azepin-7-yl)carbamate, was synthesized according to **general procedure 1**. The reaction was performed on a 505 µmole scale to provide the desired product in 73% (152 mg).

^{1}H NMR (400 MHz, $CDCl_3$): δ 8.44 (s, 1H), 8.30 (s, 1H), 7.73 (s, 1H), 7.70 (d, *J* = 15.6 Hz, 1H), 7.40 - 7.30 (m, 2H), 7.24-7.22 (m, 3H), 6.97 (d, *J* = 15.6 Hz, 1H), 5.60 (d, *J* = 7.2 Hz, 1H), 4.92 (d, *J* = 11.2 Hz, 1H), 4.41 - 4.21 (m, 2H), 3.33 - 3.21(m, 1H), 3.00 - 2.91 (m, 1H), 2.91 - 2.71 (m, 5H), 2.10 - 2.00 (m, 2H), 1.77 - 1.70 (m, 2H), 1.40 (s, 9H).

LCMS (254 nm): *m/z* for $C_{28}H_{35}N_4O_4$ $[M+H]^+$: 491.2, 95.5% pure.

VI. ^{1}H, ^{13}C NMR Spectra

General Remarks. Compounds **2-9**,**23-51**, and **53-55** were synthesized and characterized by WuXi AppTec.

^{1}H NMR (500 MHz, chloroform-d):

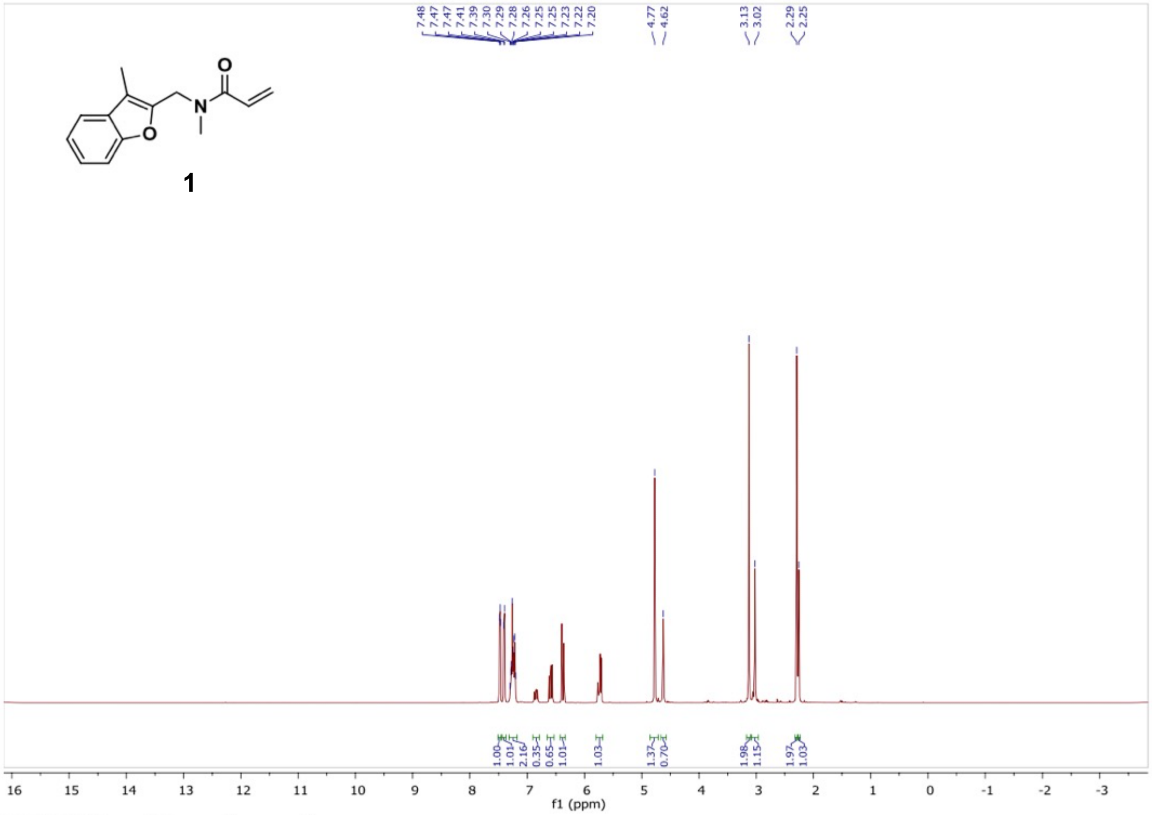

^{13}C NMR (126 MHz, chloroform-d):

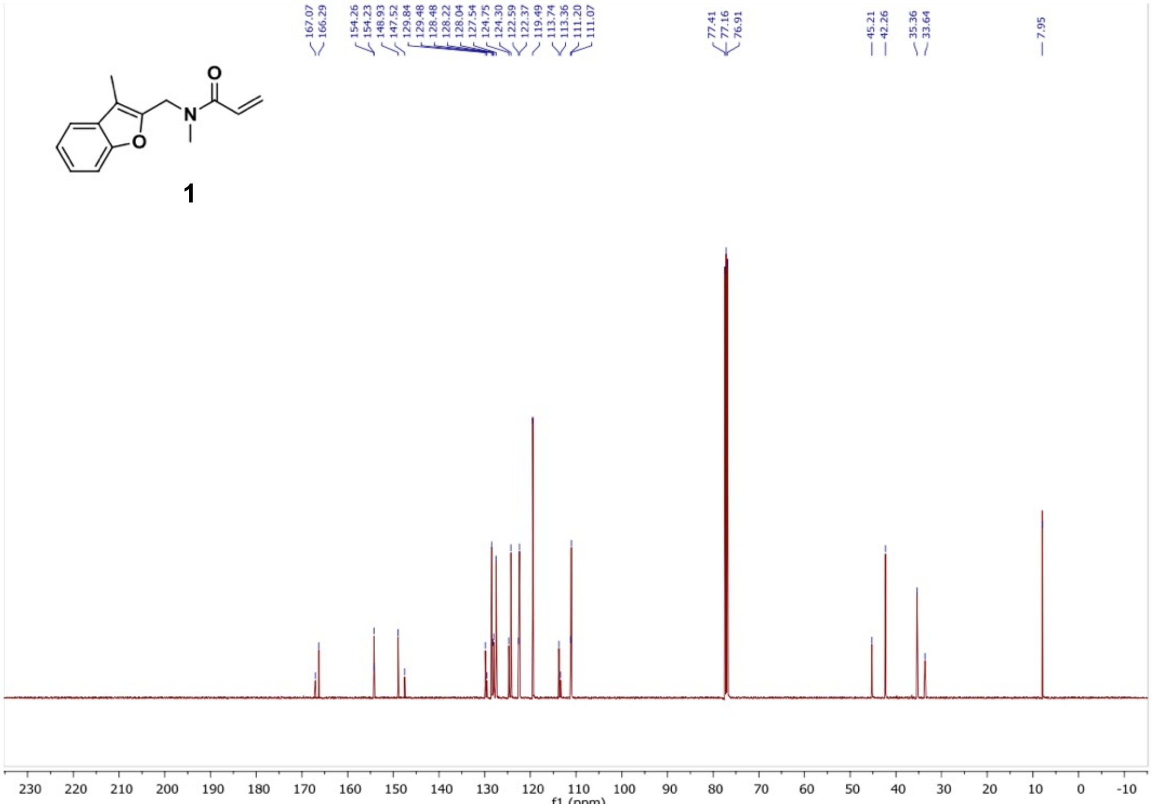

^{1}H NMR

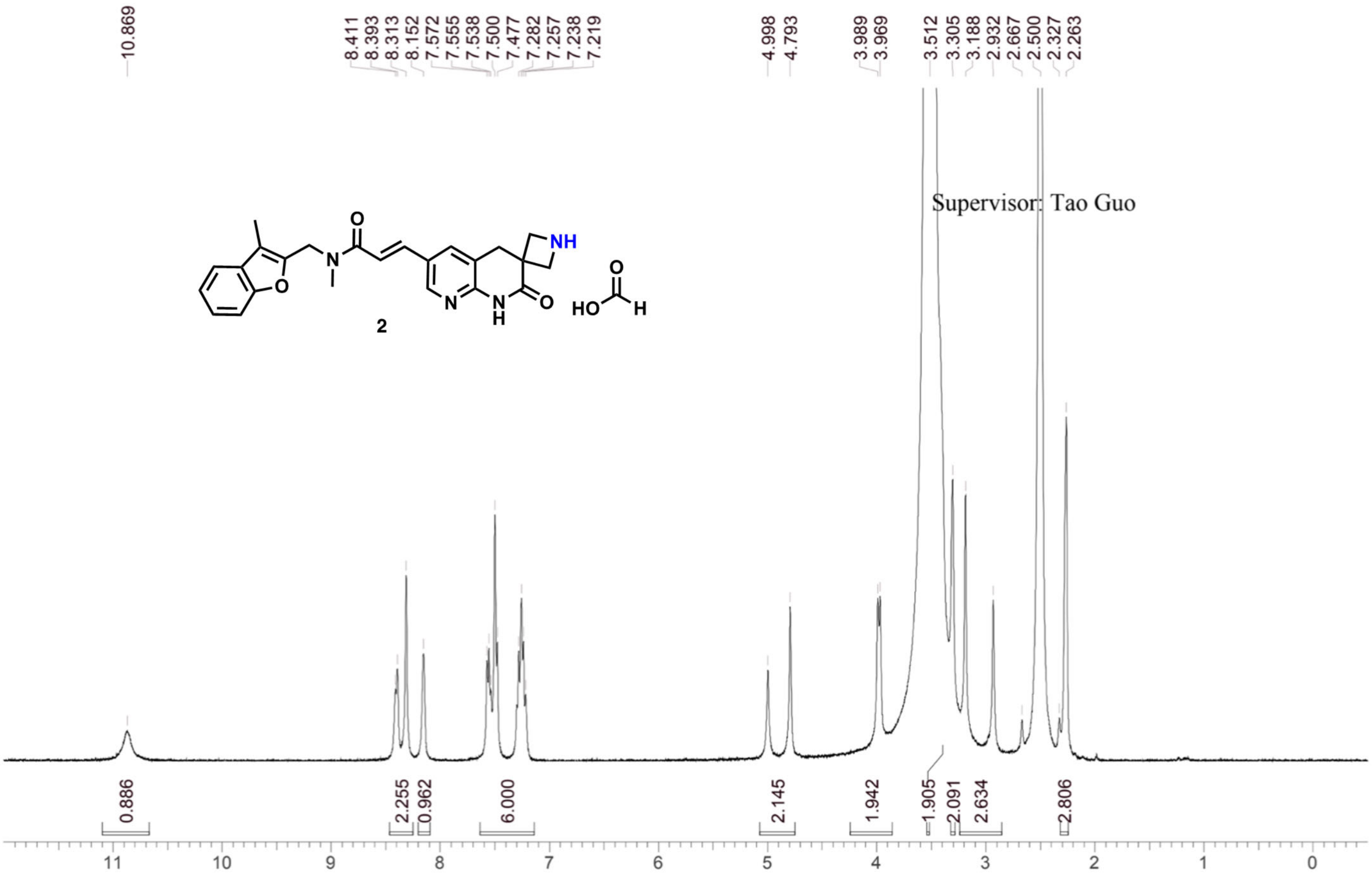

^{13}C NMR

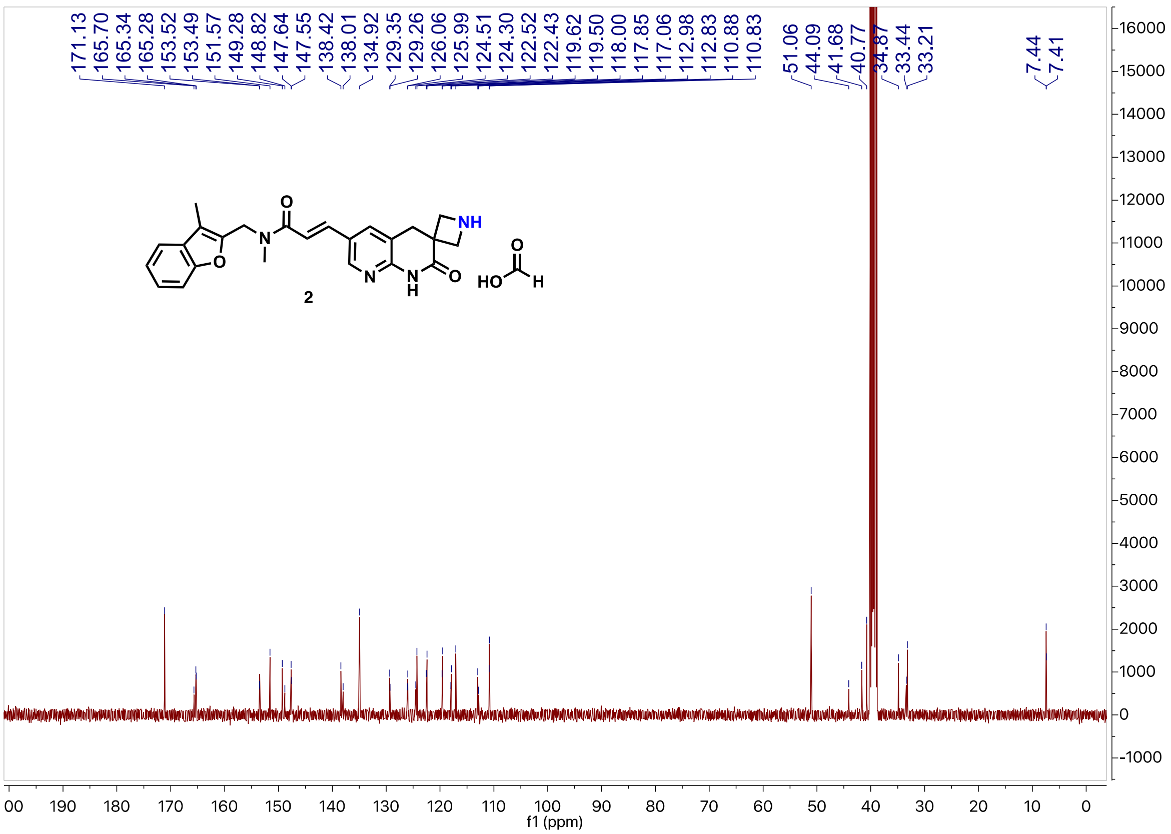

^1H NMR

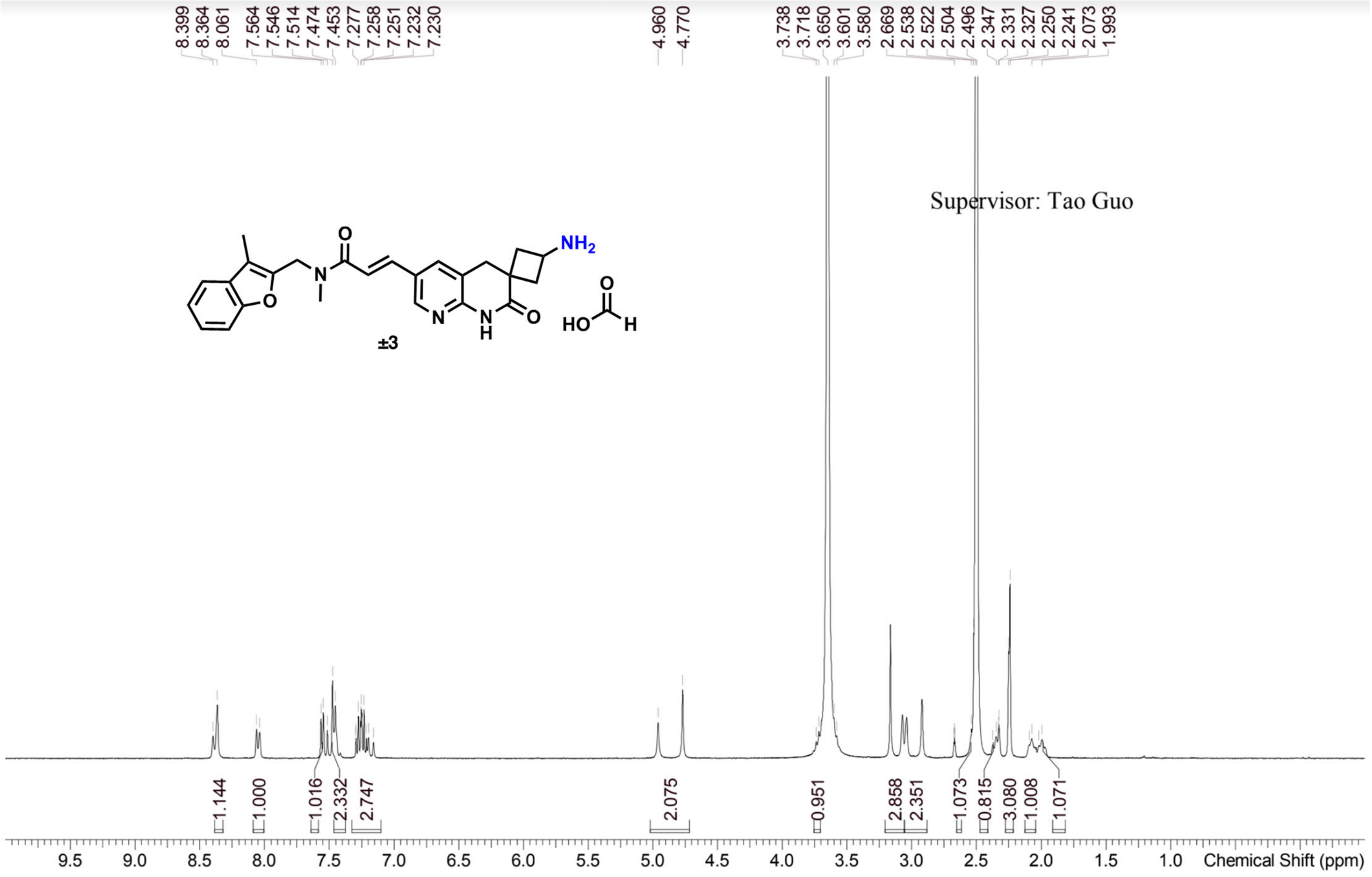

LCMS

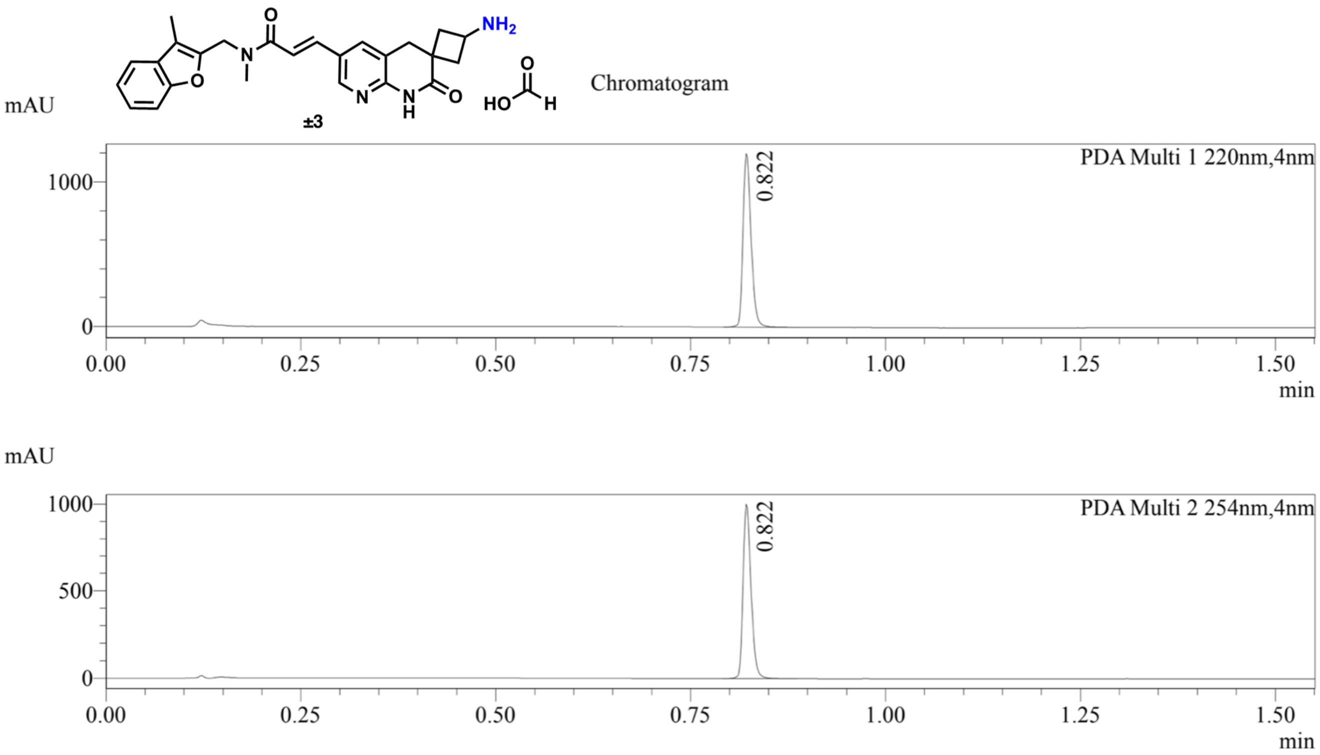

1 PDA Multi 1 / 220nm,4nm
2 PDA Multi 2 / 254nm,4nm

^{1}H NMR

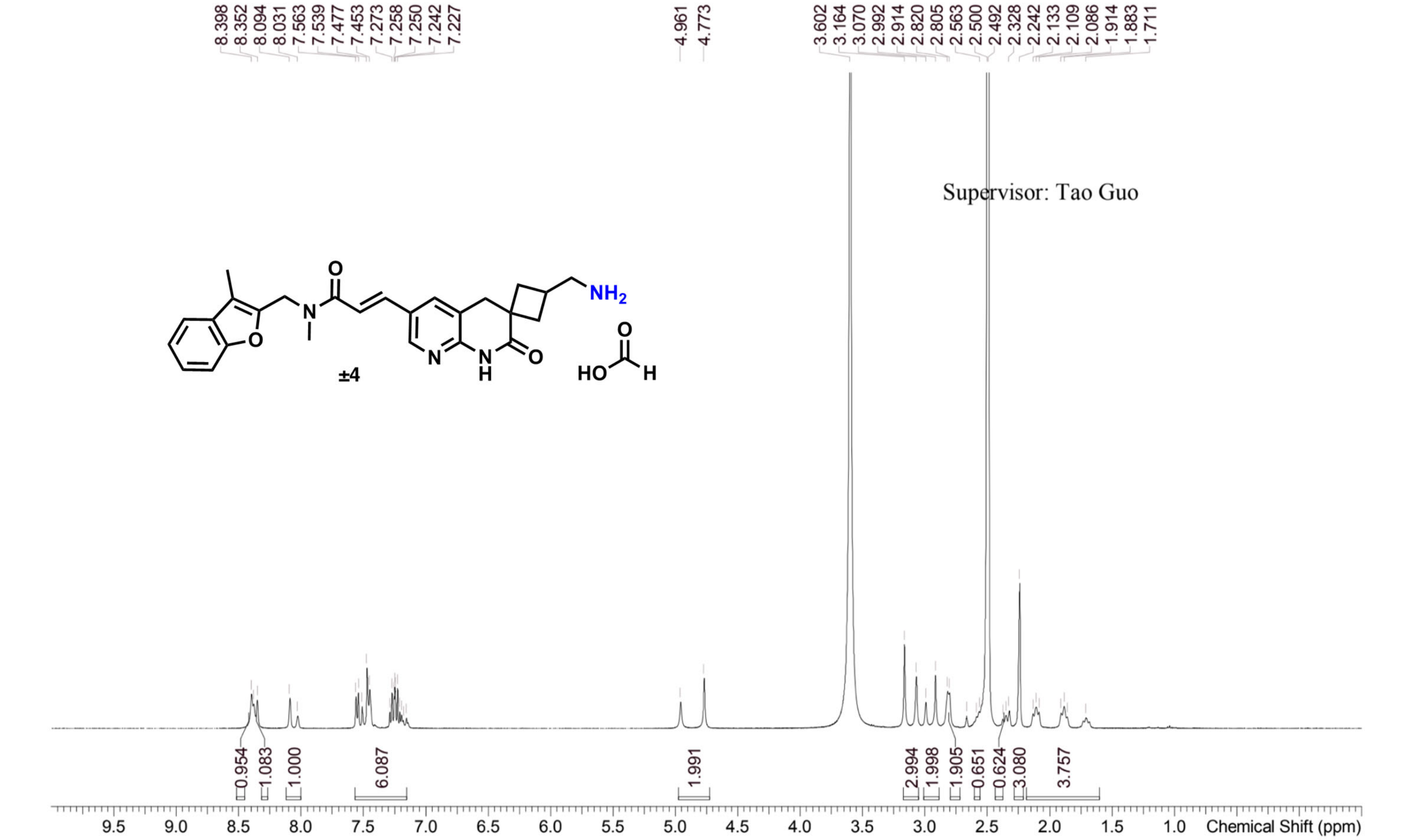

LCMS

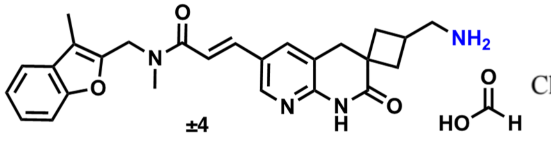

Chromatogram

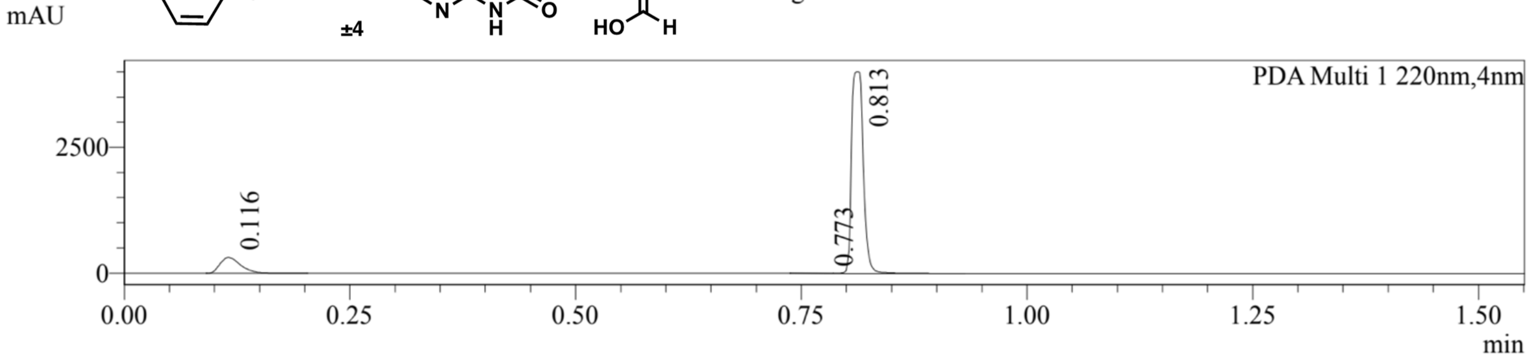

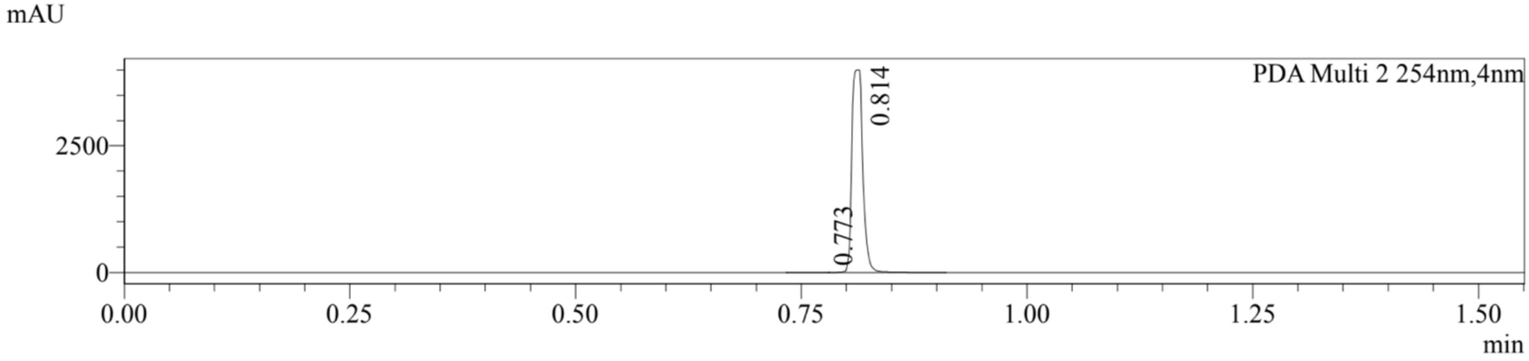

^{1}H NMR

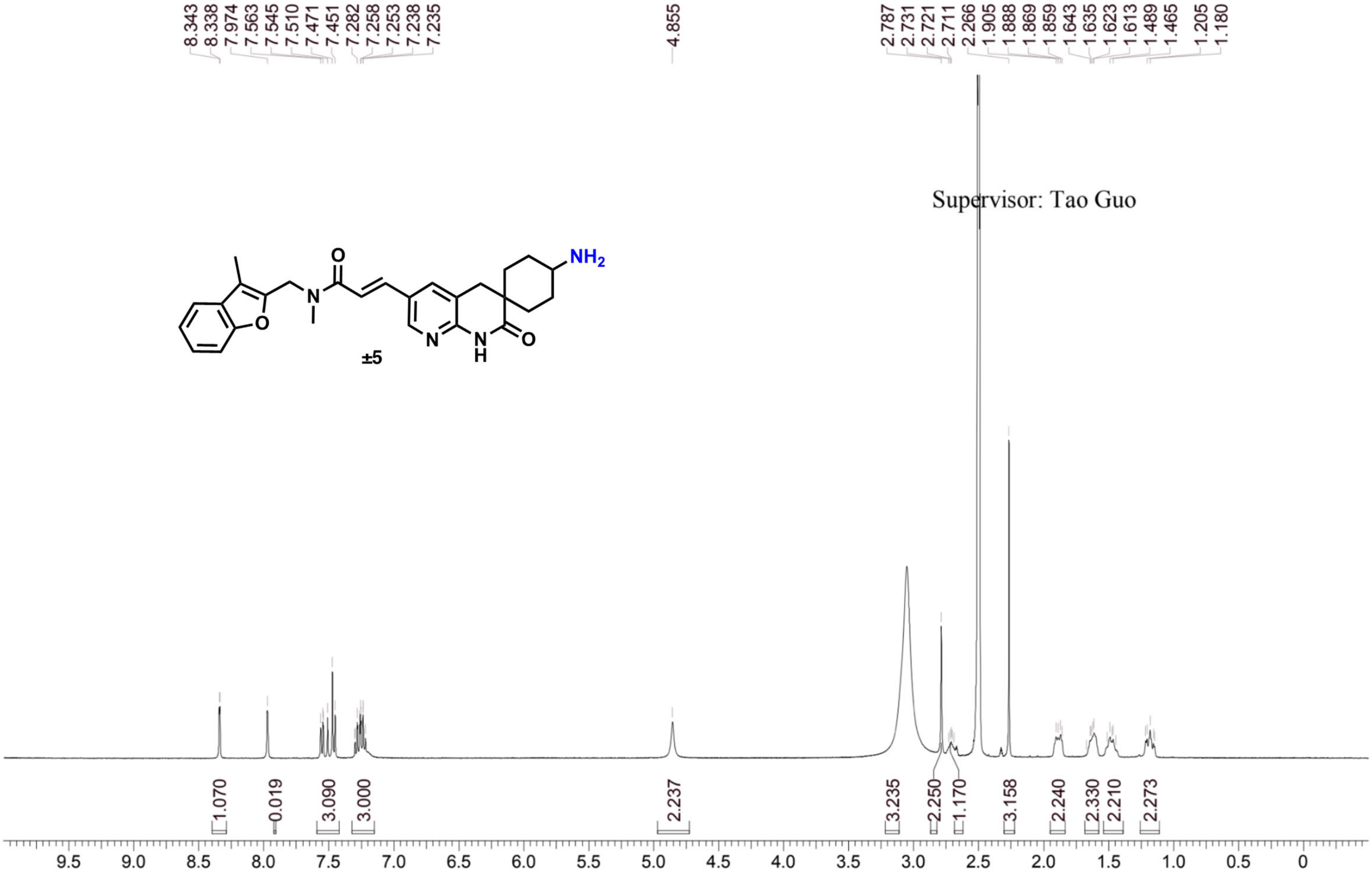

LCMS

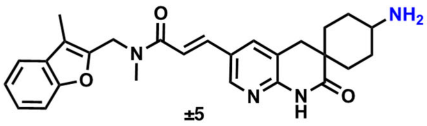

Chromatogram

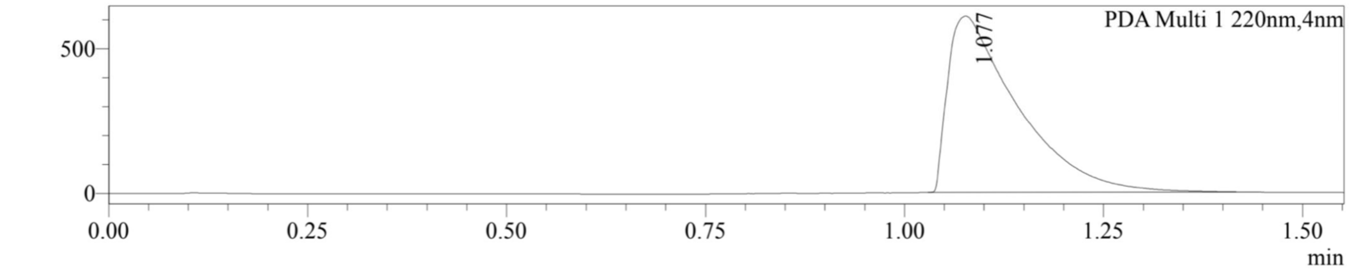

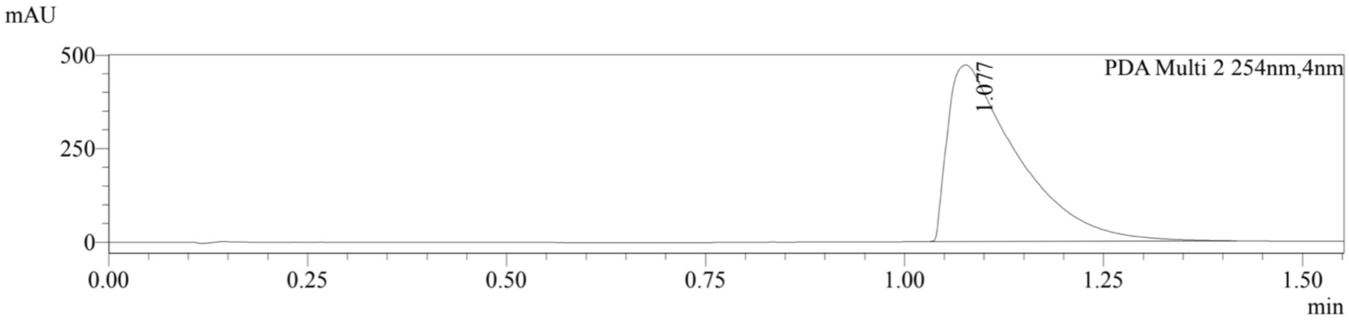

^{1}H NMR

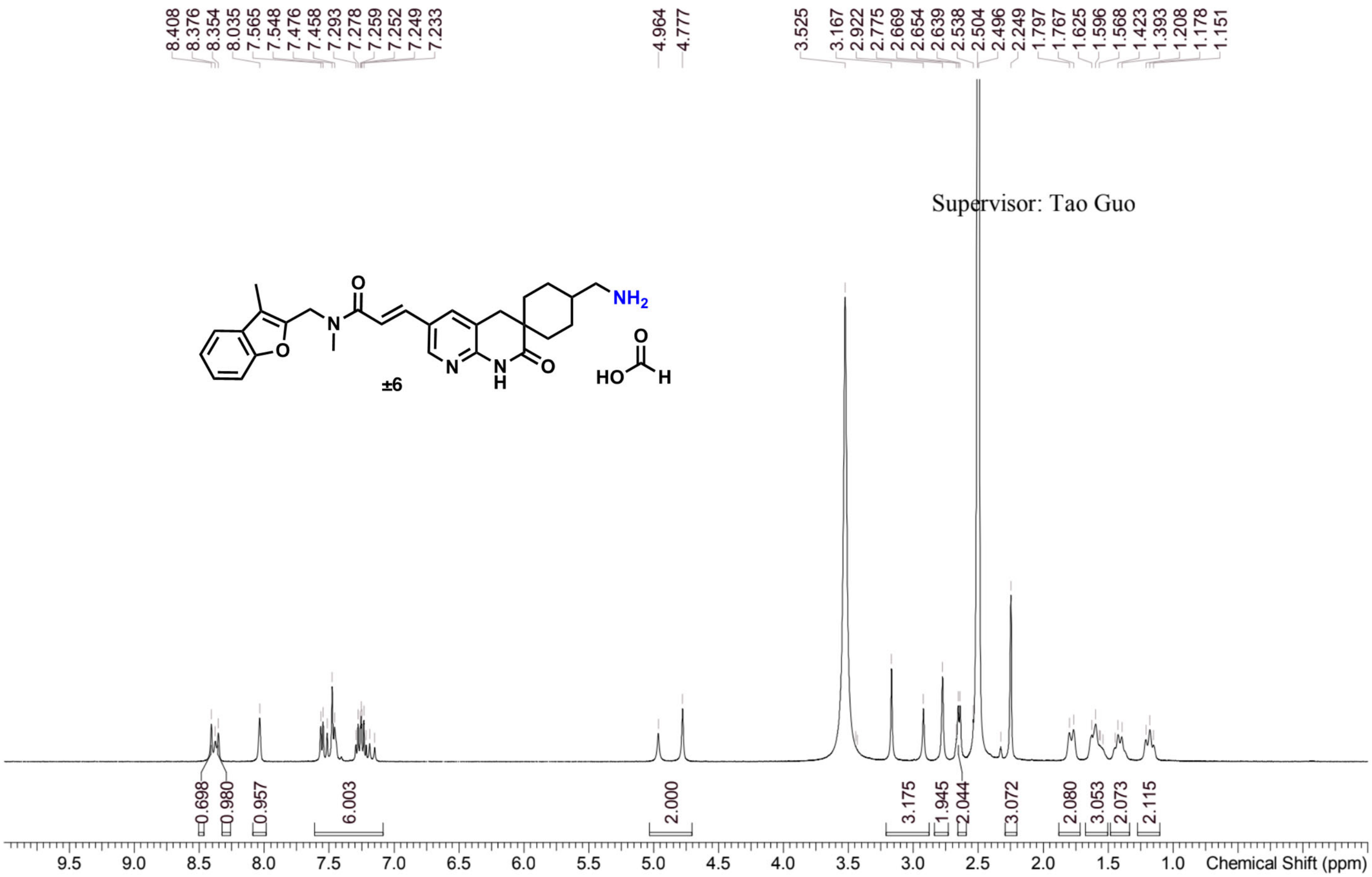

^{13}C NMR

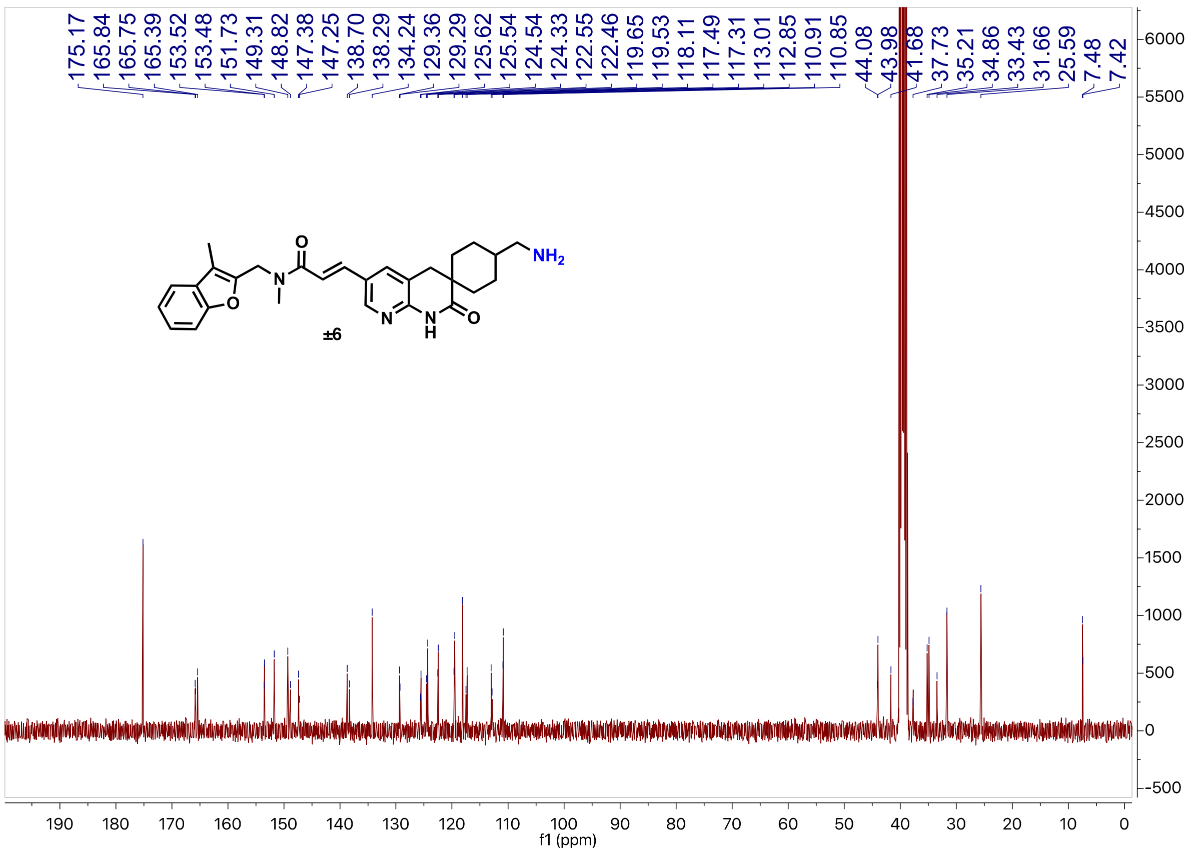

^{1}H NMR

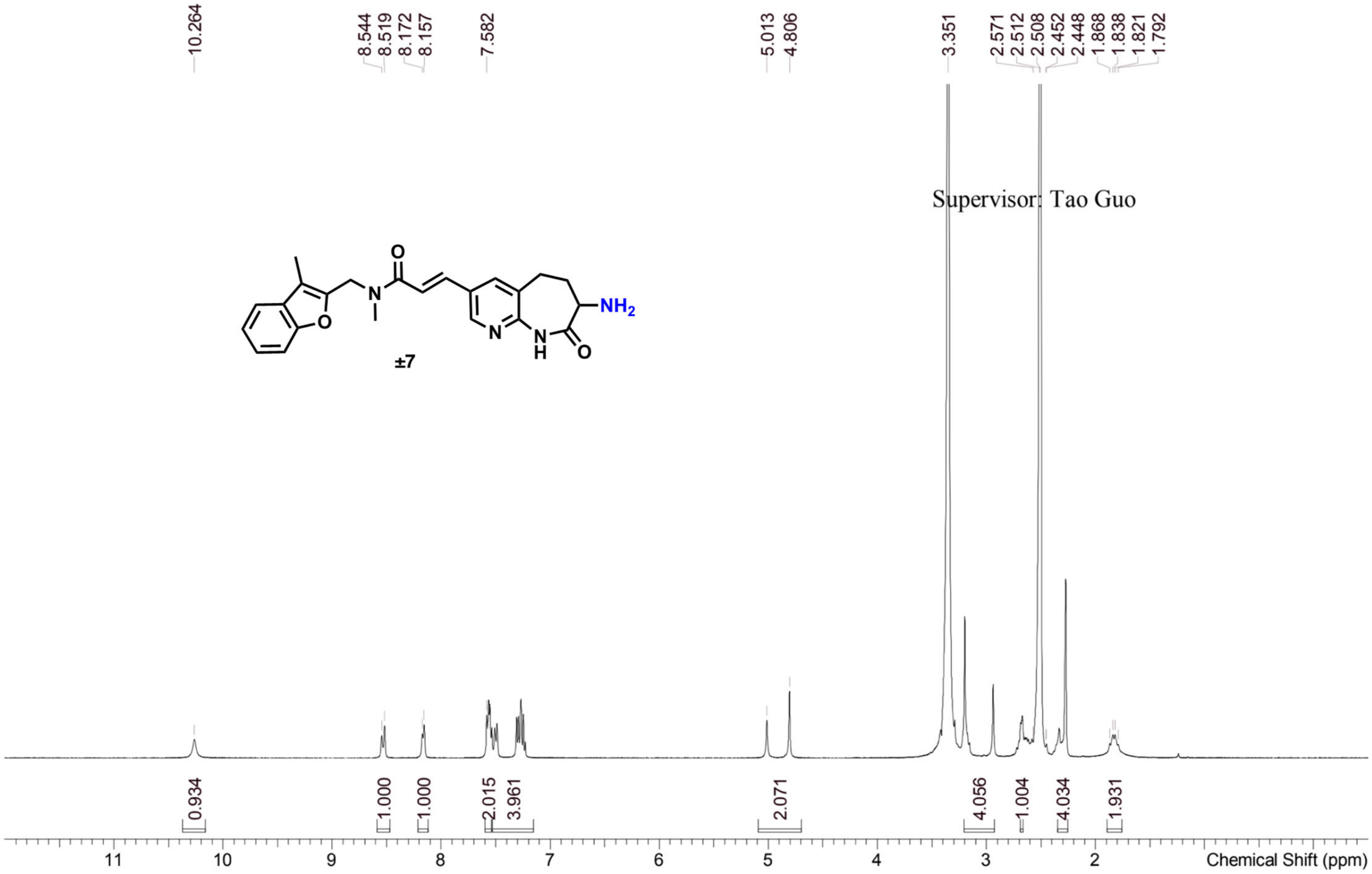

^{13}C NMR

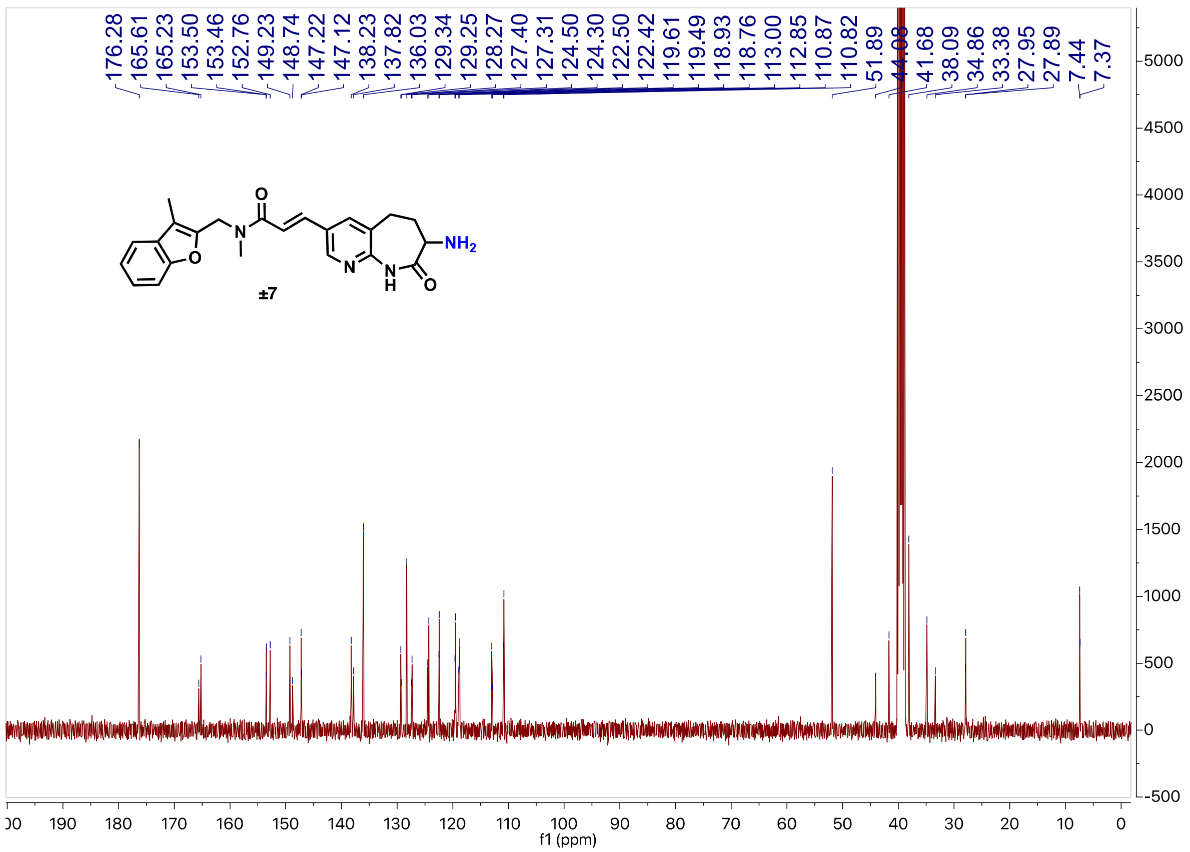

^{1}H NMR (DMSO-d_6, 25 ºC)

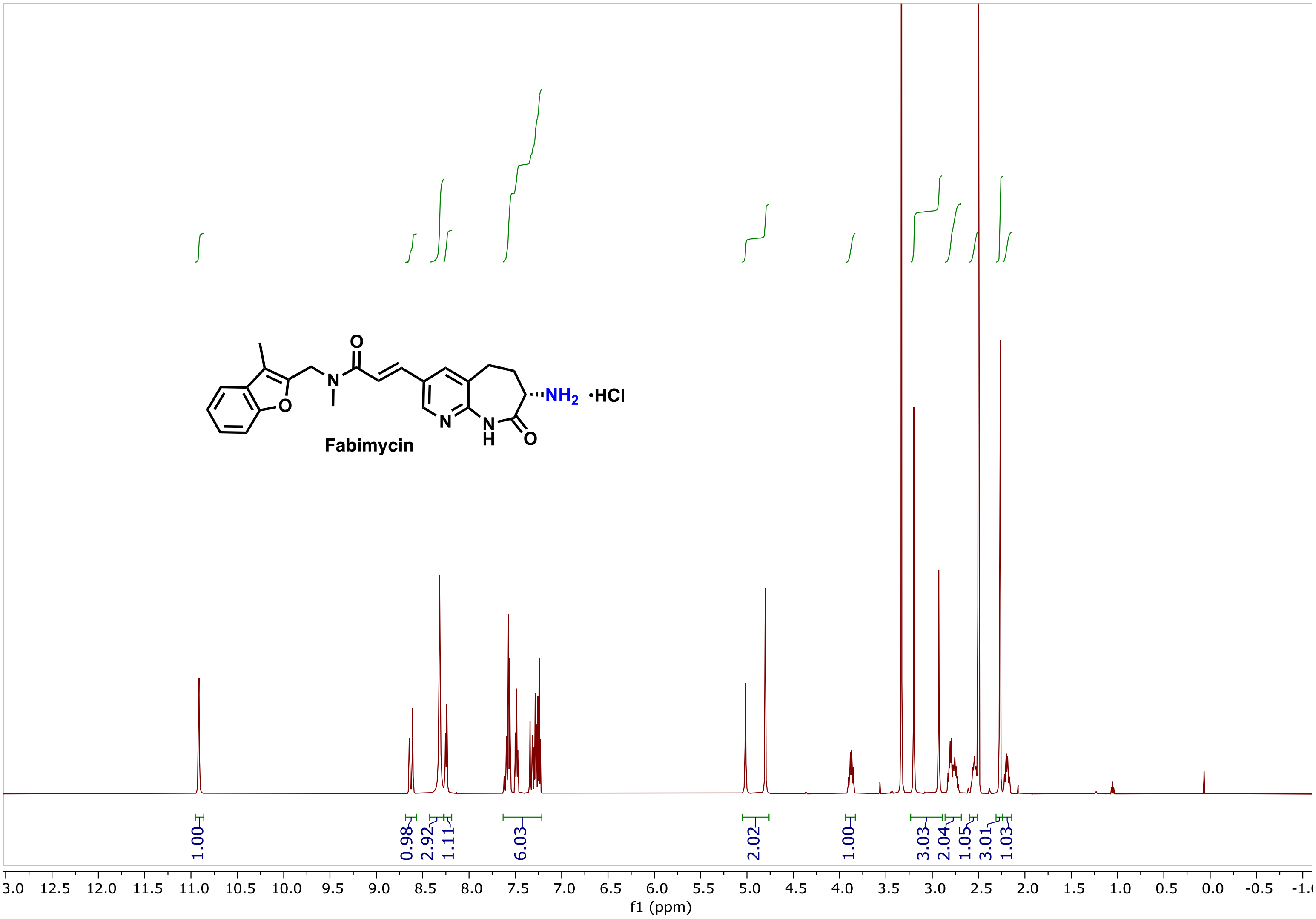

^{1}H NMR (DMSO-d_6, 120 ºC)

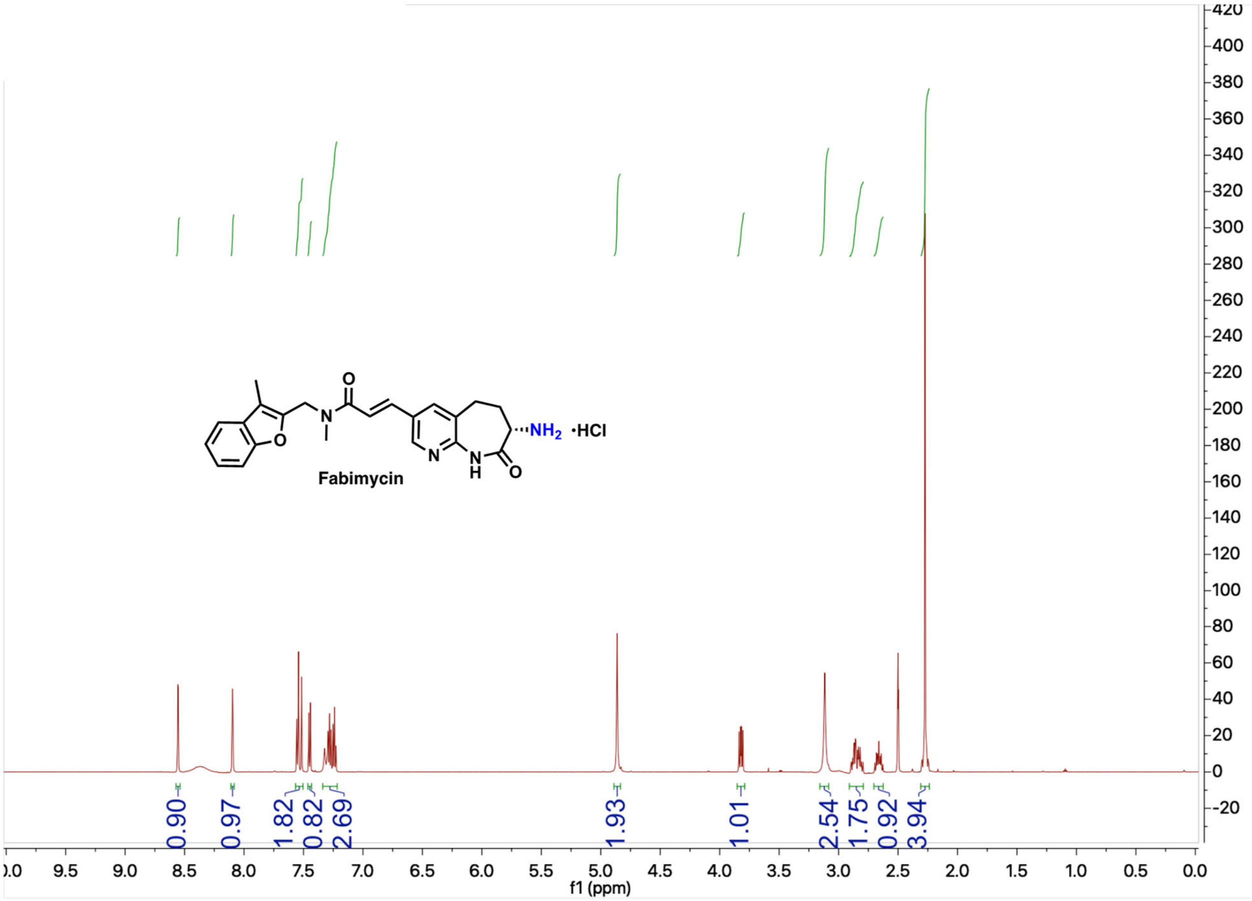

^{13}C NMR (DMSO-d_6, 120 ºC)

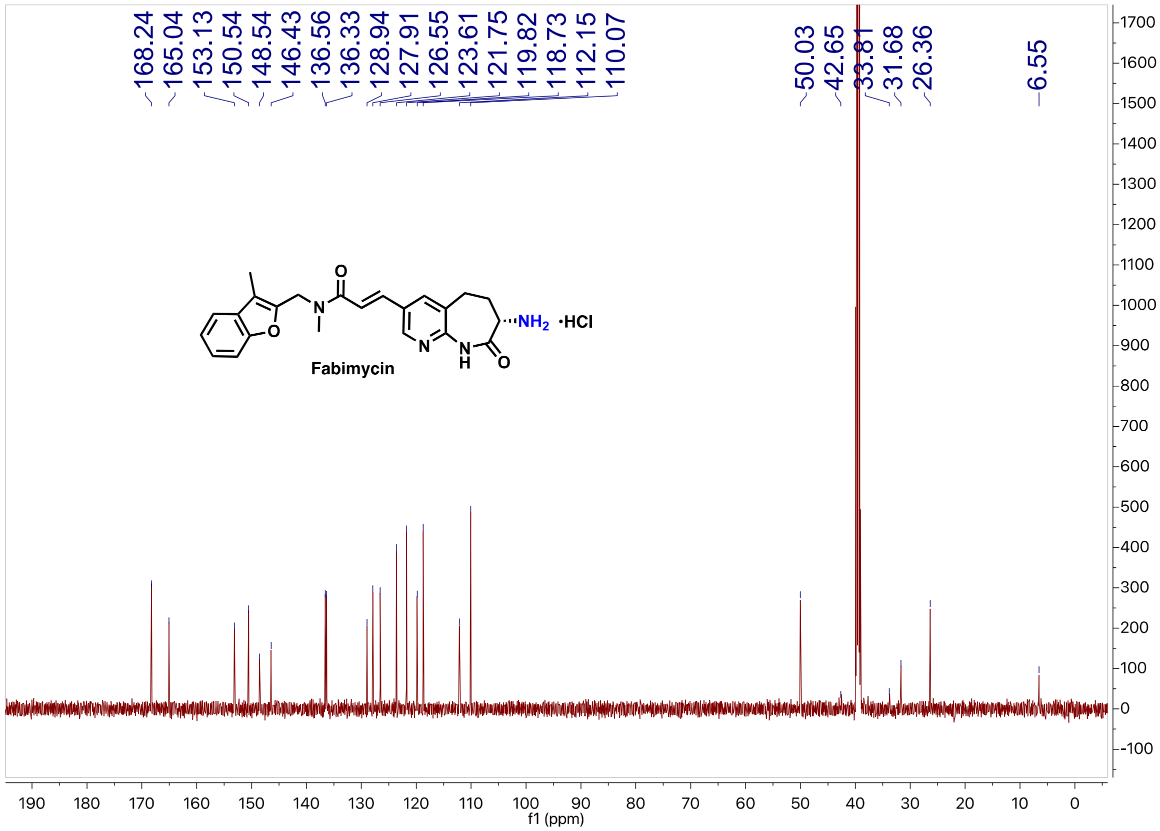

^{1}H NMR

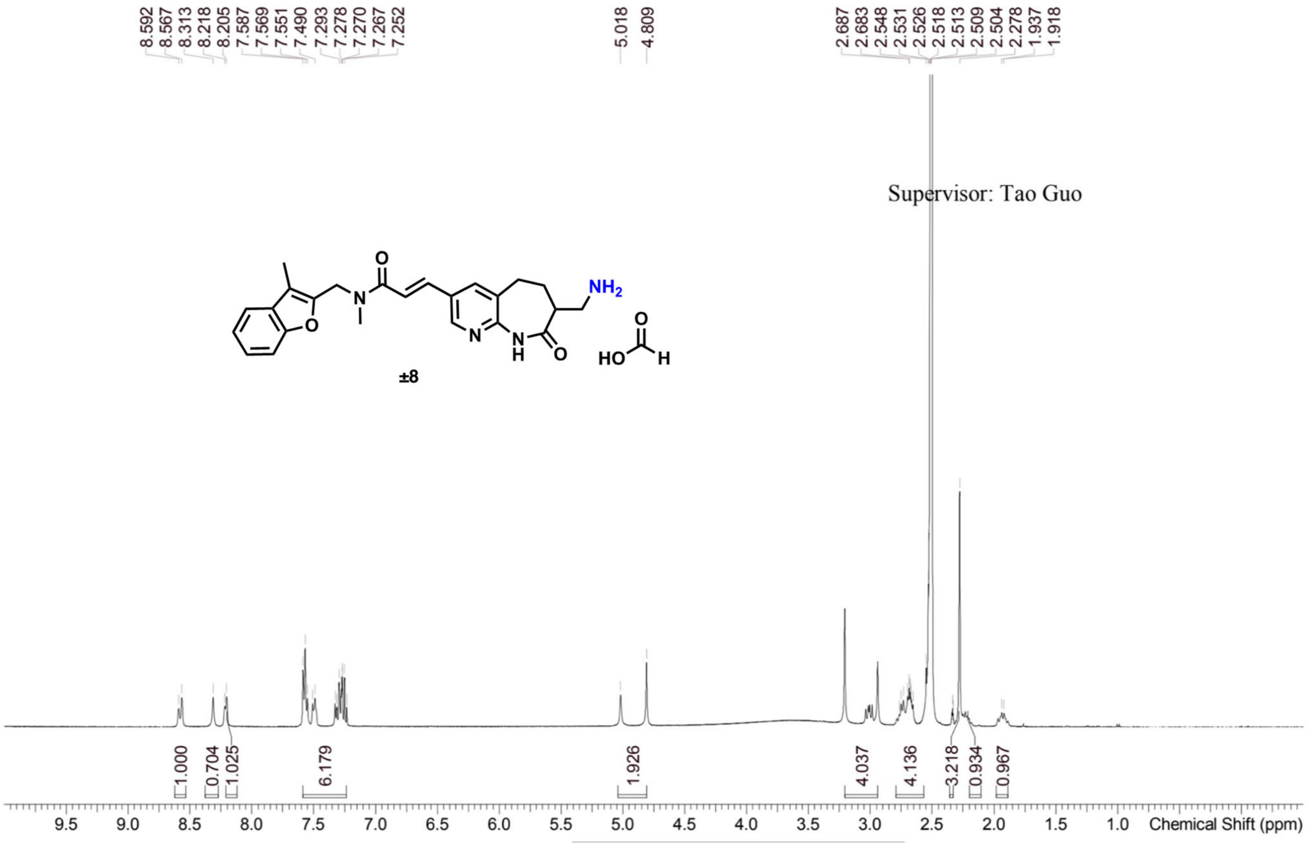

^{13}C NMR

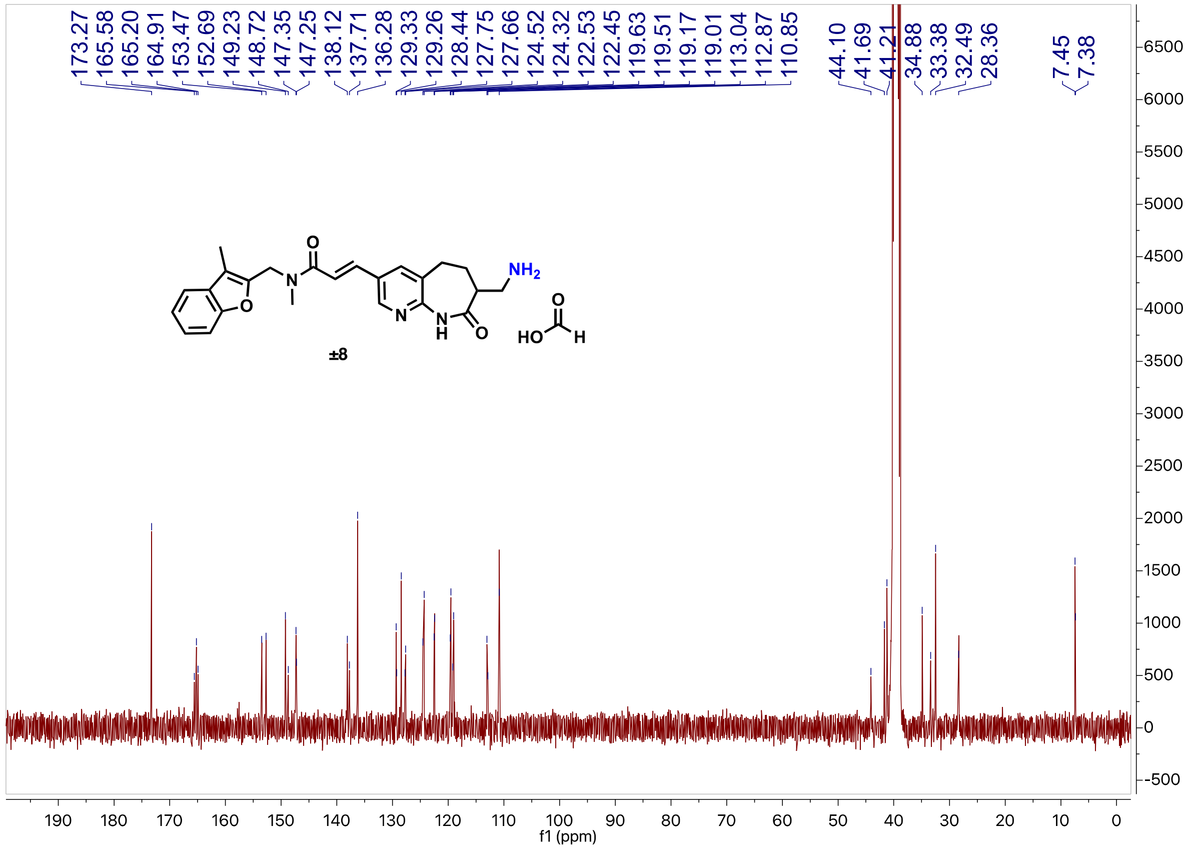

^{1}H NMR

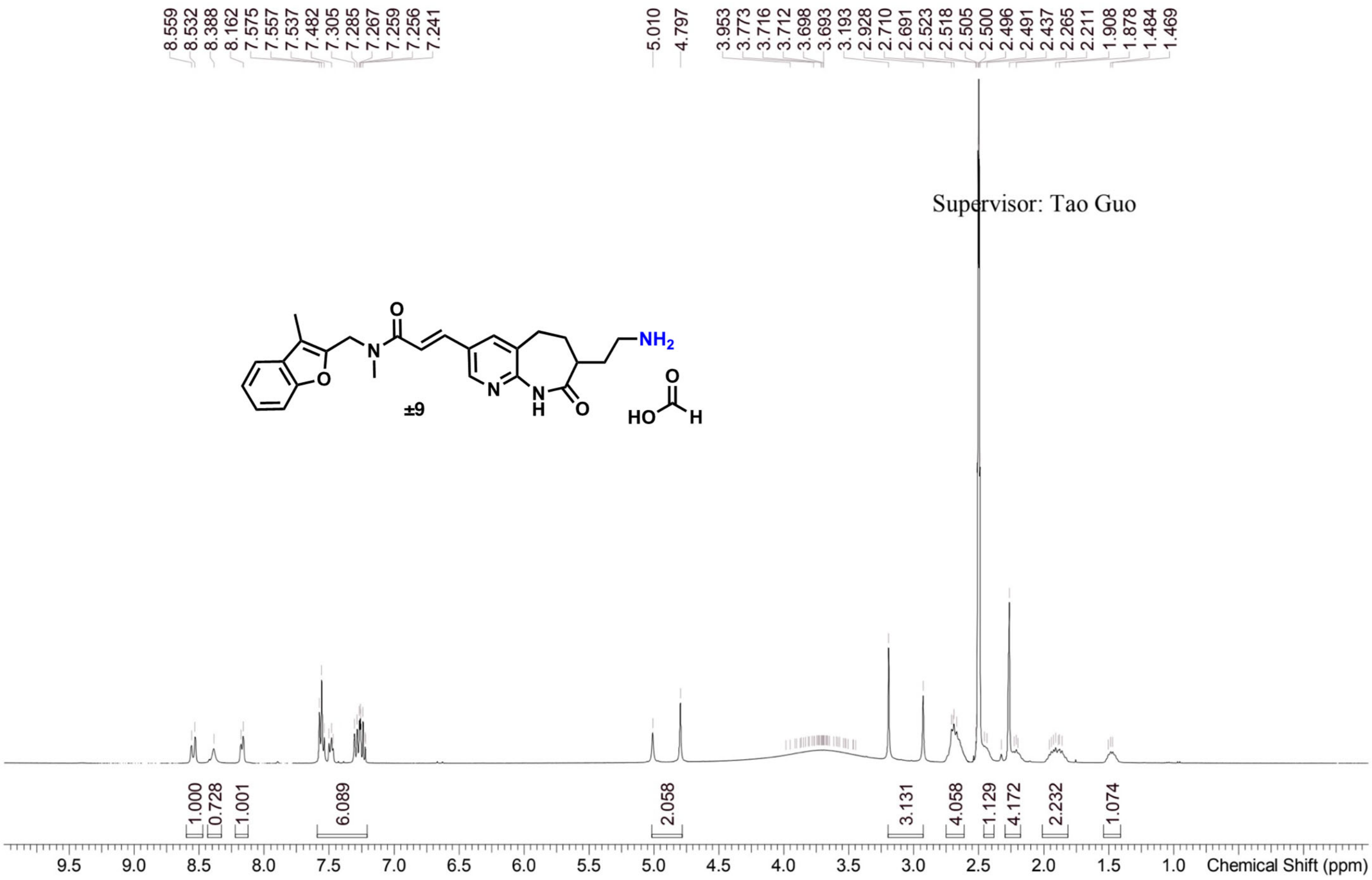

^{13}C NMR

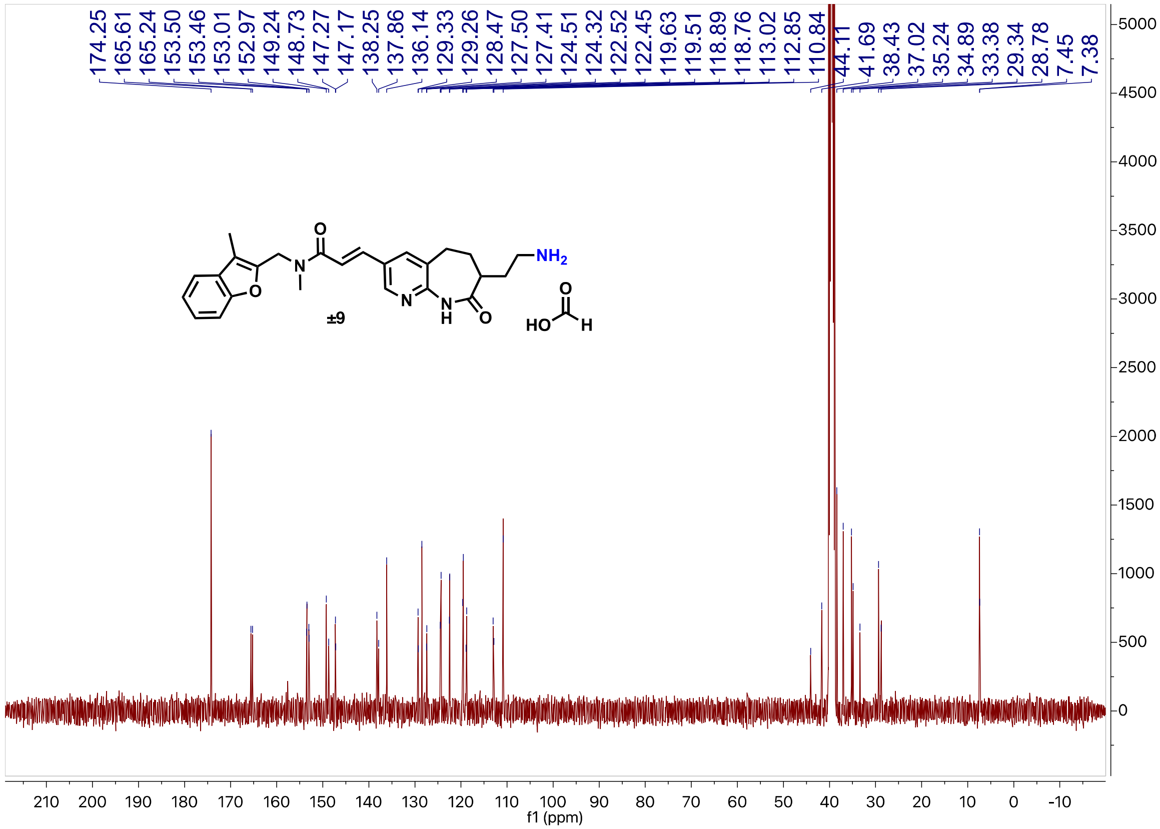

^{1}H NMR

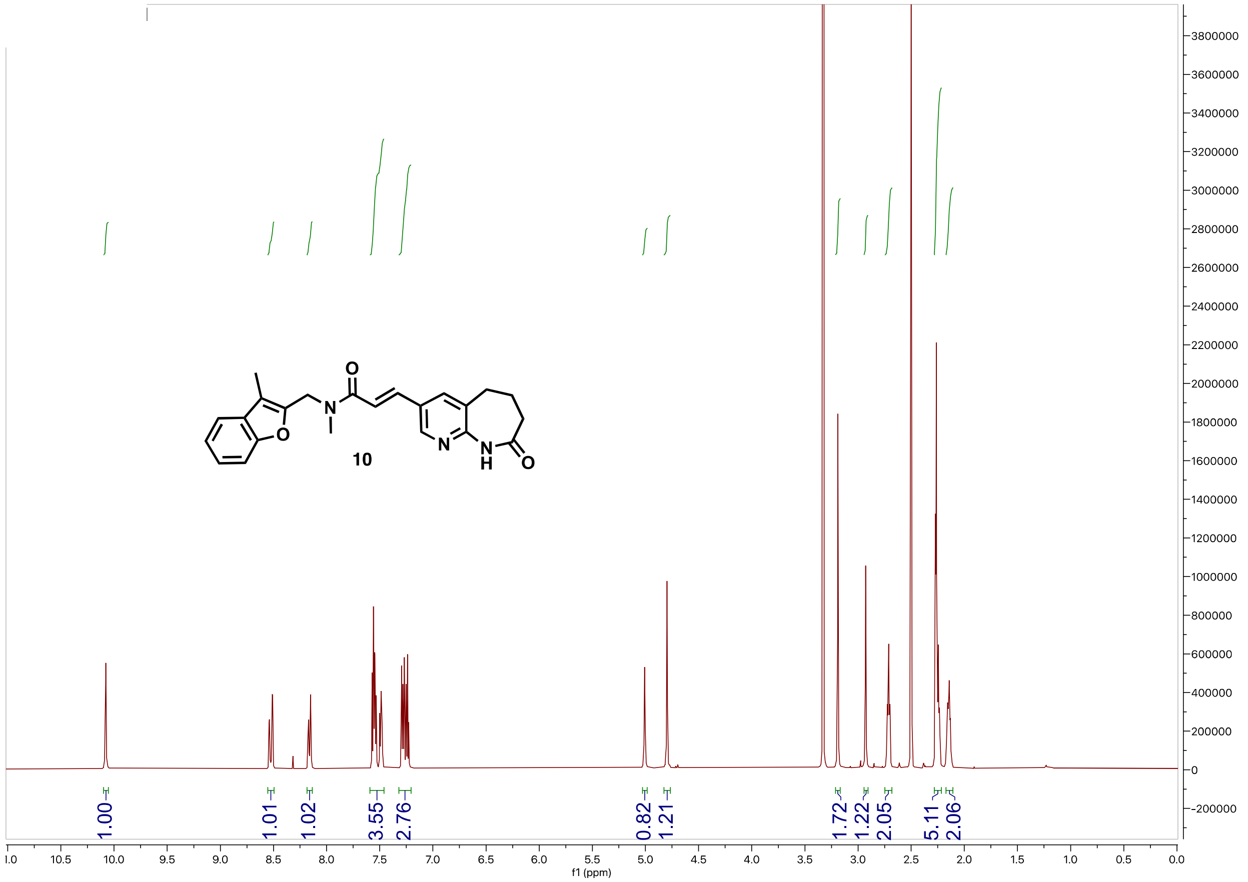

^{13}C NMR

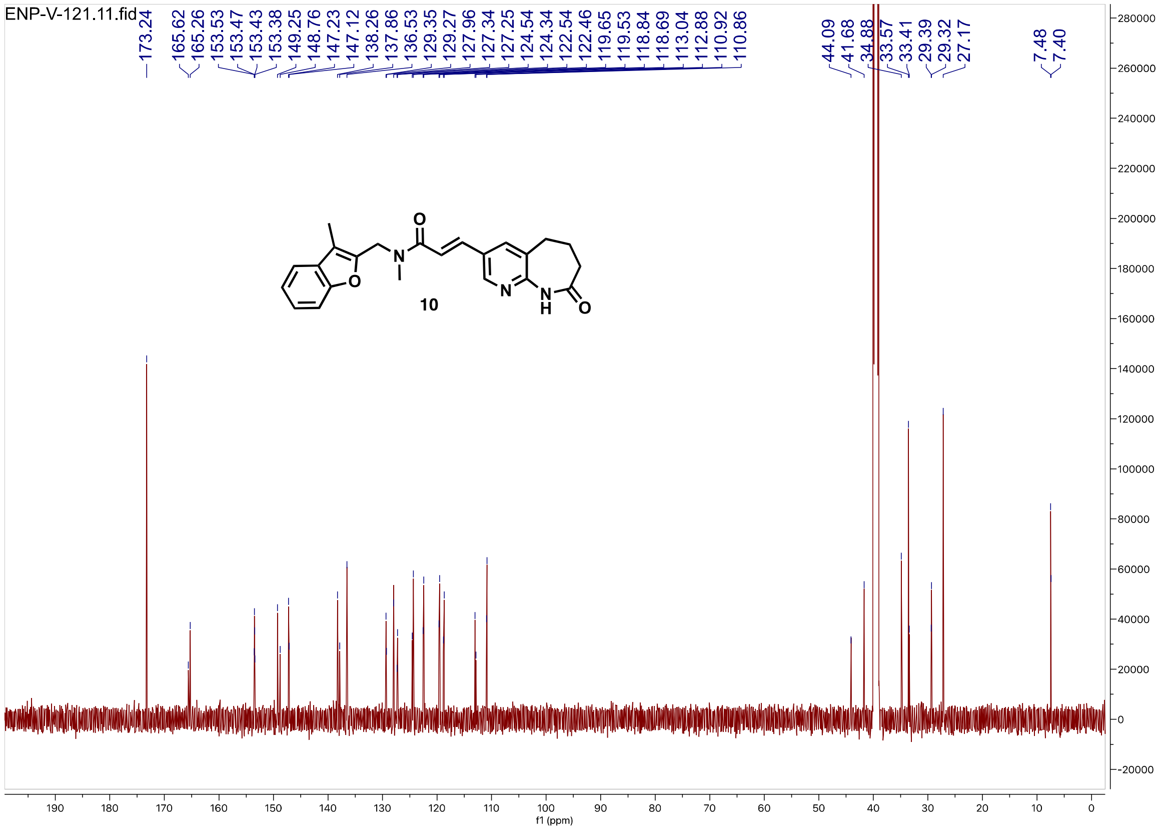

^{1}H NMR

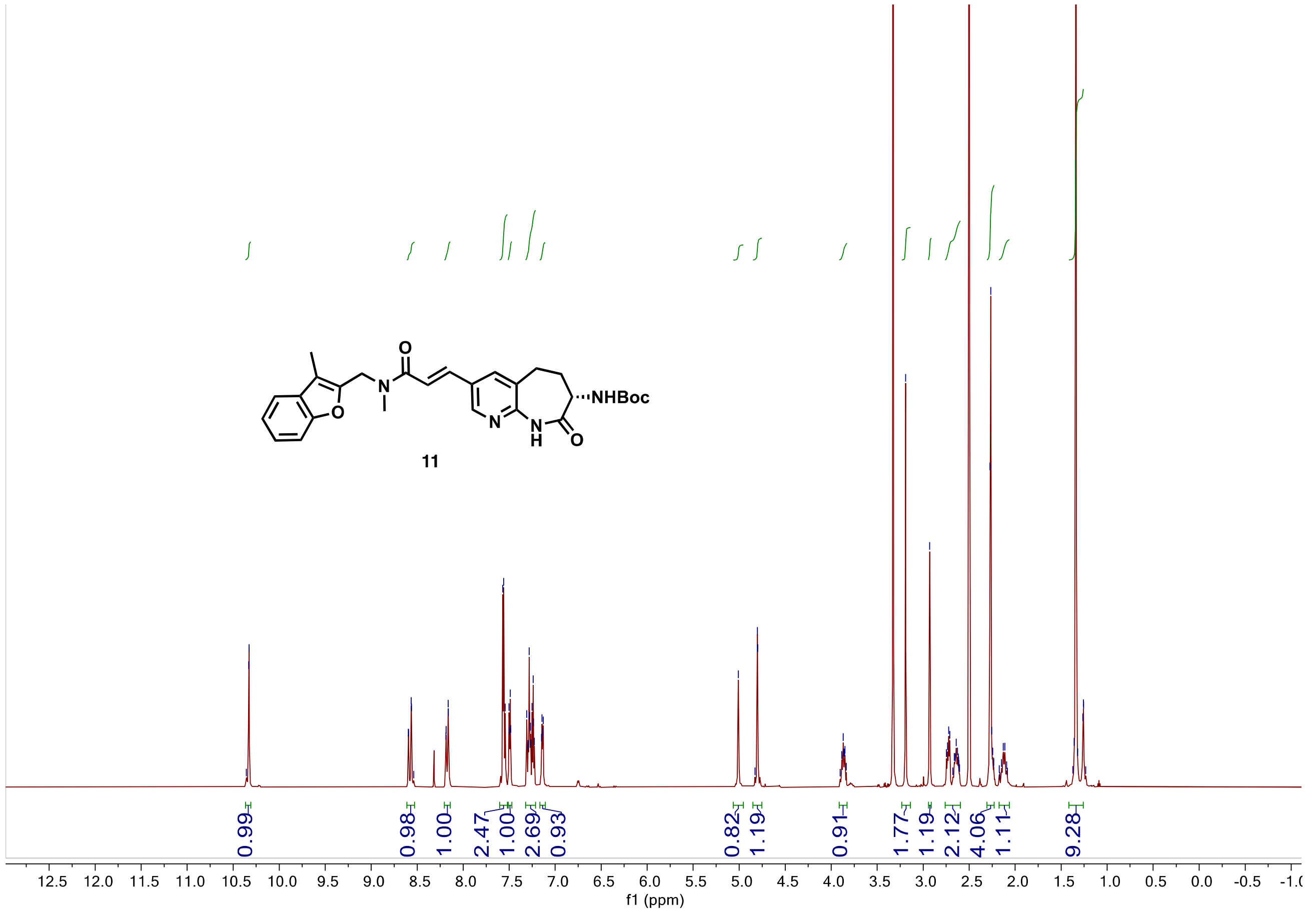
NHBoc
O
N
H
11
0.99
0.98
1.00
2.47
1.00
2.69
0.93
0.82
1.19
0.91
1.77
1.19
2.12
4.06
1.11
9.28
f1 (ppm)

^{13}C NMR

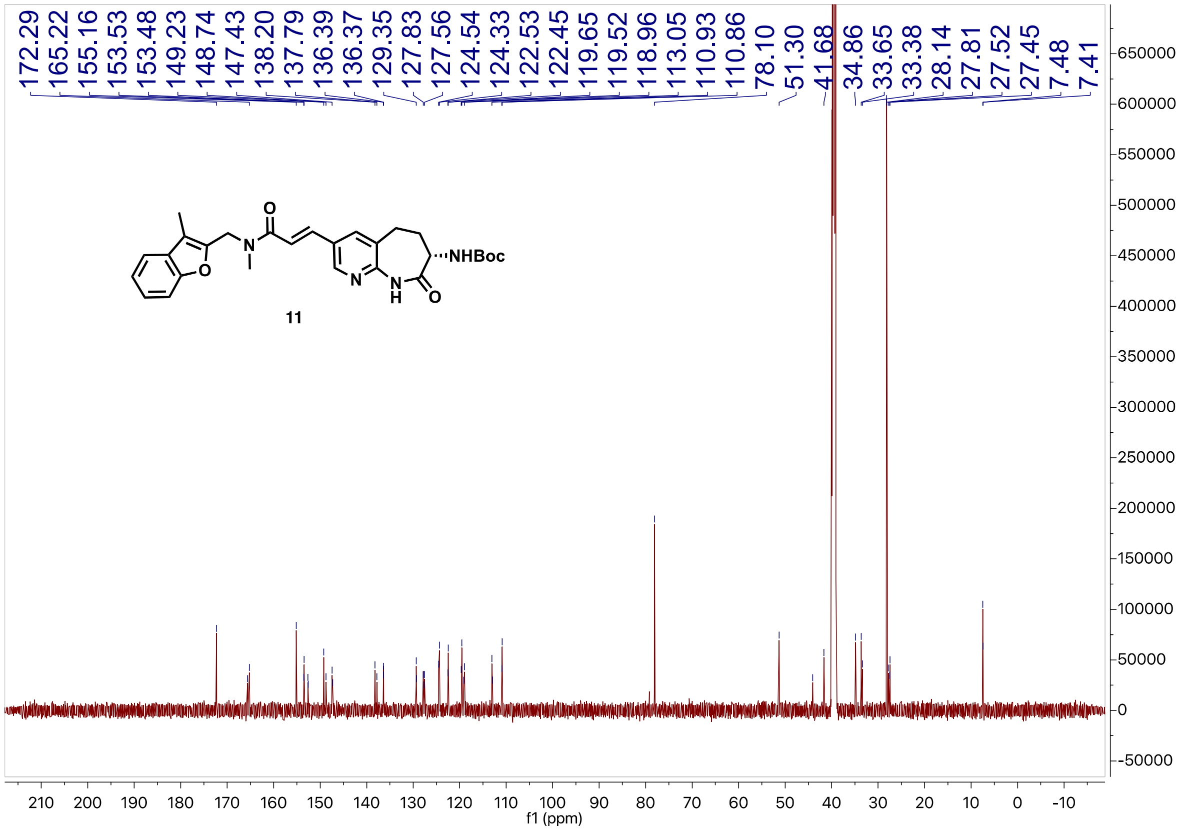
172.29
165.22
155.16
153.53
153.48
149.23
148.74
147.43
138.20
137.79
136.39
136.37
129.35
127.83
127.56
124.54
124.33
122.53
122.45
119.65
119.52
118.96
113.05
110.93
110.86
78.10
51.30
41.68
34.86
33.65
33.38
28.14
27.81
27.52
27.45
7.48
7.41
NHBoc
11
f1 (ppm)

^{1}H NMR

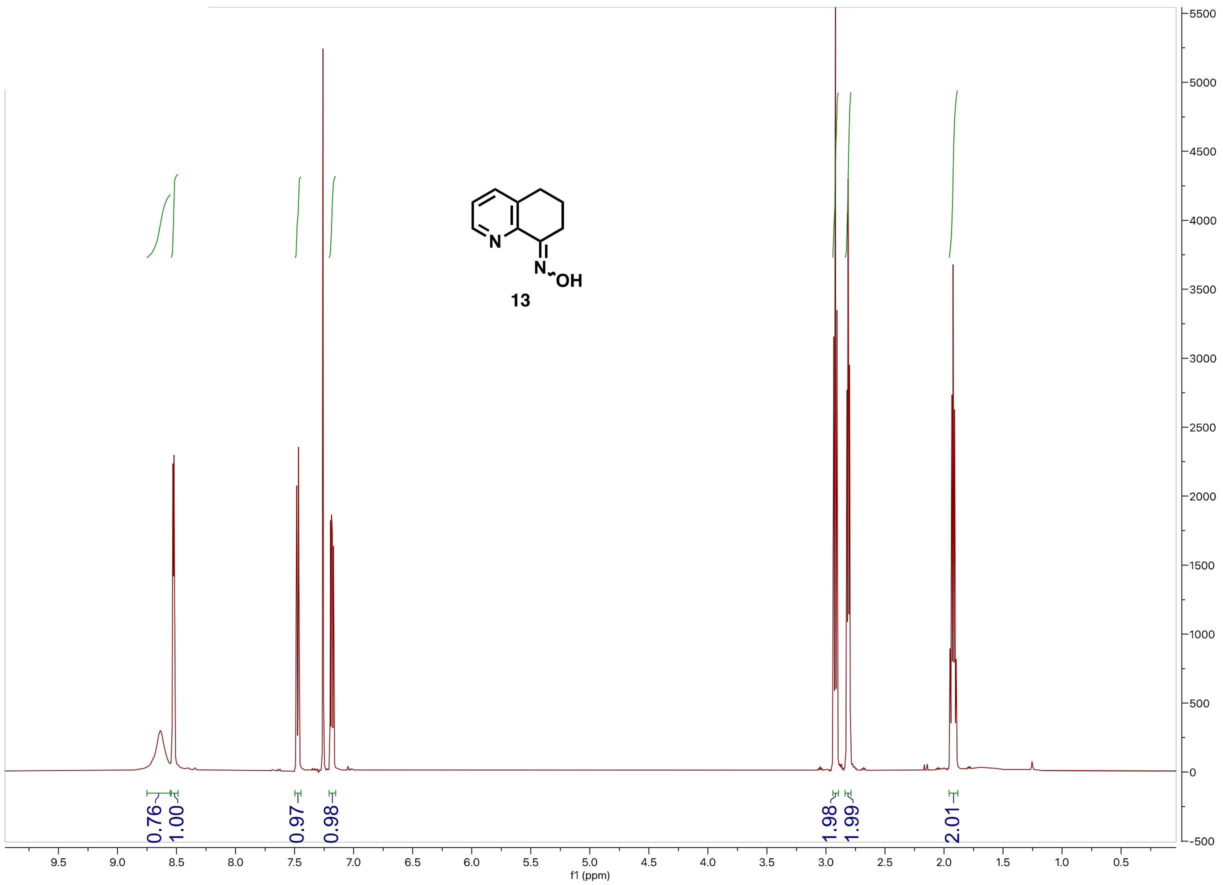

^{13}C NMR

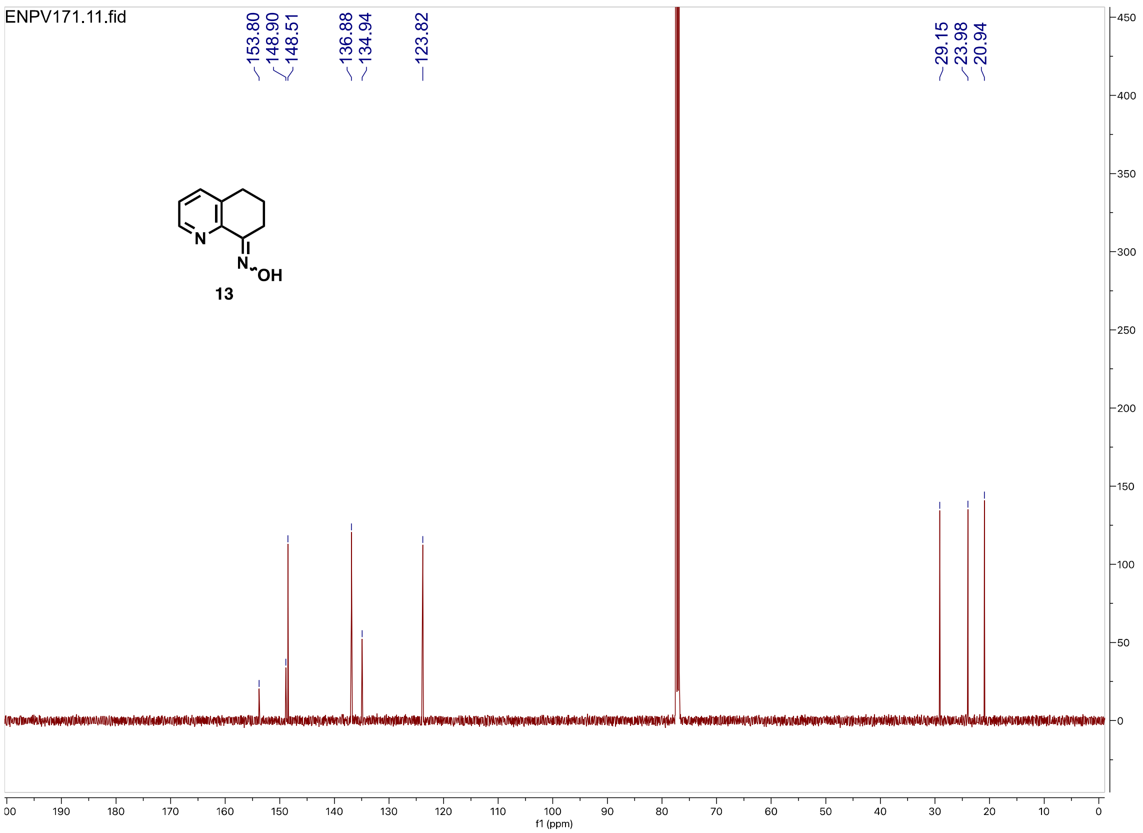

^{1}H NMR

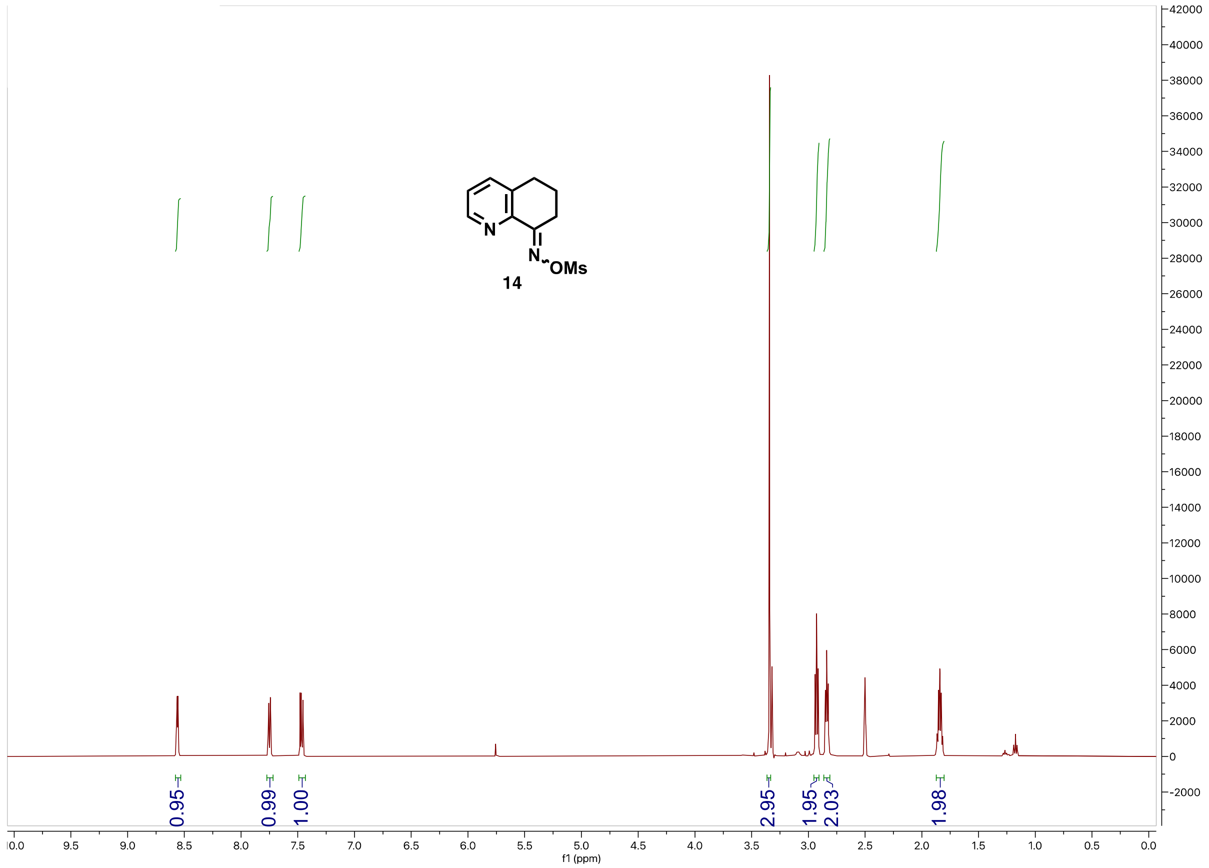

^{13}C NMR

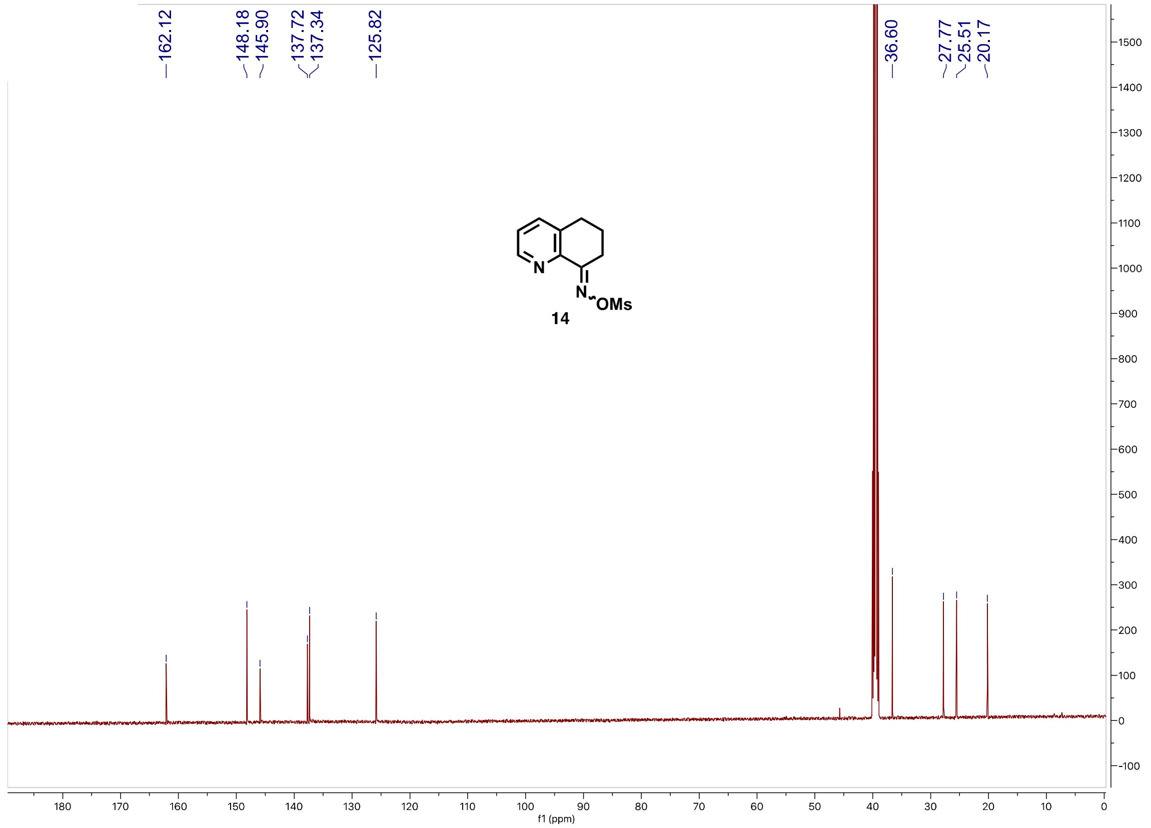

^{1}H NMR

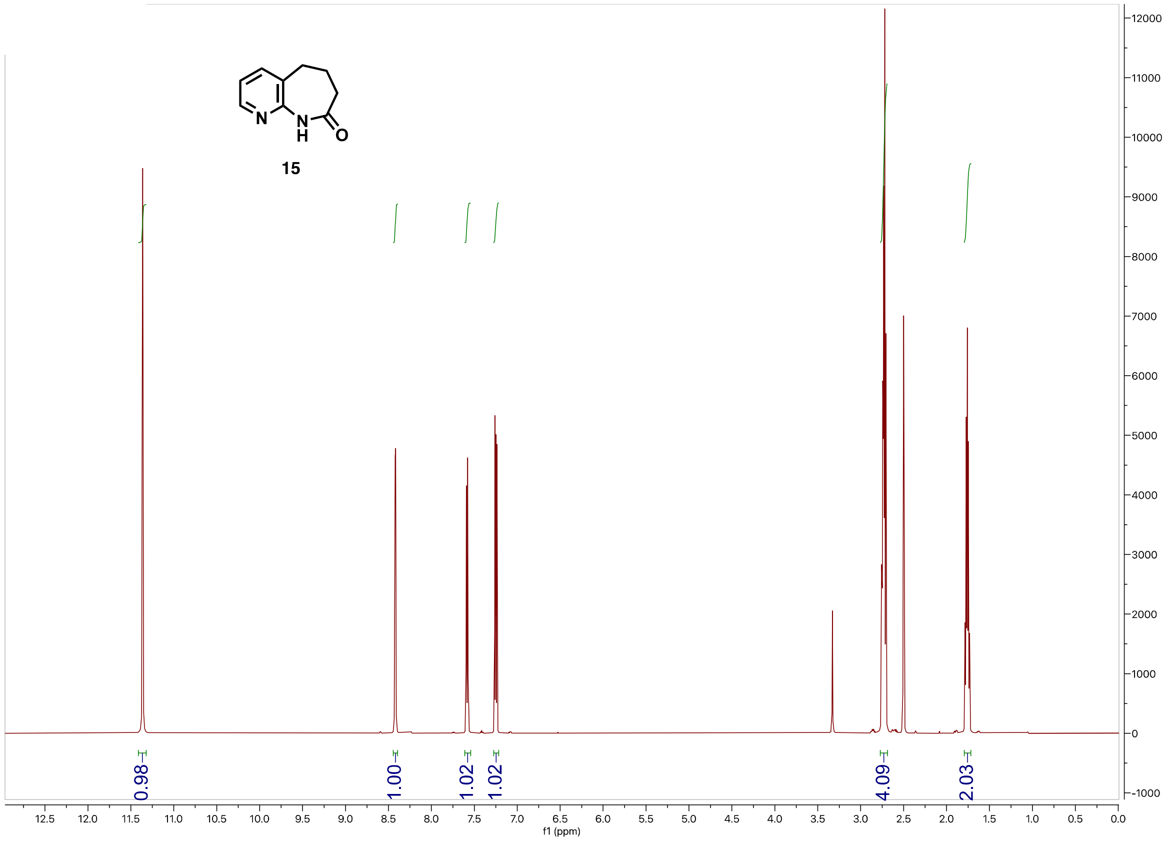

^{13}C NMR

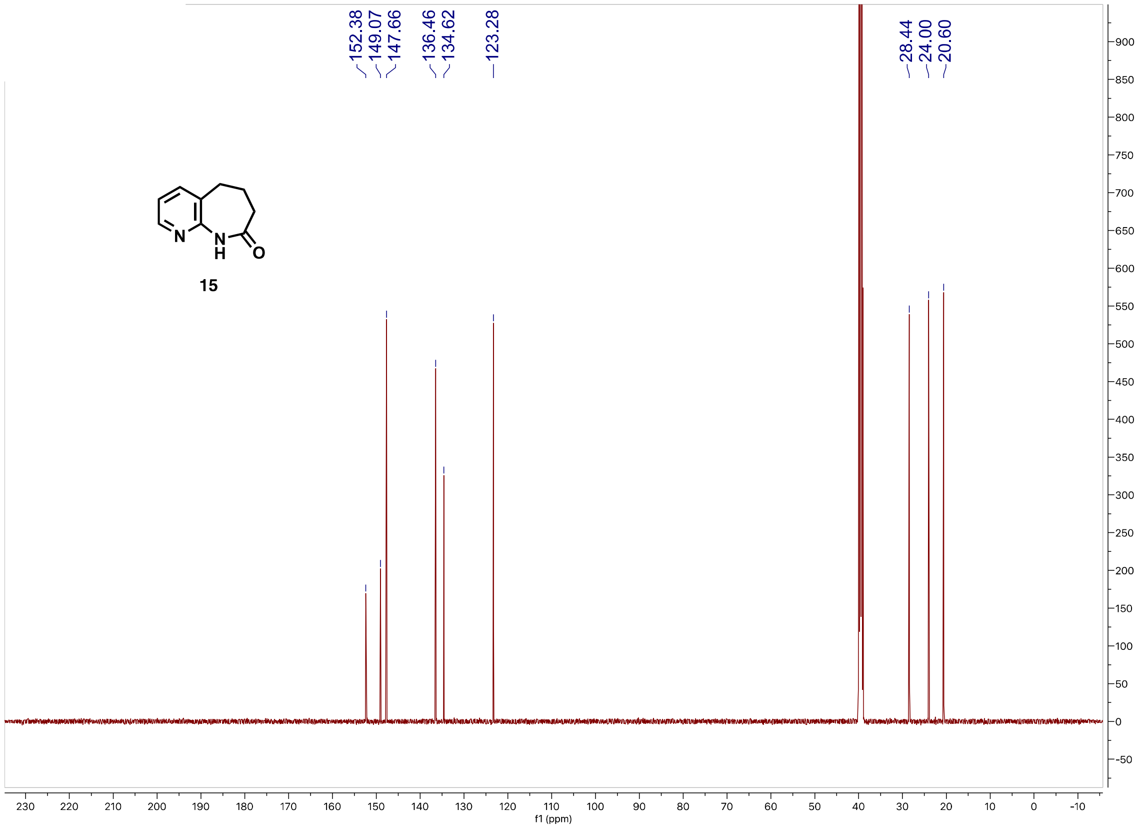

^{1}H NMR

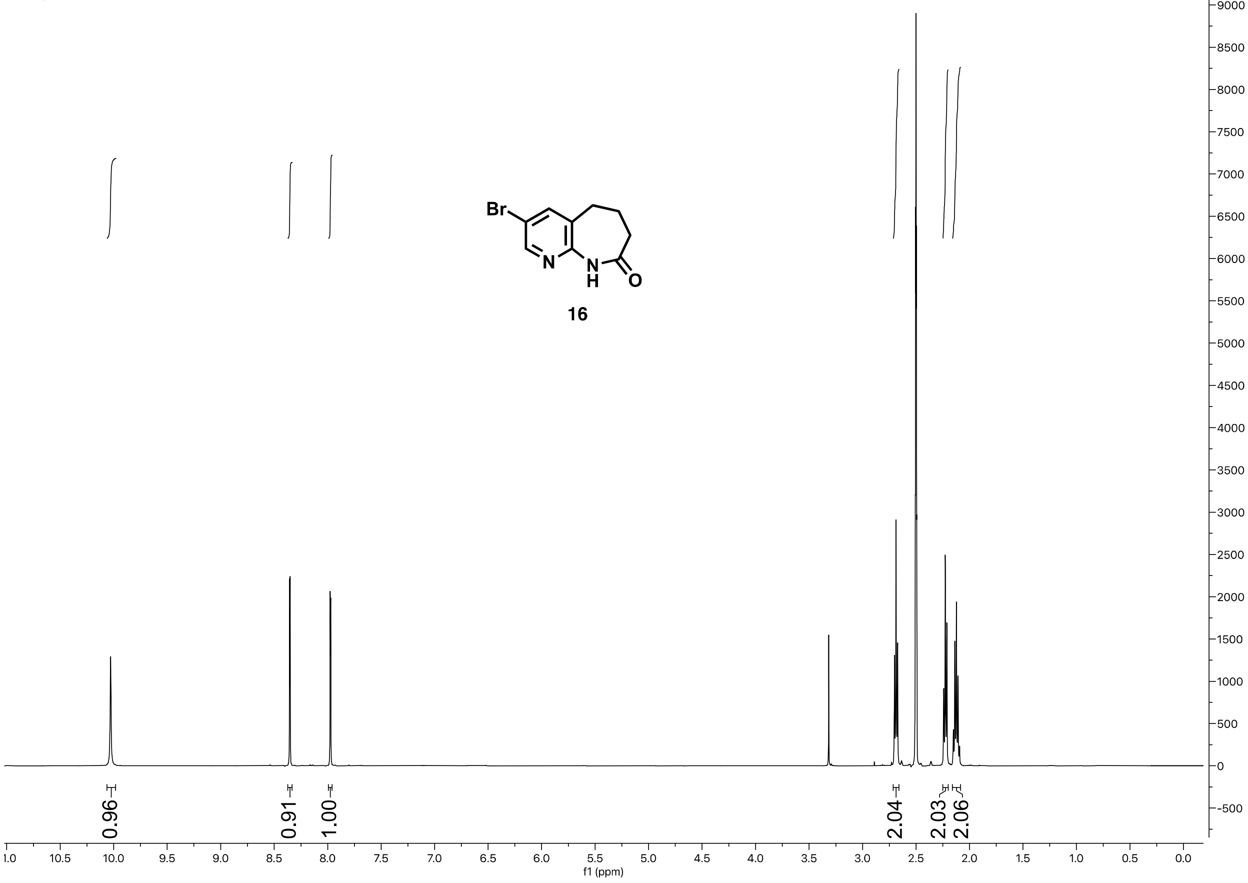

^{13}C NMR

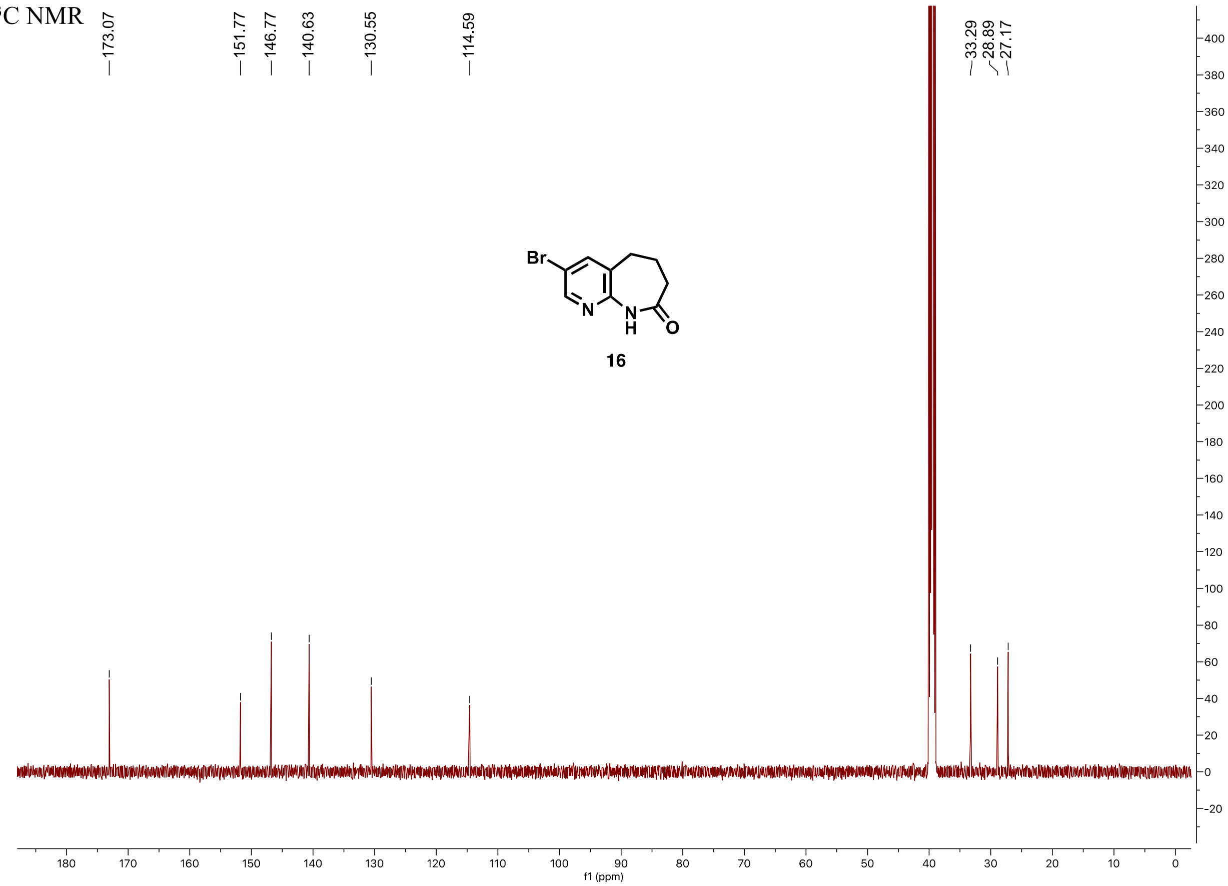

^{1}H NMR

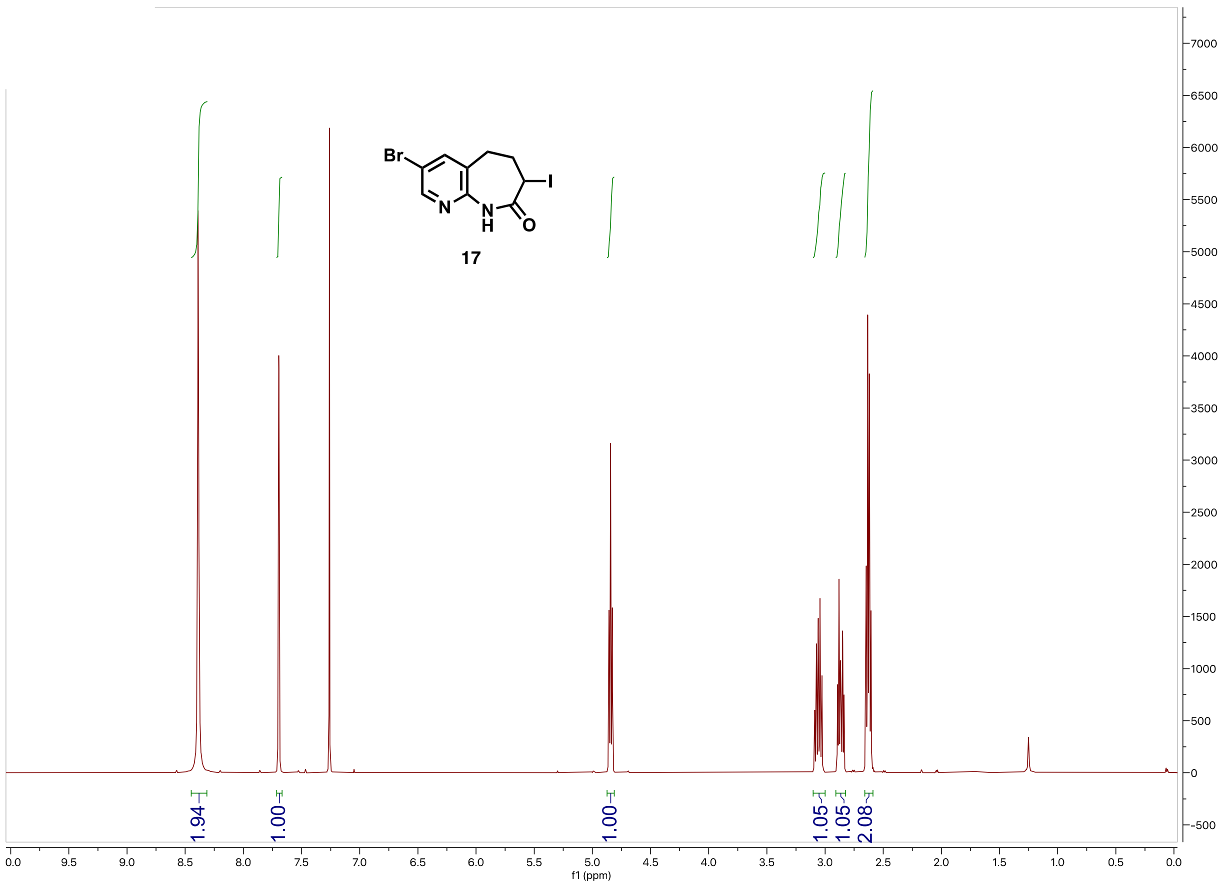

^{13}C NMR

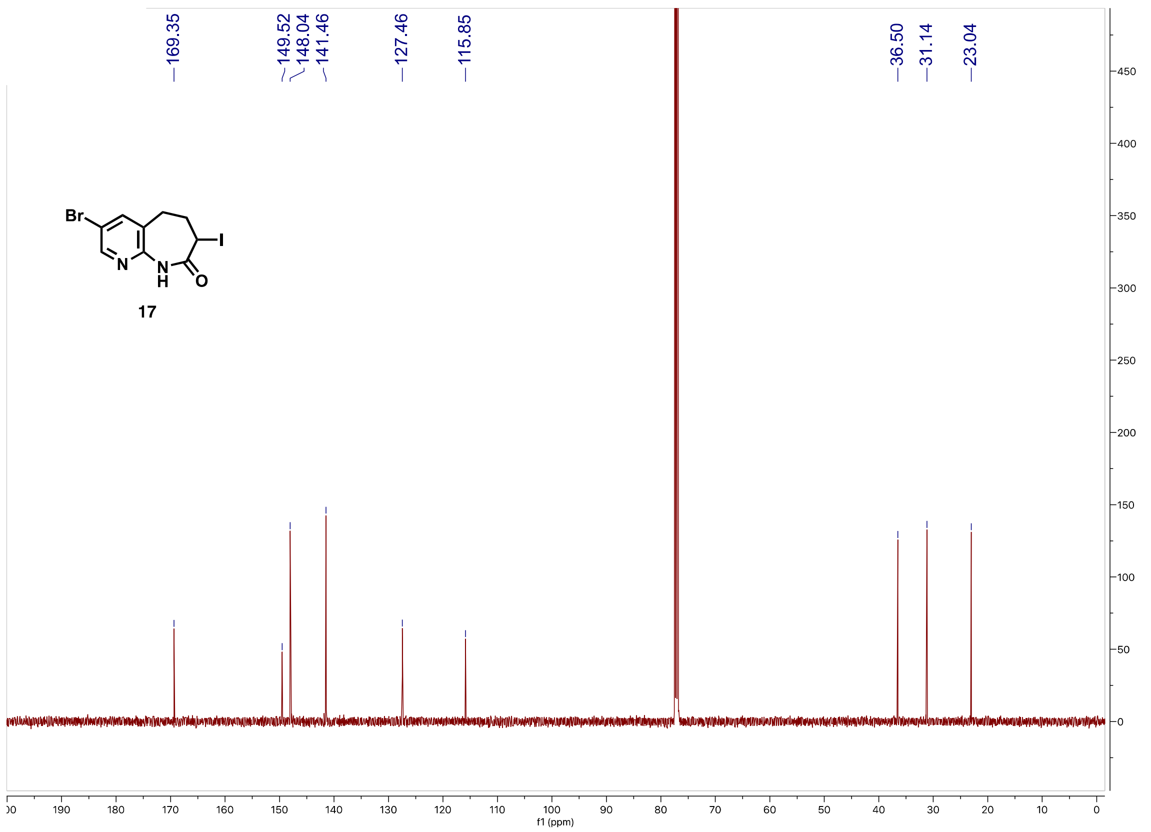

^{1}H NMR

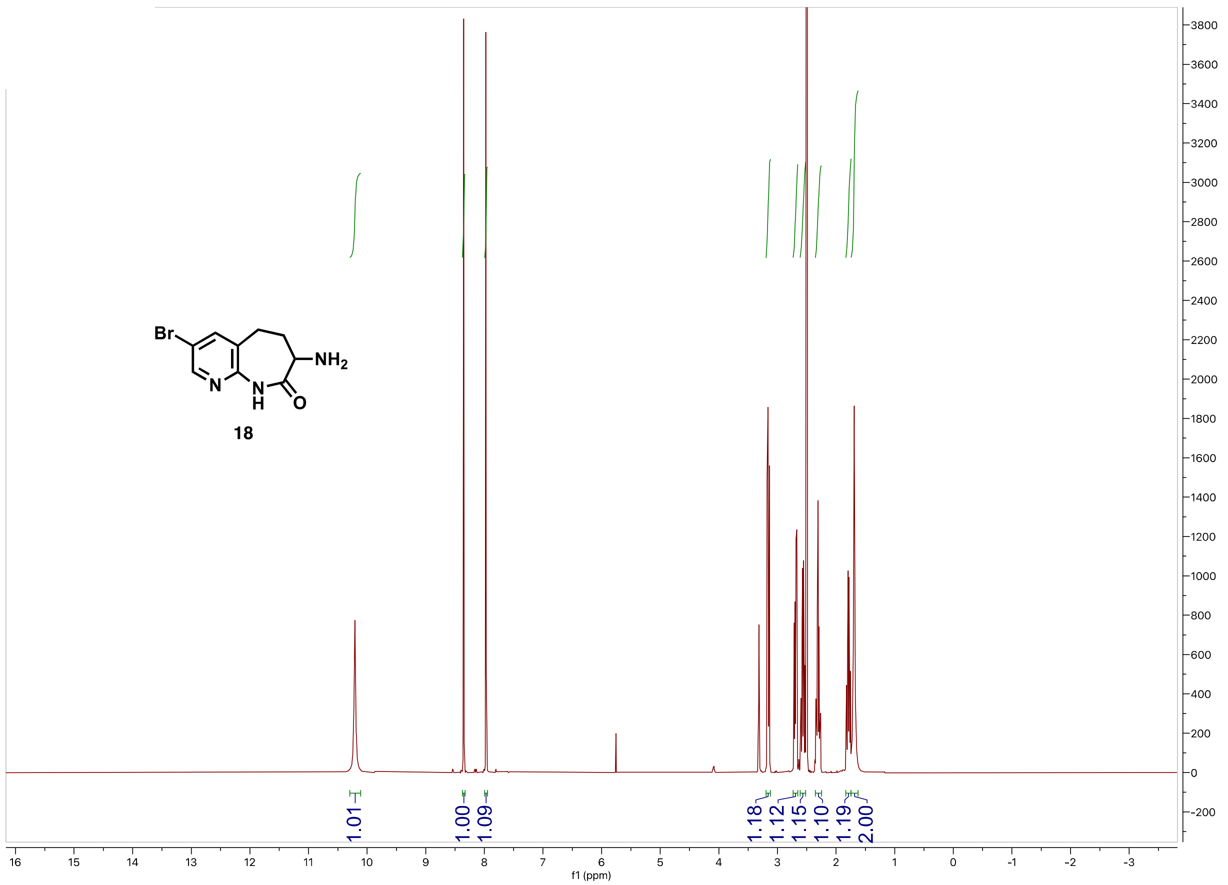

^{13}C NMR

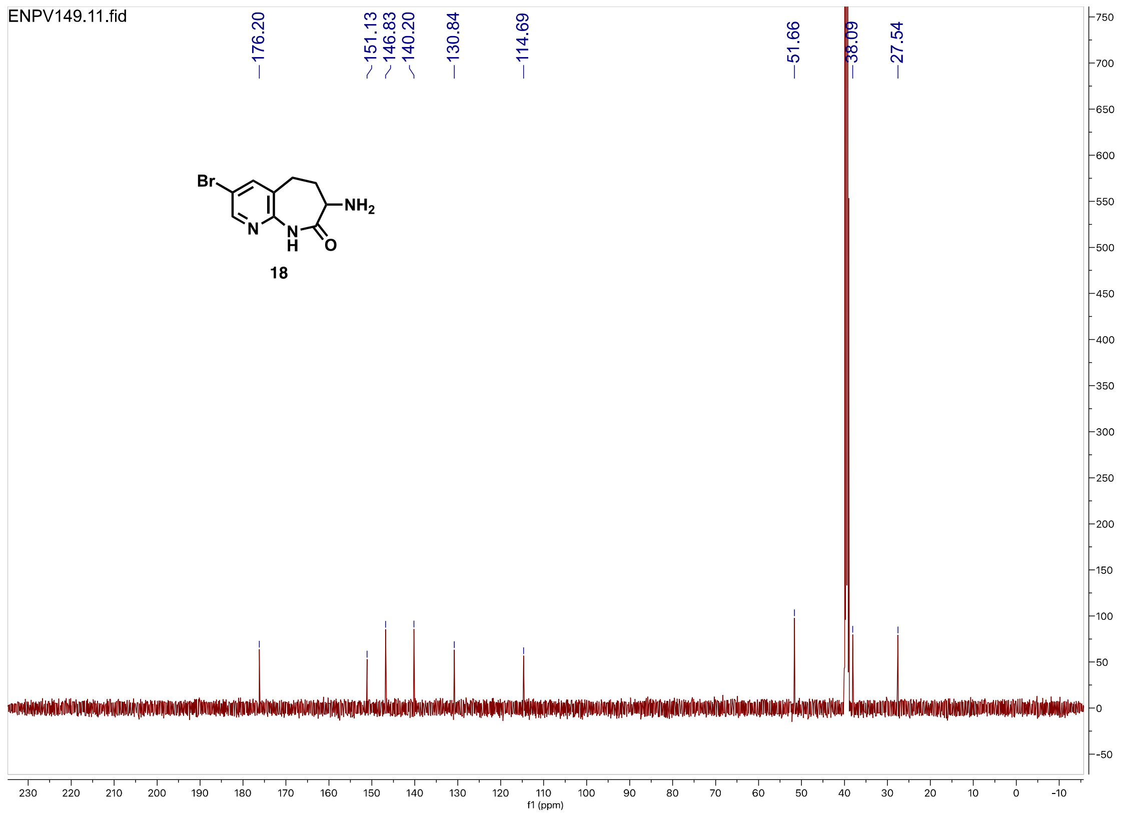

^{1}H NMR

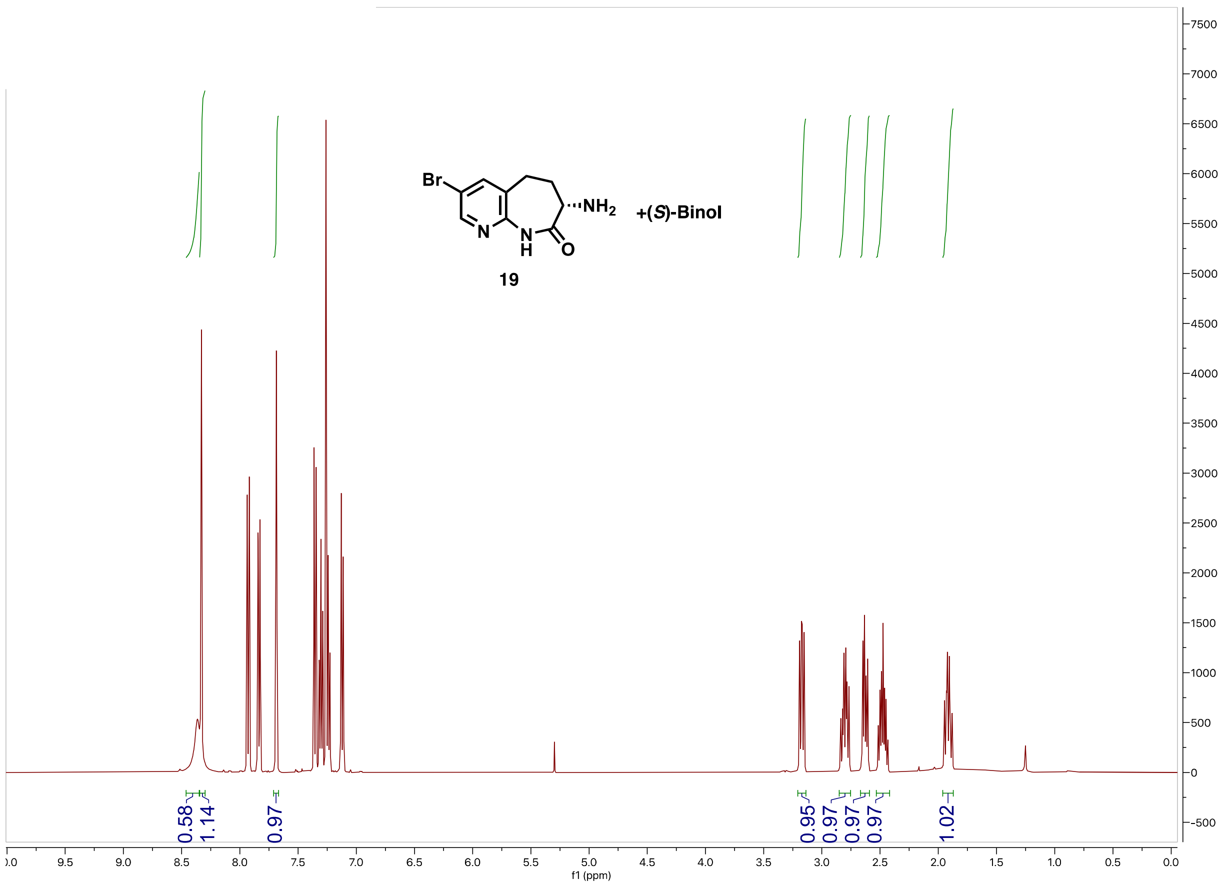

^{13}C NMR

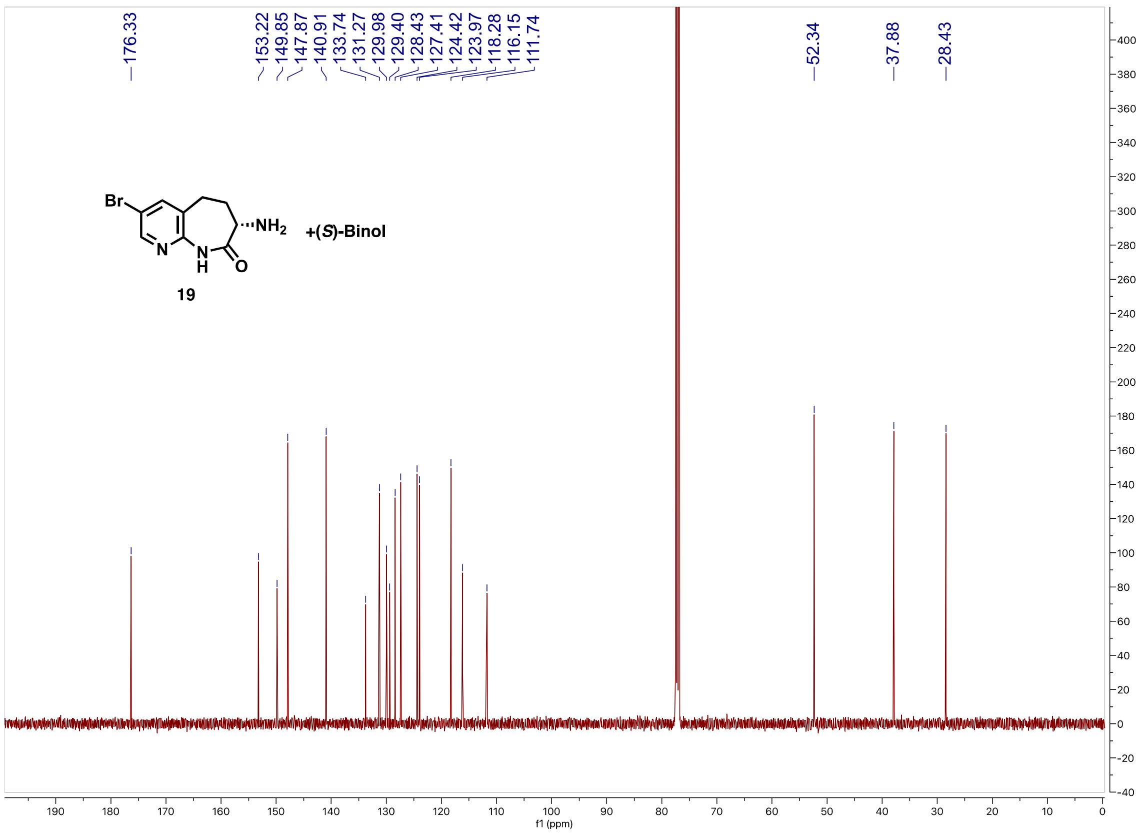

^{1}H NMR

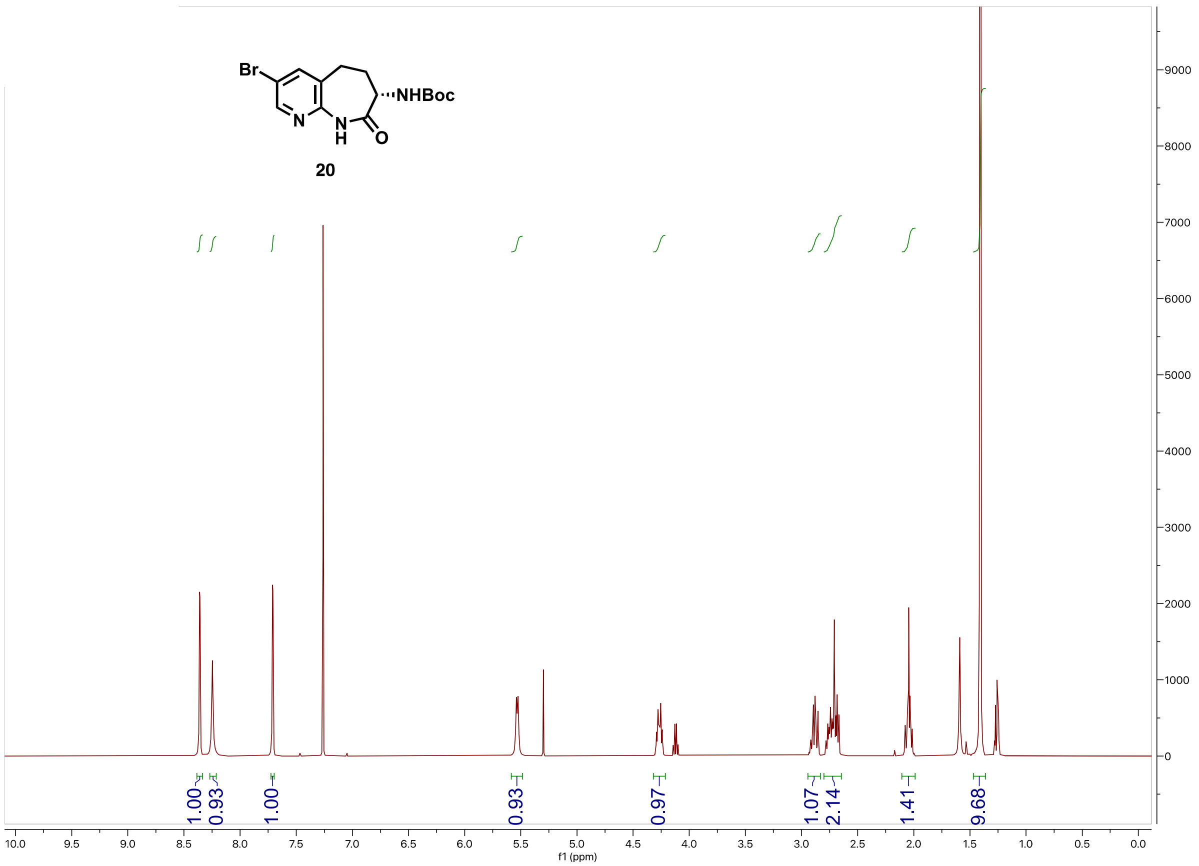

^{13}C NMR

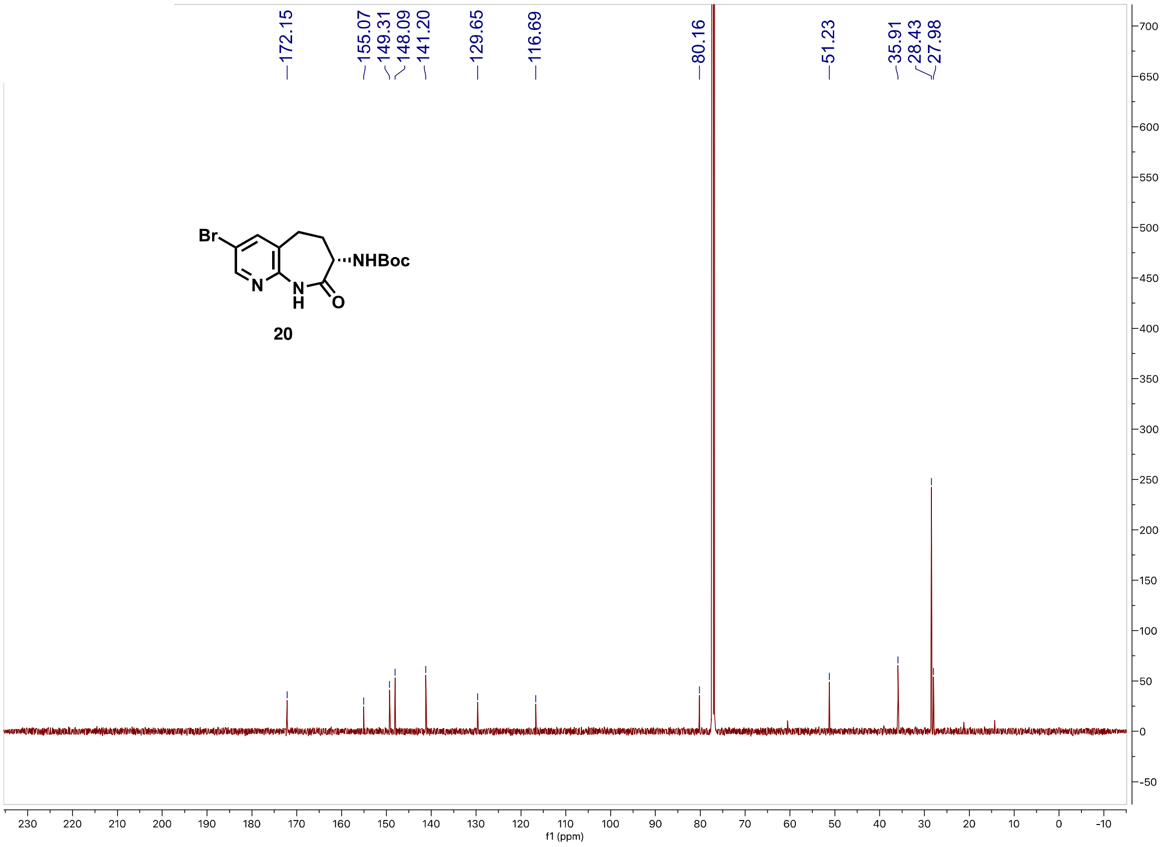

^{1}H NMR

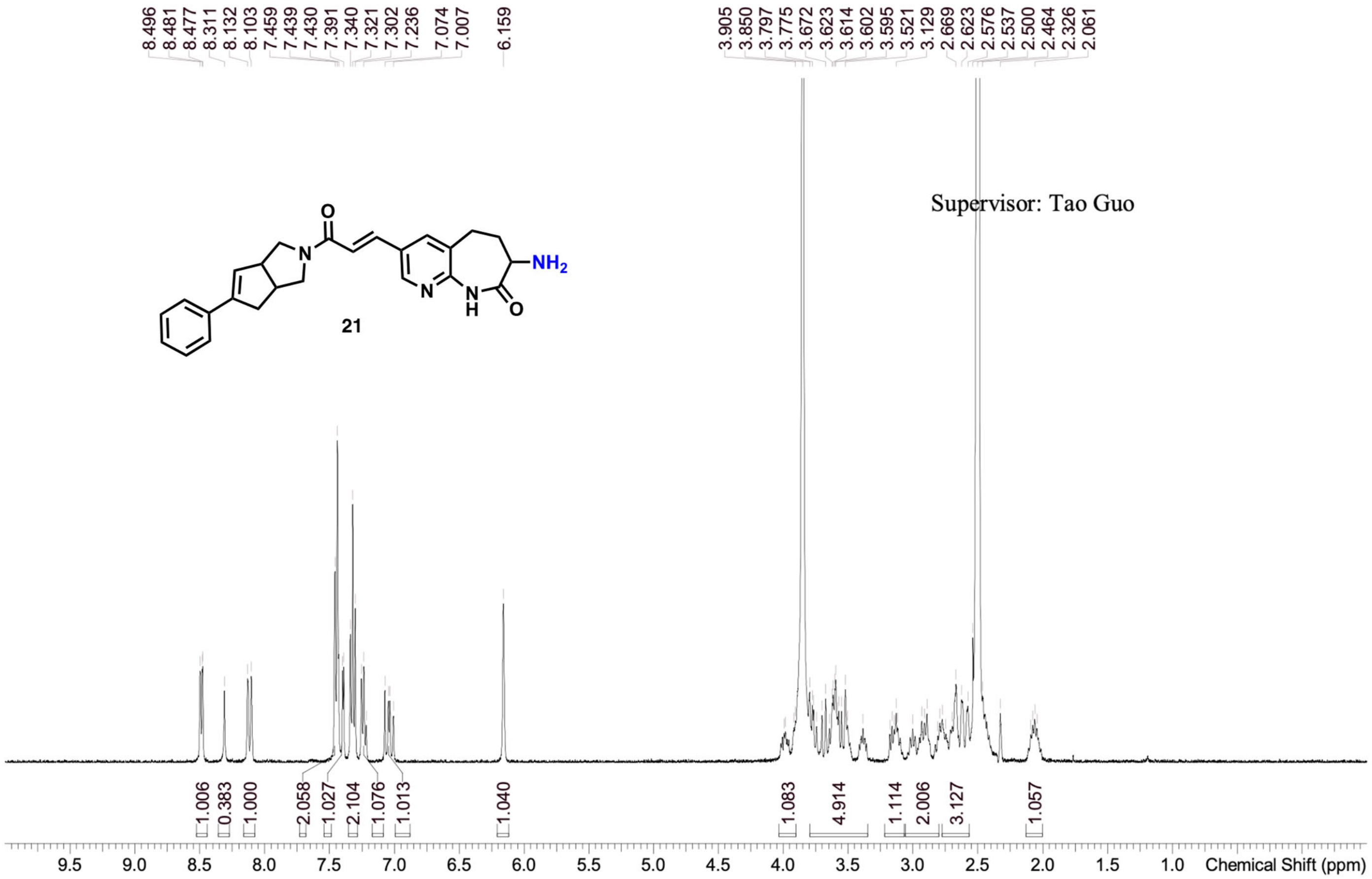

^{13}C NMR

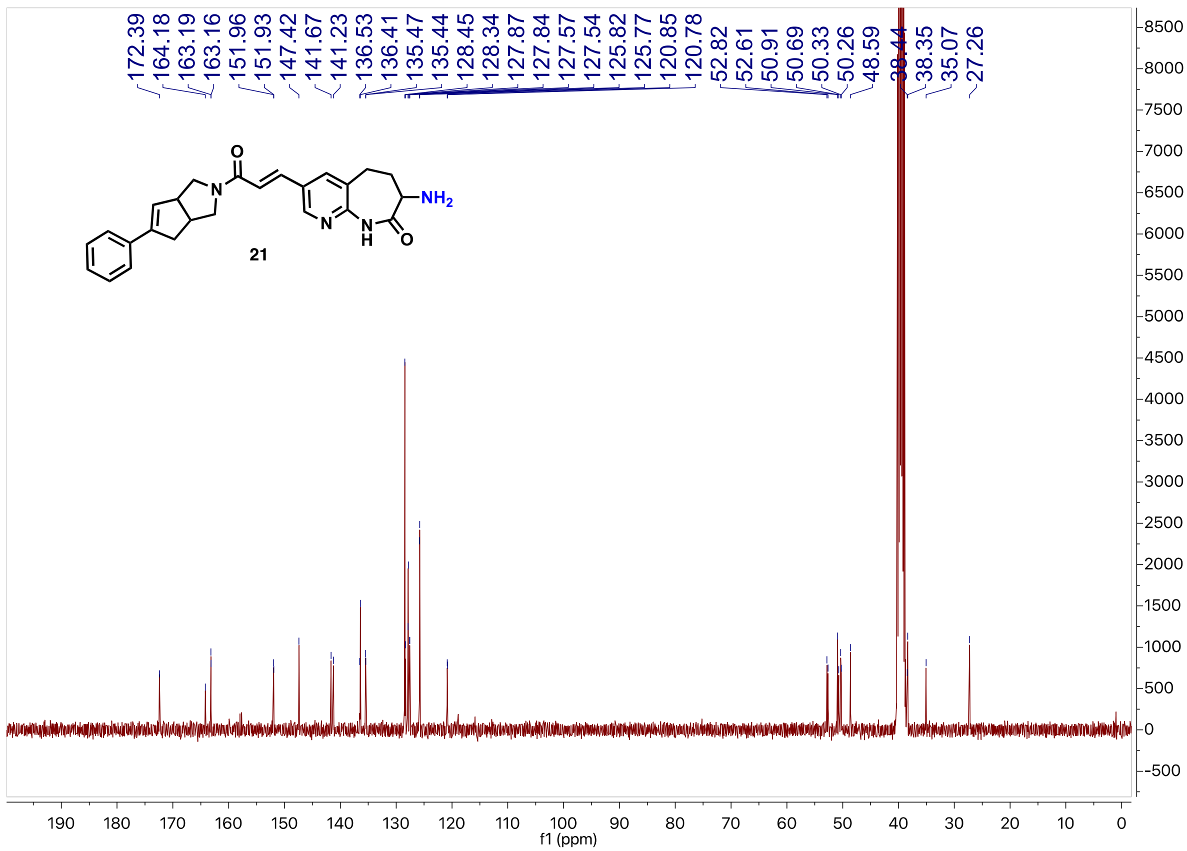

^{1}H NMR

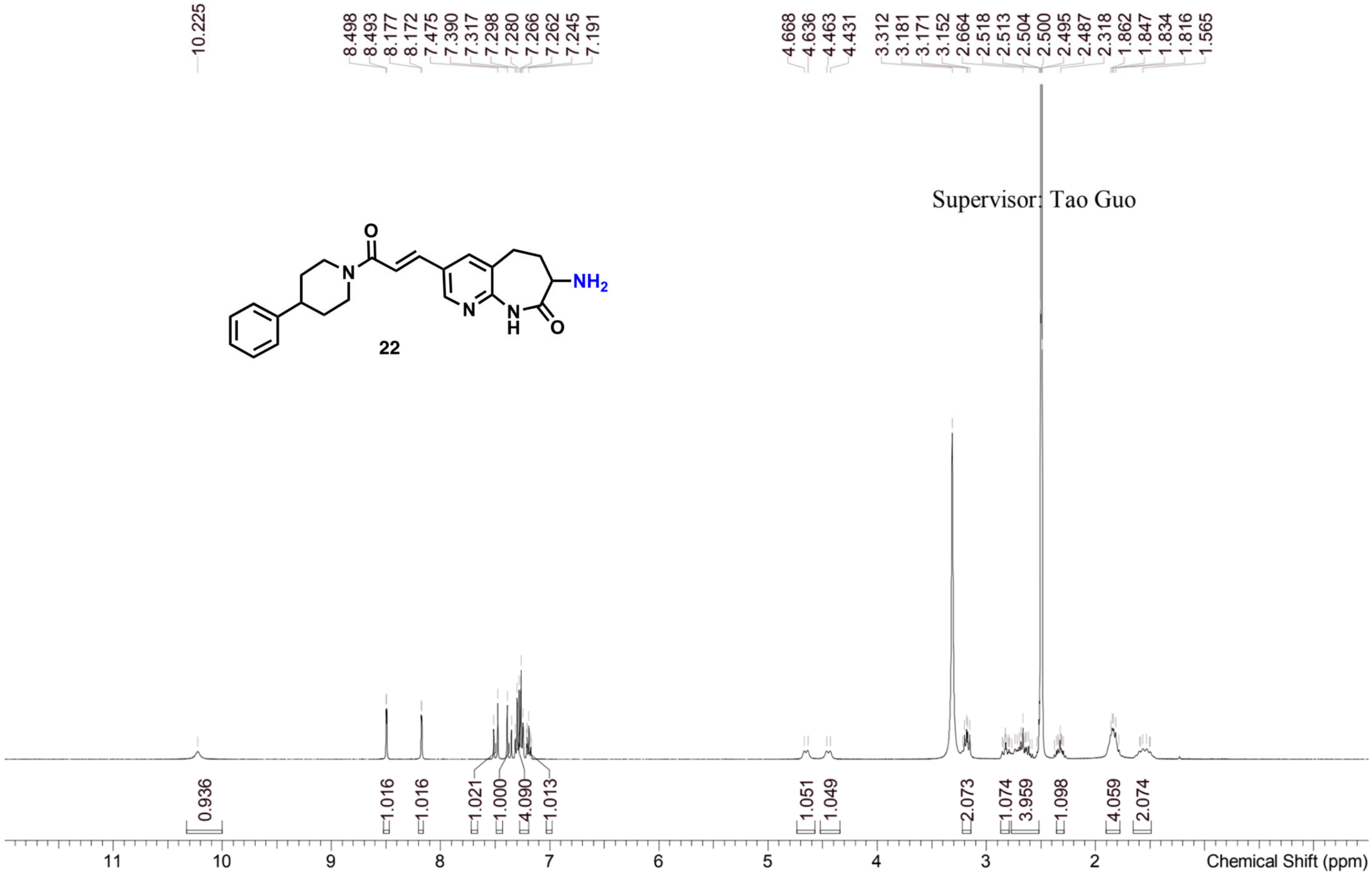

^{13}C NMR

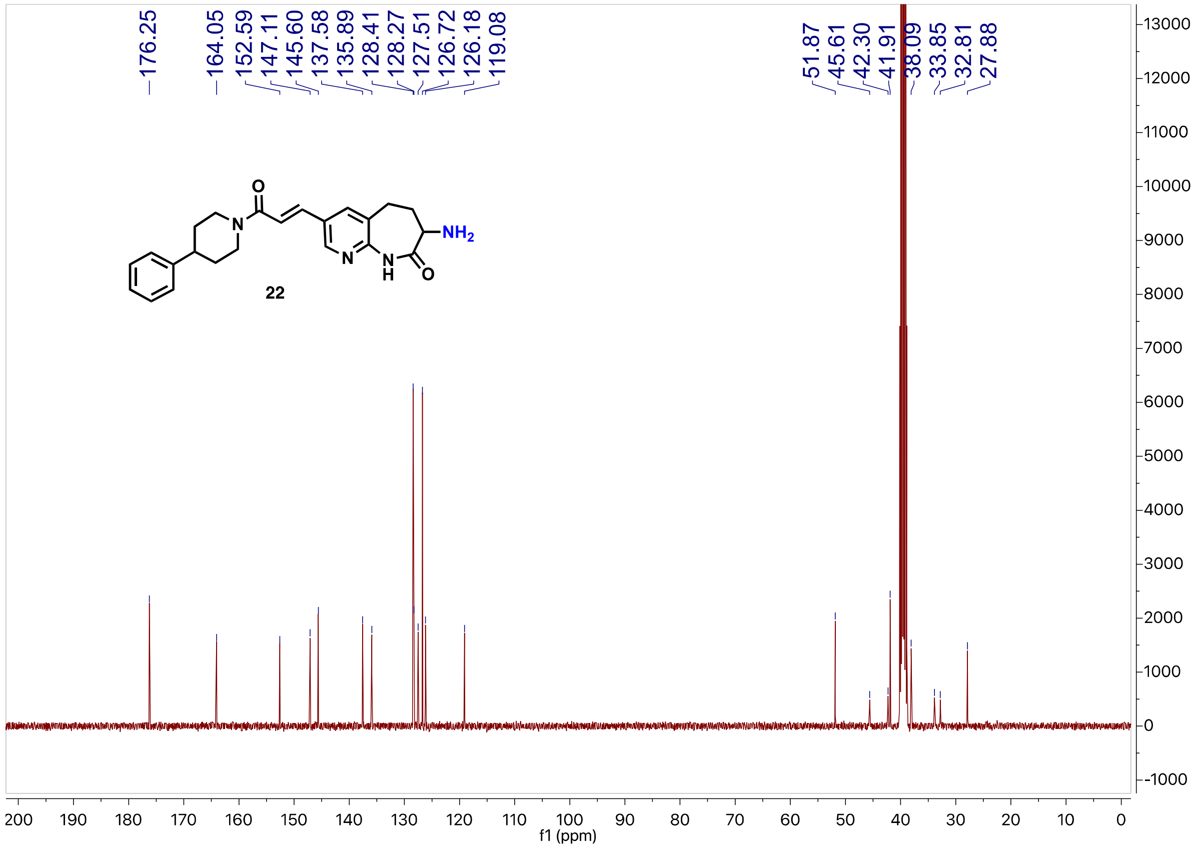

LCMS

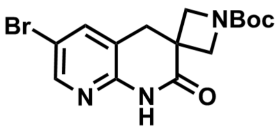

23

Chromatogram

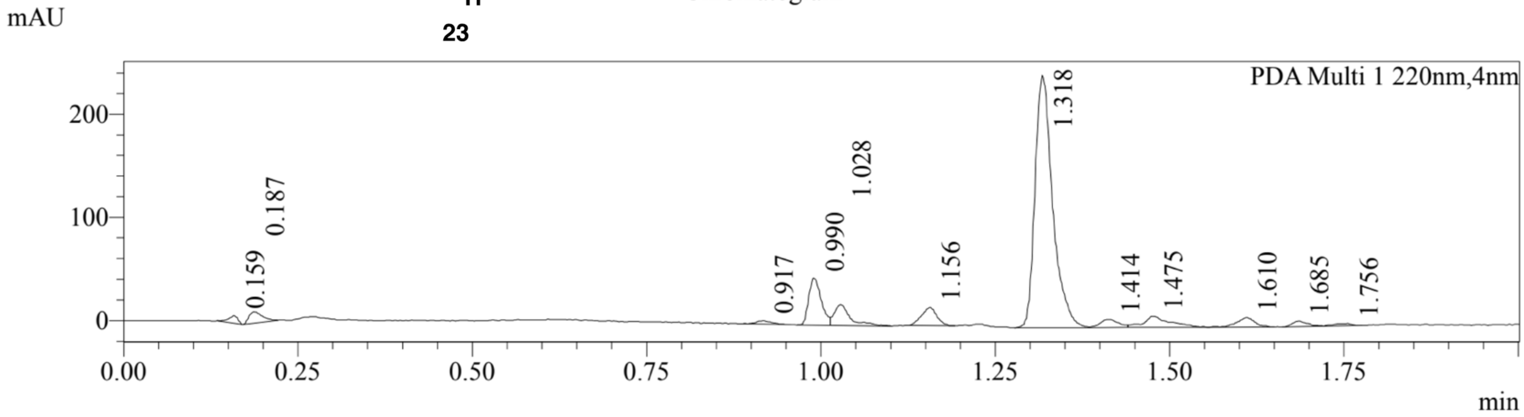

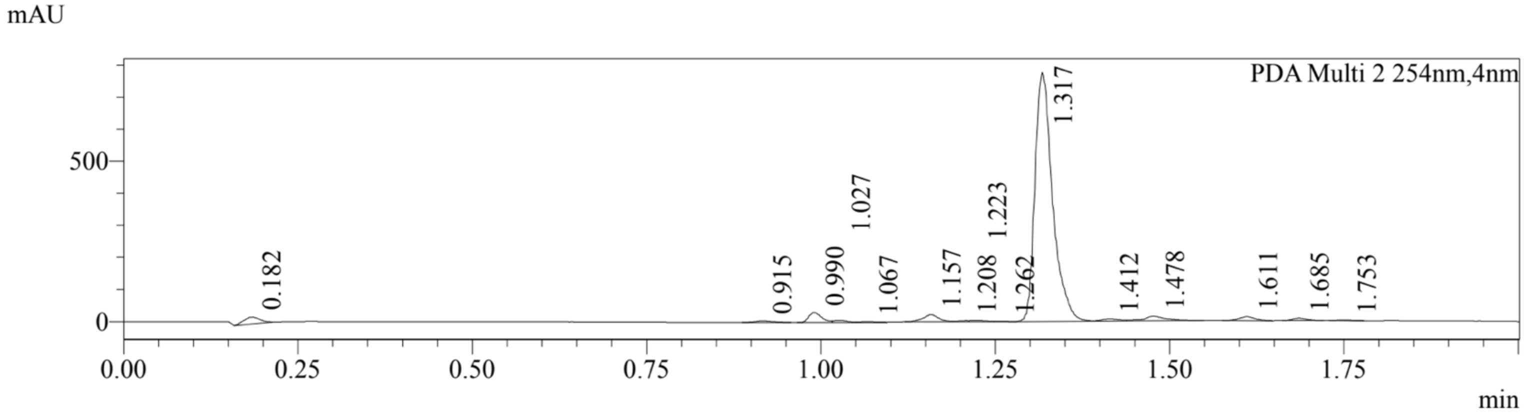

1 PDA Multi 1 / 220nm,4nm
2 PDA Multi 2 / 254nm,4nm

LCMS

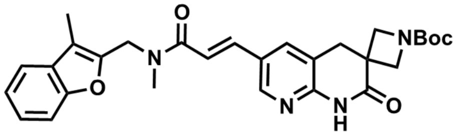

24

Chromatogram

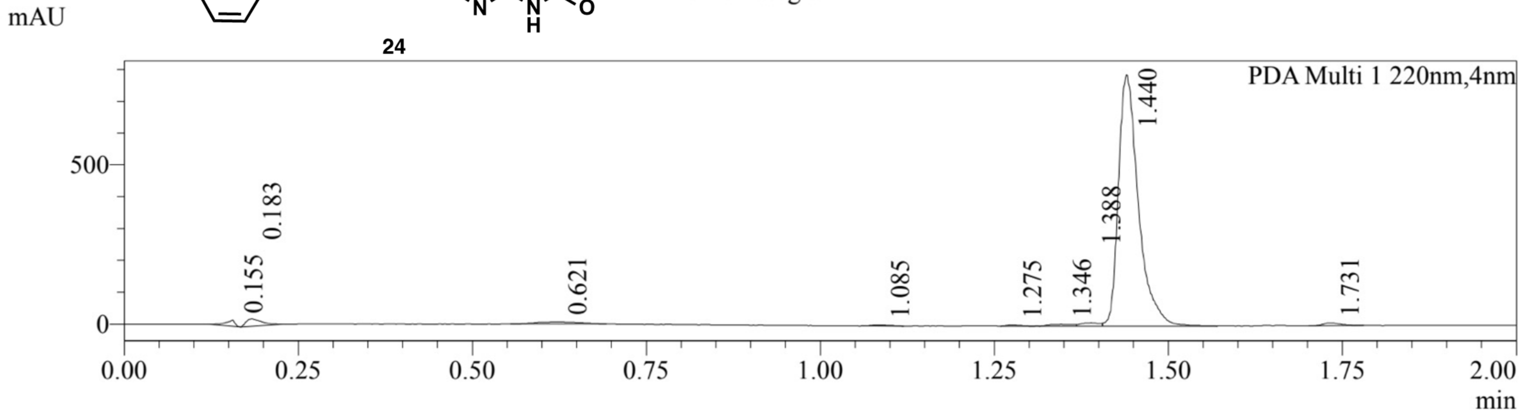

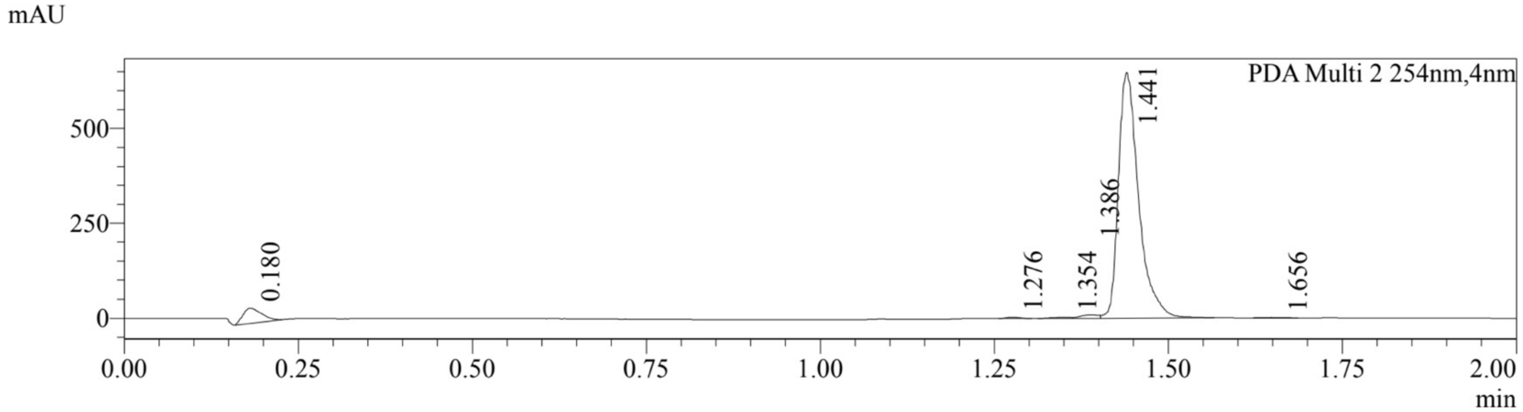

1 PDA Multi 1 / 220nm,4nm
2 PDA Multi 2 / 254nm,4nm

^{1}H NMR

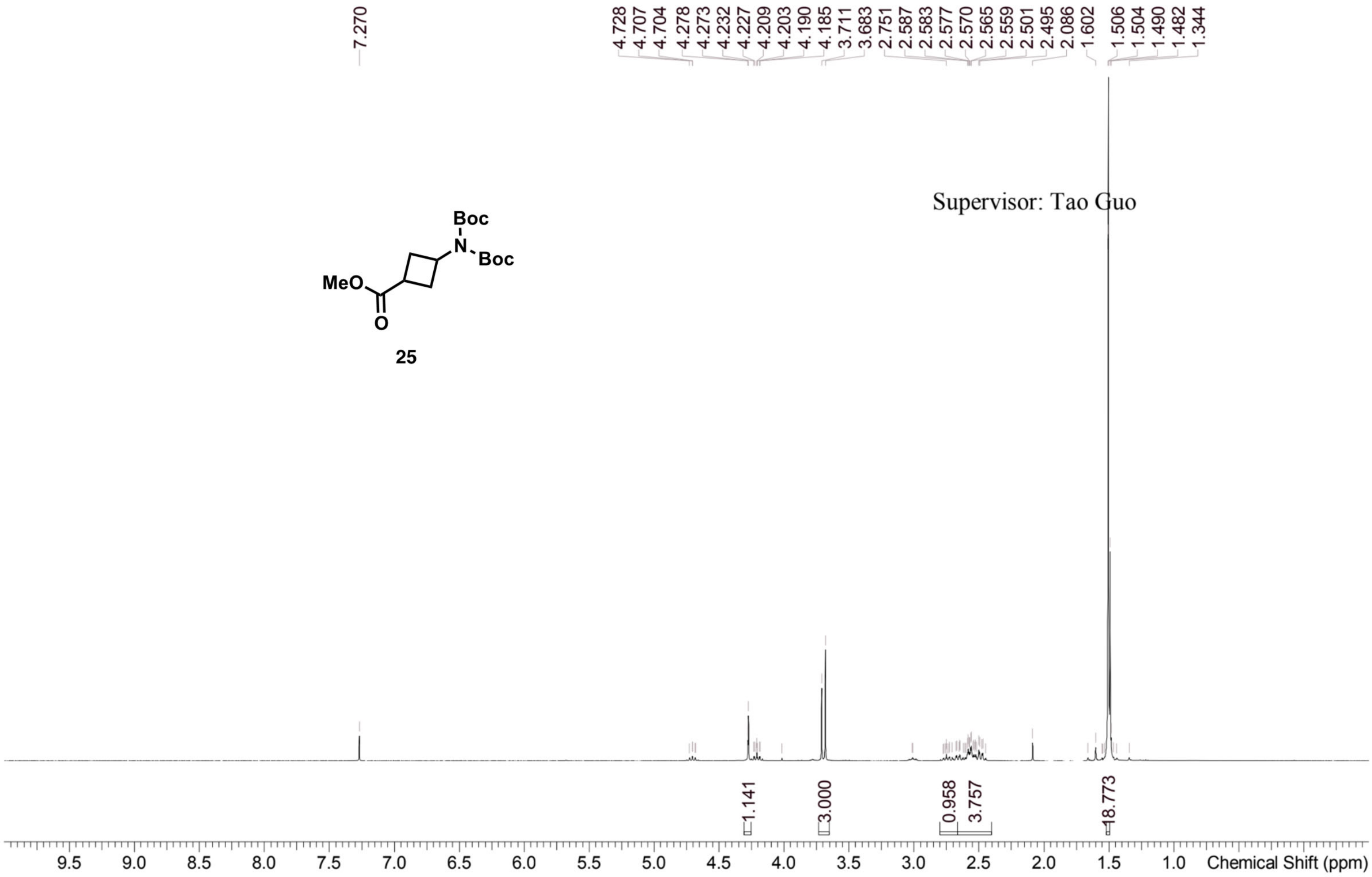

7.270
4.728 4.707 4.704 4.278 4.273 4.232 4.227 4.209 4.203 4.190 4.185 3.711 3.683 2.751 2.587 2.583 2.577 2.570 2.565 2.559 2.501 2.495 2.086 1.602 1.506 1.504 1.490 1.482 1.344
Boc
N
Boc
MeO
O
25
Supervisor: Tao Guo
1.141
3.000
0.958
3.757
18.773
9.5 9.0 8.5 8.0 7.5 7.0 6.5 6.0 5.5 5.0 4.5 4.0 3.5 3.0 2.5 2.0 1.5 1.0 Chemical Shift (ppm)

LCMS

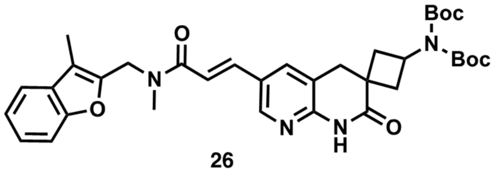

26

Chromatogram

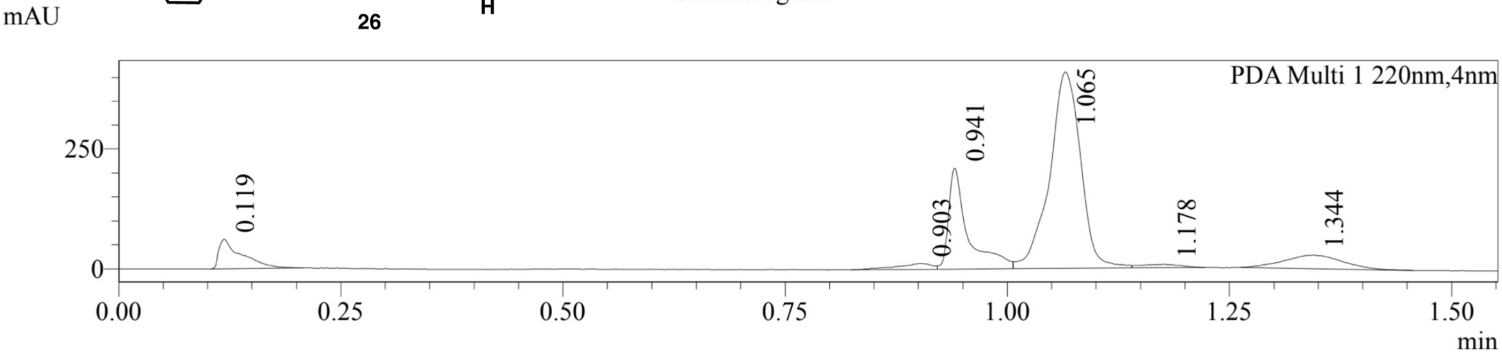

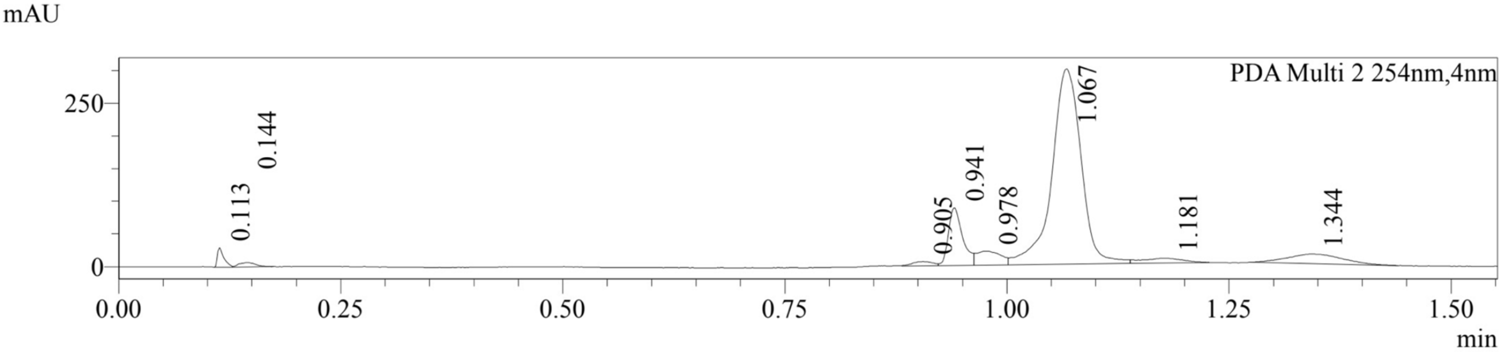

1 PDA Multi 1 / 220nm,4nm
2 PDA Multi 2 / 254nm,4nm

^{1}H NMR

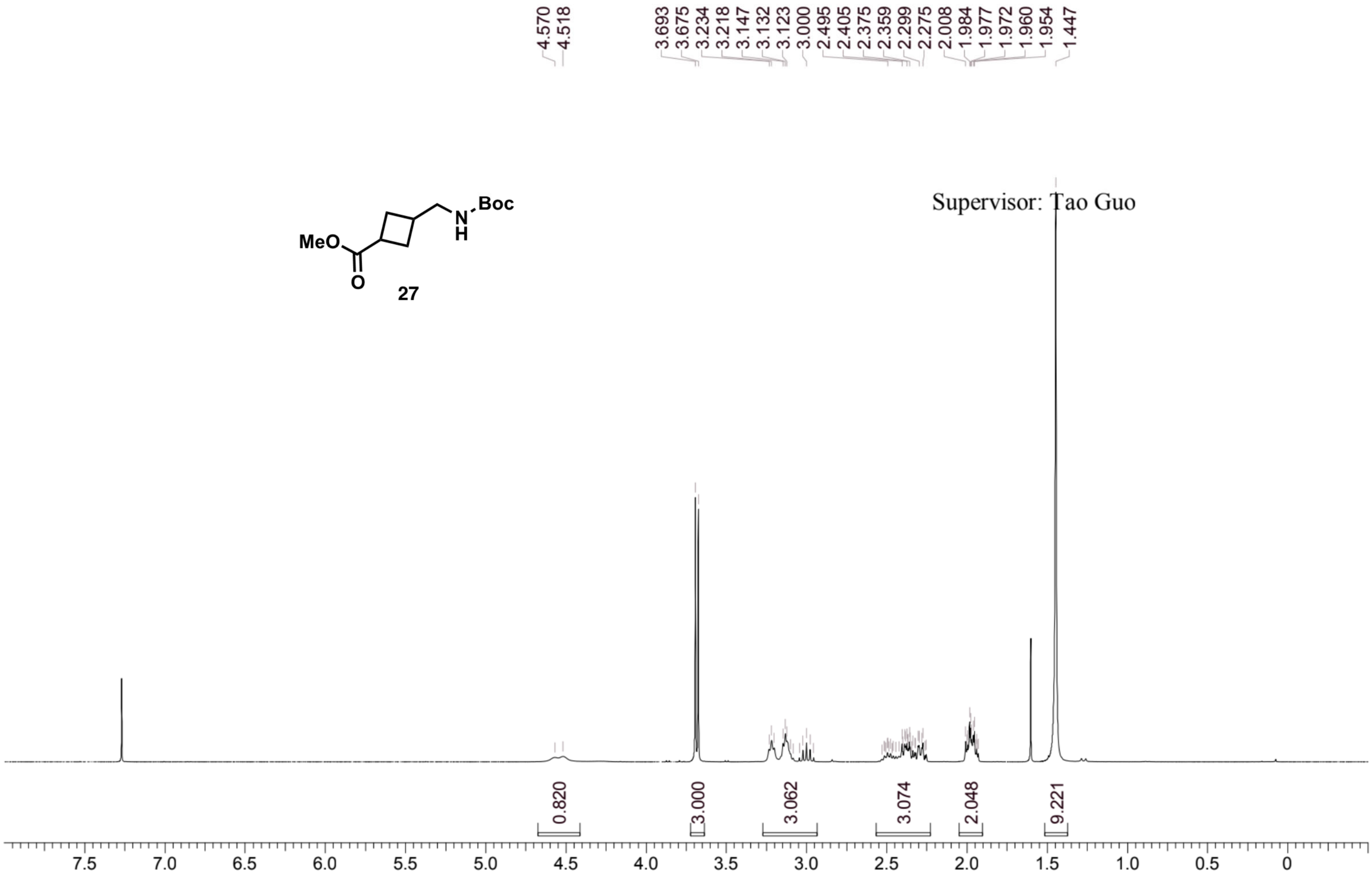

4.570
4.518
3.693
3.675
3.234
3.218
3.147
3.132
3.123
3.000
2.495
2.405
2.375
2.359
2.299
2.275
2.008
1.984
1.977
1.972
1.960
1.954
1.447
MeO
O
N
H
Boc
27
Supervisor: Tao Guo
0.820
3.000
3.062
3.074
2.048
9.221
7.5
7.0
6.5
6.0
5.5
5.0
4.5
4.0
3.5
3.0
2.5
2.0
1.5
1.0
0.5
0

^{1}H NMR

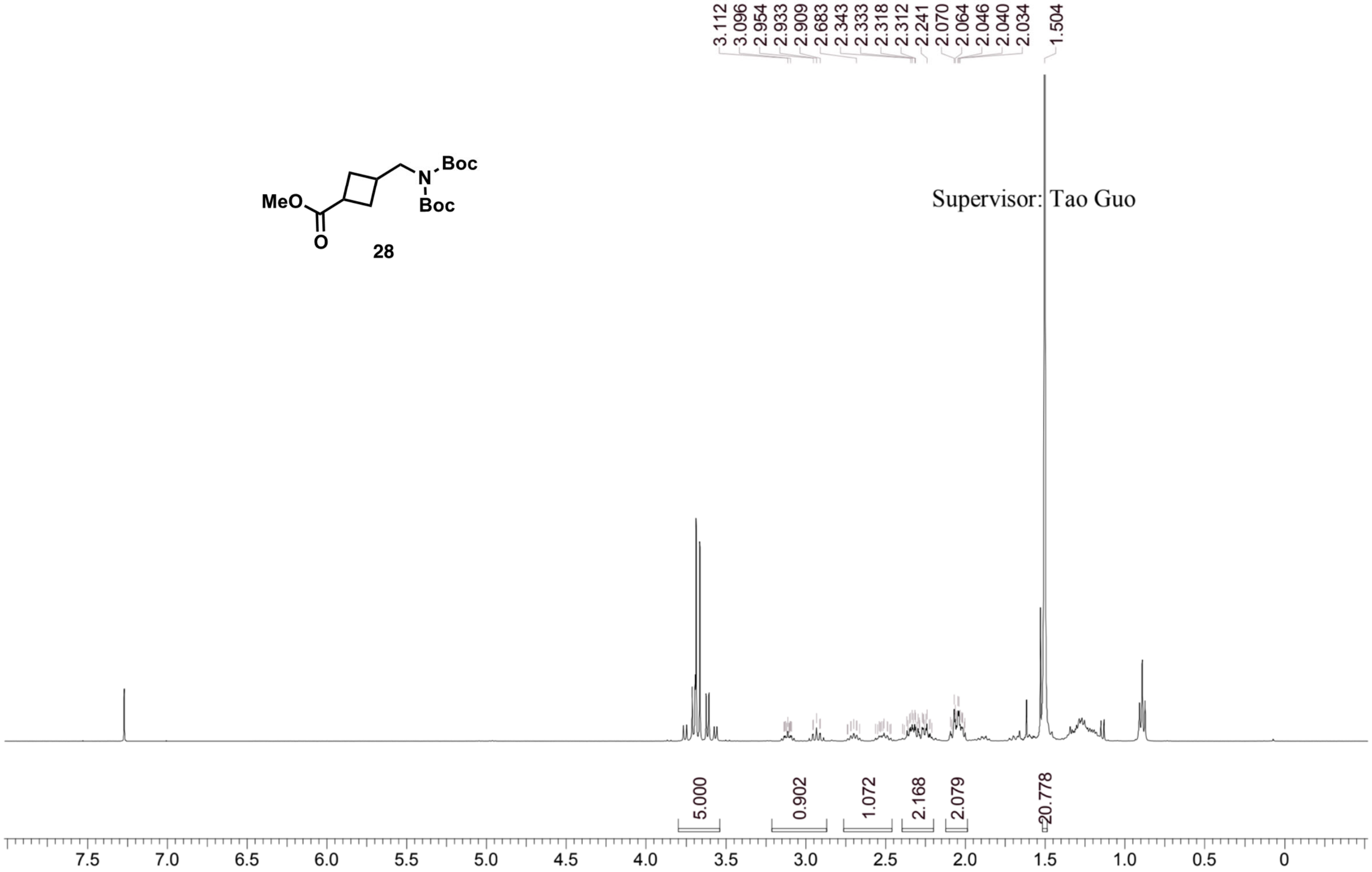
3.112
3.096
2.954
2.933
2.909
2.683
2.343
2.333
2.318
2.312
2.241
2.070
2.064
2.046
2.040
2.034
1.504
Supervisor: Tao Guo
5.000
0.902
1.072
2.168
2.079
20.778
7.5
7.0
6.5
6.0
5.5
5.0
4.5
4.0
3.5
3.0
2.5
2.0
1.5
1.0
0.5
0

LCMS

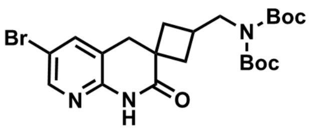

29

Chromatogram

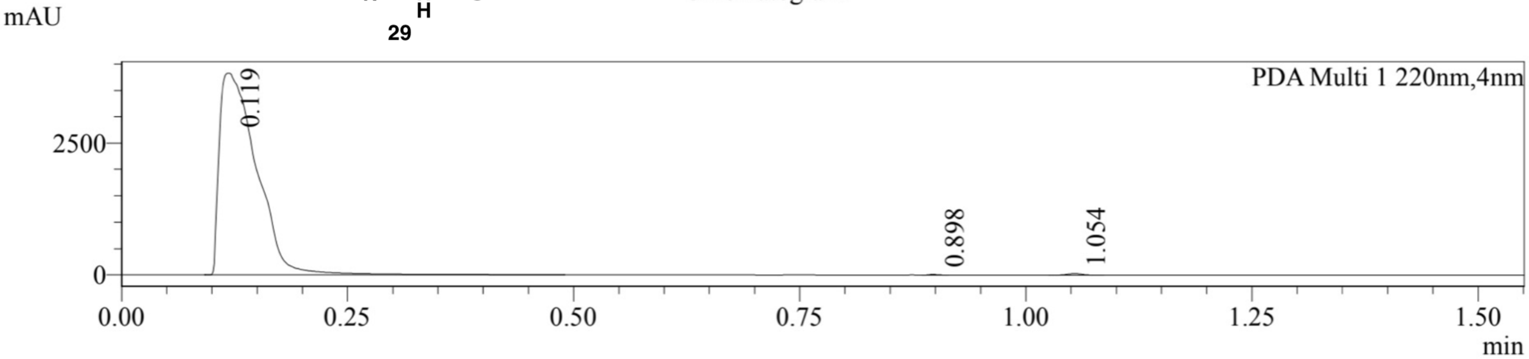

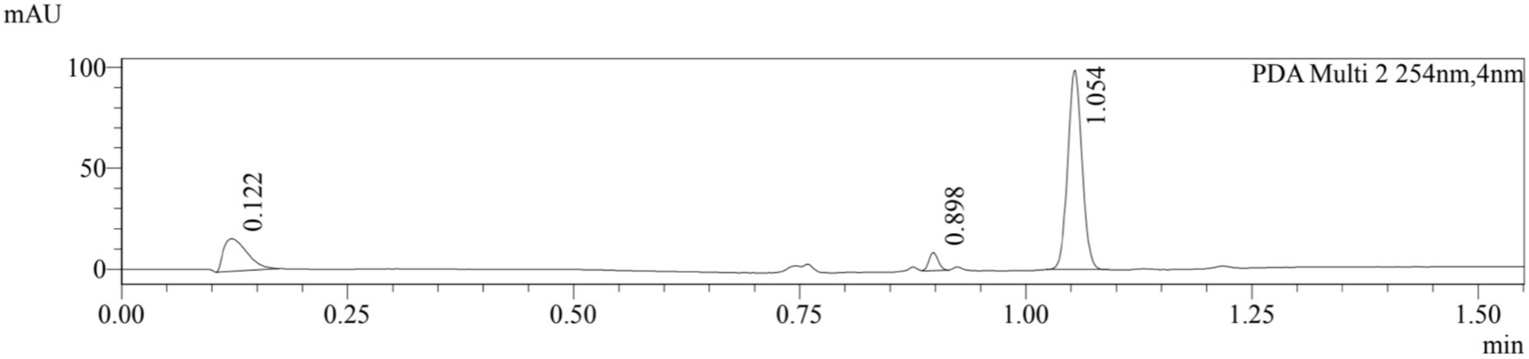

LCMS

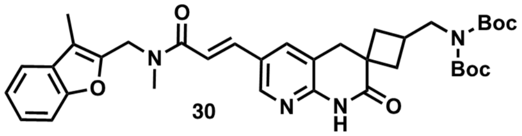

Chromatogram

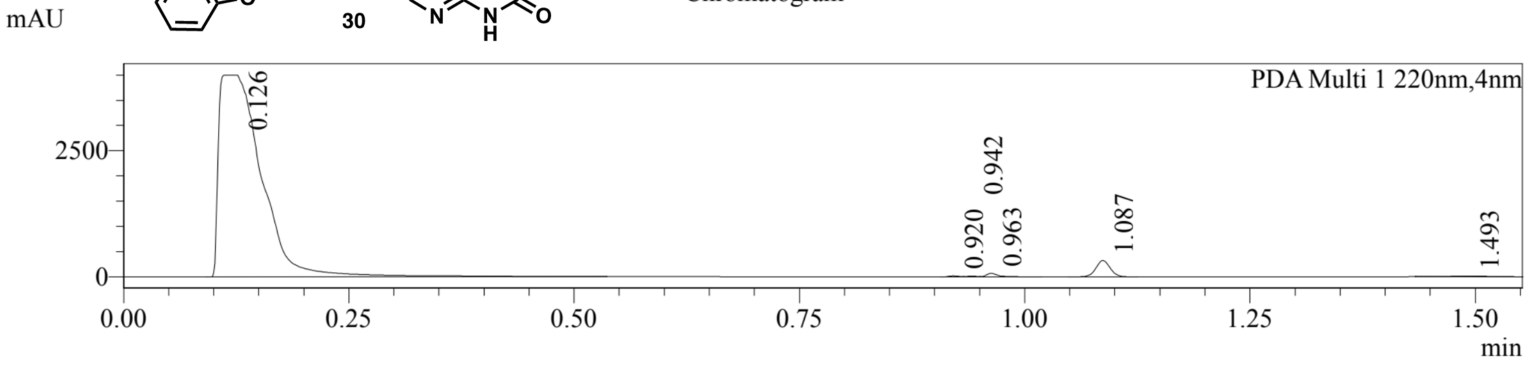

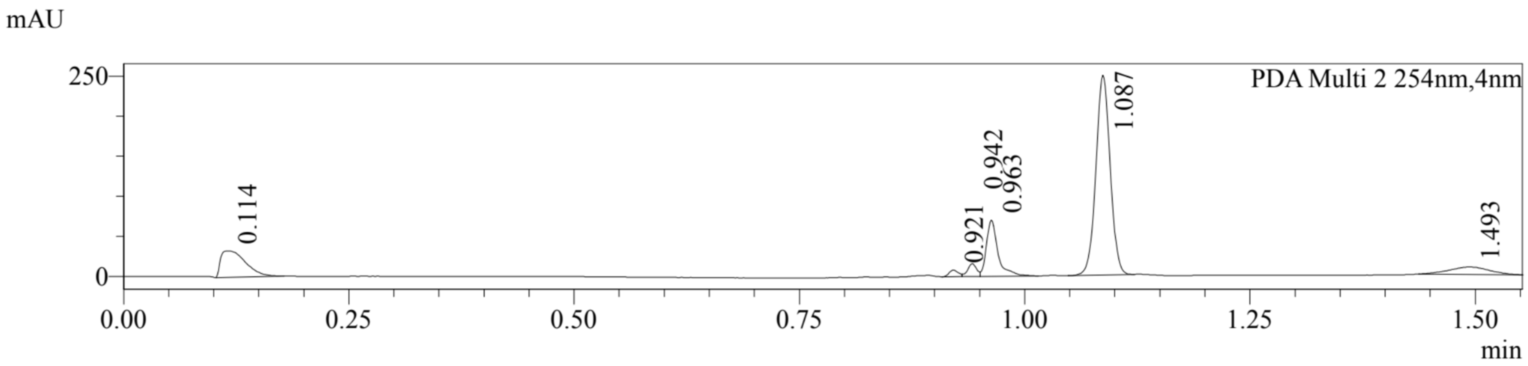

^{1}H NMR

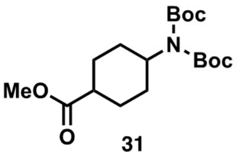

31

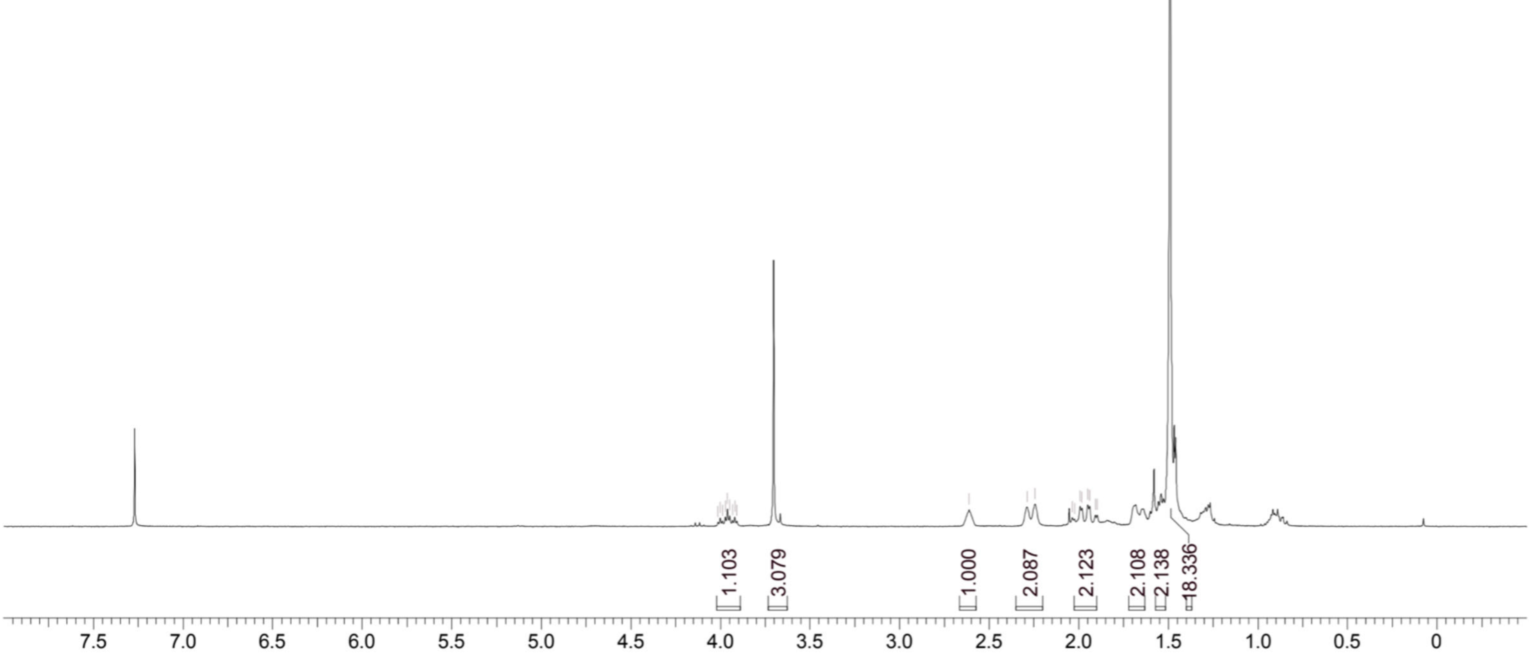
4.014
4.002
3.990
3.973
3.961
3.949
3.933
3.921
2.612
2.288
2.245
2.024
1.992
1.982
1.950
1.939
1.908
1.897
Supervisor: Tao Guo
1.103
3.079
1.000
2.087
2.123
2.108
2.138
18.336
7.5
7.0
6.5
6.0
5.5
5.0
4.5
4.0
3.5
3.0
2.5
2.0
1.5
1.0
0.5
0

^{1}H NMR

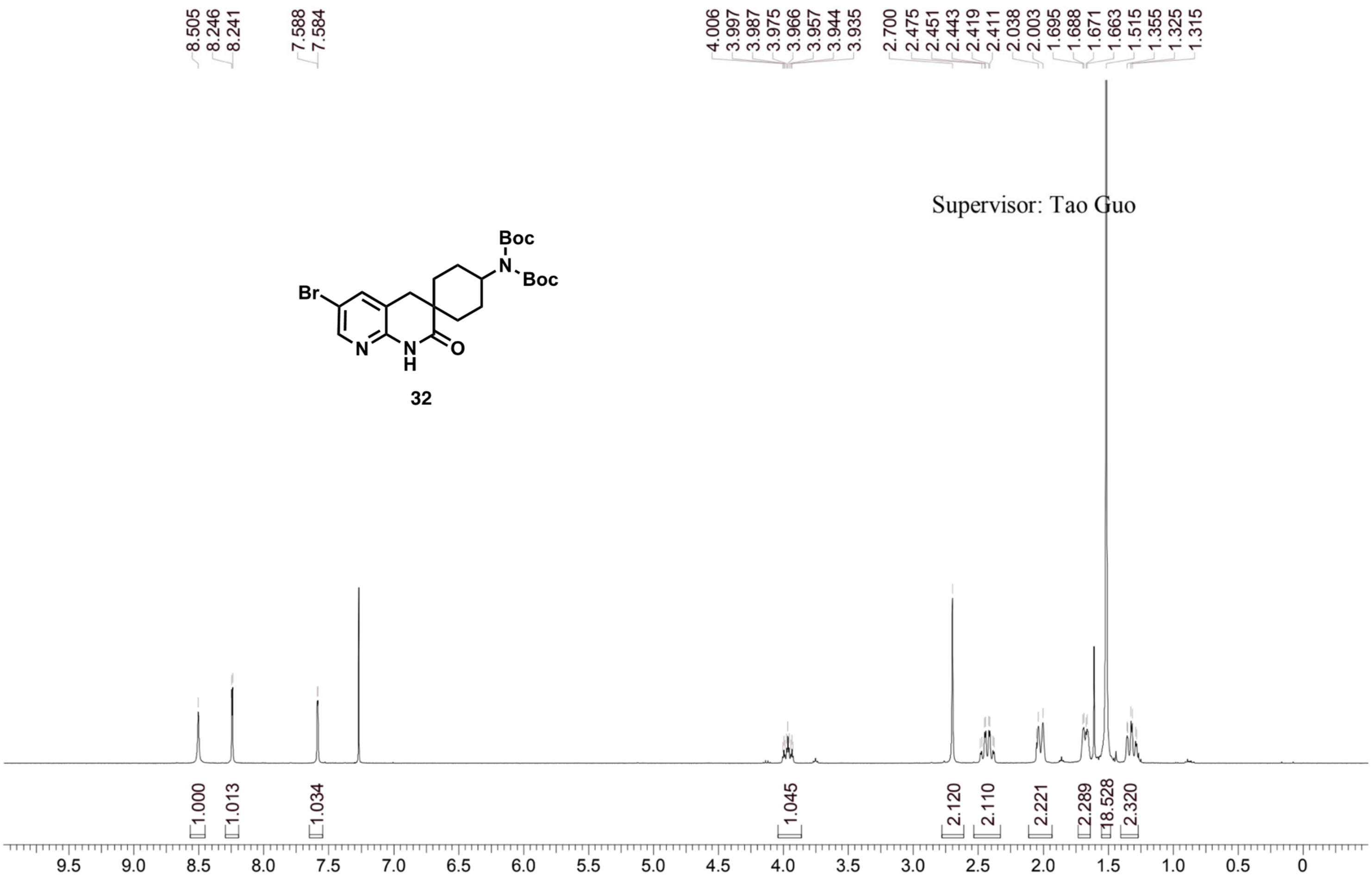
8.505 8.246 8.241 7.588 7.584
4.006 3.997 3.987 3.975 3.966 3.957 3.944 3.935 2.700 2.475 2.451 2.443 2.419 2.411 2.038 2.003 1.695 1.688 1.671 1.663 1.515 1.355 1.325 1.315
Supervisor: Tao Guo
Br
N
H
O
N
Boc
Boc
32
1.000 1.013 1.034 1.045 2.120 2.110 2.221 2.289 18.528 2.320
9.5 9.0 8.5 8.0 7.5 7.0 6.5 6.0 5.5 5.0 4.5 4.0 3.5 3.0 2.5 2.0 1.5 1.0 0.5 0

LCMS

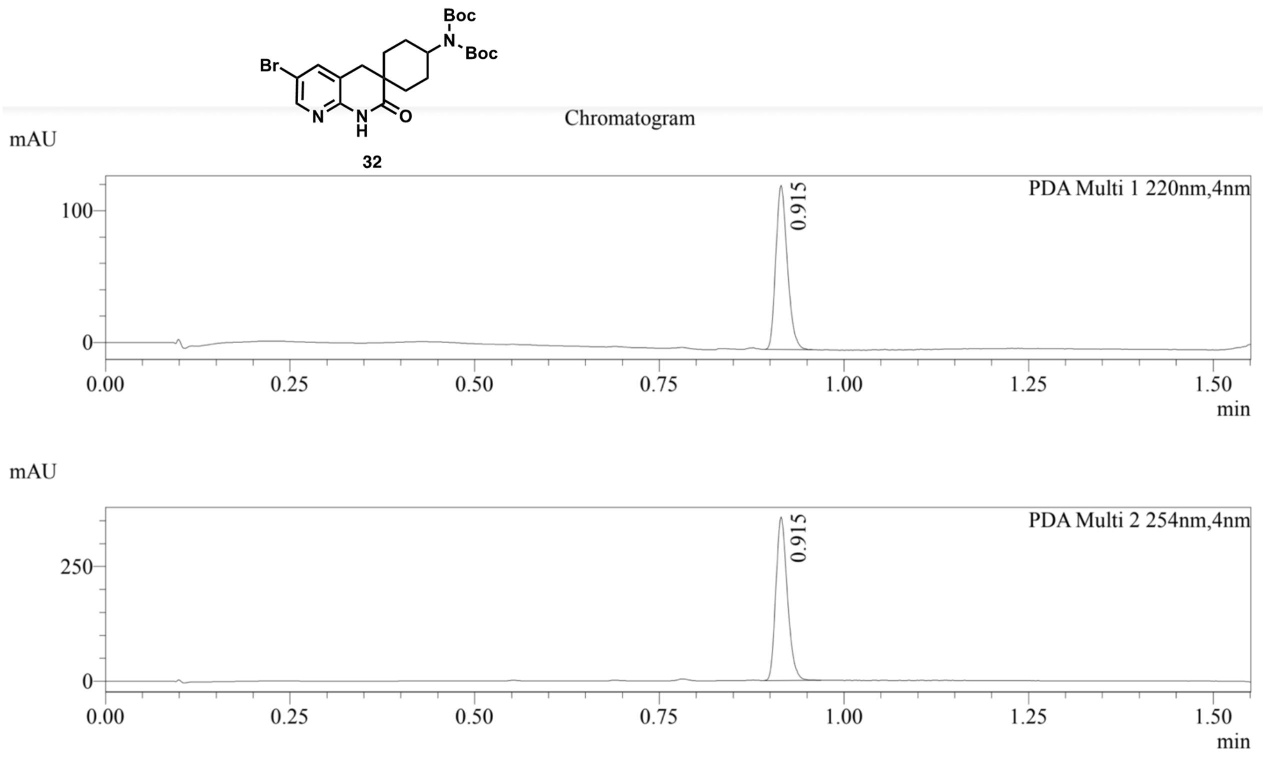
Br
Boc
Boc
32
Chromatogram
mAU
PDA Multi 1 220nm,4nm
0.915
mAU
PDA Multi 2 254nm,4nm
0.915
0.00 0.25 0.50 0.75 1.00 1.25 1.50
min

^{1}H NMR

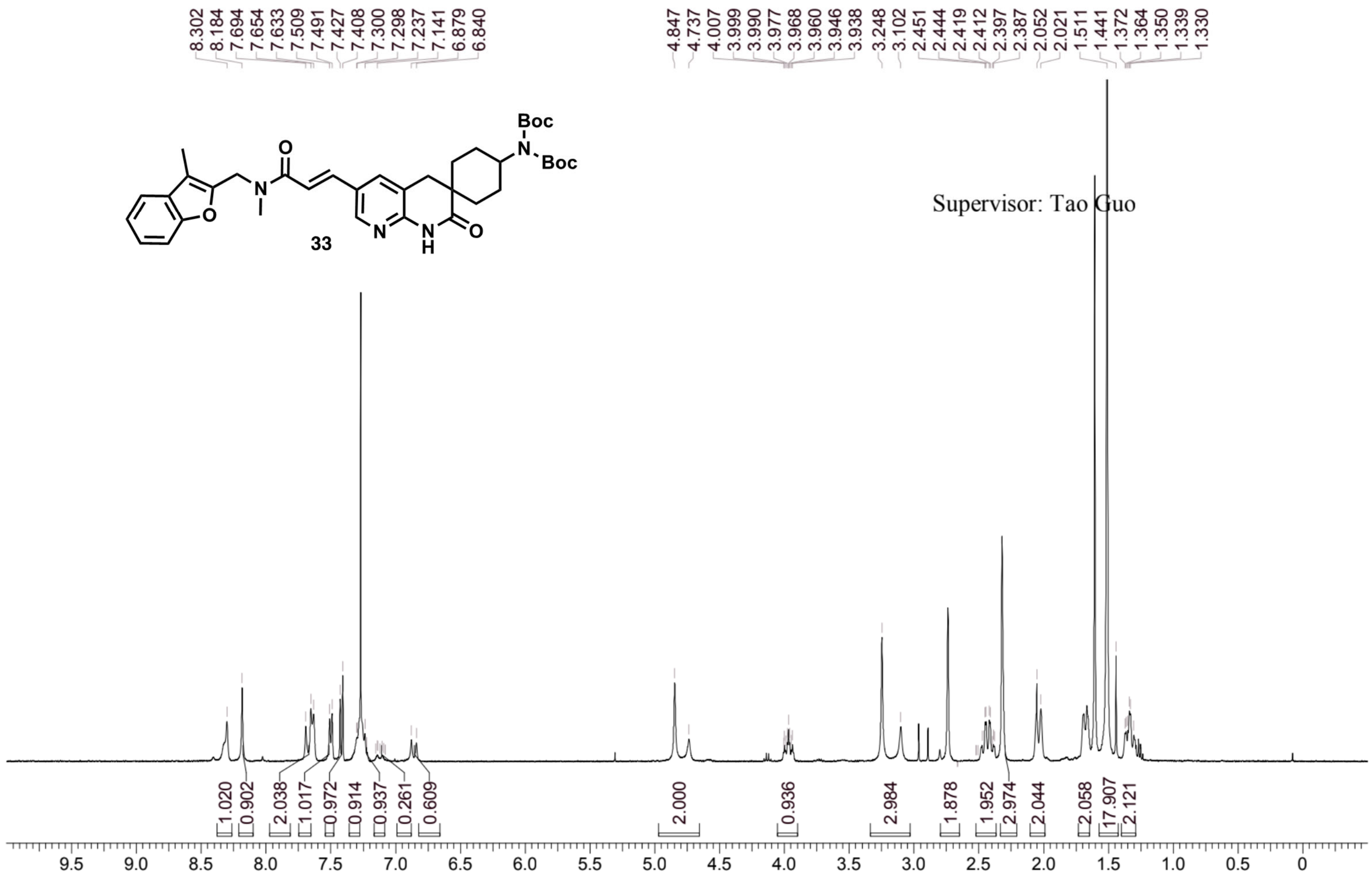

LCMS

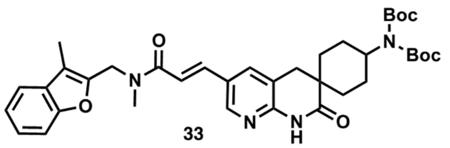

Chromatogram

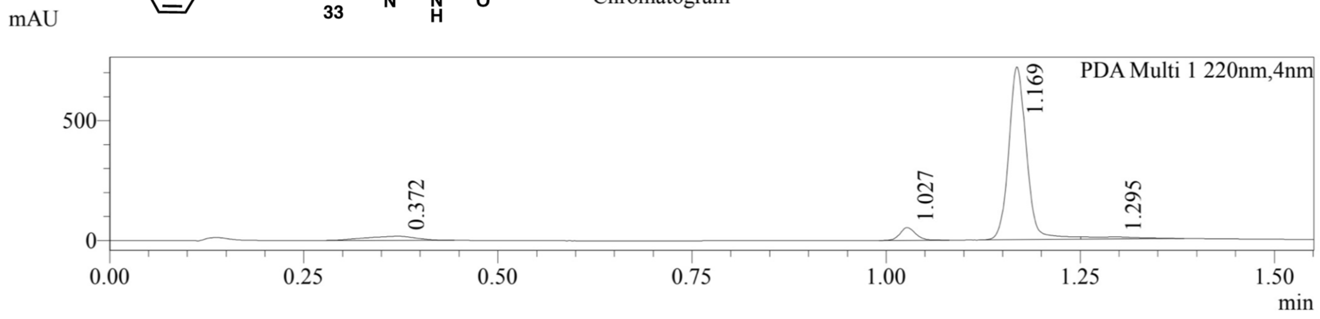

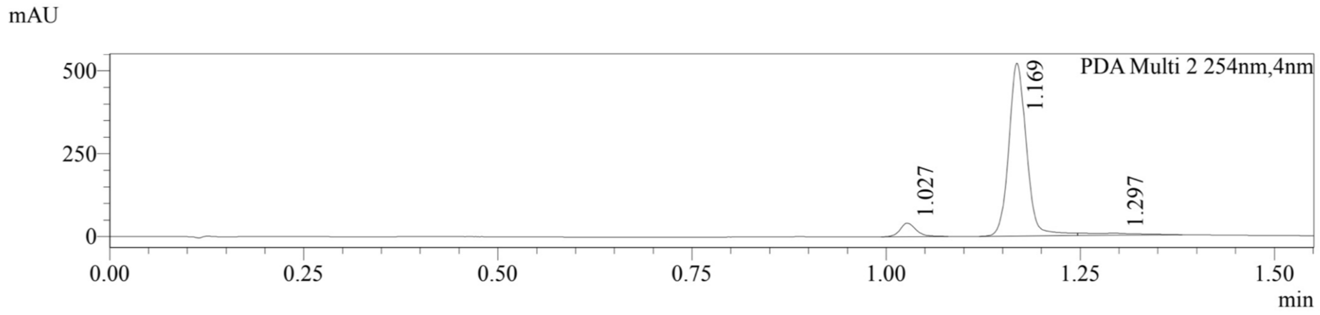

¹H NMR

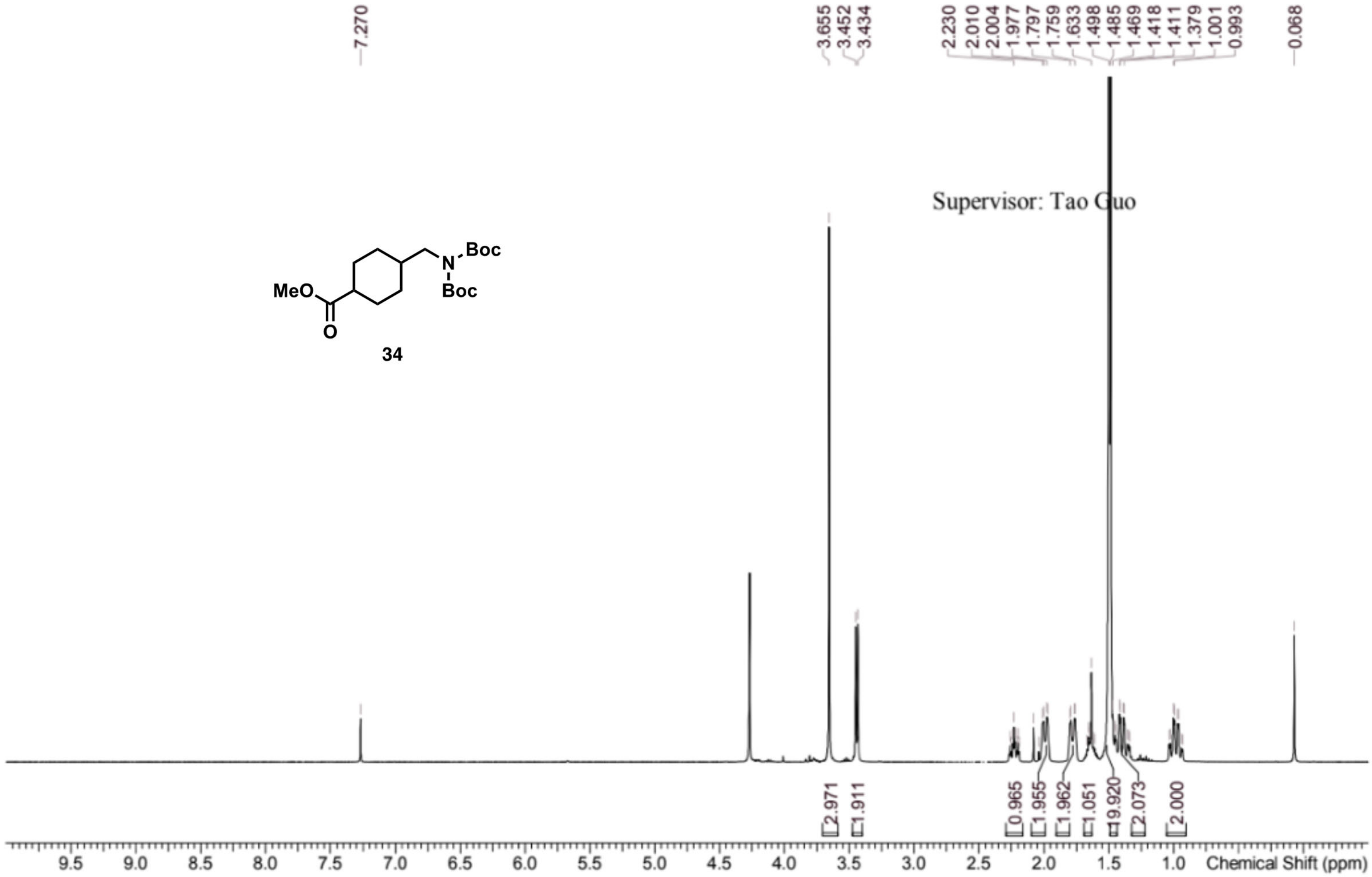

34

^{1}H NMR

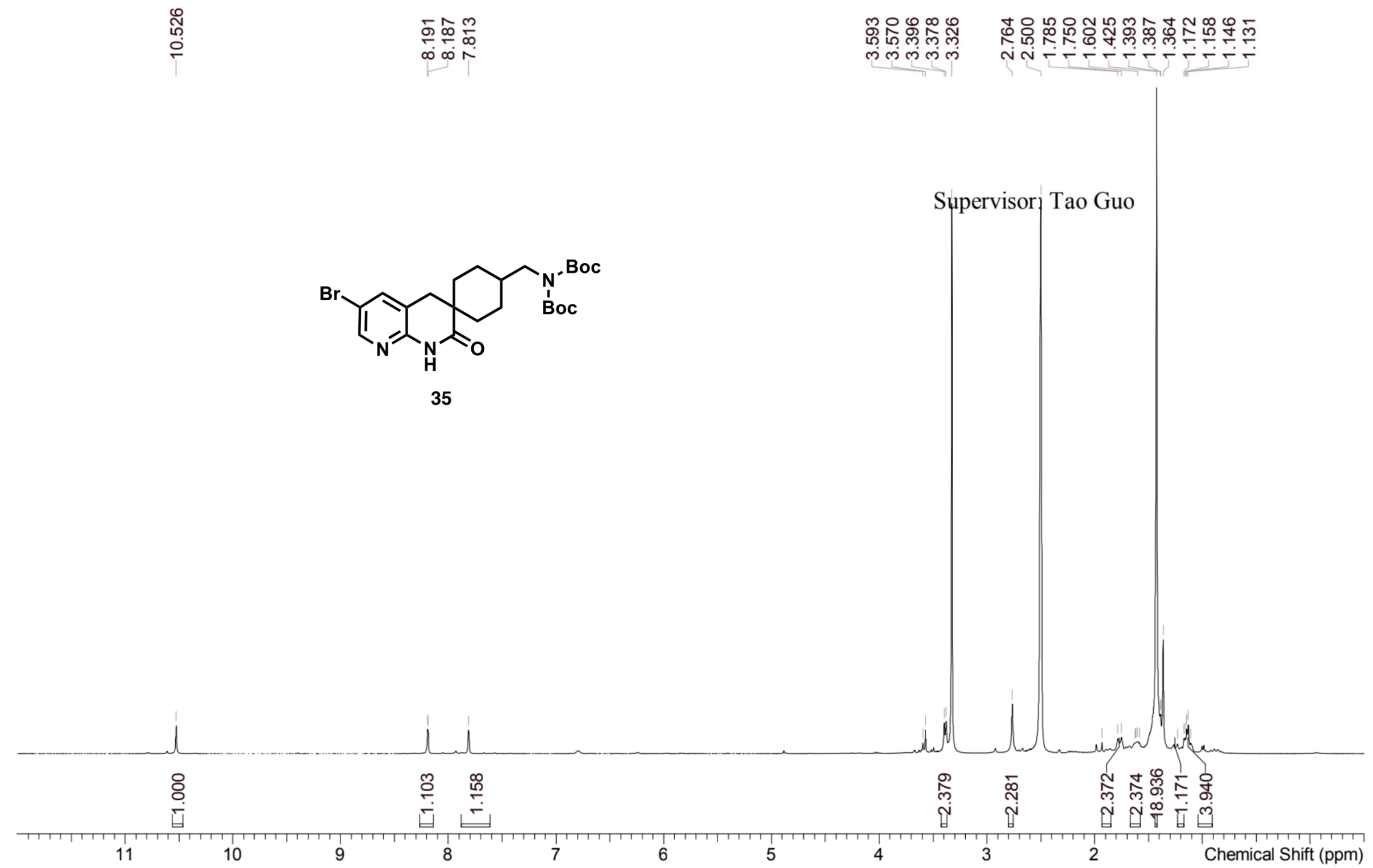

LCMS

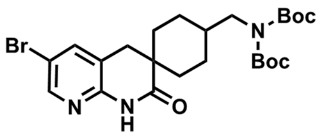

35

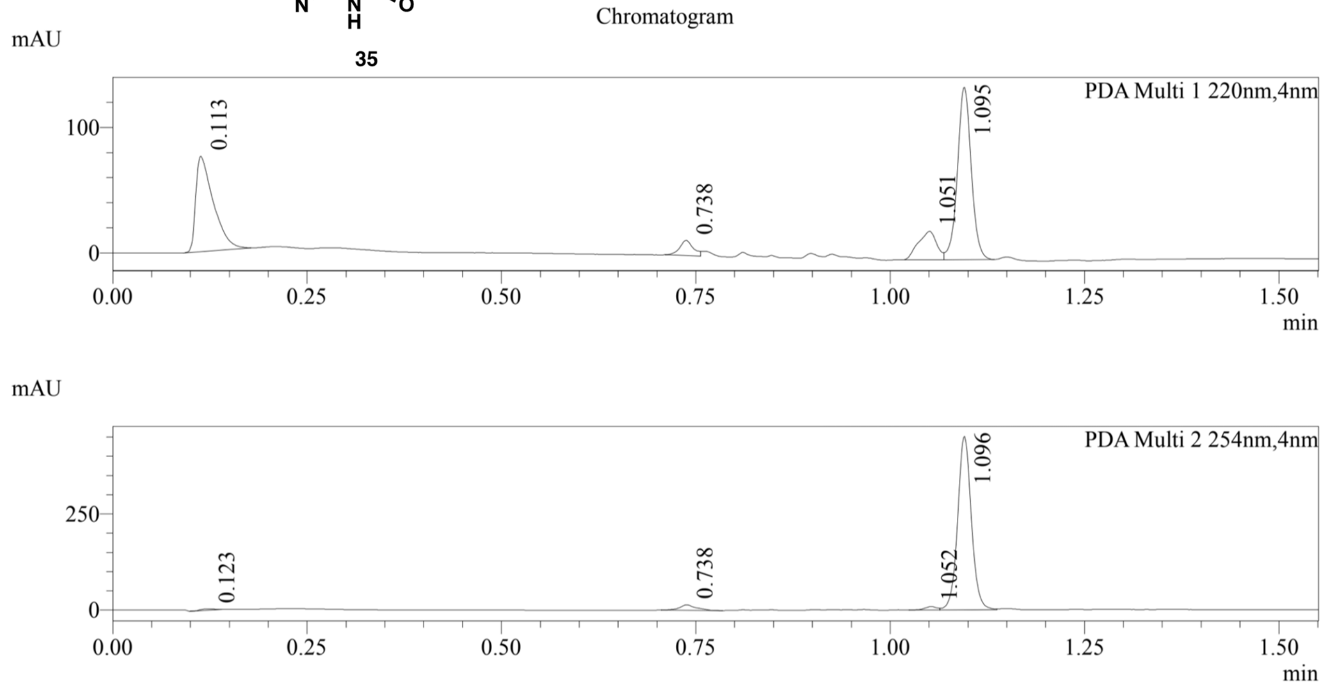

LCMS

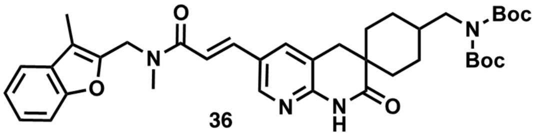

36

Chromatogram

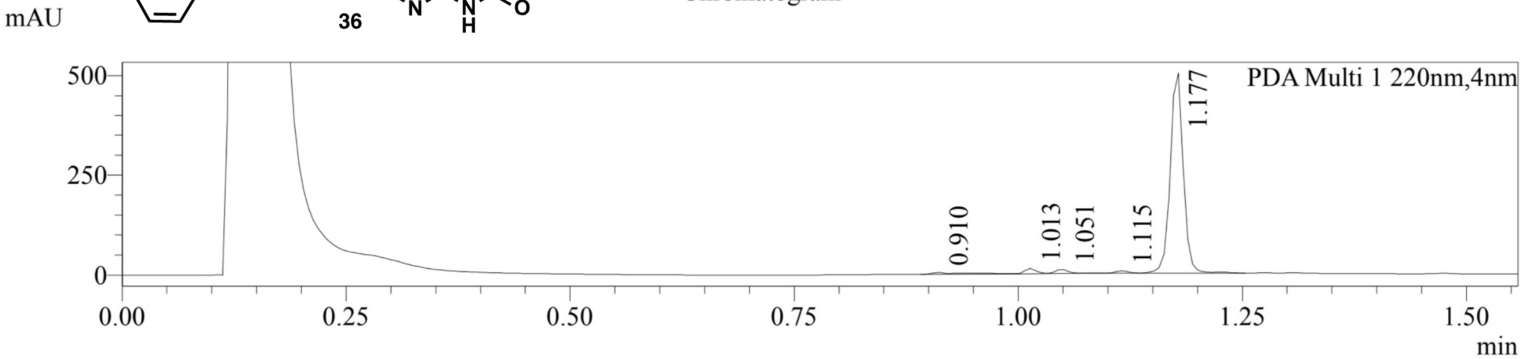

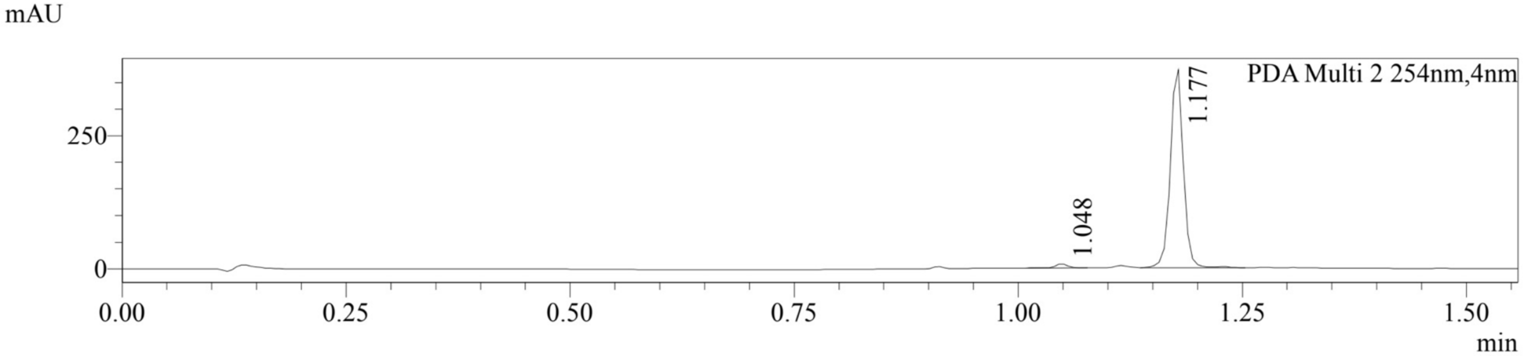

^{1}H NMR

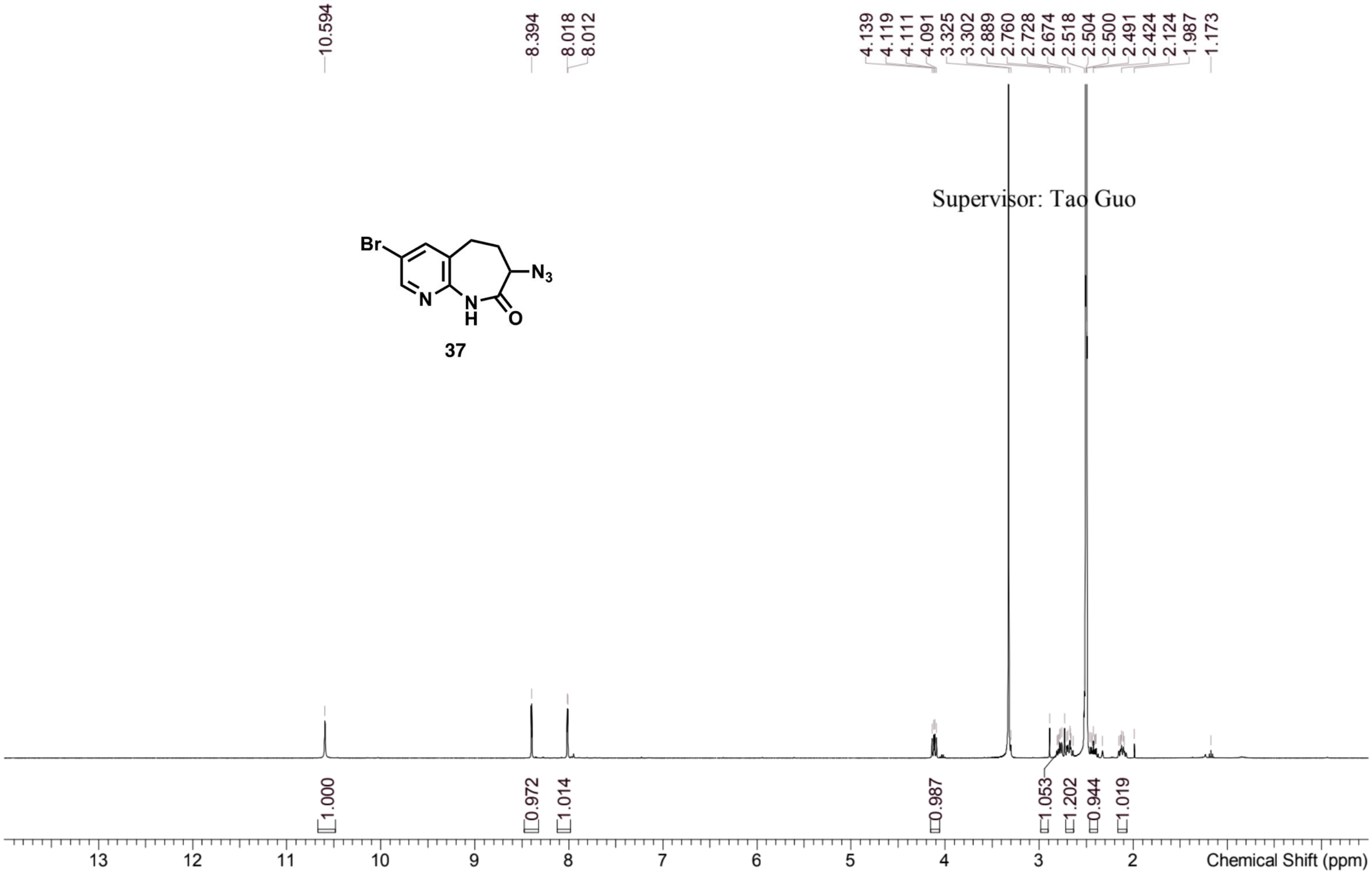
10.594
8.394
8.018
8.012
4.139
4.119
4.111
4.091
3.325
3.302
2.889
2.760
2.728
2.674
2.518
2.504
2.500
2.491
2.424
2.124
1.987
1.173
Supervisor: Tao Guo
Br
N
N
H
O
N_3
37
1.000
0.972
1.014
0.987
1.053
1.202
0.944
1.019
13
12
11
10
9
8
7
6
5
4
3
2
Chemical Shift (ppm)

LCMS

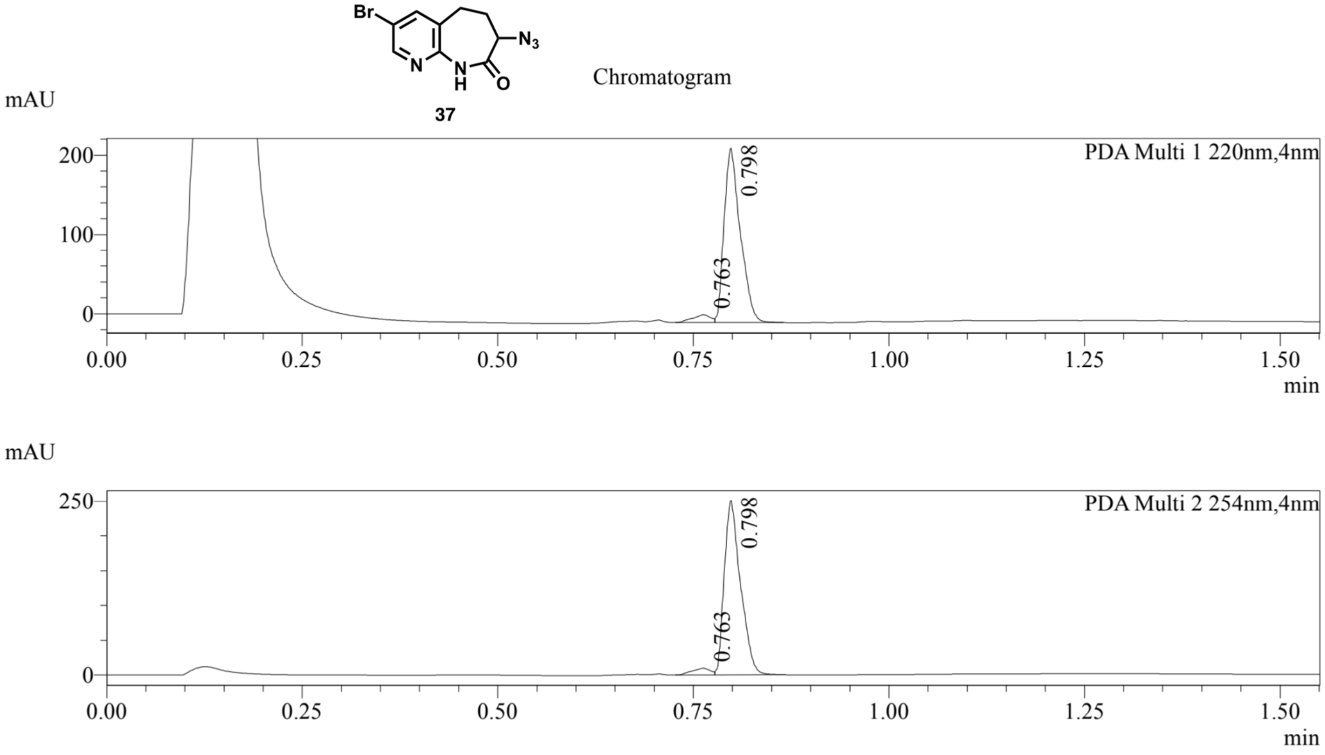
Br
N
N
H
O
N_3
37
Chromatogram
mAU
200
100
0
PDA Multi 1 220nm,4nm
0.798
0.763
0.00
0.25
0.50
0.75
1.00
1.25
1.50
min
mAU
250
0
PDA Multi 2 254nm,4nm
0.798
0.763
0.00
0.25
0.50
0.75
1.00
1.25
1.50
min

^{1}H NMR

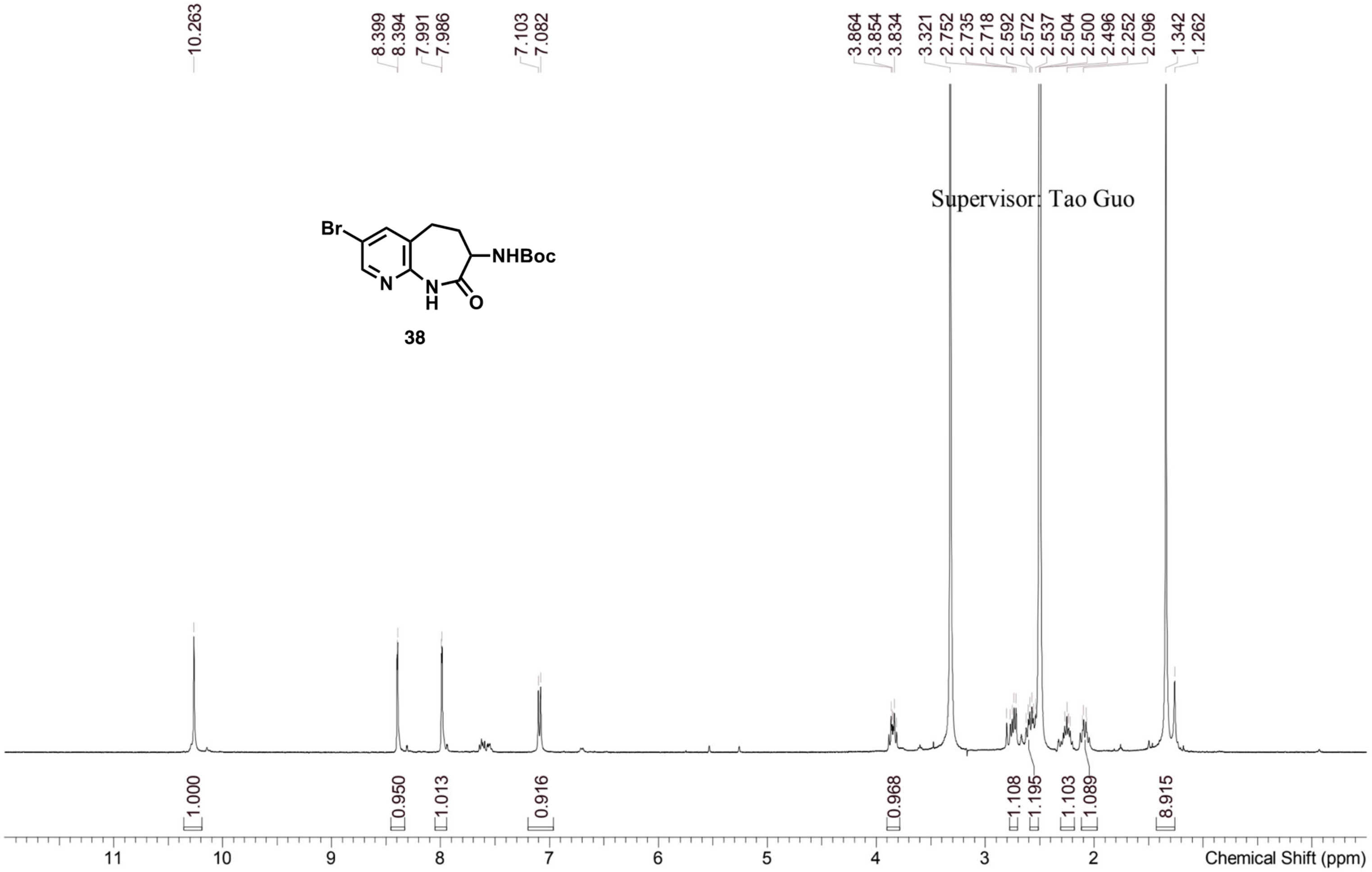
10.263
8.399
8.394
7.991
7.986
7.103
7.082
3.864
3.854
3.834
3.321
2.752
2.735
2.718
2.592
2.572
2.537
2.504
2.500
2.496
2.252
2.096
1.342
1.262
Supervisor: Tao Guo
Br
NHBoc
N
N
H
O
38
1.000
0.950
1.013
0.916
0.968
1.108
1.195
1.103
1.089
8.915
11
10
9
8
7
6
5
4
3
2
Chemical Shift (ppm)

LCMS

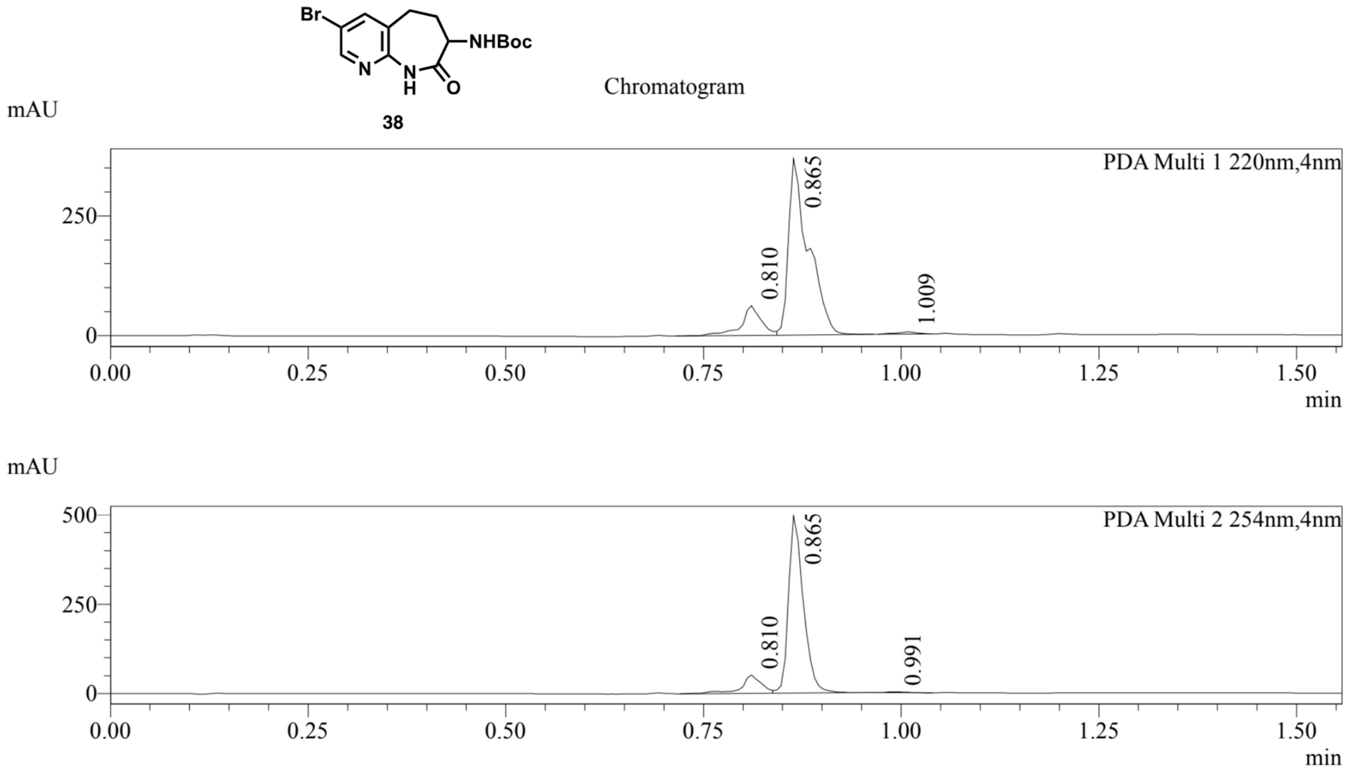
Br
NHBoc
N
N
H
O
38
Chromatogram
mAU
PDA Multi 1 220nm,4nm
250
0
0.810
0.865
1.009
0.00
0.25
0.50
0.75
1.00
1.25
1.50
min
mAU
PDA Multi 2 254nm,4nm
500
250
0
0.810
0.865
0.991
0.00
0.25
0.50
0.75
1.00
1.25
1.50
min

^{1}H NMR

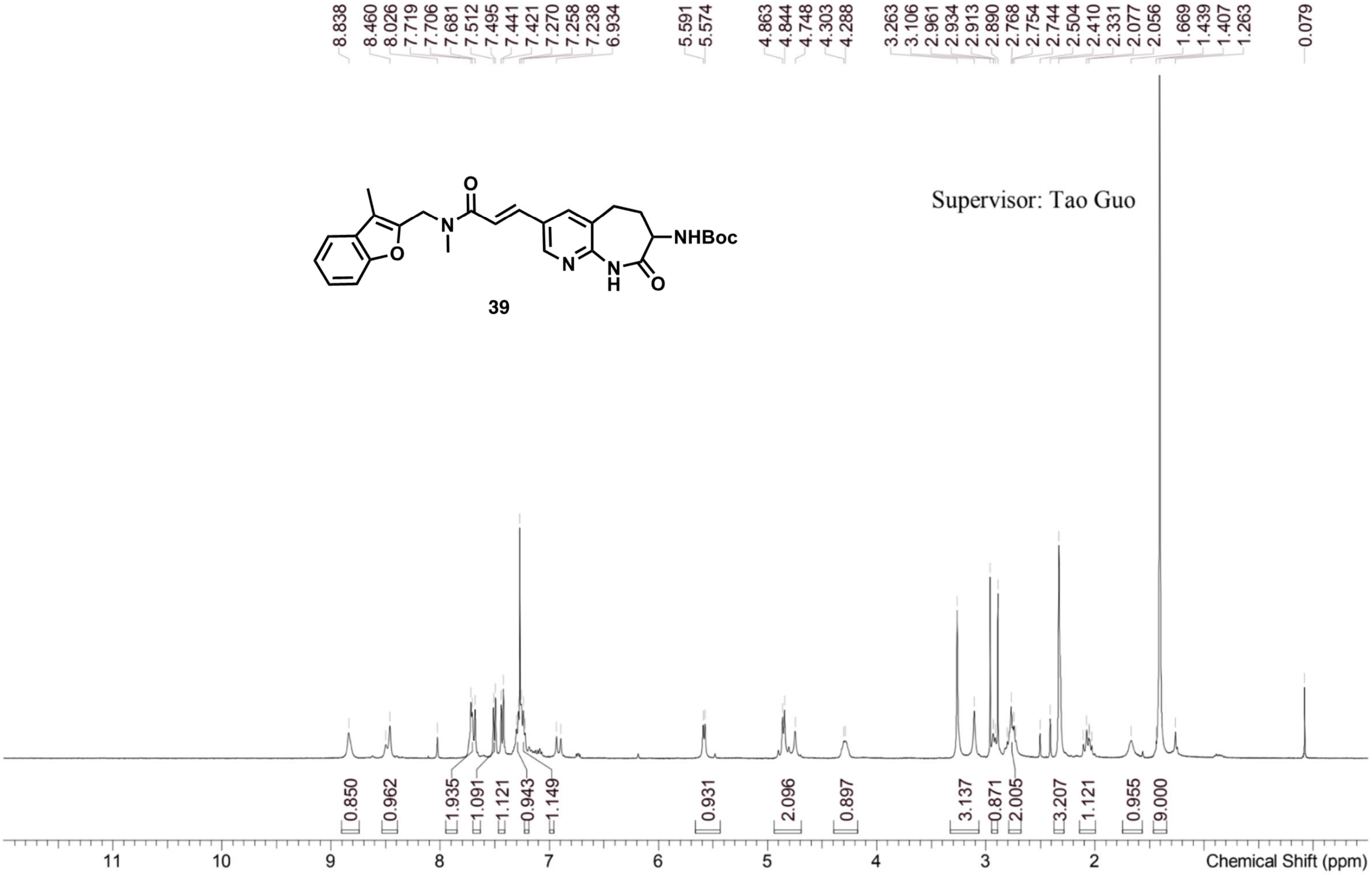

LCMS

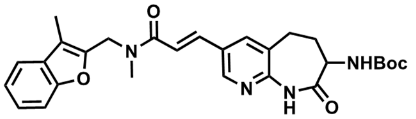

39

Chromatogram

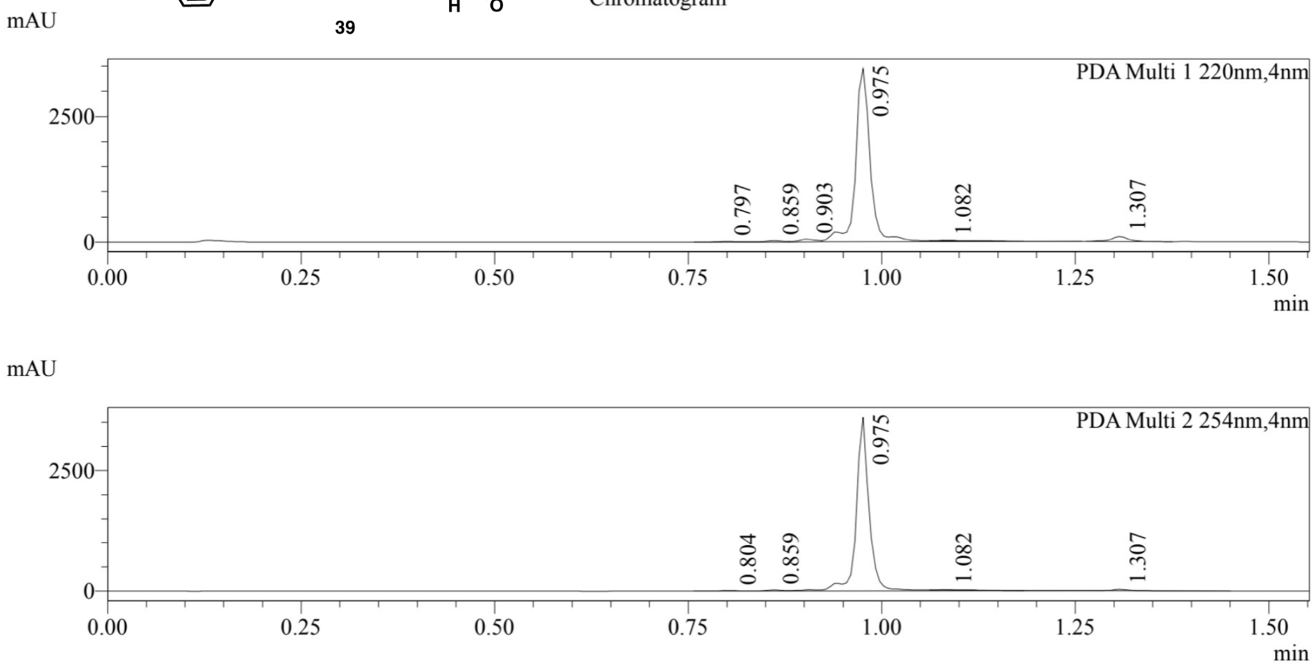

^{1}H NMR

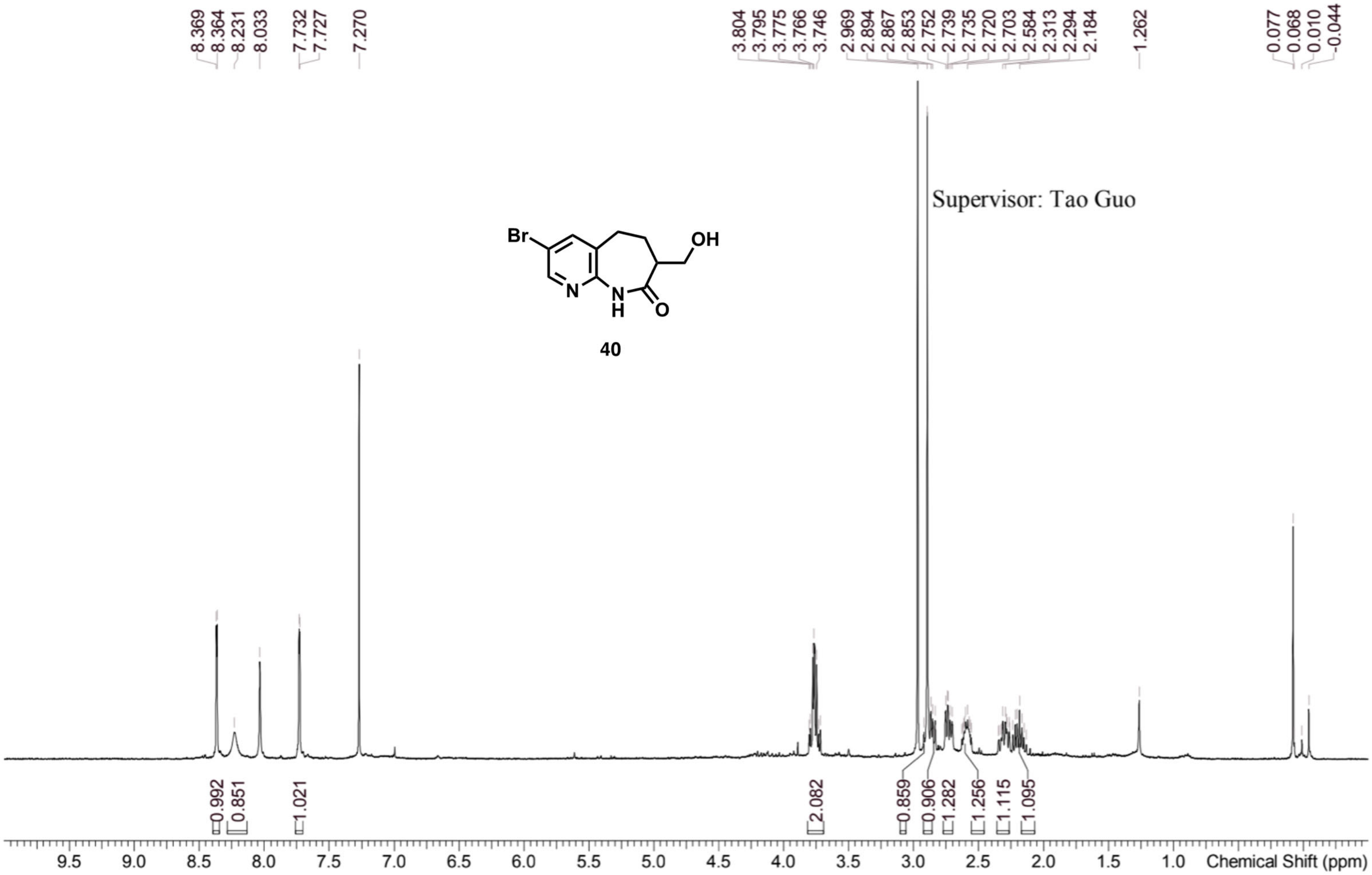

8.369
8.364
8.231
8.033
7.732
7.727
7.270
3.804
3.795
3.775
3.766
3.746
2.969
2.894
2.867
2.853
2.752
2.739
2.735
2.720
2.703
2.584
2.313
2.294
2.184
1.262
0.077
0.068
0.010
-0.044
Supervisor: Tao Guo
Br
OH
N
N
H
O
40
0.992
0.851
1.021
2.082
0.859
0.906
1.282
1.256
1.115
1.095
9.5 9.0 8.5 8.0 7.5 7.0 6.5 6.0 5.5 5.0 4.5 4.0 3.5 3.0 2.5 2.0 1.5 1.0 Chemical Shift (ppm)

LCMS

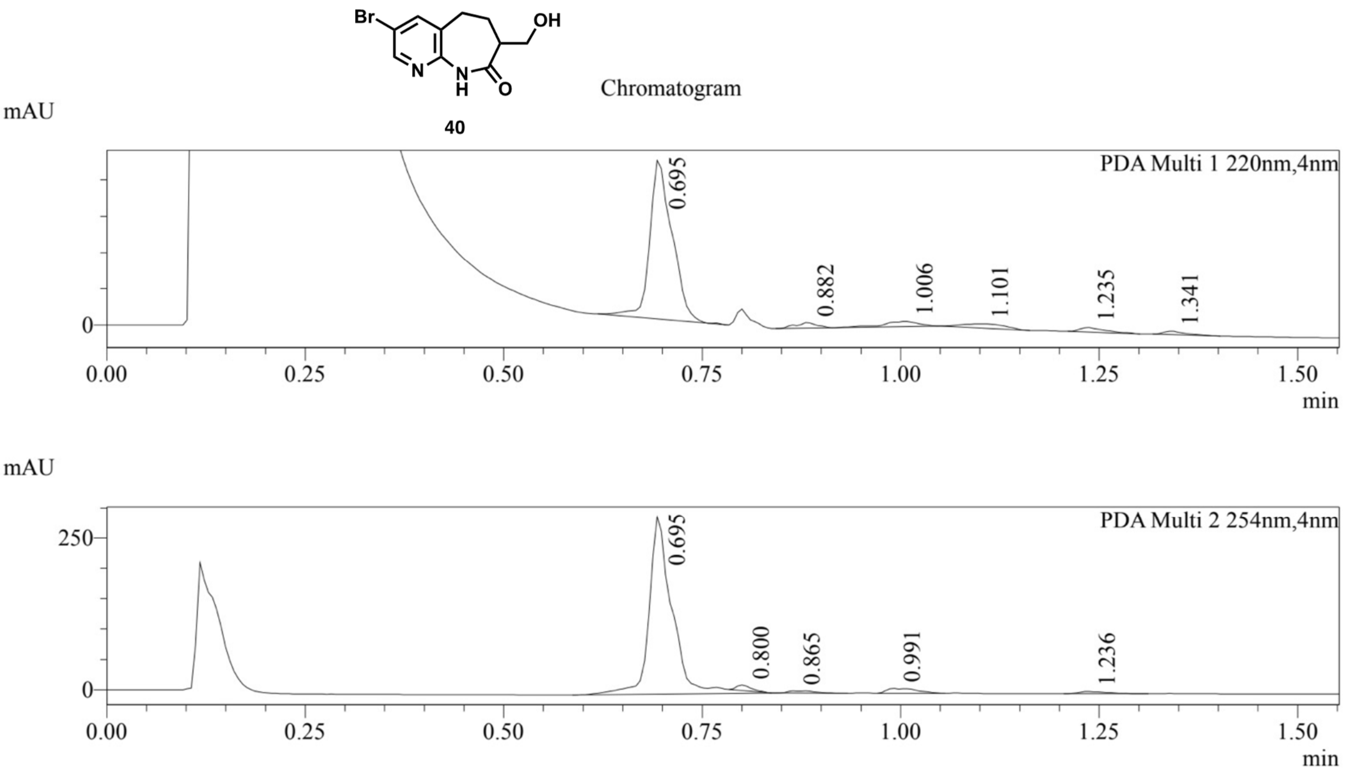

Br
OH
N
N
H
O
40
Chromatogram
mAU
PDA Multi 1 220nm,4nm
0.695
0.882
1.006
1.101
1.235
1.341
0
0.00 0.25 0.50 0.75 1.00 1.25 1.50
min
mAU
PDA Multi 2 254nm,4nm
250
0
0.695
0.800
0.865
0.991
1.236
0.00 0.25 0.50 0.75 1.00 1.25 1.50
min

^{1}H NMR

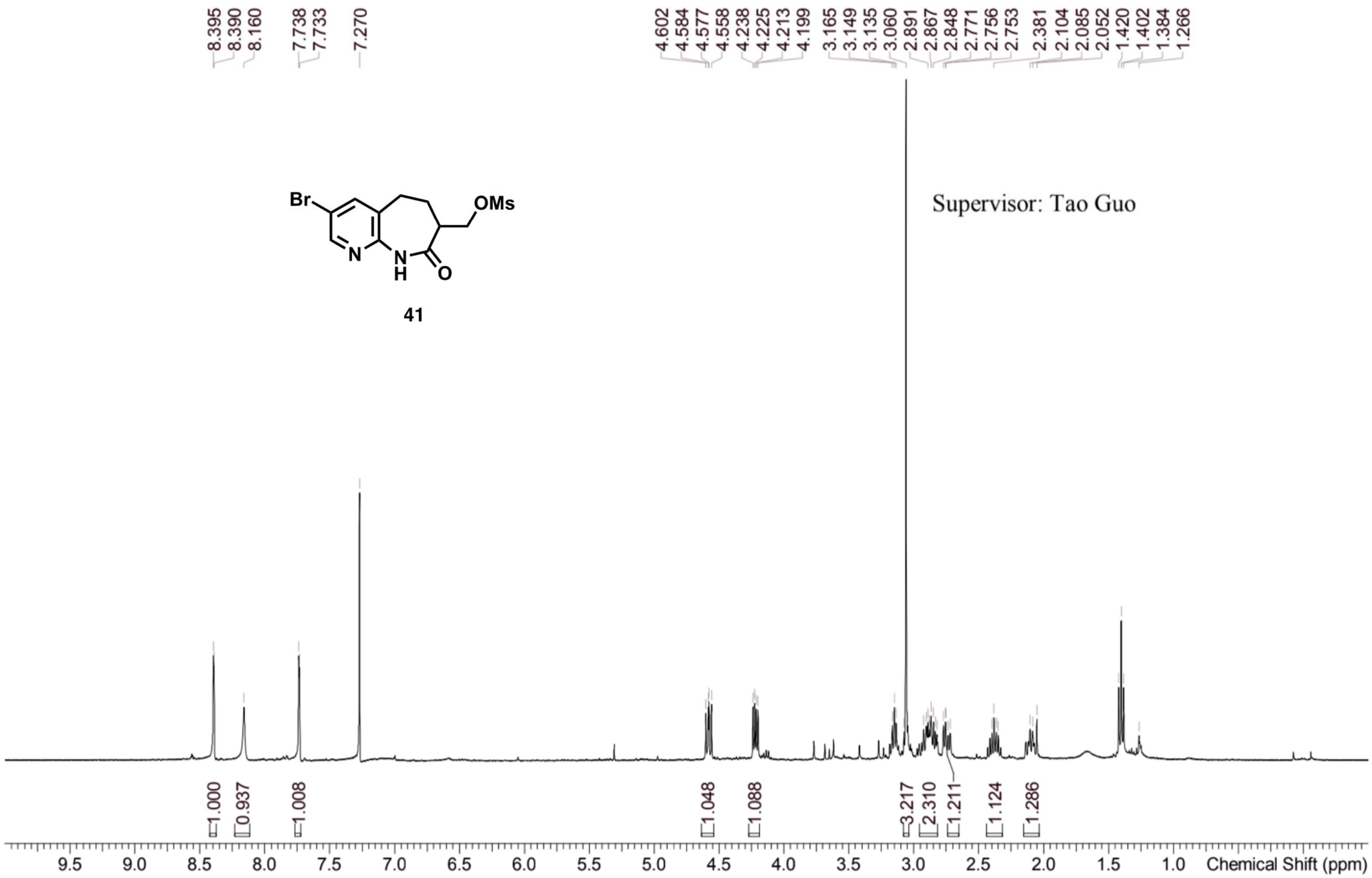

LCMS

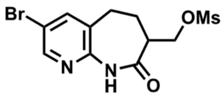

41

Chromatogram

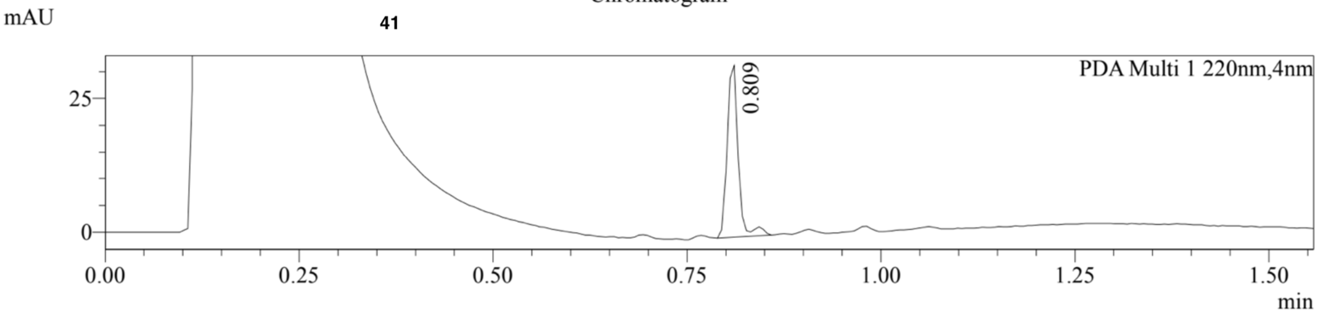

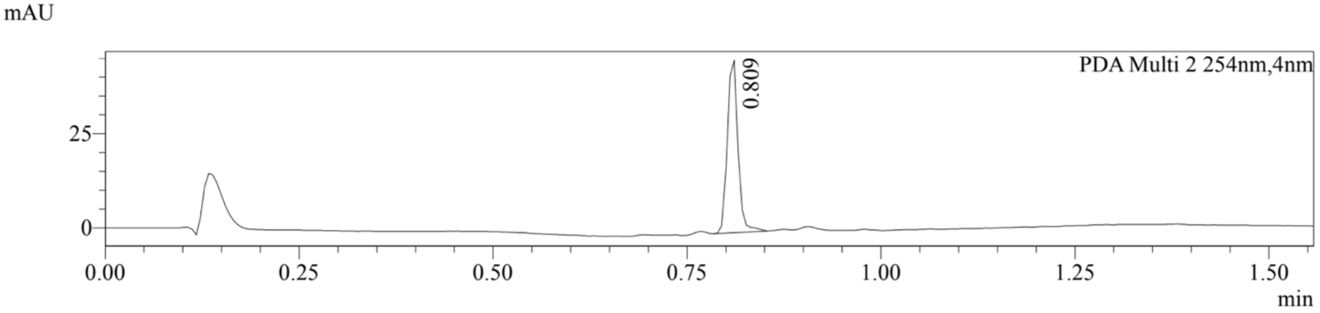

LCMS

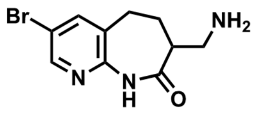

42

Chromatogram

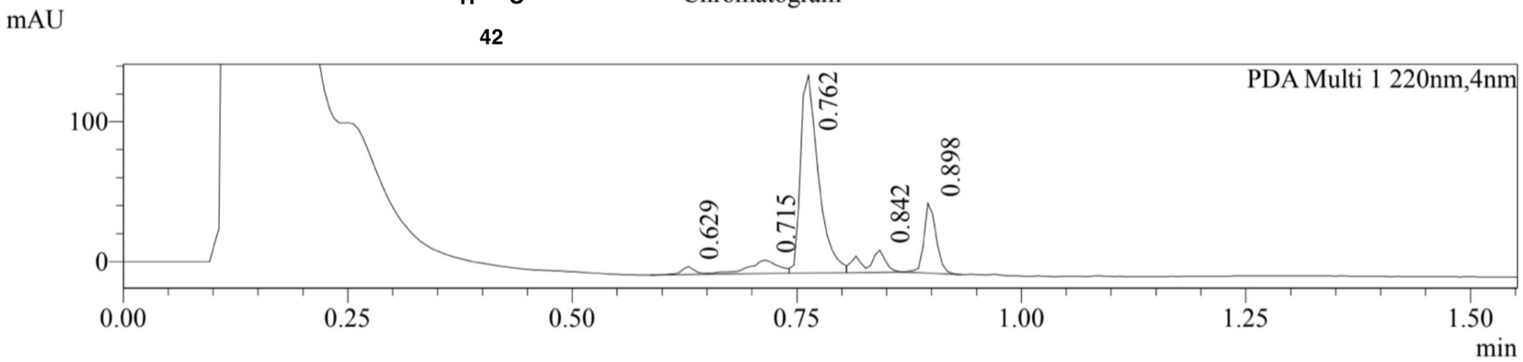

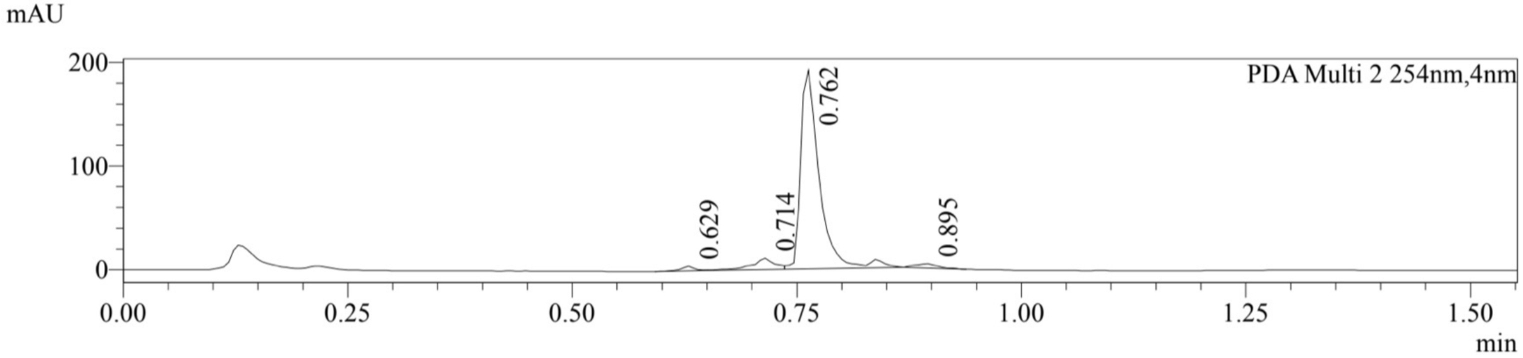

^{1}H NMR

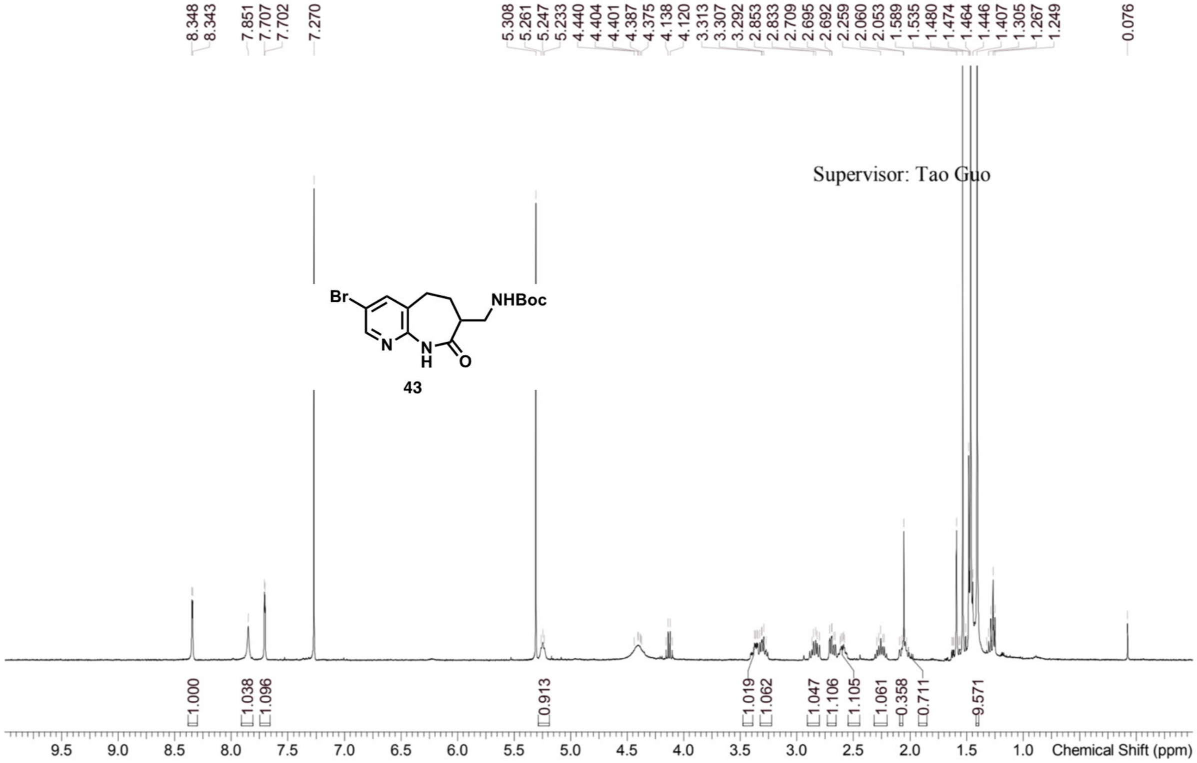

LCMS

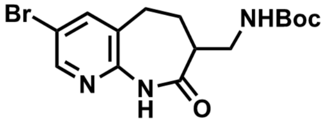

43

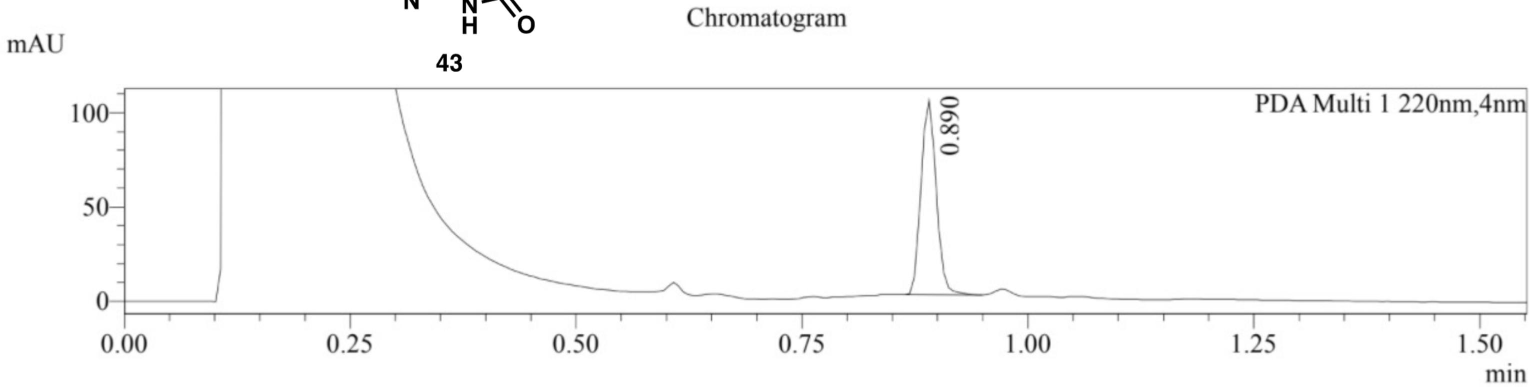

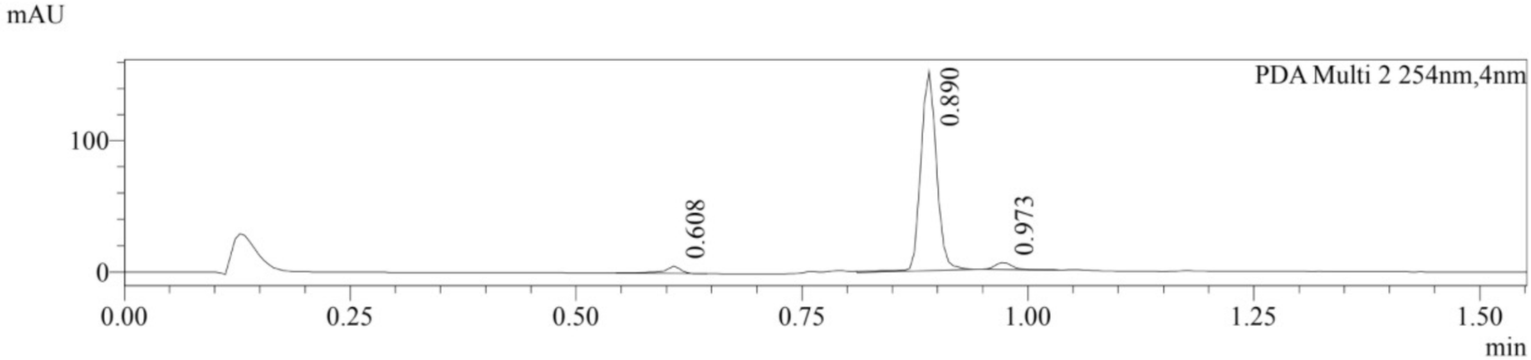

^{1}H NMR

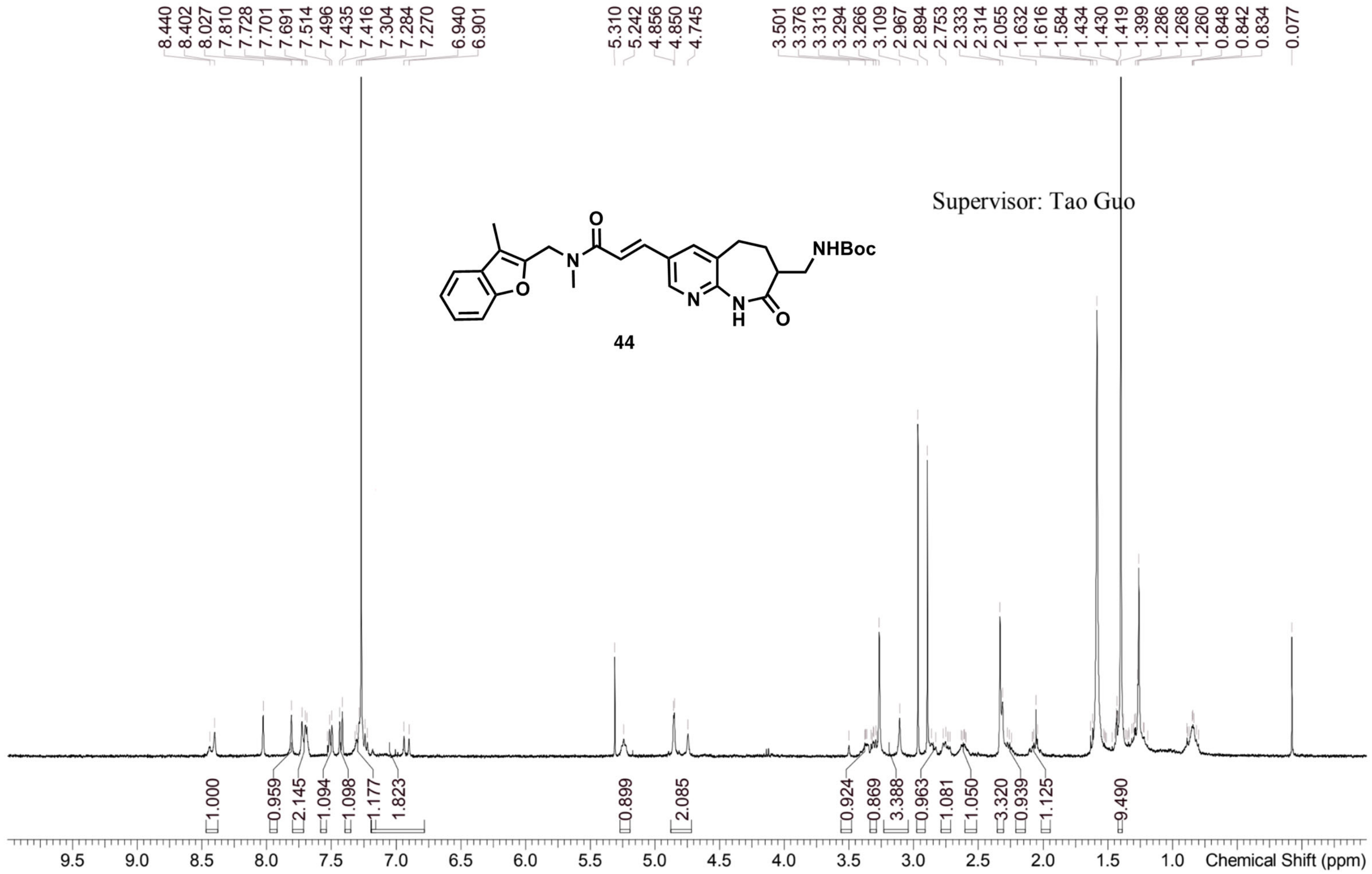

LCMS

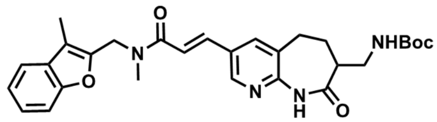

44

Chromatogram

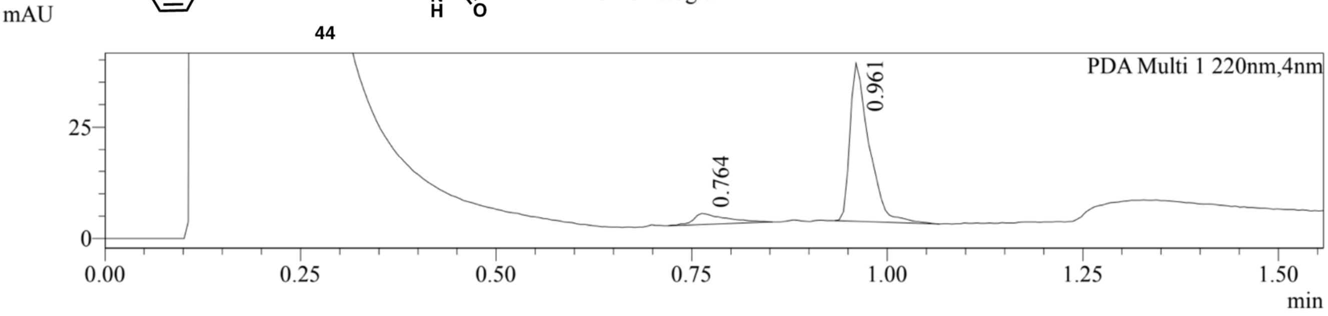

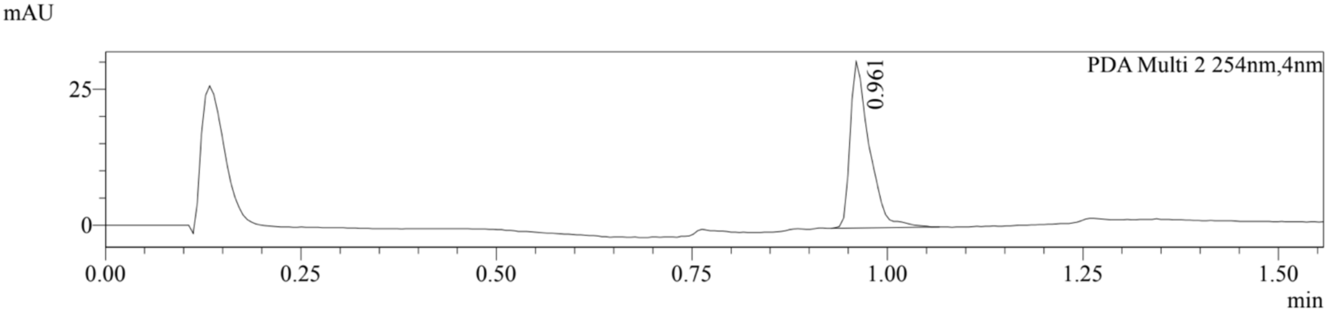

^{1}H NMR

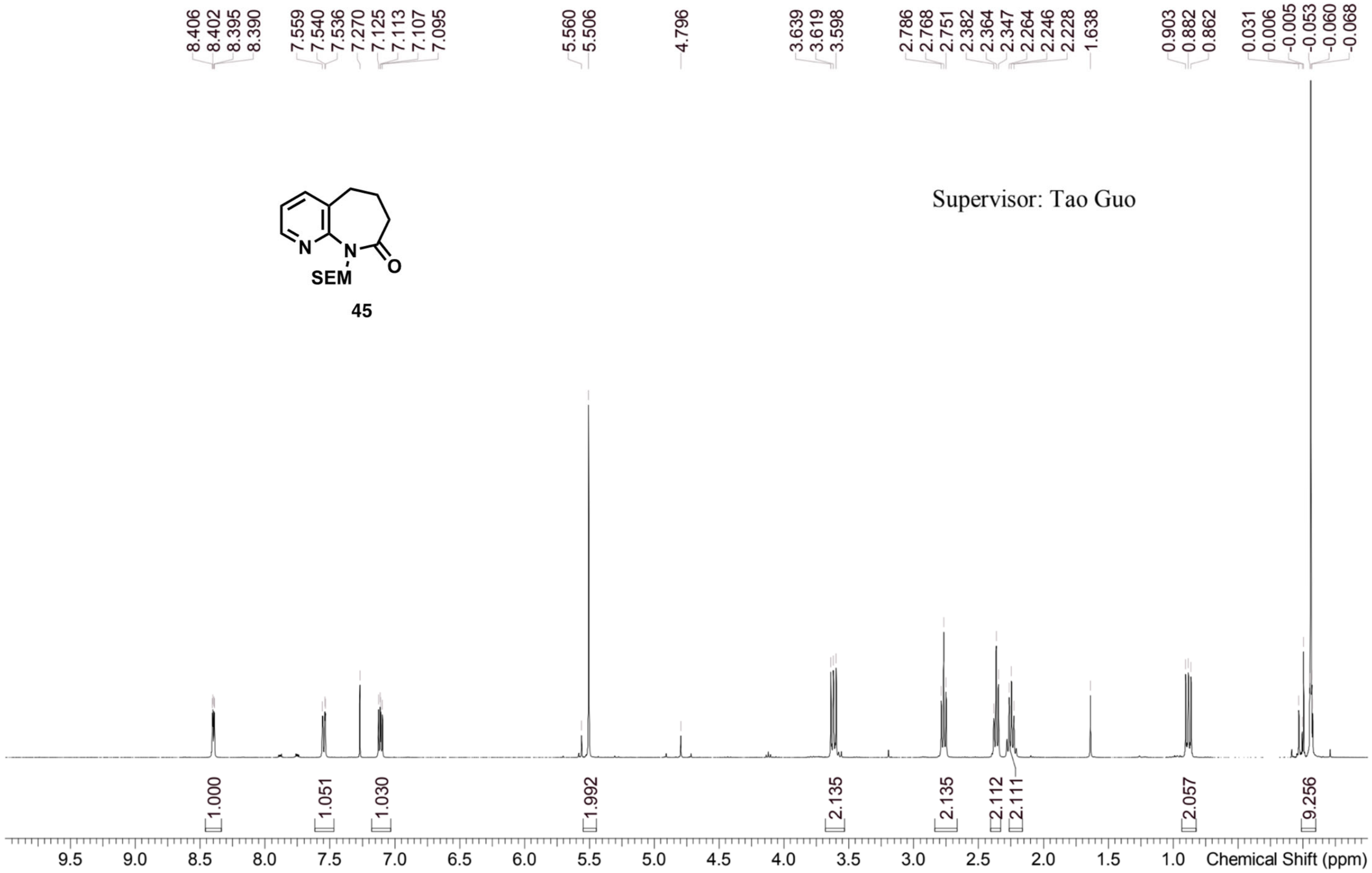

LCMS

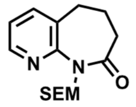

45

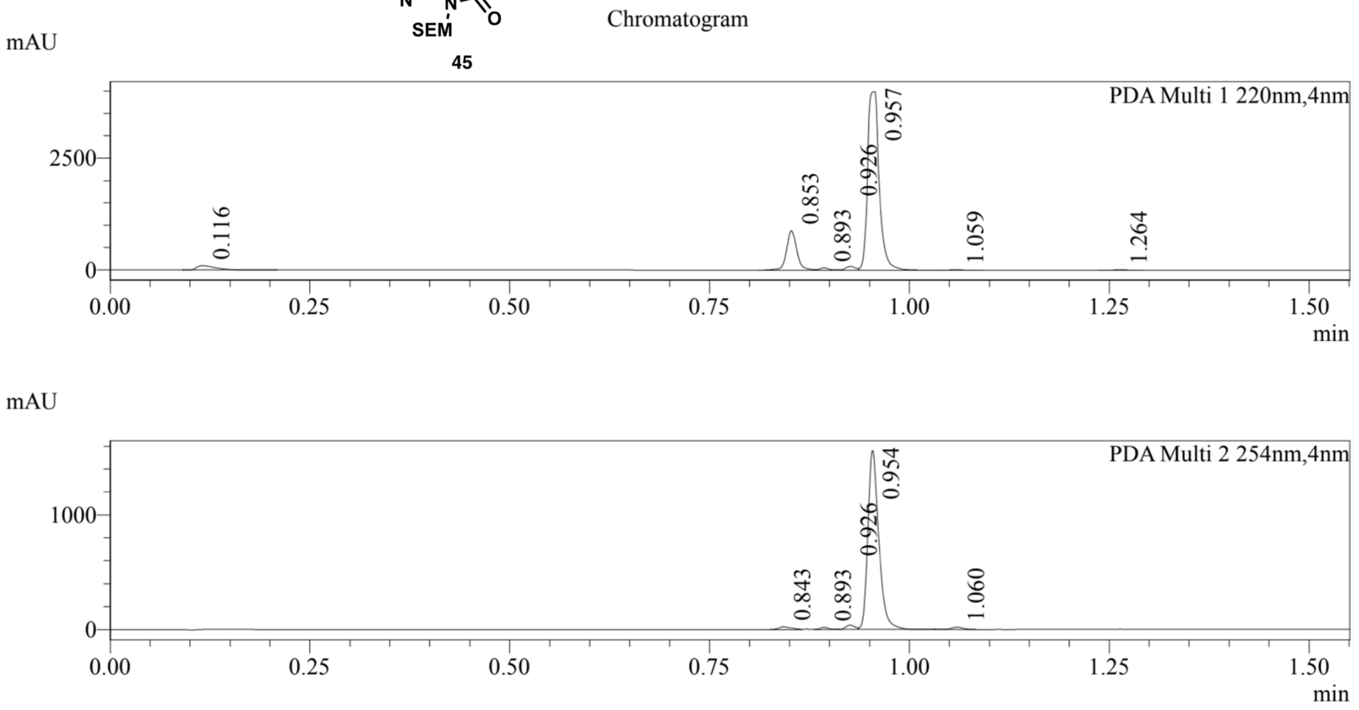

^{1}H NMR

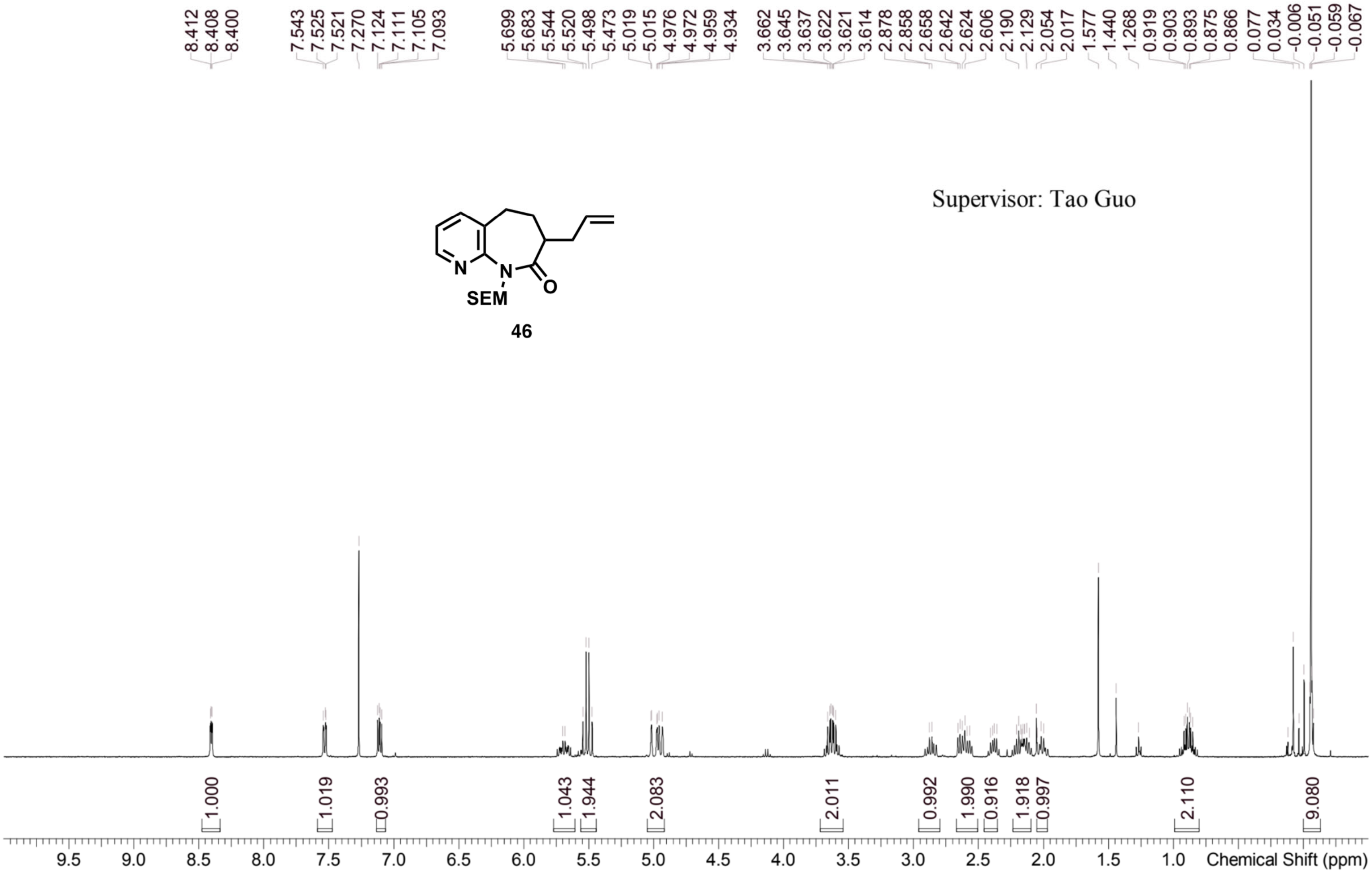

LCMS

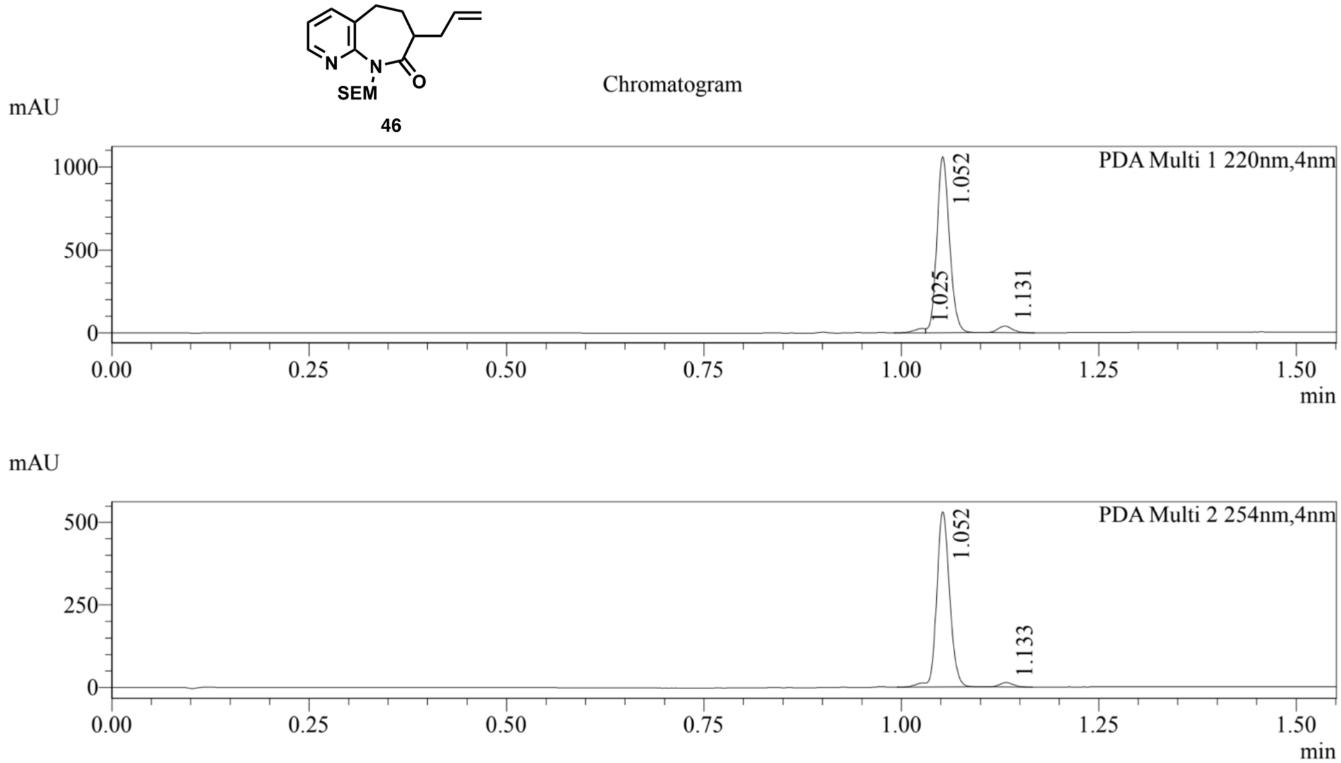

^{1}H NMR

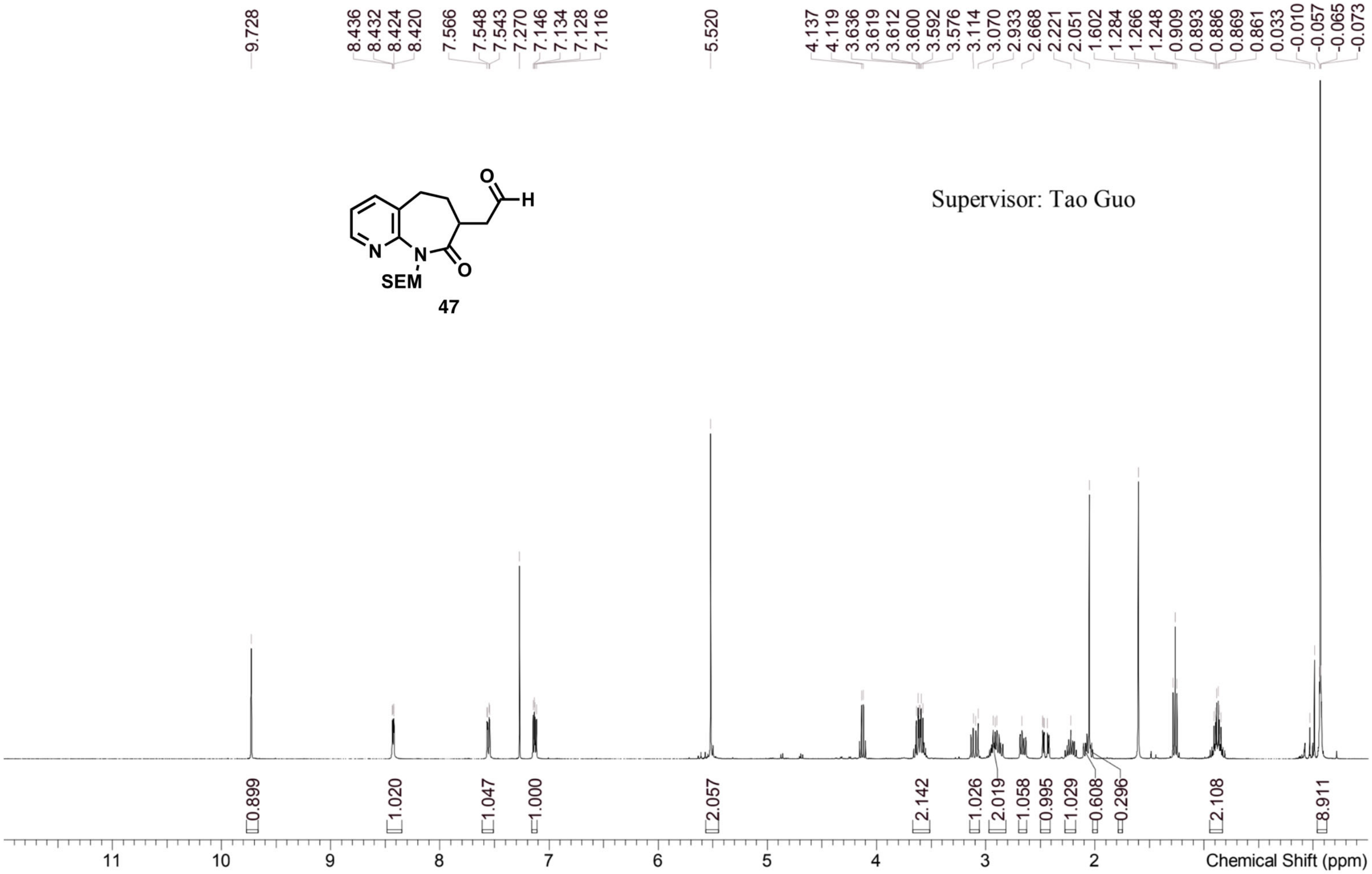
SEM
47
Supervisor: Tao Guo
Chemical Shift (ppm)

LCMS

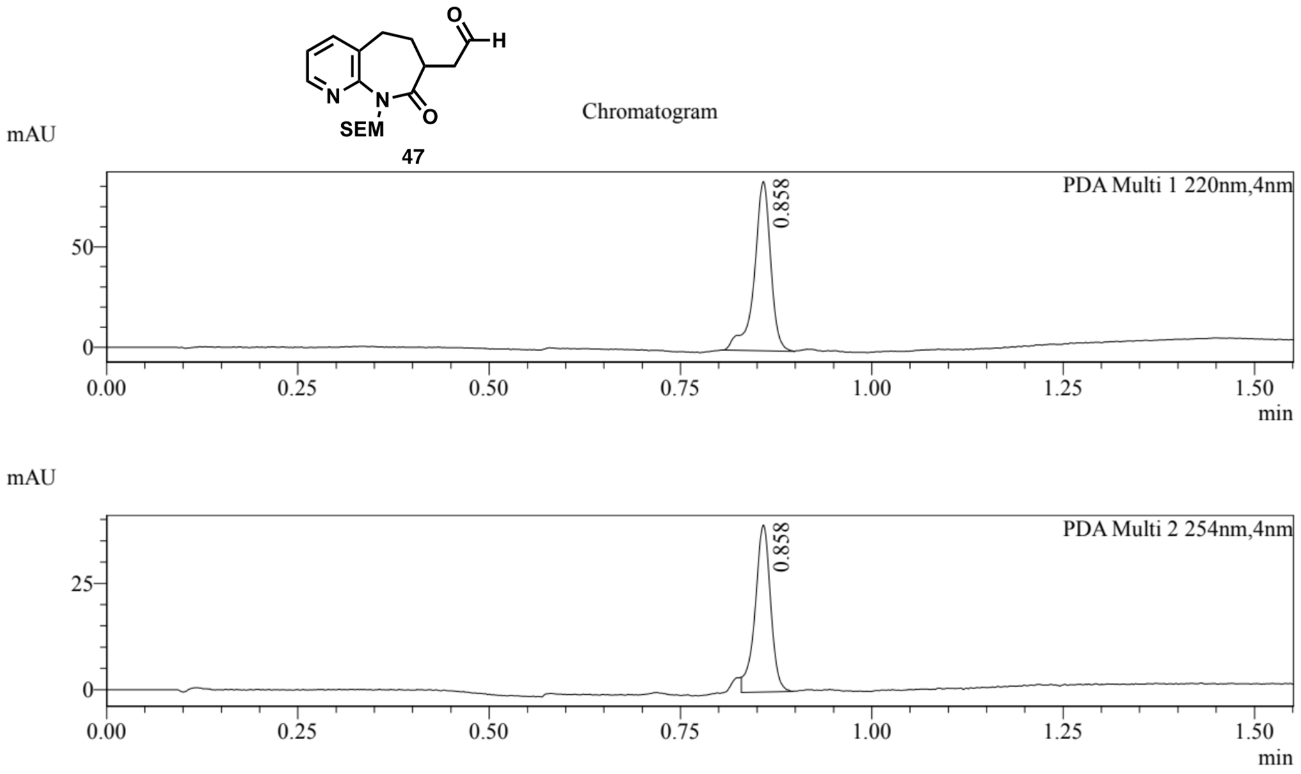
SEM
47
Chromatogram
mAU
PDA Multi 1 220nm,4nm
0.858
min
mAU
PDA Multi 2 254nm,4nm
0.858
min

LCMS

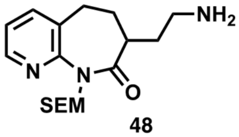

48

Chromatogram

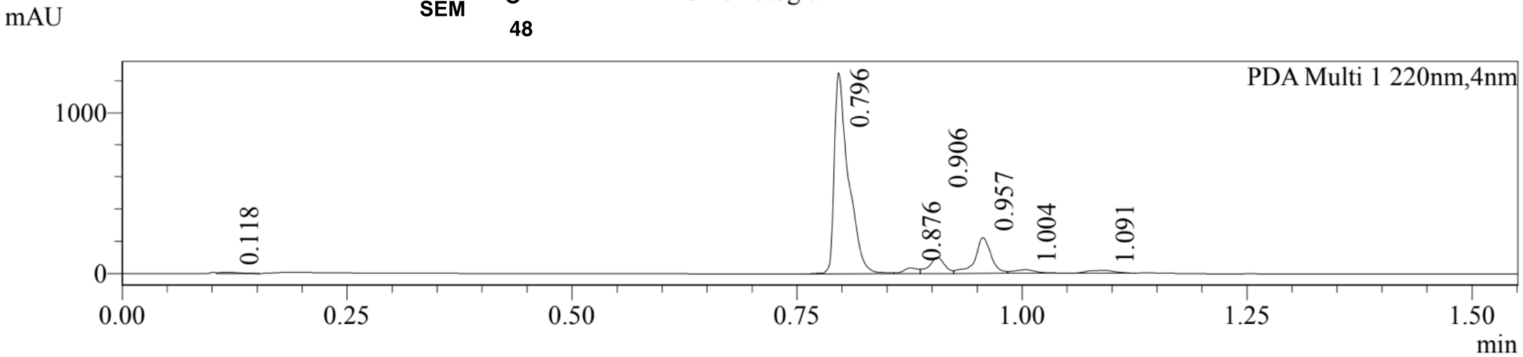

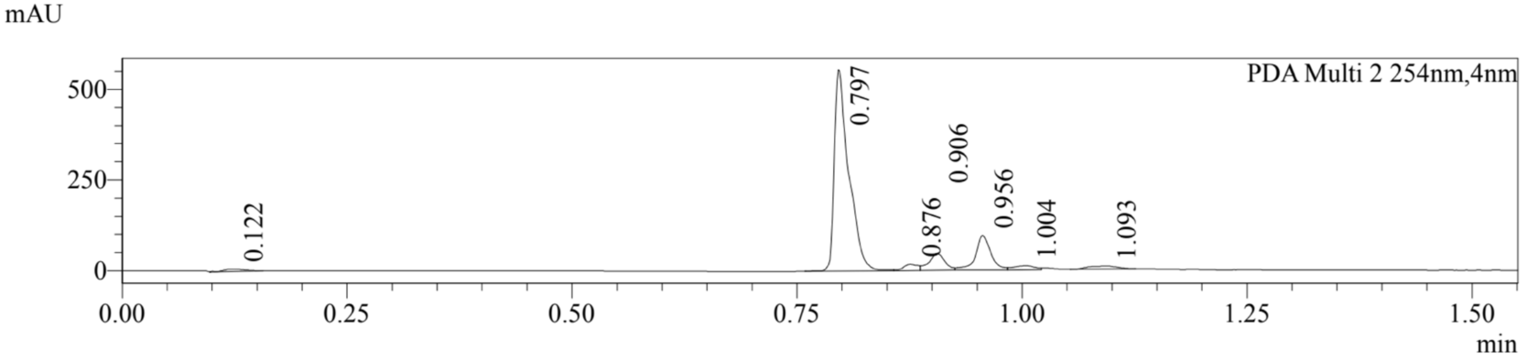

^{1}H NMR

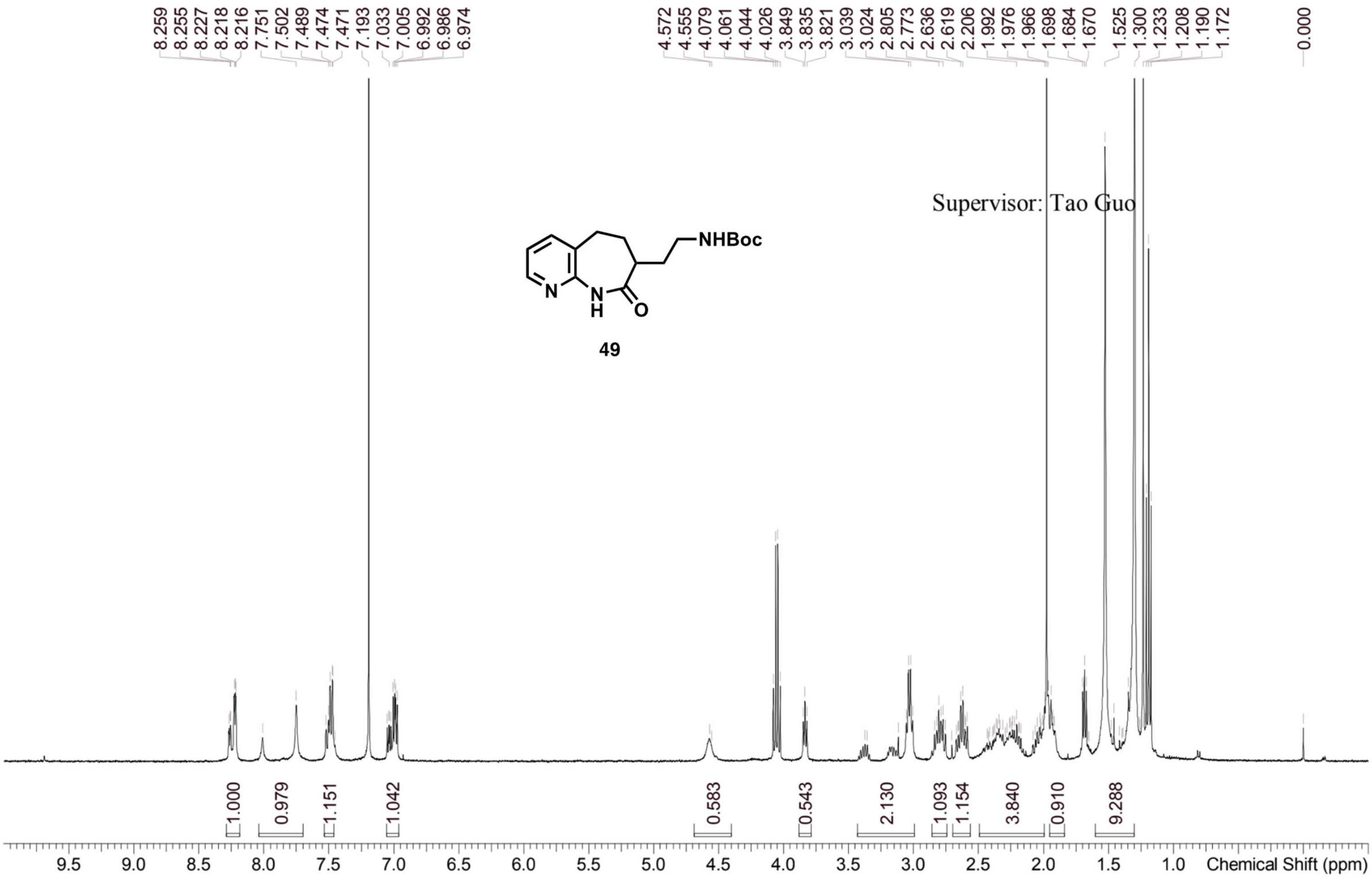

LCMS

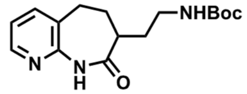

49

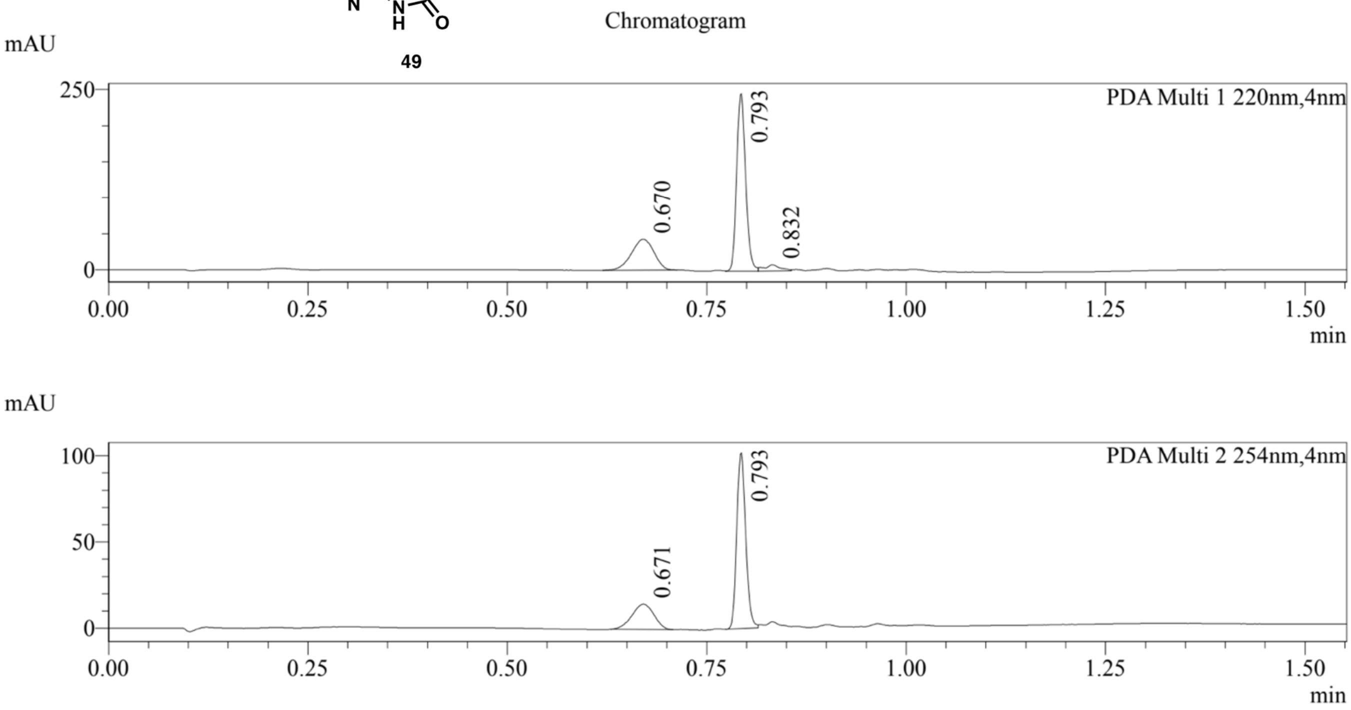

^{1}H NMR

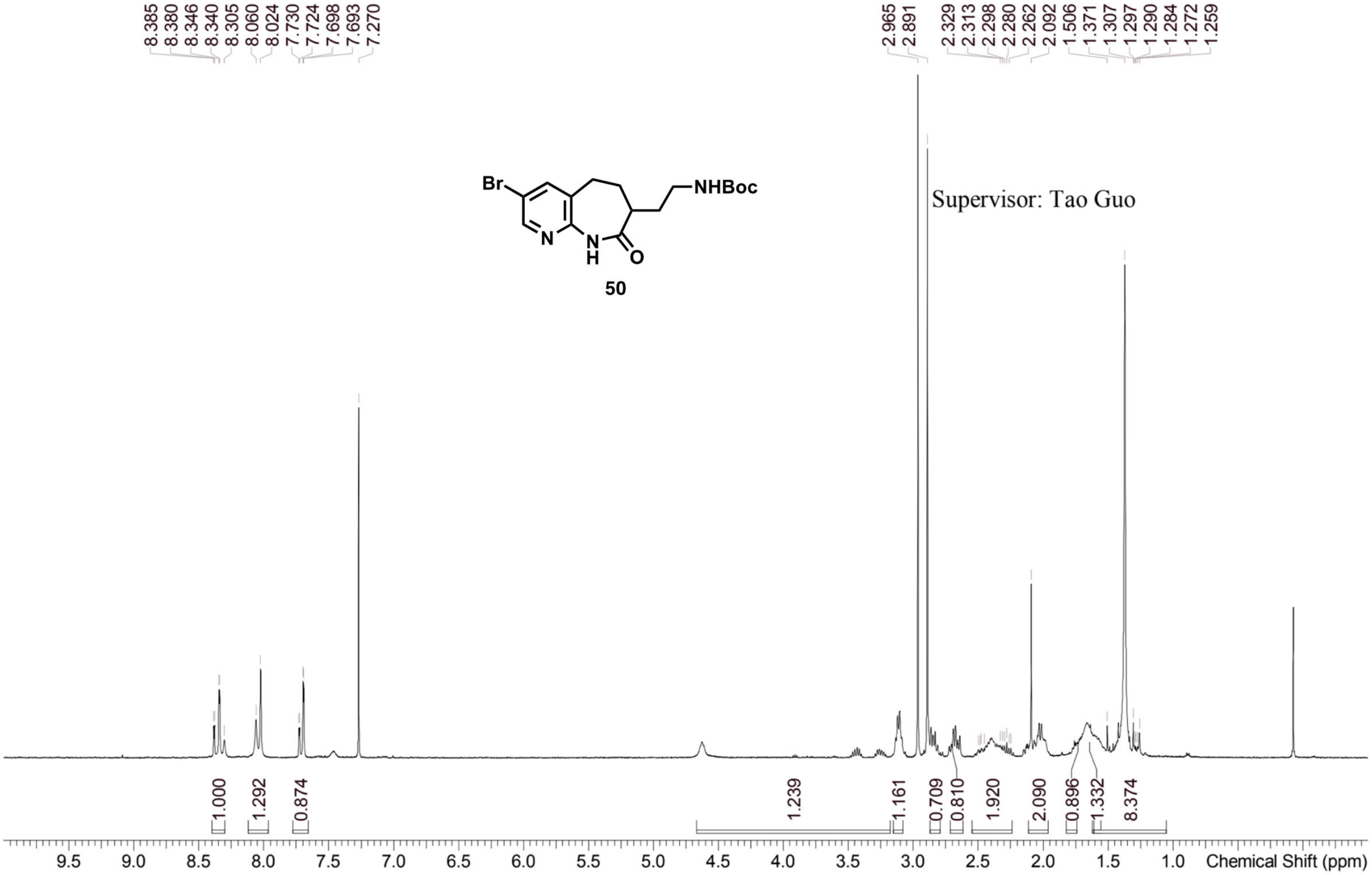

LCMS

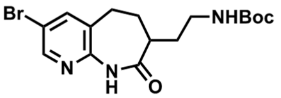

50

Chromatogram

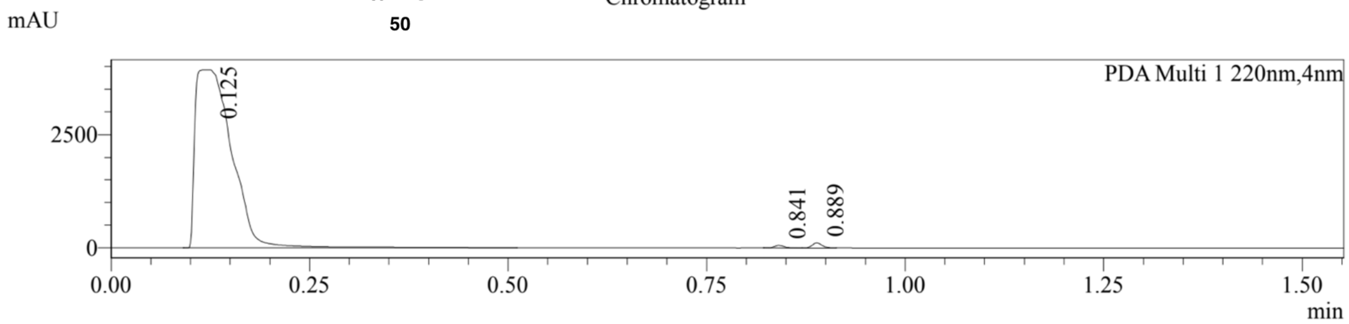

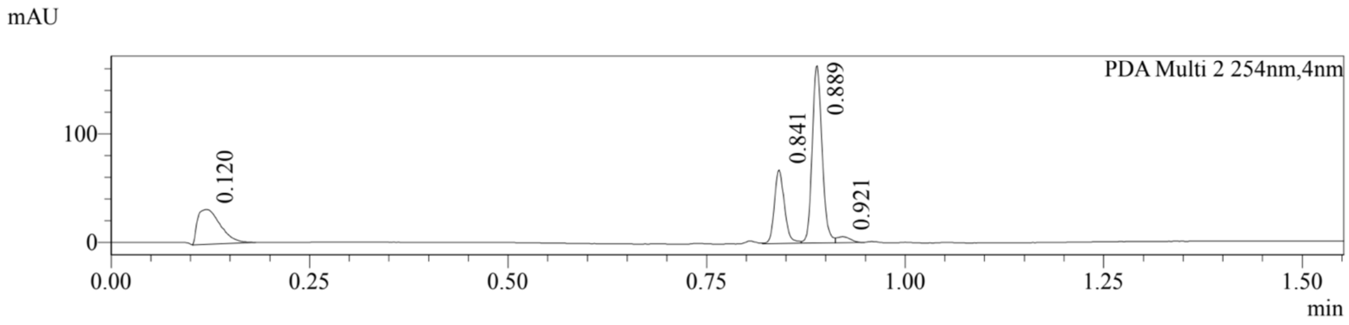

^{1}H NMR

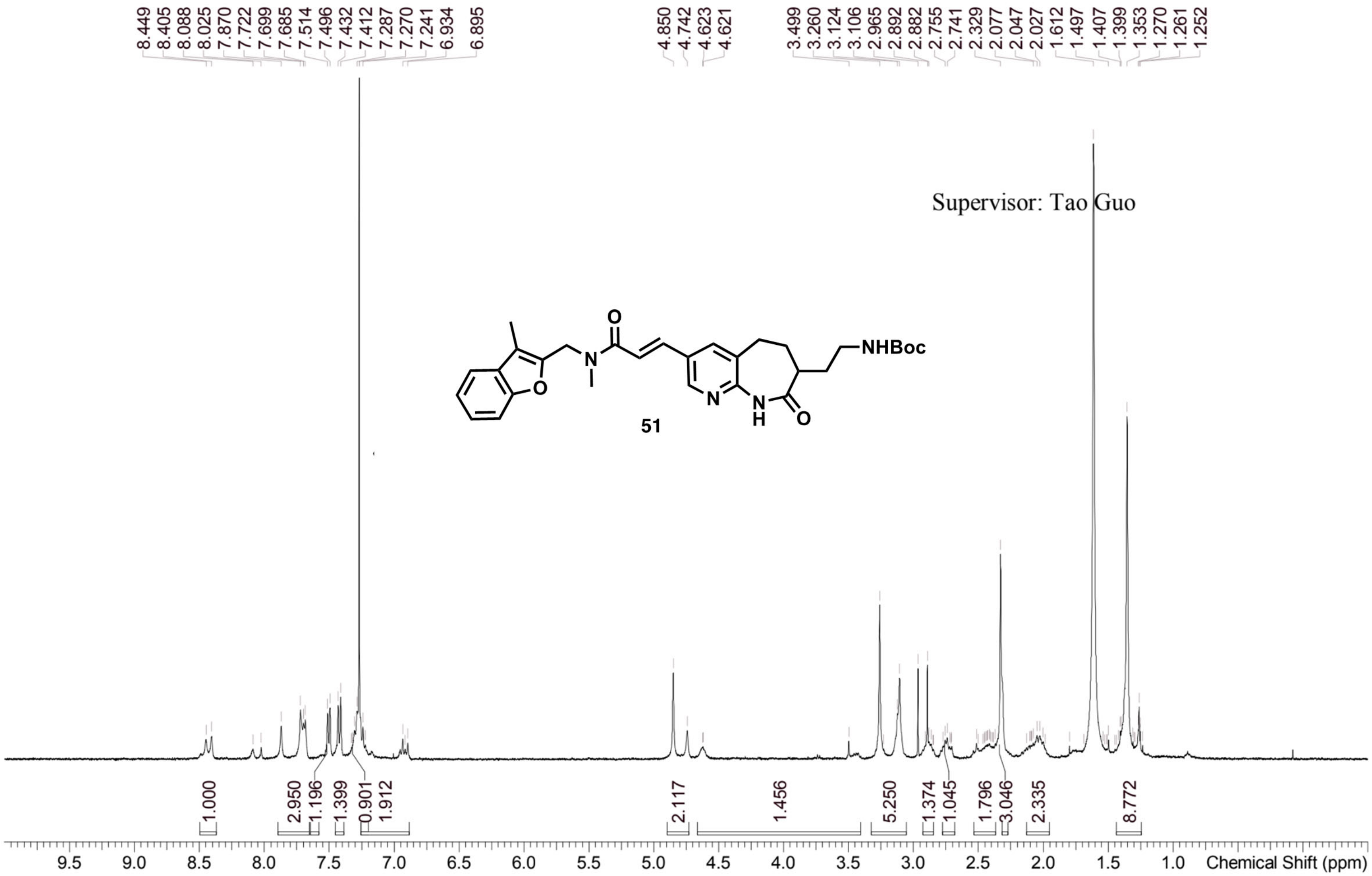

^{1}H NMR

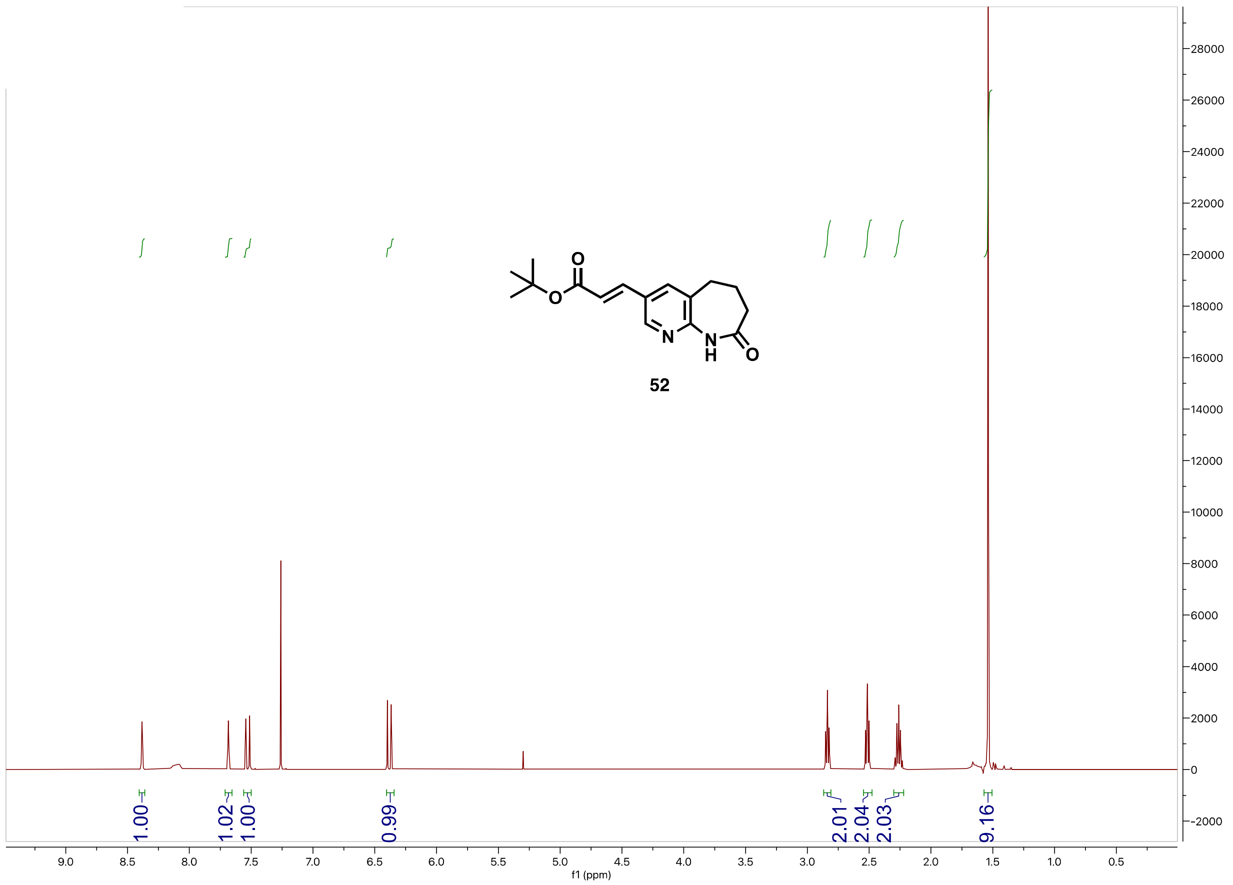

^{13}C NMR

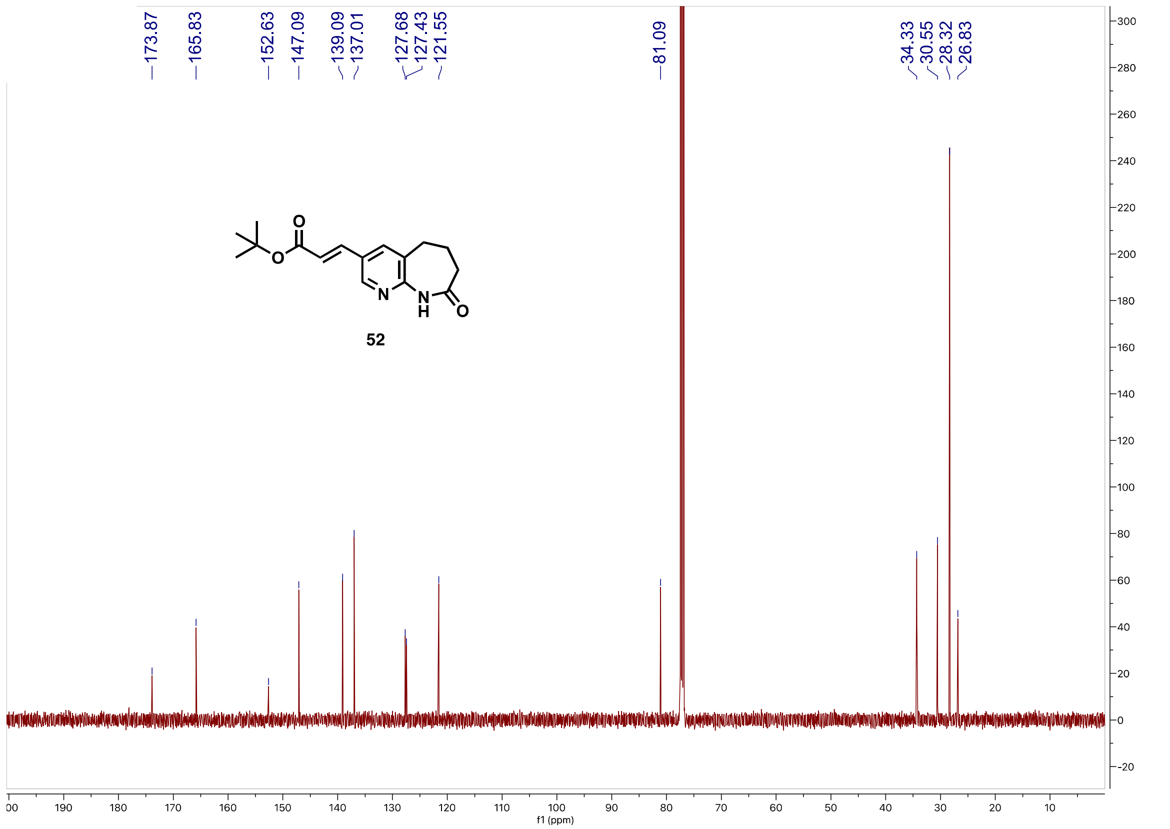

^{1}H NMR

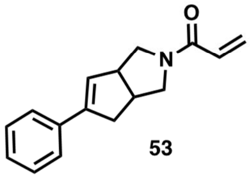

53

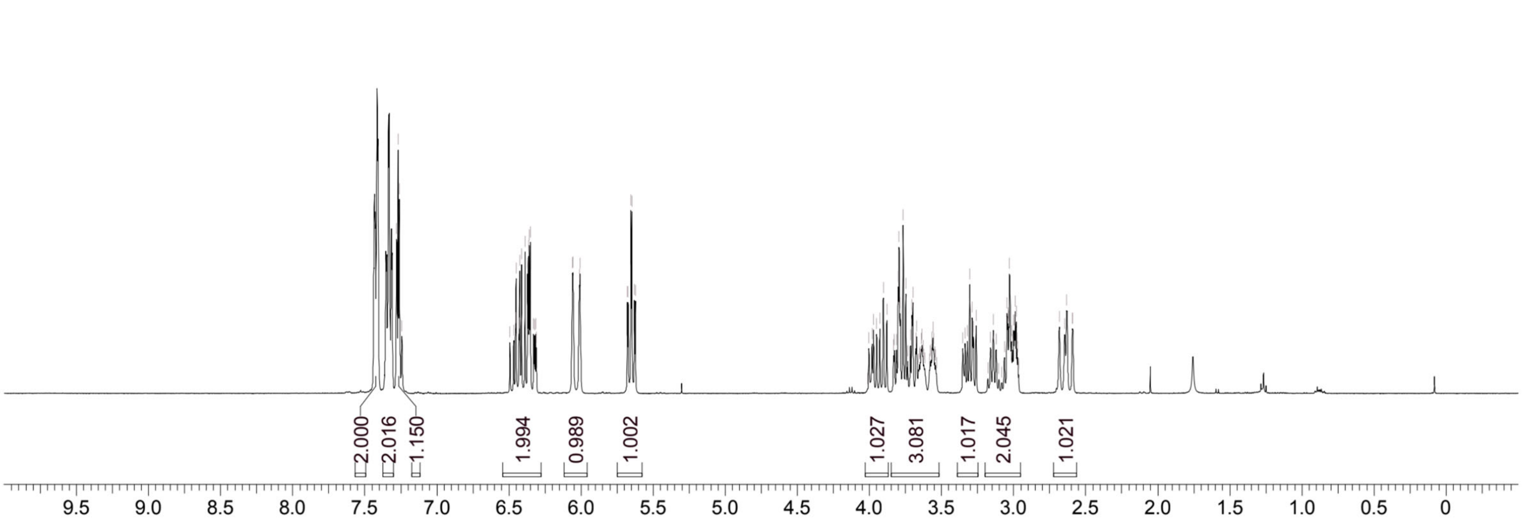
7.279
7.270
7.261
6.426
6.413
6.388
6.371
6.365
6.358
6.352
6.059
6.056
6.008
5.654
5.648
5.630
4.004
3.903
3.800
3.794
3.787
3.767
3.748
3.705
3.699
3.304
3.286
3.045
3.028
2.989
2.682
2.632
2.592
2.000
2.016
1.150
1.994
0.989
1.002
1.027
3.081
1.017
2.045
1.021
9.5
9.0
8.5
8.0
7.5
7.0
6.5
6.0
5.5
5.0
4.5
4.0
3.5
3.0
2.5
2.0
1.5
1.0
0.5
0

^{1}H NMR

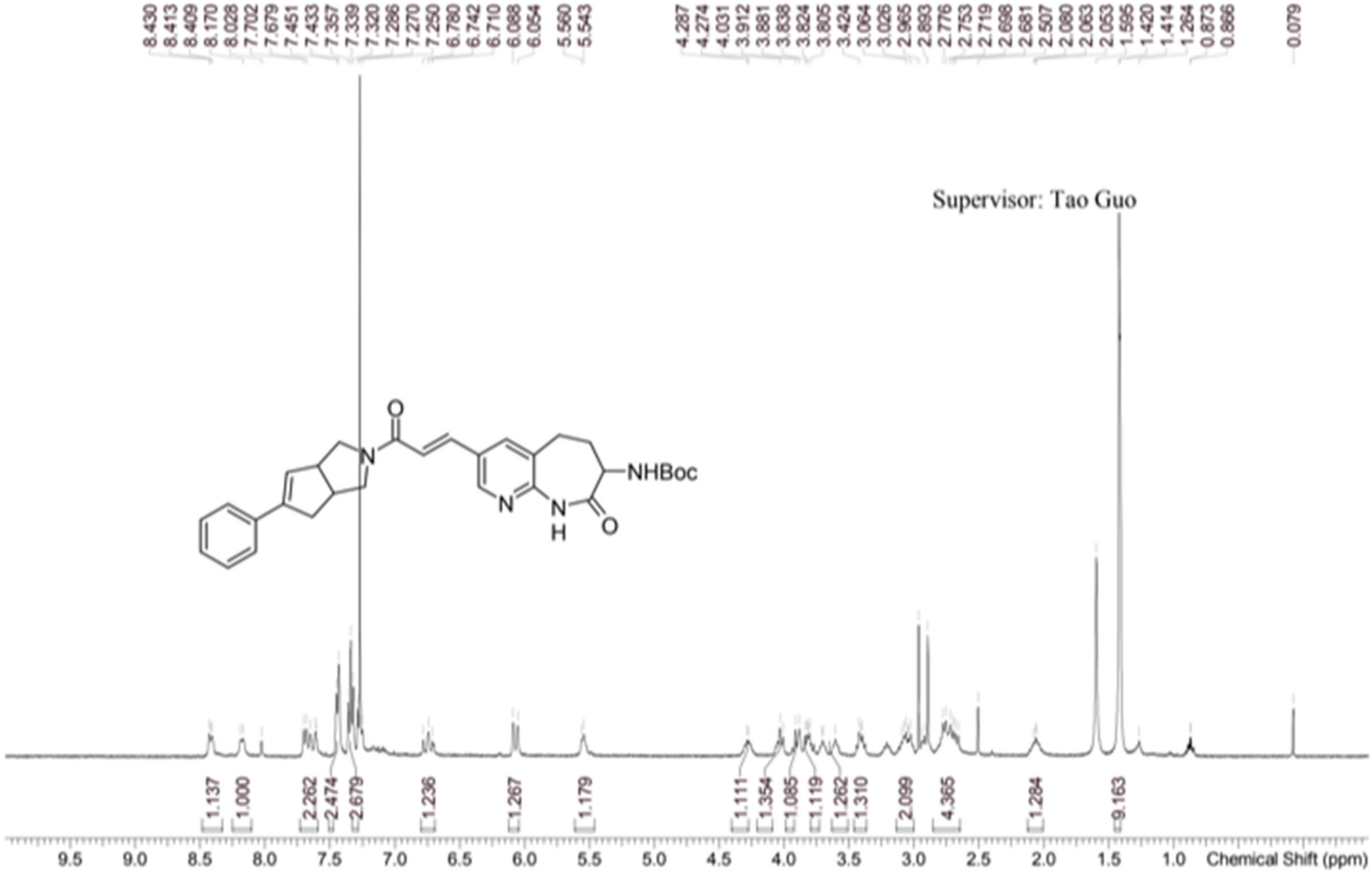

LCMS

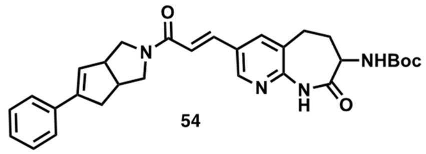

54

Chromatogram

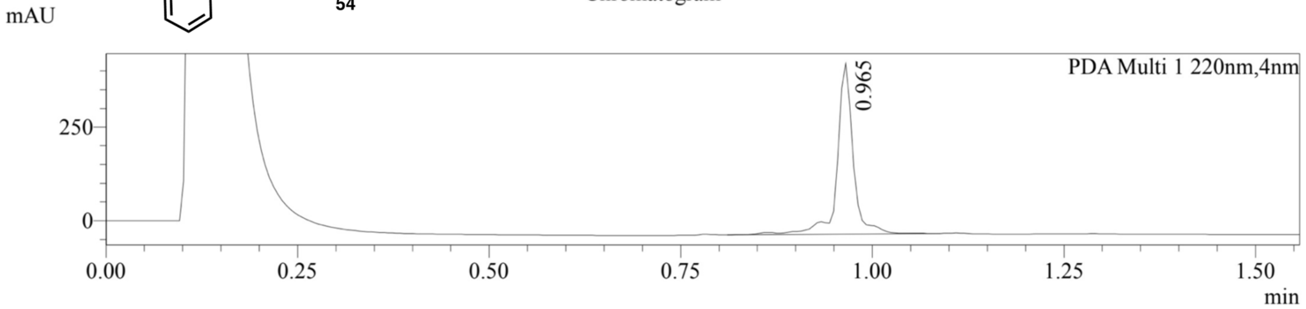

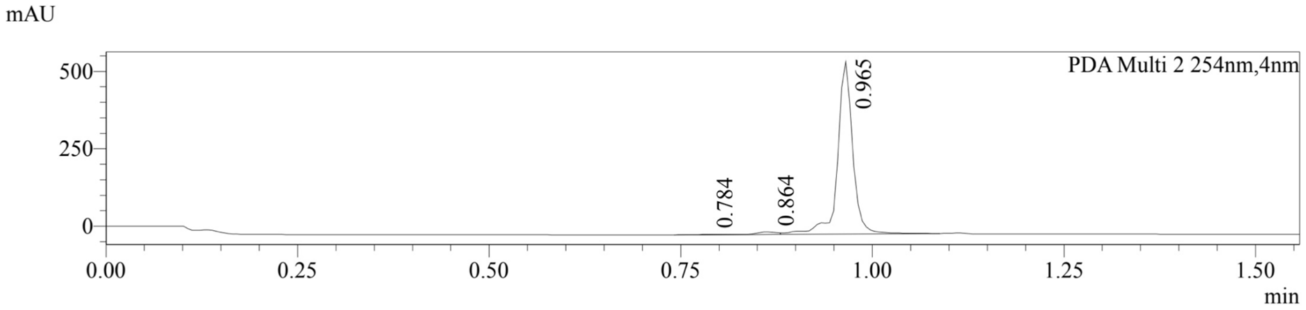

^{1}H NMR

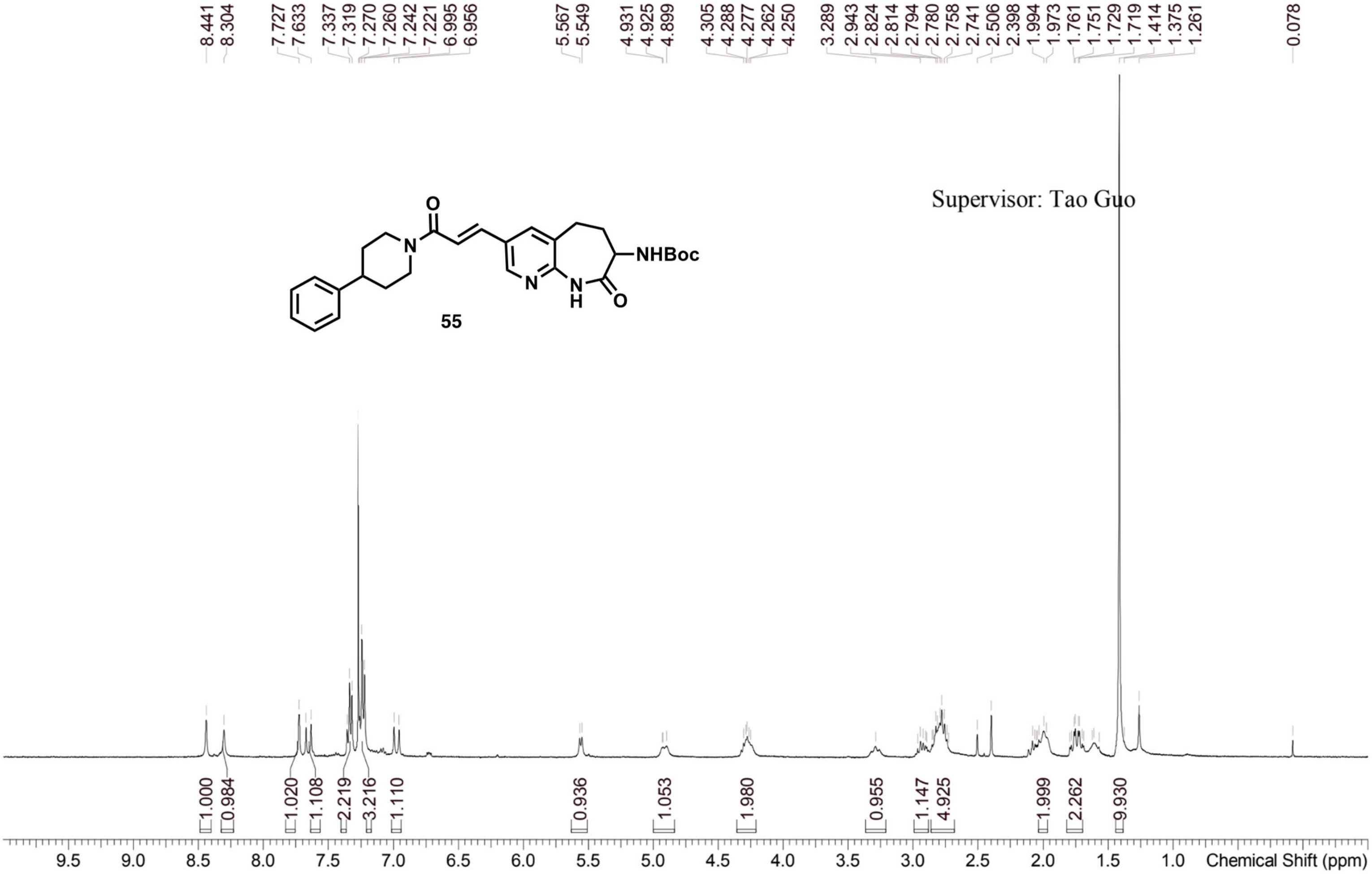

8.441 8.304 7.727 7.633 7.337 7.319 7.270 7.260 7.242 7.221 6.995 6.956 5.567 5.549 4.931 4.925 4.899 4.305 4.288 4.277 4.262 4.250 3.289 2.943 2.824 2.814 2.794 2.780 2.758 2.741 2.506 2.398 1.994 1.973 1.761 1.751 1.729 1.719 1.414 1.375 1.261 0.078
Supervisor: Tao Guo
NHBoc
55
1.000 0.984 1.020 1.108 2.219 3.216 1.110 0.936 1.053 1.980 0.955 1.147 4.925 1.999 2.262 9.930
9.5 9.0 8.5 8.0 7.5 7.0 6.5 6.0 5.5 5.0 4.5 4.0 3.5 3.0 2.5 2.0 1.5 1.0 Chemical Shift (ppm)

LCMS

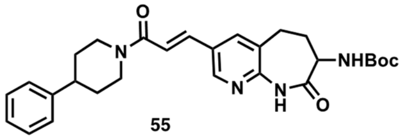

NHBoc
55

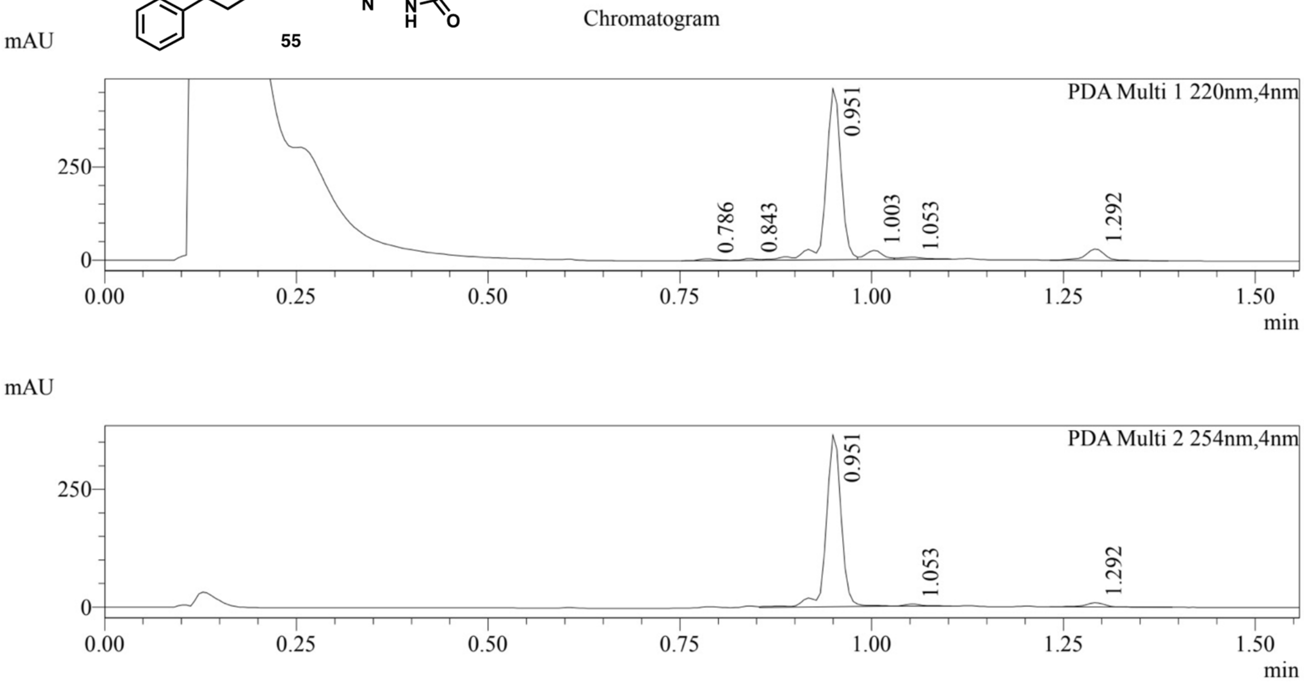

Chromatogram
mAU
PDA Multi 1 220nm,4nm
0.951
0.786
0.843
1.003
1.053
1.292
250
0
0.00 0.25 0.50 0.75 1.00 1.25 1.50
min
mAU
PDA Multi 2 254nm,4nm
0.951
1.053
1.292
250
0
0.00 0.25 0.50 0.75 1.00 1.25 1.50
min

REFERENCES

1. Higgins, P. G.; Stefanik, D.; Page, M. G.; Hackel, M.; Seifert, H., In vitro activity of the siderophore monosulfactam BAL30072 against meropenem-non-susceptible Acinetobacter baumannii. *J Antimicrob Chemother* **2012,** *67* (5), 1167-9.
2. Swenson, J. M.; Killgore, G. E.; Tenover, F. C., Antimicrobial susceptibility testing of Acinetobacter spp. by NCCLS broth microdilution and disk diffusion methods. *J Clin Microbiol* **2004,** *42* (11), 5102-8.
3. Parker, E. N.; Drown, B. S.; Geddes, E. J.; Lee, H. Y.; Ismail, N.; Lau, G. W.; Hergenrother, P. J., Implementation of permeation rules leads to a FabI inhibitor with activity against Gram-negative pathogens. *Nat Microbiol* **2020,** *5* (1), 67-75.
4. Geddes, E. J.; Li, Z.; Hergenrother, P. J., An LC-MS/MS assay and complementary web-based tool to quantify and predict compound accumulation in E. coli. *Nat Protoc* **2021,** *16* (10), 4833-4854.
5. Ling, L. L.; Schneider, T.; Peoples, A. J.; Spoering, A. L.; Engels, I.; Conlon, B. P.; Mueller, A.; Schaberle, T. F.; Hughes, D. E.; Epstein, S.; Jones, M.; Lazarides, L.; Steadman, V. A.; Cohen, D. R.; Felix, C. R.; Fetterman, K. A.; Millett, W. P.; Nitti, A. G.; Zullo, A. M.; Chen, C.; Lewis, K., A new antibiotic kills pathogens without detectable resistance. *Nature* **2015,** *517* (7535), 455-9.
6. Lee, H. Y.; Parkinson, E. I.; Granchi, C.; Paterni, I.; Panigrahy, D.; Seth, P.; Minutolo, F.; Hergenrother, P. J., Reactive Oxygen Species Synergize To Potently and Selectively Induce Cancer Cell Death. *ACS Chem Biol* **2017,** *12* (5), 1416-1424.
7. Palchaudhuri, R.; Lambrecht, M. J.; Botham, R. C.; Partlow, K. C.; van Ham, T. J.; Putt, K. S.; Nguyen, L. T.; Kim, S. H.; Peterson, R. T.; Fan, T. M.; Hergenrother, P. J., A Small Molecule that Induces Intrinsic Pathway Apoptosis with Unparalleled Speed. *Cell Rep* **2015,** *13* (9), 2027-36.
8. Llabani, E.; Hicklin, R. W.; Lee, H. Y.; Motika, S. E.; Crawford, L. A.; Weerapana, E.; Hergenrother, P. J., Diverse compounds from pleuromutilin lead to a thioredoxin inhibitor and inducer of ferroptosis. *Nat Chem* **2019,** *11* (6), 521-532.
9. Lin, F.; Xu, Y.; Chang, Y.; Liu, C.; Jia, X.; Ling, B., Molecular Characterization of Reduced Susceptibility to Biocides in Clinical Isolates of Acinetobacter baumannii. *Front Microbiol* **2017,** *8*, 1836.
10. Andes, D. R.; Lepak, A. J., In vivo infection models in the pre-clinical pharmacokinetic/pharmacodynamic evaluation of antimicrobial agents. *Curr Opin Pharmacol* **2017,** *36*, 94-99.
11. Zuluaga, A. F.; Salazar, B. E.; Rodriguez, C. A.; Zapata, A. X.; Agudelo, M.; Vesga, O., Neutropenia induced in outbred mice by a simplified low-dose cyclophosphamide regimen: characterization and applicability to diverse experimental models of infectious diseases. *BMC Infect Dis* **2006,** *6*, 55.
12. Bowen, S. E.; Watt, C. L.; Murawski, I. J.; Gupta, I. R.; Abraham, S. N., Interplay between vesicoureteric reflux and kidney infection in the development of reflux nephropathy in mice. *Dis Model Mech* **2013,** *6* (4), 934-41.
13. Ragnarsdottir, B.; Lutay, N.; Gronberg-Hernandez, J.; Koves, B.; Svanborg, C., Genetics of innate immunity and UTI susceptibility. *Nat Rev Urol* **2011,** *8* (8), 449-68.
14. Berman, J. M. S., P.; Pauls, H. W.; Ramnauth, J.; Manning, D. D.; Surman, M. D.; Xie, D.; Decornez, H. Y. Heterocyclic Compounds, Methods of Making Them and Their Use in Therapy US 7,790,709 B2, 2010.
